# Metal-free hydroarylation of the side chain carbon–carbon double bond of 5-(2-arylethenyl)-3-aryl-1,2,4-oxadiazoles in triflic acid

**DOI:** 10.3762/bjoc.13.89

**Published:** 2017-05-11

**Authors:** Anna S Zalivatskaya, Dmitry S Ryabukhin, Marina V Tarasenko, Alexander Yu Ivanov, Irina A Boyarskaya, Elena V Grinenko, Ludmila V Osetrova, Eugeniy R Kofanov, Aleksander V Vasilyev

**Affiliations:** 1 Department of Chemistry, Saint Petersburg State Forest Technical University, Institutsky per., 5, Saint Petersburg, 194021, Russia; 2 Yaroslavl State Technical University, Moskovskiy pr., 88, Yaroslavl, 150023, Russia; 3 Center for Magnetic Resonance, Research park, Saint Petersburg State University, Universitetskiy pr., 26, Saint Petersburg, Petrodvoretz, 198504, Russia; 4 Institute of Chemistry, Saint Petersburg State University, Universitetskaya nab., 7/9, Saint Petersburg, 199034, Russia; 5 Institute of Synthetic Rubber, Gapsalskaya str., 1, Saint Petersburg, 198035, Russia

**Keywords:** Friedel–Crafts reaction, hydroarylation, oxadiazoles, superelectrophilic activation, triflic acid

## Abstract

The metal-free reaction of 5-(2-arylethenyl)-3-aryl-1,2,4-oxadiazoles with arenes in neat triflic acid (TfOH, CF_3_SO_3_H), both under thermal and microwave conditions, leads to 5-(2,2-diarylethyl)-3-aryl-1,2,4-oxadiazoles. The products are formed through the regioselective hydroarylation of the side chain carbon–carbon double bond of the starting oxadiazoles in yields up to 97%. According to NMR data and DFT calculations, N^4^,C-diprotonated forms of oxadiazoles are the electrophilic intermediates in this reaction.

## Introduction

**Figure 1:**
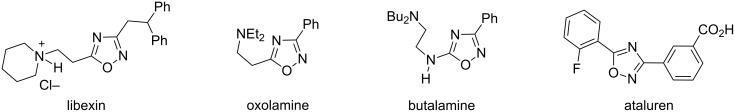
1,2,4-Oxadiazole-based drugs.

Oxadiazoles are an important class of heterocyclic compounds and great attention has been paid to their synthesis and to the studies of their chemical, physical and biological properties (see numerous reviews [1–8]). The oxadiazole ring represents an essential part of the pharmacophore in many drugs. These compounds possess different kinds of biological activities, such as analgesic [9], anti-inflammatory [10], antimicrobial [11], antidiabetic [12], and anticancer [13] to name a few. Some representatives of 1,2,4-oxadiazole-based drugs are shown in Figure 1. Libexin and oxolamine are used as antitussive (cough) agents [14], butalamine is a coronary vasodilator and local anesthetic [15], and ataluren finds application for the treatment of fibrosis [16]. Often, oxadiazole derivatives act as inhibitors of bacterial phenylalanyl-tRNA-synthetase [17], phosphodiesterase 4B2 [18], y-secretase [19] and phenol-substituted 1,2,4-oxadiazoles exhibit powerful anti-oxidant properties [20]. Moreover, they have antihypertensive [21] and antituberculosis [22] activities. In medicinal chemistry the oxadiazole ring is considered as bioisosteric replacements for ester or amide groups [23]. Thus, the further development of syntheses of 1,2,4-oxadiazoles and the investigation of their properties are of ongoing interest in chemistry and medicine.

**Scheme 1:**
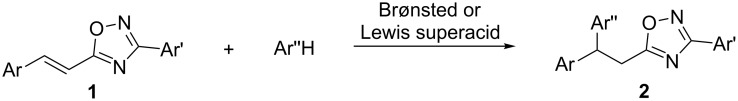
The hydroarylation of 5-(2-arylethenyl)-3-aryl-1,2,4-oxadiazoles **1** under superelectrophilic activation leading to compounds **2**.

Based on our previous works on reactions of cinnamides [24] and 5-styryl-2*H*-tetrazoles [25] with arenes under superelectrophilic activation with Brønsted or Lewis superacids, we turned our attention towards the hydroarylation of the C=C double bond in 5-styryl-substituted oxadiazoles, such as (*E*)*-*5-(2-arylethenyl)-3-aryl-1,2,4-oxadiazoles **1** (Scheme 1). The main goals of the current work were to investigate the reactions of oxadiazoles **1** with different arenes under the conditions of superelectrophilic activation and to study protonated forms of the oxadiazoles as reactive intermediates by means of NMR and DFT calculations.

It should be noted, that the metal-catalyzed hydroarylation of C=C bonds is widely used in organic synthesis [26–27]. The most efficient catalysts for these purposes are complexes of the transition metals Pt [28], Au [29], Ru [30], Rh [31–33], Ni [34], Pd [35], and Pd/Ag [36]. However, a metal-free hydroarylation variant of C=C bonds under the action of Brønsted or Lewis superacids has been developed [37–38] and we were able to extend the scope of this reaction [24–25].

**Figure 2:**
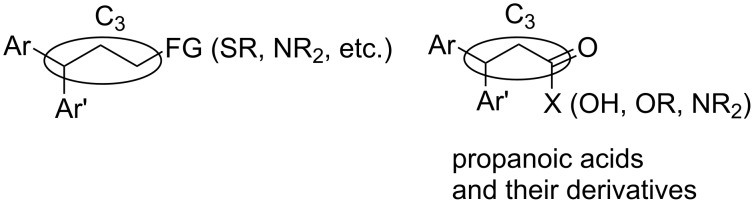
General structure for various biologically active compounds containing three carbon atoms, two aryl rings and functional groups.

The expected reaction products, oxadiazoles **2** (Scheme 1) are structurally close to many biologically active compounds and drugs [39–49], which contain a chain of three carbon atoms, two aryl rings on one end of this chain, and further functional groups on the other end of it (Figure 2, compare also with libexin in Figure 1). One may suppose that compounds of type **2** having the same structural fragments, i.e., three carbon atoms (one of them part of the oxadiazole moiety), two aryl groups, and the four remaining atoms of the oxadiazole ring, as a functional group, may also show biological activity. Thus, the synthesis of these particular 5-(2,2-diarylethyl)-substituted oxadiazoles **2** may be interesting for medicinal chemistry.

## Results and Discussion

According to literature data [50–51] the protonation of a 1,2,4-oxadiazole ring takes place mainly at the N^4^ nitrogen. However, also the N^2^ nitrogen may be protonated depending on the substituents attached to the heterocyclic system [50]. To investigate this issue in more detail we undertook a theoretical study on the protonation of 5-(2-phenylethenyl)-3-phenyl-1,2,4-oxadiazole (**1a**) by quantum-chemical calculations. Table 1 contains data obtained by DFT calculations for the different possible mono-, di- and tricationic species **A–F** derived from the protonation of **1a**. Charge distributions, contributions of the atomic orbital into LUMO, global electrophilicity indices ω [52–53], and the Gibbs free energies Δ*G*_298_ of the protonation reactions with the hydroxonium ion (H_3_O^+^) were calculated.

**Table 1:** Selected electronic characteristics for cations **A**–**F** calculated by DFT from protonation of oxadiazole **1a**.

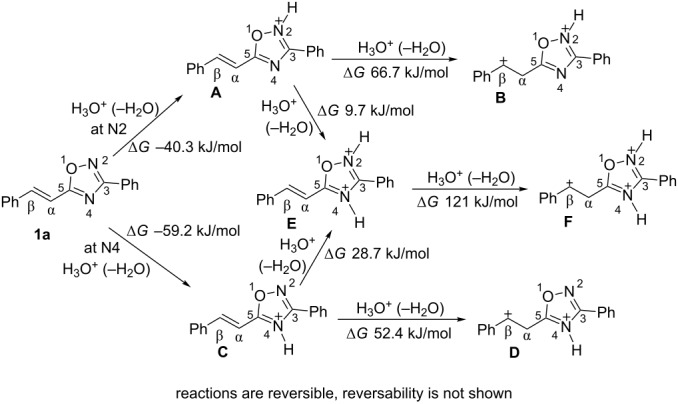							

Species	*E*_HOMO_, eV	*E*_LUMO_, eV	ω^a^, eV	q (C^β^)^b^, e	*k* (C^β^)_LUMO_^c^, %	q(N^2^)^b^, e	q(N^4^)^b^, e

**A**	−7.10	−3.49	3.87	−0.04	17.2	−0.19	−0.52
**B**	−7.95	−4.98	7.02	0.20	27.0	−0.17	−0.49
**C**	−7.18	−3.43	3.75	−0.02	26.2	−0.15	0.52
**D**	−7.83	−5.06	7.48	0.18	37.3	−0.12	−0.49
**E**	−7.72	−4.32	5.32	0.03	21.4	−0.16	−0.5
**F**	−8.46	−5.24	7.30	0.16	34.7	−0.15	−0.48

^a^Global electrophilicity index ω = (*E*_HOMO_ + *E*_LUMO_)^2^/8 (*E*_LUMO_ − *E*_HOMO_). ^b^Natural charges. ^c^Contribution of atomic orbital into the molecular orbital.

Negative values for Δ*G*_298_ of the first protonation step show that this reaction is energetically favorable at both N^2^ (formation of species **A**) and N^4^ (formation of species **C**). A further protonation may occur at the second nitrogen atom of the oxadiazole system (formation of dication **E**) or at the carbon C^α^ of the side chain C=C double bond (formation of dications **B** or **D**). Finally, the third protonation giving rise to trication **F** is rather high in energy (Δ*G*_298_ 121 kJ/mol for **E**→**F**) and therefore highly unlikely. Also the outlined route leading to species **B** is energetically unfavorable (Δ*G*_298_ = −40.3 + 66.7 = 26.4 kJ/mol) and the formation of this dication is not expected. In contrast, the generation of dication **D** should be possible (Δ*G*_298_ = −59.2 + 52.4 = −6.8 kJ/mol). Despite more negative values of Δ*G*_298_ calculated for the formation of the N^2^,N^4^-diprotonated form **E** from both **A** (Δ*G*_298_ = −40.3 + 9.7 = −30.6 kJ/mol for **1a**→**A**→**E**) and **C** (Δ*G*_298_ = −59.2 + 28.7 = −30.5 kJ/mol for **1a**→**C**→**E**), it was found by calculations, that species **E** had an imaginary frequency revealing that it may be a transition state rather than an intermediate species.

Thus, from a thermodynamic point of view, the first protonation of 5-styryl-substituted 1,2,4-oxadiazoles takes place at the N^4^ nitrogen atom leading to cation **C** (Δ*G*_298_ of this reaction has the lowest value of −59.2 kJ/mol) and the most probable dicationic species, obtained through protonation of species **C**, should be N^4^,C-diprotonated form **D**.

The calculated electronic characteristics of species **A**–**F** revealed that the dication **D** has the highest electrophilicity index ω (7.48 eV) among the other cationic species, even including trication **F** (Table 1). Therefore, dication **D** is expected to be an extremely reactive electrophile. Moreover, it has a large portion of the positive charge (0.18 e) located at the C^β^ carbon atom and a high contribution to the LUMO (37.3%), thus making this carbon atom a reactive electrophilic center through charge and orbital control. One may not exclude that N^2^- and N^4^-monoprotonated forms **A** and **C**, respectively, also may behave as electrophiles. However, the calculated negative charges on the C^β^ carbon atoms may prevent a chemical transformation on them.

Summarizing the calculation results, one may conclude that the most probable reactive electrophilic species, derived through the protonation of 5-styryl-substituted 1,2,4-oxadiazoles, is the N^4^,C-diprotonated species **D** from both the thermodynamic and electronic point of view.

**Figure 3:**
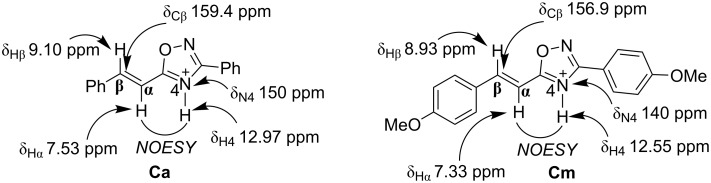
Selected ^1^H, ^13^C, ^15^N NMR data for cations **Ca** and **Cm** generated by protonation of oxadiazoles **1a** and **1m** at the N^4^ nitrogen (FSO_3_H, −80 °C for **Ca**, and −60 °C for **Cm**, with CH_2_Cl_2_ as internal standard).

Next, we carried out an NMR study of the protonation of oxadiazoles **1a** and **1m** in the superacid FSO_3_H at low temperature (−80 °C). The spectra are shown in Supporting Information File 1. Upon dissolving compounds **1a** and **1m** in fluorosulfonic acid, FSO_3_H in an NMR tube at −80 °C, the formation of N^4^-protonated species **Ca** and **Cm**, respectively, was detected (Figure 3). The proton bounded to nitrogen N^4^ resonates at δ ≈ 12.5–13 ppm at this temperature whereas at higher temperature (above −40 °C) this signal disappeared due to fast proton exchange with the superacidic medium (see Supporting Information File 1). The addition of the proton at the N^4^ position, rather than at N^2^, was proved by the NOESY correlation between this proton and the vinyl proton H^α^ (Figure 3). In the case of a protonation at N^2^, there should not be any correlation between the proton attached to N^2^ and H^α^. The assignment of all signals in the ^1^H and ^13^C NMR spectra of cations **Ca** and **Cm** was undertaken on the basis of ^1^H–^13^C HSQC and ^1^H–^15^N HSQC spectra (see spectra in Supporting Information File 1). According to the NMR study and preparative experiments, the increasing reaction temperature in trifluoromethanesulfonic acid (TfOH, to 60 °C for **1a**, and to room temperature for **1m**) led to the formation of oligomeric material. Most likely, at higher temperature the second protonation at the C=C bond takes place giving rise to the corresponding unstable highly reactive dications **D**, which were not detected by NMR.

It should be mentioned, that there have been reports [37,54–57] on NMR observations of O,C-diprotonated forms (dications) of conjugated enones in superacidic medium, which are structurally close to species **D**.

Thus, the NMR data reveal that the protonation of 5-styryl-substituted 1,2,4-oxadiazoles in superacids results in the formation of their relatively stable N^4^-protonated forms. However, these species do not react with aromatic π-nucleophiles (vide infra). Most probably, those reactive intermediates, generated under the protonation of substrates **1**, are N^4^,C-diprotonated species **D**.

**Figure 4:**
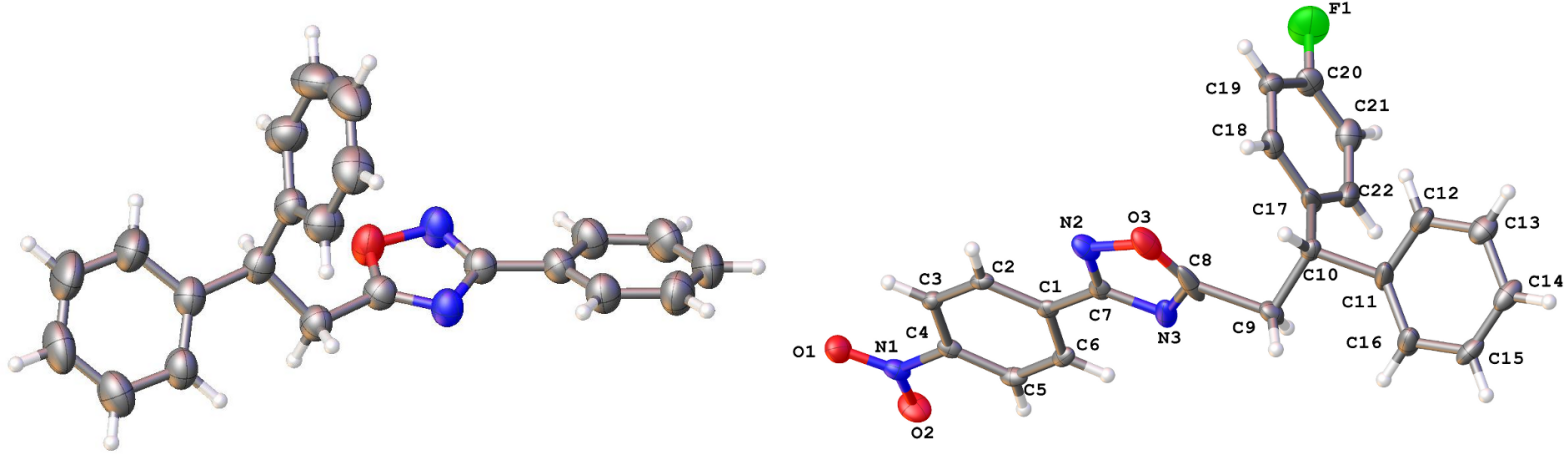
X-ray crystal structures of compounds **2a** (left) (CCDC 1526767) and **2m** (right) (CCDC 1526105); ellipsoid contours of probability levels are 50%.

The experimental results from the hydroarylation reactions of the side chain C=C double bond of oxadiazoles **1a–n** with various arenes under the action of different acidic reagents leading to oxadiazoles **2a–za** are shown in Table 2 (see also X-ray structures of **2a** and **2m** in Figure 4). First, it should be emphasized that no reaction is observed under the conditions of generation of monoprotonated species **C** (see Table 1, and Figure 3). Thus, in FSO_3_H at low temperature (−80 to −60 °C) compounds **1e** (Table 2, entries 14 and 15) and **1m** (Table 2, entry 29) do not react with benzene. At higher temperature, fluorosulfonation of the aromatic ring is observed, especially with donating arenes (see example of this reaction in our work [24]). On the other hand, compounds **1e** and **1m** readily react with benzene in TfOH at room temperature (see Table 2, entries 16 and 28). Presumably, at higher temperatures the second protonation takes place at the C=C double bond giving rise to reactive dications **D**. The acidity of H_2_SO_4_, which is lower than that of FSO_3_H and TfOH, is not sufficient to promote this reaction. Thus, the protonation of the C=C bond of **1a** in H_2_SO_4_ does not take place even at elevated temperature (75 °C, see Table 2, entry 1). Also, Lewis acids such as AlCl_3_ and AlBr_3_ are not effective in this transformation (Table 2, entries 2 and 3). The best results were obtained in neat TfOH.

**Table 2:** Hydroarylation of oxadiazoles **1a–n** with arenes under superelectrophilic activation leading to compounds **2a–za**.

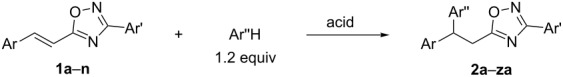				

Entry	Starting materials		Reaction conditions	Reaction products **2**, yield (%)^a^

	Oxadiazole **1**	Arene, Ar”H		

1	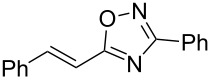 **1a**	benzene	H_2_SO_4_, 75 °C, 24 h	**1a**^b^
2	**1a**	benzene	AlCl_3_, CH_2_Cl_2_, rt, 24 h	**1a**^b^
3	**1a**	benzene	AlBr_3_, 60 °C, 3 h	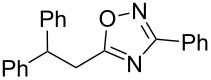 **2a** (60%)
4	**1a**	benzene	TfOH, 60 °C, 2 h	**2a** (77%)
5	**1a**	benzene	TfOH, rt, 18 h	**2a** (80%)
6	**1a**	chlorobenzene	TfOH, rt, 18 h	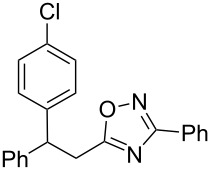 **2b** (84%) 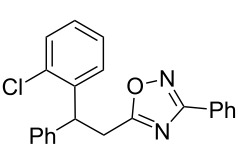 **2c** (12%)
7	**1a**	1,2-dichloro- benzene	TfOH, rt, 24 h	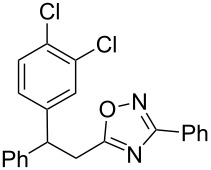 **2d** (60%) 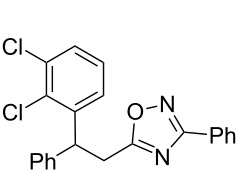 **2e** (5%)
8	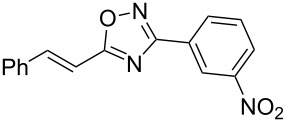 **1b**	benzene	TfOH, 60 °C, 2 h	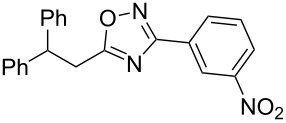 **2f** (89%)
9	**1b**	benzene	TfOH-SbF_5_ (20 mol %), rt, 0.5 h	**2f** (64%)
10	**1b**	*tert*-butyl- benzene	TfOH, 60 °C, 2 h	**2f** (96%)
11	**1b**	anisole	TfOH, 60 °C, 2 h	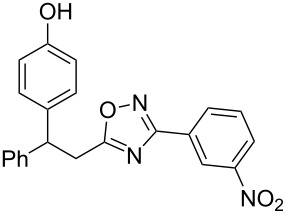 **2g** (39%)
12	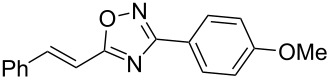 **1c**	benzene	TfOH, rt,12 h	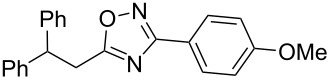 **2h** (80%)
13	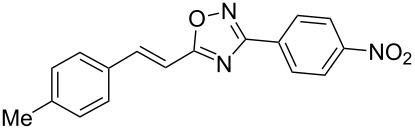 **1d**	benzene	TfOH, rt, 2 h	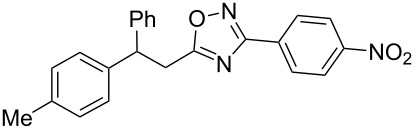 **2i** (60%) 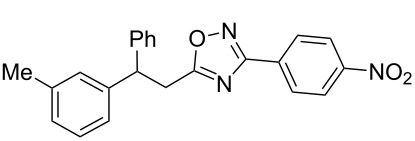 **2j** (31%)
14	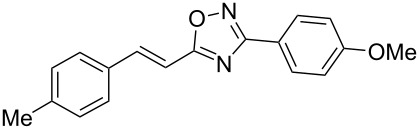 **1e**	benzene	FSO_3_H, −80 °C, 2 h	**1e**^b^
15	**1e**	benzene	FSO_3_H, −60 °C, 3 h	**1e**^b^
16	**1e**	benzene	TfOH, rt, 1 h	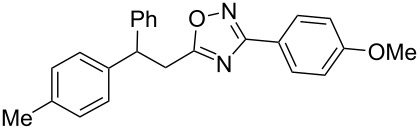 **2k** (90%)
17	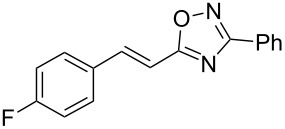 **1f**	benzene	TfOH, 60 °C, 3 h	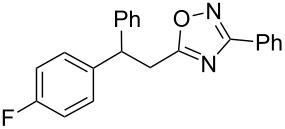 **2l** (93%)
18	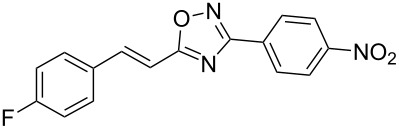 **1g**	benzene	TfOH, rt, 52 h	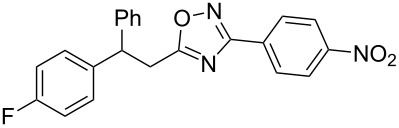 **2m** (91%)
19	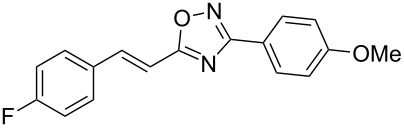 **1h**	benzene	TfOH, rt, 24 h	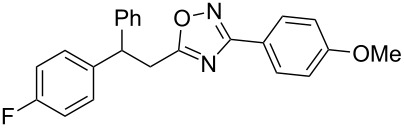 **2n** (90%)
20	**1h**	anisole	TfOH, rt, 24 h	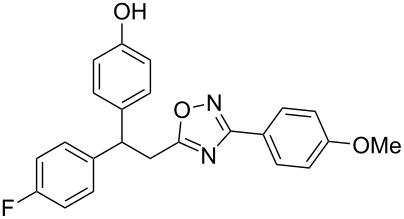 **2o** (50%) 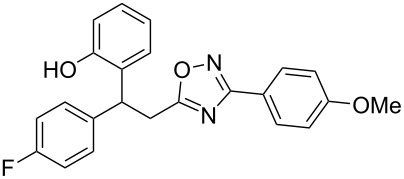 **2p** (25%)
21	**1h**	chlorobenzene	TfOH, rt, 24 h	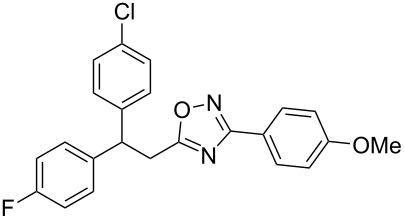 **2q** (50%) 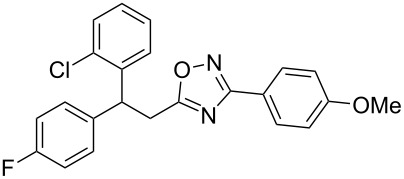 **2r** (8%)
22	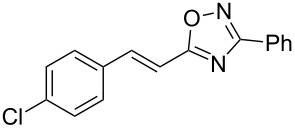 **1i**	benzene	TfOH, rt, 20 h	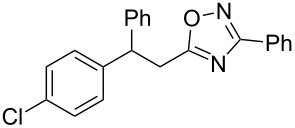 **2s** (94%)
23	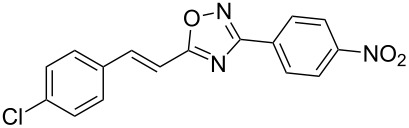 **1j**	benzene	TfOH, rt, 24 h	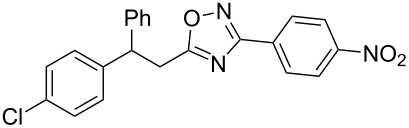 **2t** (97%)
24	**1j**	toluene	TfOH, rt, 24 h	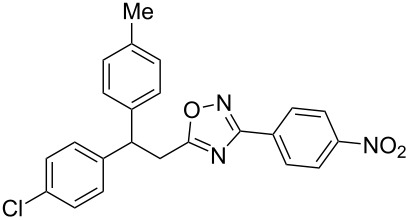 **2u** (60%) 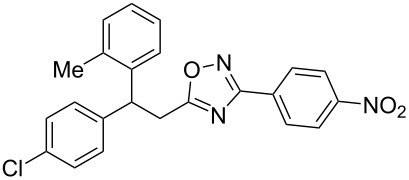 **2v** (5%)
25	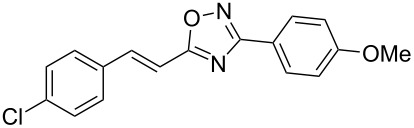 **1k**	benzene	TfOH, rt, 12 h	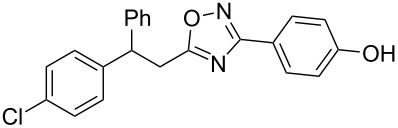 **2w** (95%)
26	**1k**	toluene	TfOH, rt, 12 h	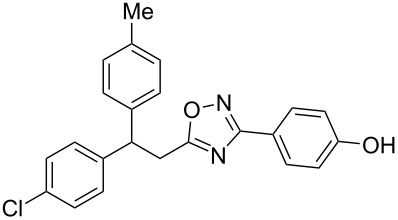 **2x** (67%)
27	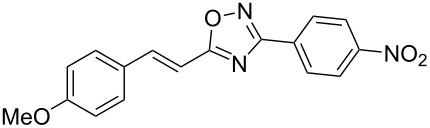 **1l**	benzene	TfOH, rt, 2 h	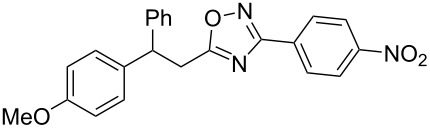 **2y** (92%)
28	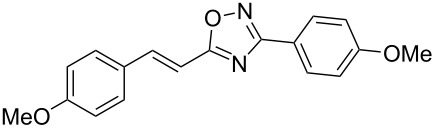 **1m**	benzene	TfOH, rt, 2 h	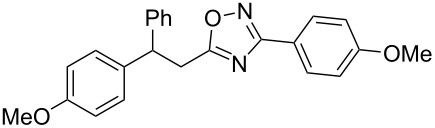 **2z** (50%)
29	**1m**	benzene	FSO_3_H, −80 °C, 2 h	**1m**^b^
30	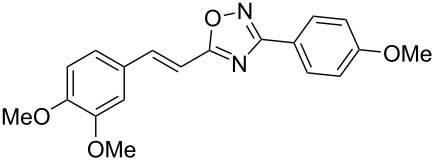 **1n**	benzene	TfOH, rt, 1 h	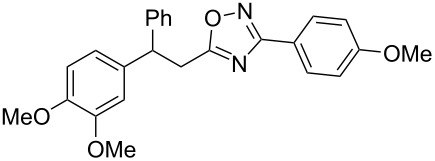 **2za** (53%)

^a^Isolated yields. ^b^Quantitative recovery of unreacted starting oxadiazole.

The substituents present in the aromatic ring of the styryl group of oxadiazoles **1** play a crucial role for the protonation and reactivity of these compounds. Thus, styryl-substituted oxadiazoles **1b**,**c** and substrates **1f–k**, bearing electron-accepting halogen substituents, need higher reaction temperatures up to 60 °C (Table 2, entries 4, 8, 10, 11, and 17) or longer reaction times of 24–52 h (Table 2, entries 18–24) at rt in TfOH. On the other hand, electron-donating groups attached to the styryl moiety of oxadiazoles **1d**,**e**,**l–n** facilitate the protonation of the C=C double bond, resulting in a reduced reaction time to 1–2 h at room temperature (Table 2, entries 13, 16, 27, 28, and 30). Increasing the acidity of the reaction medium promotes the protonation of deactivated oxadiazoles. Thus, compound **1b** in the system TfOH-SbF_5_ (20 mol %) reacted with benzene within 0.5 h at room temperature (Table 2, entry 9), but in less acidic neat TfOH the reaction took 2 h at 60 °C (Table 2, entry 8).

Different arenes may be involved in this reaction. The corresponding hydroarylation products were obtained by reaction with benzene, chlorobenzene, 1,2-dichlorobenzene, toluene, and anisole. Electron-rich polymethylated aromatics, such as isomeric xylenes, mesitylene, pseudocumene, or durene gave mixtures of oligomeric products. Probably, these products are formed through multiple electrophilic substitution reactions of these arenes by the reactive dication species **D**. When oxadiazole **1b** reacted with *tert*-butylbenzene (Table 2, entry 10), product **2f** lacking the *tert*-butyl group was isolated. In this case, an *ipso*-substitution of the *tert*-butyl group by a proton under the superacidic conditions took place. Reactions with some arenes gave regioisomeric products, for instance, anisole (**2o + 2p**, Table 2, entry 20), 1,2-dichlorobenzene (**2d + 2e**, Table 2, entry 7), chlorobenzene (**2b + 2c**, Table 2, entry 6 and **2q + 2r**, entry 21), and toluene (**2u + 2v**, Table 2, entry 24). The exact structures of these regioisomers were determined on the basis of multiplet signals of the aromatic protons in the ^1^H NMR spectra. The observation of regioisomeric products points out the high reactivity of the intermediate dicationic species **D**. The formation hydroxy-substituted oxadiazoles **2g** (Table 2, entry 11), **2o** and **2p** (Table 2, entry 20), **2w** (Table 2, entry 25), and **2x** (Table 2, entry 26) may be explained by demethylation of the corresponding methoxy group under action of TfOH at elevated temperature (60 °C) or for prolonged reaction times (12 or 24 h) at room temperature. See reviews [58–59] on the dealkylation of ethers by various Brønsted and Lewis acids.

Additionally, the reactions were carried out under microwave (MW) irradiation (Table 3) analogously to our recent study on the hydroarylation of styryl tetrazoles [25]. Indeed, under MW activation the reactions in TfOH proceeded within 5–20 min at 120 °C (Table 3, entries 4–9) with formation of oxadiazoles **2** in high yields (compare the yields under thermal and MW heating in Table 3). The MW-activated process without any acid (Table 3, entry 1) or in a weaker acid (H_2_SO_4_, Table 3, entry 2) did not proceed at all. Apart from that, the conversion of **1a** in the presence of AlCl_3_ was rather low (Table 3, entry 3). It should be emphasized that the oxadiazole ring is stable under the superacidic conditions and no destruction was noticed.

**Table 3:** Hydroarylation of oxadiazoles **1** with arenes under microwave (MW) activation in TFOH at 120 °C.

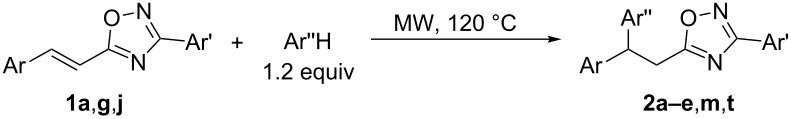					

Entry	Starting materials		Reaction conditions	Reaction products, yield (%), MW irradiation^a^	Conditions, yield (%), conventional heating

	Oxadiazole **1**	Arene, Ar”H			

1	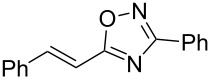 **1a**	benzene	15 min (without any acid)	**1a**^b^	
2	**1a**	benzene	H_2_SO_4_, 15 min	**1a**^b^	H_2_SO_4_, 75 °C, 24 h: **1a**^b,c^
3	**1a**	benzene	AlCl_3_, CH_2_Cl_2_, 30 min	**1a** (80%) 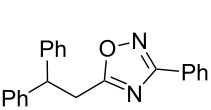 **2a** (8%)	AlCl_3_, CH_2_Cl_2_, rt, 24 h: **1a**^b,c^
4	**1a**	benzene	TfOH, 10 min	**2a** (92%)	TfOH, 60 °C, 2 h: **2a** (77%)^c^
5	**1a**	chloro- benzene	TfOH, 20 min	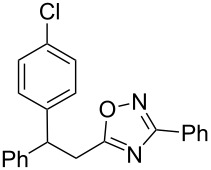 **2b** (70%) 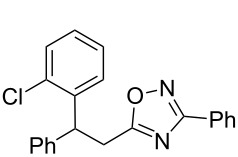 **2c** (24%)	TfOH, rt, 18 h: **2b** (84%) + **2c** (12%)^c^
6	**1a**	1,2-dichloro- benzene	TfOH, 20 min	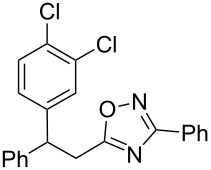 **2d** (50 %) 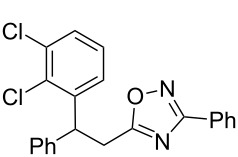 **2e** (20%)	TfOH, rt, 18 h: **2d** (60%) + **2e** (5%)^c^
7	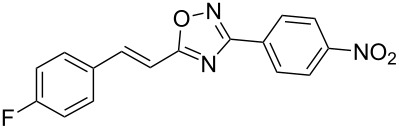 **1g**	benzene	TfOH, 10 min	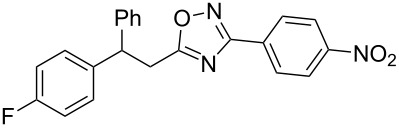 **2m** (94%)	TfOH, rt, 52 h: **2m** (91%)^c^
8	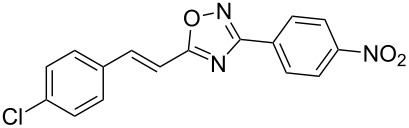 **1j**	benzene	TfOH, 5 min	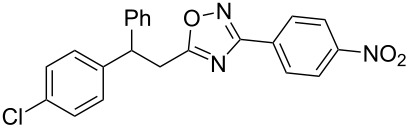 **2t** (76%)	TfOH, rt, 24 h: **2t** (97%)^c^ TfOH, 120 °C, 5 min: **2t** (95%)^d^

^a^Isolated yields. ^b^Quantitative recovery of unreacted starting oxadiazole. ^c^Data from Table 2. ^d^Reaction was carried out in glass high pressure tube.

## Conclusion

We have developed an efficient method for the hydroarylation of the C=C double bond of 5-(2-arylethenyl)-3-aryl-1,2,4-oxadiazoles based on their TfOH-promoted reaction with arenes under thermal or microwave activation to form 5-(2,2-diarylethyl)-3-aryl-1,2,4-oxadiazoles in high yields. The reactive electrophilic intermediates of this hydroarylation process are N^4^,C-diprotonated forms of the starting oxadiazoles.

## Supporting Information

File 1 Experimental part, NMR spectra and DFT calculations.
