# Supplementary material for: Metal-free hydroarylation of the side chain carbon–carbon double bond of 5-(2-arylethenyl)-3-aryl-1,2,4-oxadiazoles in triflic acid
Source: Beilstein J Org Chem. 2017 May 11;13:883–94. doi: 10.3762/bjoc.13.89 (PMC5433146; doi:10.3762/bjoc.13.89)

Supporting Information

for

Metal-free hydroarylation of the side chain carbon–carbon double bond of 5-(2-arylethenyl)-3-aryl-1,2,4-oxadiazoles in triflic acid

Anna S. Zalivatskaya[1], Dmitry S. Ryabukhin[1], Marina V. Tarasenko[2], Alexander Yu. Ivanov[3], Irina A. Boyarskaya[4], Elena V. Grinenko[1], Ludmila V. Osetrova[5], Eugeniy R. Kofanov[2], and Aleksander V. Vasilyev[1,4,§,*]

Address: [1]Department of Chemistry, Saint Petersburg State Forest Technical University, Institutsky per., 5, Saint Petersburg, 194021, Russia, [2]Yaroslavl State Technical University, Moskovskiy pr., 88, Yaroslavl, 150023, Russia, [3]Center for Magnetic Resonance, Research park, Saint Petersburg State University, Universitetskiy pr., 26, Saint Petersburg, Petrodvoretz, 198504, Russia, [4]Institute of Chemistry, Saint Petersburg State University, Universitetskaya nab., 7/9, Saint Petersburg, 199034, Russia and [5]Institute of Synthetic Rubber, Gapsalskaya str., 1, Saint Petersburg, 198035, Russia

Email: A.V. Vasilyev - aleksvasil@mail.ru

*Corresponding author:

[§]Tel.: 07 812 670 93 52; fax: 07 812 670 93 90

Experimental part, NMR spectra and DFT calculations

Contents

1. Experimental Part. Preparation and characterization of compounds S2
2. NMR spectra (^{1}H, ^{13}C,^{19}F, NOESY, HSQC) for compounds **1**, **2** and cations **C** S13
3. Data on DFT calculations of **1a** and cations **A, B, C, D, E, F** S57

1. Experimental Part. Preparation and characterization of compounds

1.1. General

NMR spectra were recorded on a Bruker AM 500 spectrometer (at 500 MHz, at 125 MHz, and at 470 MHz for ^{1}H, ^{13}C, and ^{19}F NMR spectra, respectively) using $CDCl_3$, $(CD_3)_2CO$, DMSO-d_6 for compounds **1** and **2**. The ^{1}H and ^{13}C spectra were calibrated using the residual signals of non-deuterated solvents as internal reference. ^{19}F NMR spectra were referenced through the solvent lock (2H) signal according to IUPAC recommended secondary referencing method and the manufacturer's protocols and the chemical shifts are reported relative to $CFCl_3$ (δ 0.0 ppm). Spectra of protonated forms **C** of oxadiazoles **1** in FSO_3H with CH_2Cl_2 as internal standard (δ_H 5.32 ppm, and δ_C 53.0 ppm) were recorded on a Bruker Avance III 400 spectrometer (at 400 MHz and 100 MHz for ^{1}H and ^{13}C NMR spectra, respectively). High resolution mass spectra (HRMS) were obtained using a Varian 902-MS MALDI Mass Spectrometer with 9.4 Tesla superconducting magnet equipped with a UV laser (Nd) in the positive ion mode or with a Bruker maXis HRMS-ESI-QTOF. GC–MS data were obtained with a G2570A GC/MSD Agilent Technologies 6850c equipped with a column HP-5MS (3m × 0.25mm), a thickness of the stationary phase of 0.25μm. Microwave reactions were carried out with a DISCOVER SP. The preparative reactions were monitored by thin layer chromatography on silica gel plates (Silufol UV-254) using UV light for detection. Column chromatography was performed on silica gel Chemapol 40/100 (0.04–0.10 mm) with a petroleum ether–ethyl acetate mixture as eluent.

DFT calculations. All computations were carried out at the DFT/HF hybrid level of theory using Becke's three-parameter hybrid exchange functional in combination with the gradient-corrected correlation functional of Lee, Yang, and Parr (B3LYP) by using the GAUSSIAN 2009 program packages.[1] The geometries optimization were performed using the 6-311+G(2d,2p) basis set

[1] Frisch, M. J.; Trucks, G. W.; Schlegel, H. B.; Scuseria, G. E.; Robb, M. A.; Cheeseman, J. R.; Scalmani, G.; Barone, V.; Mennucci, B.; Petersson, G. A.; Nakatsuji, H.; Caricato, M.; Li, X.; Hratchian, H. P.; Izmaylov, A. F.; Bloino, J.; Zheng, G.;. Sonnenberg, J. L; Hada, M.; Ehara, M.; Toyota, K.; Fukuda, R.; Hasegawa, J.; Ishida, M.; Nakajima, T.; Honda, Y.; Kitao, O.; Nakai, H.; Vreven, T.; Montgomery, Jr., J. A.; Peralta, J. E.; Ogliaro, F.; Bearpark, M.; Heyd, J. J.; Brothers, E.; Kudin, K. N.; Staroverov, V. N.; Keith, T.; Kobayashi, R.; Normand, J.; Raghavachari, K.; Rendell, A.; Burant, J. C.; Iyengar, S. S.; Tomasi, J.; Cossi, M.; Rega, N.; Millam, J. M.; Klene, M.; Knox, J. E.; Cross, J. B.; Bakken, V.; Adamo, C.; Jaramillo, J.; Gomperts, R.; Stratmann, R. E.; Yazyev, O.; Austin, A. J.; Cammi, R.; Pomelli, C.; Ochterski, J. W.; Martin, R. L.; Morokuma, K.; Zakrzewski, V. G.; Voth, G. A.; Salvador, P.; Dannenberg, J. J.; Dapprich, S.; Daniels, A. D.;

(standard 6-311 basis set added with polarization (d,p) and diffuse functions). Optimizations were performed on all degrees of freedom and solvent-phase optimized structures were verified as true minima with no imaginary frequencies. The Hessian matrix was calculated analytically for the optimized structures in order to prove the location of correct minima and to estimate the thermodynamic parameters. Gibbs free energies were calculated for 25 °C. Solvent-phase calculations used the Polarizable Continuum Model (PCM).

X-ray analysis. Single crystal X-ray analysis was performed with a single crystal diffractometer Agilent Technologies (Oxford Diffraction) «Supernova». A suitable crystal was selected for study and kept at 100(2) K during data collection. Using Olex2[2] the structure was solved with the ShelXS[3] structure solution program using Direct Methods and refined with the ShelXL refinement package using Least Squares minimisation. CCDC 1526767 (**2a**) and CCDC 1526105 (**2m**) contain the supplementary crystallographic data, which can be obtained free of charge at www.ccdc.cam.ac.uk/conts/retrieving.htmL or from the Cambridge Crystallographic Data Centre, 12 Union Road, Cambridge CB2 1EZ, UK; Fax: (internat.) + 44-1223-336-033; E-mail: deposit@ccdc.cam.ac.uk.

1.2. Preparation and characterization of starting oxadiazoles **1**.

Starting oxadiazoles **1** in yields of 80–90% were obtained by the reaction of the corresponding amidoximes with cinnamoyl chlorides in pyridine,[4] or from *O*-acylamidoximes in KOH–DMSO system.[5]

(*E*)-3-Phenyl-5-(2-phenylethenyl)-1,2,4-oxadiazole (1a). Colorless solid. M.p. 95-97 °C; ^{1}H NMR (500 MHz, $(CD_3)_2CO$), δ, ppm: 7.31 d (*J* = 16.4 Hz, 1H, =CH), 7.48 – 7.51 m ($3H_{arom.}$), 7.57 – 7.59 m ($3H_{arom.}$), 7.83 – 7.84 m ($2H_{arom.}$), 7.97 d (*J* = 16.4 Hz, 1H, =CH), 8.12 – 8.14 m ($2H_{arom.}$); ^{13}C NMR (125 MHz, $(CD_3)_2CO$), δ, ppm: 110.9, 127.8, 128.8 , 129.4, 126.6, 129.7, 131.2, 131.9, 135.3, 143.4, 167.9, 174.1; HRMS: calcd for [M+H] $C_{16}H_{13}N_2O$ 249.1022; found 249.1014.

Farkas, O.; Foresman, J. B.; Ortiz, J. V.; Cioslowski, J.; Fox, D. J. *Gaussian 09, Revision C.01*, Gaussian, Inc., Wallingford CT, **2010**.

[2] Dolomanov, O. V.; Bourhis, L. J.; Gildea, R. J.; Howard, J. A. K.; Puschmann, H. *J. Appl. Cryst.* **2009**, *42*, 339-341.

[3] *SHELXS*; Sheldrick, G. M. *Acta Cryst.* **2008**, *A64*, 112-122.

[4] Chiou, S.; Shine, H.J. *J. Heterocyclic Chem.* **1989**, *26*, 125-128.

[5] Baykov, S.; Sharonova, T.; Osipyan, A.; Rozhkov, S.; Shetnev, A.; Smirnov, A. *Tetrahedron Lett.* **2016**, *57*, 2898–2900.

(*E)*-3-(3-Nitrophenyl)-5-(2-phenylethenyl) -1,2,4-oxadiazole (1b). Yellow solid. M.p.160-162 °C; ^{1}H NMR (500 MHz, $(CD_3)_2CO$), δ, ppm: 7.36 d (*J* = 16.4 Hz, 1H, =CH), 7.49 – 7.52 m ($3H_{arom.}$), 7.85 – 7.87 m ($2H_{arom.}$), 7.92 t (*J* = 8.0 Hz, $1H_{arom.}$), 8.03 d (*J* = 16.4 Hz, 1H, =CH). 8.46 – 8.48 m ($1H_{arom.}$), 8.51 – 8.53 m ($1H_{arom.}$), 8.87 – 8.88 m ($1H_{arom.}$);^{13}C NMR (125 MHz, $(CD_3)_2CO$), δ, ppm: 110.8, 122.8, 126.7, 129.2, 130.0, 131.7, 131,8, 133.8, 135.5, 144.5, 168.0, 177.2; HRMS: calcd for [M+H] $C_{16}H_{12}N_3O_3$ 294.0873; found 294.0880.

(*E)*-3-(4-Methoxyphenyl)-5-(2-phenylethenyl)-1,2,4-oxadiazole (1c). Colorless solid. M.p. 130-132 °C; ^{1}H NMR (500 MHz, $(CD_3)_2CO$), δ, ppm: 3.92 s (3H, OMe), 7.14 d (*J* = 8.8 Hz, $2H_{arom.}$), 7.30 d (*J* = 16.4 Hz, 1H, =CH), 7.50 – 7.54 m ($3H_{arom.}$), 7.84 – 7.86 m ($2H_{arom.}$), 7.96 d (*J* = 16.4 Hz, 1H, =CH), 8.08 d (*J* = 8.8 , $2H_{arom.}$); ^{13}C NMR (125 MHz, $(CD_3)_2CO$), δ, ppm: 55.7, 111.0, 115.0, 120.0, 128.8, 129.4, 129.7, 131.1, 135.4, 143.1, 162.9, 168.9, 175.9; HRMS: calcd for [M+H] $C_{17}H_{15}N_2O_2$ 324.0979; found 324.0987.

(*E)*-3-(4-Nitrophenyl)-5-(2-(4-methylphenyl)ethenyl)-1,2,4-oxadiazole (1d). Yellow solid. M.p. 185-187 °C; ^{1}H NMR (500 MHz, DMSO-d6), δ, ppm: 2.49 s (3H, Me), 7.31 – 7.37 m ($3H_{arom.}$), 7.38 d (*J* = 16.3 Hz, 1H, =CH), 7.92 d (*J* = 7.6 Hz, $1H_{arom.}$), 8.16 d (*J* = 16.3 Hz, 1H, =CH), 8.33 d (*J* = 8.8 Hz, $2H_{arom.}$), 8.43 d (*J* = 8.8 Hz, $2H_{arom.}$); ^{13}C NMR (125 MHz, DMSO-d6), δ, ppm: 19.9, 111.3, 124.9, 127.0, 129.0, 131.1, 131.4, 132.8, 138.2, 141.8, 144.1, 167.3, 176.6; HRMS: calcd for [M+H] $C_{17}H_{14}N_3O_3$ 308.1030; found 308.1037.

(*E)*-3-(4-Methoxyphenyl)-5-(2-(4-methylphenyl)ethenyl)-1,2,4-oxadiazole (1e). Colorless solid. M.p. 123-125 °C; ^{1}H NMR (500 MHz, $(CD_3)_2CO$), δ, ppm: 2.38 s (3H, Me),3.90 s (3H, OMe), 7.10 d (*J* = 8.6 Hz, $2H_{arom.}$),7.21 d (*J* = 16.4 Hz, 1H, =CH), 7.31 d(*J* = 7.7 Hz, $2H_{arom.}$),7.71 d (*J* = 7.4 Hz, $2H_{arom.}$),7.89 d (*J* = 16.4 Hz, 1H, =CH),8.04 d (*J* = 8.6 Hz, $2H_{arom.}$); ^{13}C NMR (125 MHz, $(CD_3)_2CO$), δ, ppm: 21.5, 55.9, 110.3, 115.4, 120.5, 129.2, 129.8, 130.7, 130.0, 141.9, 143.5, 163.2, 169.2, 174.4; HRMS: calcd for [M+H] $C_{18}H_{17}N_2O_2$ 293.1285; found 293.1269

(*E)*-5-(2-(4-Fluorophenyl)ethenyl)-3-phenyl-1,2,4-oxadiazole (1f). Colorless solid. M.p. 122-124 °C; ^{1}H NMR (500 MHz, $(CD_3)_2CO$), δ, ppm: 7.26 – 7.29 m (1H, =CH), 7.26 – 7.29 m ($2H_{arom.}$), 7.56 – 7.59 m ($3H_{arom.}$), 7.90 – 7.94 m ($2H_{arom.}$), 7.95 d (*J* = 16.4 Hz, 1H, =CH), 8.11 – 8.13 m ($2H_{arom.}$); ^{13}C NMR (125 MHz, $(CD_3)_2CO$), δ, ppm: 111.2 d (*J* = 2.52 Hz), 117.0 d (*J* = 22.2 Hz),128.1, 128.2, 130.0, 131.5 d (*J* = 8.6 Hz), 132.3, 142.4, 154.9, 165.0 d (*J* = 249.6 Hz), 169.6, 176.5; ^{19}F NMR (470 MHz, $(CD_3)_2CO$), δ, ppm: -109.52 - -109.46 m (1F); HRMS: calcd for [M+H] $C_{16}H_{12}FN_2O$ 267.0928; found 267.0900

(*E)*-5-(2-(4-Fluorophenyl)ethenyl)-3-(4-nitrophenyl)-1,2,4-oxadiazole (1g). Yellow solid. M.p. 221-223 °C; ^{1}H NMR (500 MHz, DMSO-d6), δ, ppm: 8.43 d (*J* = 8.5 Hz, $2H_{arom.}$), 8.31 d (*J* = 8.5 Hz, $2H_{arom.}$), 7.97 d (*J* = 16.3 Hz, 1H, =CH), 7.96 – 7.94 m ($2H_{arom.}$), 7.46 d (*J* = 16.3 Hz, 1H, =CH), 7.32 m ($2H_{arom.}$); ^{13}C NMR (125 MHz, DMSO-d6), δ, ppm: 109.7, 111.9 d (*J* = 3 Hz),, 113.5,

117.0 d (J = 22.1 Hz), 124.4, 128.4, 130.9 d (J = 10.7 Hz), 142.3, 143.1, 162.8, 165.1 d (J = 250 Hz), 169.8; ^{19}F NMR (470 MHz, DMSO-d6), δ, ppm: -108.02 - -107.96 m (1F); HRMS: calcd for [M+H] $C_{16}H_{12}FN_2O$ 267.0928; found 267.09222

(*E)*-5-(2-(4-Fluorophenyl)ethenyl)-3-(4-methoxyphenyl)-1,2,4-oxadiazole (1h). Yellow solid. M.p. 144-145 °C; ^{1}H NMR (500 MHz, $(CD_3)_2CO$), δ, ppm: 3.89 s (3H, OMe), 7.1 d (J = 8.95 Hz, 2$H_{arom.}$), 7.24-7.29 m (2$H_{arom.}$), 7.25 d (J = 16.4, 1H, =CH), 7.90-7.93m (2H_{arom}), 7.93 d (J = 16.4 Hz, 1H, =CH), 8.05 dd (J = 7.0, 2.07 Hz, 2$H_{arom.}$); ^{13}C NMR (125 MHz, $(CD_3)_2CO$), δ, ppm: 55.9, 111.3 d (J = 2.9 Hz), 115.4, 117.0 d (J = 22.2 Hz), 120.4, 129.8, 131.4 d (J = 9.1 Hz), 132.3, 142.2, 151.0, 163.2, 165.0 d (J = 249.6 Hz), 169.3; ^{19}F NMR (470 MHz, $(CD_3)_2CO$), δ, ppm: -109.84 - -109.78 m (1F); HRMS: calcd for [M+Na] $C_{17}H_{13}FN_2O_2$ 319.0859; found 319.0839

(*E)*-5-(2-(4-Chlorophenyl)ethenyl)-3-phenyl-1,2,4-oxadiazole (1i). Colorless solid. M.p. 154-156 °C; ^{1}H NMR (500 MHz, $(CD_3)_2CO$), δ, ppm: 7.36 d (J = 16.4 Hz, 1H, =CH), 7.53 – 7.60 m (5$H_{arom.}$), 7.88 d (J = 8.5 Hz, 2$H_{arom.}$), 7.96 d (J = 16.4 Hz, 1H, =CH), 8.11 – 8.13 m (2$H_{arom.}$); ^{13}C NMR (125 MHz, $(CD_3)_2CO$), δ, ppm: 112.1, 128.0, 128.1, 130.0, 130.2, 130.8, 132.3, 134.5, 136.82, 142.2, 169.6, 176.4; HRMS: calcd for [M+H] $C_{16}H_{12}ClN_2O$ 283.0633; found 283.0639

(*E)*-5-(2-(4-Chlorophenyl)ethenyl)-3-(4-nitrophenyl)-1,2,4-oxadiazole (1j). Yellow solid. M.p. 195-197 °C; ^{1}H NMR (500 MHz, DMSO-d_6), δ, ppm: 8.43 d (J = 8.7 Hz, 2$H_{arom.}$), 8.31 d (J = 8.7 Hz, 2$H_{arom.}$), 8.0 d (J = 16.4 Hz, 1H, =CH), 7.91 d (J = 8.4 Hz, 2$H_{arom.}$), 7.55 d (J = 8.4 Hz, 2$H_{arom.}$), 7.54 d (J = 16.4 Hz, 1H, =CH); ^{13}C NMR (125 MHz, DMSO-d_6), δ, ppm: 110.5, 124.5, 129.1,130.2, 132.0, 133.1, 135.3, 142.1, 149.2, 151.1, 166.8, 175.9; HRMS: calcd for [M+Na] $C_{16}H_{10}ClN_2NaO_3$: 350.0308, found 350.0293.

(*E)*-5-(2-(4-Chlorophenyl)ethenyl)-3-(4-methoxyphenyl)-1,2,4-oxadiazole (1k). Colorless solid. M.p. 148-151 °C; ^{1}H NMR (500 MHz, DMSO-d_6), δ, ppm: 3.84 s (3H, OMe), 7.12 d (J = 8.4 Hz, 2$H_{arom.}$), 7.45 d (J = 16.4 Hz, 1H, =CH), 7.53 d (J = 8.4 Hz, 2$H_{arom.}$), 7.87 d (J = 8.4 Hz, 2$H_{arom.}$), 7.91 d (J = 16.4 Hz, 1H, =CH), 7.98 d (J = 8.7 Hz, 2$H_{arom.}$),; ^{13}C NMR (125 MHz, DMSO-d_6), δ, ppm: 55.4, 111.1, 114.6, 118.5, 128.7, 128.9, 130.0, 133.2, 135.1, 141.3, 161.7, 167.7, 174.9; HRMS: calcd for [M+Na] $C_{17}H_{13}ClN_2NaO_2$: 335.0563, found 335.0546.

(*E)*-5-(2-(4-Methoxyphenyl)ethenyl)-3-(4-nitrophenyl)-1,2,4-oxadiazole (1l). Yellow solid. M.p. 230-232 °C; ^{1}H NMR (500 MHz, DMSO-d6), δ, ppm: 3.83 s (3H, OMe), 7.04 t (J = 8.7 Hz, 2$H_{arom.}$), 7.33 d (J = 16.5 Hz, 1H, =CH), 7.84 d (J = 8.8 Hz, 2$H_{arom.}$), 7.95 d (J = 16.5 Hz, 1H, =CH), 8.32 – 8.30 m (2$H_{arom.}$), 8.43 d (J = 8.8 Hz, 2$H_{arom.}$); ^{13}C NMR (125 MHz, DMSO-d6), δ, ppm: 55.4, 107.2, 109.2, 114.5, 117.7, 124.4, 128.4, 130.4, 143.3, 168.3, 170.1, 171.3, 173.7; HRMS: calcd for [M+H] $C_{17}H_{14}N_3O_4$ 324.0979; found 324.0987.

(*E)*-3-(4-Methoxyphenyl)-5-(2-(4-methoxyphenyl)ethenyl)-1,2,4-oxadiazole (1m). Yellow solid. M.p. 110-112 °C; ^{1}H NMR (500 MHz, $(CD_3)_2CO$), δ, ppm: 3.87 s (3H, OMe), 3.89 s (3H,

OMe),7.09 – 7.12 m ($2H_{arom.}$), 7.09 – 7.12 m (1H, =CH), 7.77 d (*J* = 8.8 Hz, $2H_{arom.}$), 7.87 d (*J* = 16.4 Hz, 1H, =CH), 8.04 d (*J* = 8.8 Hz, $2H_{arom.}$); ^{13}C NMR (125 MHz, $(CD_3)_2CO$), δ, ppm: 55.7, 55.8, 108.5, 115.2, 115.3,120.4, 128.2, 129.6, 130.7, 143.1, 162.7, 163.0, 168.9, 176.4; HRMS: calcd for [M+H] $C_{18}H_{17}N_2O_3$ 309.1234; found 309.1238.

(*E)*-3-(4-Methoxyphenyl)-5-(2-(3,4-dimethoxyphenyl)ethenyl)-1,2,4-oxadiazole (1n). Yellow solid. M.p. 108-110 °C; ^1H NMR (500 MHz, $(CD_3)_2CO$), δ, ppm: 3.89 s (3H, OMe) 3.88 s (3H, OMe) 3.92 s (3H, OMe), 7.04 d (*J* = 8.3 Hz, $1H_{arom.}$),7.10 d (*J* = 8.9 Hz, $2H_{arom}$), 7.15 d (*J* = 16.4, 1H, =CH), 7.15 d (*J* = 16.4, 1H, =CH), 7.33 dd (*J* = 8.3, 2.0, $1H_{arom.}$), 7.48 s ($1H_{arom.}$), 7.84 d (*J* = 16.4 Hz, 1H, =CH), 8.04 d (*J* = 8.9 Hz, $2H_{arom.}$); ^{13}C NMR (125 MHz, $(CD_3)_2CO$), δ, ppm: 55.7, 56.0, 56.1, 108.6, 110.9, 112.4, 115.2, 120.3, 123.8, 128.4, 129.6, 143.5, 150.7, 152.7, 163.0, 169.0, 176.5; HRMS: calcd for [M+H] $C_{19}H_{19}N_2O_4$ 339.1339; found 339.1346.

1.3. Preparation and characterization of oxadiazoles **2**.

General procedure for the hydroarylation reaction. In a similar manner as described in the literature,[6] oxadiazole **1** (1.2 mmol) was added to a mixture of the arene (1.2 equiv) and TfOH (1 mL). The reaction mixture was stirred at rt or at 60 °C for the given time (1–52 h, see Table 2). Then, the mixture was poured into water (50 mL) and extracted with chloroform (3 × 50 mL). The extracts were combined, washed with water (50 mL), saturated aq. $NaHCO_3$ (50 mL), and water again (2 × 50 mL), and dried over Na_2SO_4. After removal of the solvent by distillation under reduced pressure the residue was subjected to chromatographic separation on silica gel using hexane–ethyl acetate as an eluent.

Reactions using FSO_3H (1 mL with 0.5 mL of CH_2Cl_2, at −80 or −60 °C), TfOH-SbF_5 (20 mol %) or AlX_3 (X = Cl, Br) (5 equiv in 5 mL of benzene), and under microwave irradiations were carried out and worked up in the same way.

3-Phenyl-5-(2,2-diphenylethyl)-1,2,4-oxadiazole (2a). Colorless solid, yield of 80 %. M.p. 120-122 °C; ^1H NMR (500 MHz, $CDCl_3$), δ, ppm: 3.84 d (*J* = 8.1 Hz, 2H, CH_2),4.8 t (*J* = 8.1 Hz, 1H, CH),7.18 t (*J* = 7.5 Hz, $2H_{arom.}$),7.30 t (*J* = 7.7 Hz, $3H_{arom.}$),7.44 d (*J* = 7.5 Hz, $4H_{arom.}$),7.49 – 7.54 m ($4H_{arom.}$),8.00 dd (*J* = 7.9, *J* = 1.7 Hz, $2H_{arom.}$); ^{13}C NMR (125 MHz, $CDCl_3$): 33.2, 49.6, 127.6, 128.0, 128.6, 129.5, 129.9, 132.1, 144.2, 168.9, 179.7; HMRS: calcd for [M+H] $C_{22}H_{19}N_2O$, 327.1492; found 327.1501.

5-(2-(4-Chlorophenyl)-2-phenylethyl)-3-phenyl-1,2,4-oxadiazole (2b) and **5-(2-(2-chlorophenyl)-2-phenylethyl)-3-phenyl-1,2,4-oxadiazole (2c)** were obtained as a mixture of

[6] Gurskaya, L. Yu.; Belyanskaya, D. S.; Ryabukhin, D. S.; Nilov, D. I.; Boyarskaya, I. A.; Vasilyev, A. V. *Beilstein J. Org. Chem.* **2016**, *12,* 950–956.

isomers. Colorless solid, m.p.130-132 °C (for the ratio of **2b : 2c** ~ 7 : 1). **2b:** yield of 84 %; ^{1}H NMR (500 MHz, $(CD_3)_2CO$), δ, ppm. (selected signals from the spectrum of mixture of isomers): 3.85 d (*J* = 8.2 Hz, 2H, CH_2), 4.25 t (*J* = 8.2 Hz, 1H, CH), 7.20 t (*J* = 7.4 Hz, $1H_{arom.}$), 7.29 – 7.34 m ($4H_{arom.}$), 7.43 – 7.47 m ($4H_{arom.}$), 7.50 – 7.54 m ($3H_{arom.}$), 7.98 – 8.00 m ($2H_{arom.}$); ^{13}C NMR (125 MHz, $(CD_3)_2CO$), δ, ppm (selected signals from the spectrum of mixture of isomers): 33.1, 48.9, 127.8, 128.0, 128.6, 128.9, 129.5, 129.7, 129.9, 130.4, 130.8, 132.2, 143.2, 143.7, 168.9, 179.5. **2c:** yield of 12 %; ^{1}H NMR (500 MHz, $(CD_3)_2CO$), δ, ppm. (selected signals from the spectrum of mixture of isomers): 3.79 – 3.87 m (2H, CH_2), 5.31 t (*J* = 8.1 Hz, 1H, CH); ^{13}C NMR (125 MHz, $CD_3)_2CO$), δ, ppm (selected signals from the spectrum of mixture of isomers): 32.8, 45.5, 127.9, 128.4, 129.3, 130.7, 132.9. HRMS: calcd for [M+H] $C_{22}H_{18}ClN_2O$, 361.1102; found 361.1098 (for mixture of isomers **2b** and **2c**).

5-(2-(3,4-Dichlorophenyl)-2-phenylethyl)-3-phenyl-1,2,4-oxadiazole (2d) and **5-(2-(2,3-dichlorophenyl)-2-phenylethyl)-3-phenyl-1,2,4-oxadiazole (2e)** were obtained as mixture of isomers. Colorless solid, m.p.112-114 °C (for the ratio of **2d : 2e** ~ 12 : 1). **2d:** yield of 60 %; ^{1}H NMR (500 MHz, $CD_3)_2CO$), δ, ppm (selected signals from the spectrum of mixture of isomers): 3.90 dd (*J*=8.2, 4.2 Hz, 2H, CH_2), 4.86 t (*J*=8.2 Hz, 1H, CH), 7.22 t (*J* = 7.4 Hz, $1H_{arom.}$), 7.30 – 7.34 m ($2H_{arom.}$), 7.45 – 7.59 m ($7H_{arom.}$), 7.68 – 7.69 m ($1H_{arom.}$), 7.99 (*J* = 8.2 Hz, $2H_{arom.}$);^{13}C NMR (125 MHz, (CD3)2CO) δ, ppm (selected signals from the spectrum of mixture of isomers): 32.7, 48.6, 113.3. **2e:** yield of 5%; ^{1}H NMR (500 MHz, $CD_3)_2CO$), δ, ppm (selected signals from the spectrum of mixture of isomers): 3.93d (*J* = 8.2 Hz, 2H, CH_2), 4.86 t (*J* = 8.2 Hz, 1H, CH), 7.83 – 7.84 m ($1H_{arom.}$), 8.13 dd (*J* = 7.5, 2.1 Hz, $1H_{arom.}$); ^{13}C NMR (125 MHz, $CD_3)_2CO$), δ, ppm (selected signals from the spectrum of mixture of isomers): 32.9, 46.4. Spectral data for mixture of isomers **2d** and **2e:** ^{13}C NMR (125 MHz, $CD_3)_2CO$,] δ, ppm: 127.9, 128.1, 128.6, 128.9, 129.2, 129.8, 130.0, 130.1, 130.9, 131.5, 131.6, 132.2, 132.3, 132.9, 135.6, 143.2, 143.7, 145.4; HRMS: calcd for [M+H] $C_{23}H_{18}Cl_3N_2O_2$, 459.0428; found 459.0423 (for mixture of isomers **2d** and **2e**).

3-(3-Nitrophenyl)-5-(2,2-diphenylethyl)-1,2,4-oxadiazole (2f). Oily compound, yield of 89 %; ^{1}H NMR (500 MHz, $CDCl_3$), δ, ppm: 3.75 d (*J* = 8.1 Hz, 2H, CH_2), 4.81 t (*J* = 8.1 Hz, H, CH), 7.23-7.26 m ($2H_{arom.}$), 7.33-7.34 m ($8H_{arom.}$), 7.65 t (*J* = 8.0 Hz, $H_{arom.}$), 8.33-8.37 m ($2H_{arom.}$), 8.88 br.s ($H_{arom.}$); ^{13}C NMR (125 MHz, $CDCl_3$), δ, ppm: 33.0, 48.5, 122.5, 125.5, 127.0, 127.5, 128.6, 128.7, 129.9, 132.9, 142.3, 148.5,166.6, 179.0; HRMS: calcd for [M+Na] $C_{22}H_{17}N_3NaO_3$, 394.1168; found 394.1163.

3-(3-Nitrophenyl)-5-(2-phenyl-2-(4-hydroxyphenyl)ethyl)-1,2,4-oxadiazole (2g). Oily compound, yield of 39 %; ^{1}H NMR (500 MHz, $CDCl_3$) δ, ppm: 3.68 d (*J* = 8.2 Hz, 2H, $-CH_2$), 4.71 t (*J* = 8.2 Hz, H, -CH), 4.98 br.s (1H, OH), 6.75 d (*J* = 8.4 Hz, $2H_{arom.}$), 7.14 d (*J* = 8.4 Hz, $2H_{arom.}$), 7.21 t (*J* = 6.8 Hz, $1H_{arom.}$), 7.28-7.32 m ($4H_{arom.}$), 7.64 t (*J* = 8.1 Hz, $1H_{arom.}$), 8.33 d (*J* = 8.1 Hz,

$2H_{arom.}$), 8.87 s ($1H_{arom}$); ^{13}C NMR (125 MHz, $CDCl_3$) δ, ppm: 33.3, 47.8, 115.6, 122.5, 125.5, 126.9, 127.4, 128.6, 128.7, 128.8, 129.9, 132.9, 134.5, 142.6, 148.5, 154.5, 166.6, 179.0; HRMS: calcd for [M+H] $C_{22}H_{18}N_3O_4$, 388.1292; found 388.1326. Calcd for [M+Na] $C_{22}H_{17}N_3NaO_4$, 410.1117; found 410.1127

3-(4-Methoxyphenyl)-5-(2,2-diphenylethyl)-1,2,4-oxadiazole (2h). Colorless solid, yield of 80 %. M.p. 203-205 °C; ^{1}H NMR (500 MHz, $CDCl_3$) δ, ppm: 3.66 d (*J* = 8.1 Hz, 2H, CH_2), 3.84 s (3H, OMe), 4.75 t (*J* = 8.1 Hz, 1H, CH), 7.17 – 7.20 m ($2H_{arom.}$), 7.27 s ($4H_{arom.}$), 7.28 s ($4H_{arom.}$), 7.94 d (*J* = 8.8 Hz, $2H_{arom.}$);^{13}C NMR (125 MHz, CDCl3) δ, ppm: 33.1, 48.7, 55.2, 114.3, 119.3, 127.0, 127.8, 128.9, 129.2, 142.5, 161.9, 168.0, 177.8; HRMS: calcd for [M+H] $C_{23}H_{21}N_2O_2$, 357.1598; found 357.1592

5-(2-(4-Methylphenyl)-2-phenylethyl)-3-(4-nitrophenyl)-1,2,4-oxadiazole (2i) and 5-(2-(3-methylphenyl)-2-phenylethyl)-3-(4-nitrophenyl)-1,2,4-oxadiazole (2j) were obtained as mixture of isomers. Oily compound (for the ratio of **2i : 2j** ~ 2 : 1). **2i**: yield of 60 %; **^{1}**H NMR (500 MHz, $(CD_3)_2CO$), δ, ppm. (from the spectrum of mixture of isomers): 2.40 s (3H, Me), 3.87 – 3.94 m (2H, CH_2), 5.07 t (*J* = 8.2 Hz, 1H, CH), 7.13 – 7.19 m ($2H_{arom.}$), 7.21 – 7.25 m ($3H_{arom.}$), 7.31 – 7.36 m ($4H_{arom.}$), 7.39 d (*J* = 7.5 Hz, $1H_{arom.}$), 7.45 – 7.49 m ($1H_{arom.}$), 8.29 dd (*J* = 8.9 Hz, 2.2 Hz, $2H_{arom.}$); **^{13}**C NMR (125 MHz, $(CD_3)_2CO$) δ, ppm. (from the spectrum of mixture of isomers): 21.0 , 33.6, 45.2, 125.1, 127.2, 127.6, 128.4, 128.9, 129.5, 131.6, 133.6, 137.0, 141.6, 143.5, 150.5, 167.6, 180.7. **2j:** yield of 31%; **^{1}**H NMR (500 MHz, $(CD_3)_2CO$), δ, ppm. (selected signals from the spectrum of mixture of isomers): 2.27 s (3H, Me), 3.87 – 3.94 m (2H, CH_2), 4.80 t (*J* = 8.2 Hz, 1H, CH), 7.56 d (*J* = 7.8 Hz, $2H_{arom.}$),8.42 d (*J* = 8.0 Hz, $2H_{arom.}$); **^{13}**C NMR (125 MHz, $(CD_3)_2CO$), δ, ppm. (selected signals from the spectrum of mixture of isomers): 20.0 , 33.3, 49.2, 127.4, 128.2, 130.1, 140.9, 144.3.HRMS: calcd for [M+H] $C_{23}H_{21}N_2O_2$, 386.1499; found 386.1512 (for mixture of isomers **2i** and **2j**).

3-(4-Methoxyphenyl)-5-(2-phenyl-2-(4-methylphenyl)ethyl)-1,2,4-oxadiazole (2k). Oily compound, yield of 90 %; ^{1}H NMR (500 MHz, $(CD_3)_2CO$) δ, ppm: 2.24 s (3H, Me), 3.77 – 3.80 m (2H, -CH_2), 3.86 s (3H, OMe), 4.74 t (*J* = 8.2 Hz, 1H, -CH), 7.05 d (*J* = 8.9 Hz, $2H_{arom.}$), 7.09 d (*J* = 7.9 Hz, $2H_{arom.}$), 7.16-7.20 m ($1H_{arom.}$), 7.27-7.31 m ($4H_{arom.}$), 7.41 – 7.44 m ($2H_{arom.}$), 7.93 d (*J* = 8.9 Hz, $2H_{arom.}$);^{13}C NMR (125 MHz, $(CD_3)_2CO$) δ, ppm: 20.9, 33.3, 49.2, 55.8, 115.2, 120.2, 127.5, 128.5, 128.6, 129.4, 129.6, 130.1, 137.1, 141.2, 144.5. 163.0, 168.6, 179.4; HRMS: calcd for [M+H] $C_{24}H_{23}N_2O_2$, 371.1754; found 371.1759.

5-(2-(4-Fluorophenyl)-2-phenylethyl)-3-phenyl-1,2,4-oxadiazole (2l): Colorless solid, yield of 93 %. M.p. 95-97 °C; **^{1}**H NMR (500 MHz, $(CD_3)_2CO$), δ, ppm: 3.84 d (*J* = 8.2 Hz, 2H, CH_2), 4.82 t (*J* = 8.2 Hz, 1H, CH), 7.06 t (*J* = 8.8 Hz, $2H_{arom.}$), 7.20 t (*J* = 7.3 Hz, $1H_{arom.}$), 7.31 t (*J* = 7.7 Hz, $2H_{arom.}$), 7.44 d (*J* = 7.7 Hz, $2H_{arom.}$), 7.47 – 7.49 m ($2H_{arom.}$), 7.52 – 7.55 m ($2H_{arom.}$), 7.61 –

7.68 m ($1H_{arom.}$), 8.00 dd (*J* = 7.8, 1.58 Hz, $2H_{arom.}$); **^{13}C** NMR (125 MHz, $(CD_3)_2CO$), δ, ppm: 33.3, 48.8, 116.1 d (*J* = 21.3 Hz), 127.7, 128.0, 128.5, 129.6, 129.9, 130.4 d (*J* = 7.8 Hz), 132.1, 134.3, 140.3 d (*J* = 3.2 Hz), 144.0, 162.5 d (*J* = 243.3 Hz), 168.9, 179.5; ^{19}F NMR (470 MHz, $(CD_3)_2CO$), δ, ppm: -117.50– -117.44 (m, 1F); HRMS: calcd for [M+H] $C_{22}H_{18}FN_2O$, 345.1398; found 345.1406.

5-(2-(4-Fluorophenyl)-2-phenylethyl)-3-(4-nitrophenyl)-1,2,4-oxadiazole (2m): Colorless solid, yield of 91 %. M.p. 135-137 ºC; ^{1}H NMR (500 MHz, $(CD_3)_2CO$) δ, ppm: 3.90 d (*J* = 8.2 Hz, 2H, CH_2), 4.84 t (*J* = 8.2 Hz, 1H, CH), 7.06 t (*J* = 8.8, $2H_{arom.}$), 7.06 t (*J* = 8.8, $2H_{arom.}$), 7.20 t (*J* = 7.5 Hz, $1H_{arom.}$), 7.31 t (*J* = 7.5 Hz, $2H_{arom.}$), 7.47-7.50 (m, $2H_{arom.}$), 8.25 d (*J* = 8.9, $2H_{arom.}$), 8.25 d (*J* = 8.9, $2H_{arom.}$), 8.38 (d, *J* = 8.8 Hz, $2H_{arom.}$); ^{13}C NMR (125 MHz, $(CD_3)_2CO$), δ, ppm: 33.2, 48.7, 116.1 (d, *J* = 21.3 Hz), 125.0, 127.7, 128.4, 129.2, 129.5, 130.4 (d, *J* = 8.0), 133.5, 140.1 (d, *J* = 3.3 Hz), 143.8, 150.5, 162.5 d (*J* = 243.4 Hz),167.5, 180.4;^{19}F NMR (470 MHz, $(CD_3)_2CO$): -116.18– -116.12 m (1F); HRMS: calcd for [M+H] $C_{22}H_{17}FN_3O_3$, 390.1248; found 390.1245.

5-(2-(4-Fluorophenyl)-2-phenylethyl)-3-(4-methoxyphenyl)-1,2,4-oxadiazole (2n): Oily compound, yield of 90 %; ^{1}H NMR (500 MHz, $(CD_3)_2CO$), δ, ppm: 3.81 d (*J* = 8.2 Hz, 2H, CH_2), 3.86 s (3H, OMe), 4.81 t (*J* = 8.2 Hz, 1H, CH), 7.07-7.04 m ($4H_{arom.}$), 7.19 t (*J* = 7.5 Hz, $1H_{arom.}$), 7.30 t (*J* = 7.7 Hz, $2H_{arom.}$), 7.43 d (*J* = 7.5 Hz, $2H_{arom.}$), 7.47-7.49 m ($2H_{arom.}$), 7.92 d (*J* = 8.9 Hz, $2H_{arom.}$); ^{13}C NMR (125 MHz, $(CD_3)_2CO$), δ, ppm: 33.3, 48.8, 55.9, 115.3, 116.1 d (*J* = 21.3 Hz), 120.2, 127.7, 128.5, 129.6 d (*J* = 8.0 Hz), 130.5 d (*J* = 8.0 Hz), 140.4 d (J = 3.0 Hz), 144.1, 162.5 d (*J* = 241.8 Hz), 163.1, 168.6, 179.2; ^{19}F NMR (470 MHz, $(CD_3)_2CO$) δ, ppm: -116.39– -116.33 m (1F); HRMS: calcd for [M+H] $C_{23}H_{19}FN_2NaO_2$, 397.1328; found 397.1321.

5-(2-(4-Fluorophenyl)-2-(4-hydroxyphenyl)ethyl)-3-(4-methoxyphenyl)-1,2,4-oxadiazole (2o) and **5-(2-(4-Fluorophenyl)-2-(2-hydroxyphenyl)ethyl)-3-(4-methoxyphenyl)-1,2,4-oxadiazole (2p)** were obtained as mixture of isomers. Oily compound (for the ratio of **2o : 2p** ~ 2 : 1). **2o**: yield of 51 %; **^{1}H** NMR (500 MHz, $(CD_3)_2CO$), δ, ppm. (selected signals from the spectrum of mixture of isomers): 3.60 dd (*J* = 8.1 Hz, 2H, -CH_2), 3.85 s (3H, OMe) 4.67 t (*J* = 8.1, 1H, -CH), 6.74 d (*J* = 8. 5, $2H_{arom.}$), 6.89 d (*J* = 8.8, $2H_{arom.}$), 6.95 – 6.98 m ($2H_{arom.}$), 7.10 d (*J* = 8.5, $2H_{arom.}$), 7.20 – 7.22 m ($2H_{arom.}$), 7.94 d (*J* = 8.8 , $2H_{arom.}$); **^{13}C** NMR (125 MHz, $(CD_3)_2CO$) δ, ppm. (selected signals from the spectrum of mixture of isomers): 33.6, 47.2, 55.5, 114.4 d (*J* = 7.9 Hz), 115.7 d (*J* = 21.2 Hz), 115.8, 115.9, 128.6, 128.90, 129.2 d (*J* = 8.2 Hz), 129.4, 134.8, 155.6 d (*J* = 235.7 Hz),158.3, 162.0, 168.0, 179.3; ^{19}F NMR [470 MHz, $(CD_3)_2CO$] δ, ppm. (from the spectrum of mixture of isomers): -117.05 – -116.90 m (1F). **2p:** yield of 25 %; **^{1}H** NMR (500 MHz, $(CD_3)_2CO$) δ, ppm. (selected signals from the spectrum of mixture of isomers): 3.72 – 3.75 m (2H, CH_2), 3.76 s (3H, OMe) 4.68 t (*J* = 8.1 Hz, 1H, CH), 6.82 d (*J* = 8.8 Hz, $2H_{arom.}$), 6.95 – 6.98 m ($3H_{arom.}$), 7.16 d (*J* = 8.7 Hz, $2H_{arom.}$), 7.89 d (*J* = 8.7 Hz, $2H_{arom.}$);**^{13}C** NMR (125 MHz, $(CD_3)_2CO$),

δ, ppm. (selected signals from the spectrum of mixture of isomers): 55.4, 177.4 ; ^{19}F NMR (470 MHz, $(CD_3)_2CO$), δ, ppm. (selected signals from the spectrum of mixture of isomers): -110.27– -110.17 (m, 1F). HRMS: calcd for [M+H] $C_{23}H_{20}FN_2O_3$, 391.1452; found 391.1441 (for mixture of isomers **2o** and **2p**).

5-(2-(4-Chlorophenyl)-2-(4-fluorophenyl)ethyl)-3-(4-methoxyphenyl)-1,2,4-oxadiazole (2q) and **5-(2-(2-chlorophenyl)-2-(4-fluorophenyl)ethyl)-3-(4-methoxyphenyl)-1,2,4-oxadiazole (2r)** were obtained as mixture of isomers. Oily compound (for the ratio of **2q : 2r** ~ 6 : 1). **2q**: yield of 50 %; ^{1}H NMR (500 MHz, $(CD_3)_2CO$), δ, ppm. (selected signals from the spectrum of mixture of isomers): 3.60 d (*J*= 8.2 Hz, 2H, CH_2), 3.85 s (3H, OMe) 4.71 t (*J* = 8.2, 1H, CH), 6.94 – 6.99 m ($4H_{arom.}$), 7.17 – 7.21 m ($3H_{arom.}$), 7.23 – 7.26 m ($3H_{arom.}$), 7.93 d (*J* = 8.8 Hz, $2H_{arom.}$); ^{13}C NMR (125 MHz, $(CD_3)_2CO$), δ, ppm. (selected signals from the spectrum of mixture of isomers): 33.2, 48.2, 55.9, 115.38, 116.3 d (*J* = 21.6 Hz), 129.6, 129.7, 130.5, 130.8 d (*J* = 8.0 Hz), 145.6, 148.3, 154.1, 155.7, 162.3 d (*J* = 218.2 Hz), 163.7, 172.7, 179.0; ^{19}F NMR (470 MHz, $(CD_3)_2CO$), δ, ppm. (from the spectrum of mixture of isomers): -114.95– -114.89 m (1F). **2r:** yield of 8 %; ^{1}H NMR (500 MHz, $(CD_3)_2CO$)], δ, ppm. (selected signals from the spectrum of mixture of isomers): 5.23 t (*J* = 8.0 Hz, 1H, CH); ^{13}C NMR (125 MHz, (CD3)2CO)] δ, ppm. (selected signals from the spectrum of mixture of isomers): 33.0, 44.9, 51.8; ^{19}F NMR (470 MHz, $(CD_3)_2CO$)] δ, ppm. (from the spectrum of mixture of isomers): -108.97– -108.70 (m, 1F). HRMS: calcd for [M+H] $C_{23}H_{19}ClFN_2O_2$, 409.1114; found 409.1102 (for mixture of isomers **2q** and **2r**).

5-(2-(4-Chlorophenyl)-2-phenylethyl)-3-phenyl-1,2,4-oxadiazole (2s). Colorless solid, yield of 94 %. M.p.101-103 °C; ^{1}H NMR (500 MHz, $(CD_3)_2CO$) δ, ppm: 3.84 – 3.86 m (2H, CH_2), 4.82 t (*J* = 8.2 Hz, 1H, CH), 7.20 – 7.22 m ($1H_{arom.}$), 7.30 – 7.34 m ($4H_{arom.}$), 7.44 d (*J* = 7.8 Hz, 2Harom.), 7.47 d (*J* = 8.5 Hz, $2H_{arom.}$), 7.52 – 7.55 (m, $2H_{arom.}$); ^{13}C NMR (125 MHz, $(CD_3)_2CO$), δ, ppm: 33.1, 48.9, 127.6, 127.8, 128.0, 128.6, 129.5, 129.6, 129.9, 130.4, 132.1, 133.0, 143.1, 143.7, 168.9, 179.5; HRMS: calcd for [M+H] $C_{22}H_{18}ClN_2O$, 361.1102; found 361.1098.

5-(2-(4-Chlorophenyl)-2-phenylethyl)-3-(4-nitrophenyl)-1,2,4-oxadiazole (2t). Colorless solid, yield of 97 %. M.p. 130-132ºC; ^{1}H NMR (500 MHz, $(CD_3)_2CO$), δ, ppm: 3.92 d (*J* = 8.2 Hz, 2H, CH_2), 4.85 t (*J* = 8.2 Hz, 1H, CH), 7.21 t (*J* = 7.4 Hz, $1H_{arom}$), 7.34-7.30 m ($4H_{arom.}$), 7.49-7.45 m ($4H_{arom.}$), 8.26 d (*J* = 8.9 Hz, $2H_{arom.}$), 8.40 d (*J* = 8.9 Hz, $2H_{arom.}$); ^{13}C NMR (125 MHz, $(CD_3)_2CO$), δ, ppm: 33.1, 48.9, 125.2, 127.9, 128.6, 129.3, 129.6, 129.7, 130.5, 131.1, 133.6, 143.1, 143.6, 150.6, 167.7, 180.5; HRMS: calcd for [M+H] $C_{22}H_{18}ClN_2O$, 406.0953; found 406.0961.

5-(2-(4-Chlorophenyl)-2-(4-methylphenyl)ethyl)-3-(4-nitrophenyl)-1,2,4-oxadiazole (2u) and **5-(2-(4-Chlorophenyl)-2-(2-methylphenyl)ethyl)-3-(4-nitrophenyl)-1,2,4-oxadiazole (2v)** were obtained as mixture of isomers. Oily compound (for the ratio of **2u : 2v** ~ 12 : 1). **2u**: yield of 60 %; ^{1}H NMR (500 MHz, $(CD_3)_2CO$), δ, ppm. (from the spectrum of mixture of isomers): 2.25 s

(3H, Me), 3.89 d (J = 8.2 Hz, 2H, CH_2), 4.80 t (J = 8.2 Hz, 1H, CH), 7.09 0- 7.13m ($2H_{arom.}$), 7.31 – 7.33 m ($4H_{arom.}$), 7.46 d (J = 8.5 Hz, $2H_{arom.}$), 8.26 d (J = 8.9 Hz, $2H_{arom.}$), 8.40 d (J = 8.9 Hz, $2H_{arom.}$); **^{13}C** NMR (125 MHz, $(CD_3)_2CO$) δ, ppm. (from the spectrum of mixture of isomers): 21.1, 32.2, 48.6, 125.2, 128.5, 129.3, 129. 6, 130.3, 130.4, 133.7, 137.7, 140.6, 143.4, 150.7, 154.1, 167.7, 180.6. **2v:** yield 5%;**^{1}**H NMR (500 MHz, $(CD_3)_2CO$) δ, ppm. (selected signals from the spectrum of mixture of isomers): 2.24 s (3H, Me), 3.89 d (J = 8.2 Hz, 2H, CH_2), 4.73 t (J = 8.2 Hz, 1H, CH). HRMS: calcd for [M+H] $C_{23}H_{19}ClN_3O_3$, 420.1109; found 420.1117 (for mixture of isomers **2u** and **2v)**.

5-(2-(4-Chlorophenyl)-2-phenylethyl)-3-(4-hydroxyphenyl)-1,2,4-oxadiazole (2w). Oily compound, yield of 95 %; **^{1}**H NMR (500 MHz, $(CD_3)_2CO$), δ, ppm: 3.79 d (J = 8.1 Hz, 2H, CH_2), 4.79 t (J = 8.1 Hz, 1H, CH), 6.95 d (J = 8.7 Hz, $1H_{arom.}$), 7.19 t (J = 7.4 Hz, $1H_{arom.}$), 7.30 – 7.33 m ($4H_{arom.}$), 7.41 – 7.46 m ($4H_{arom.}$), 7.89 d (J = 8.7 Hz, $1H_{arom.}$), 7.99 d (J = 7.2 Hz, $1H_{arom.}$); **^{13}**C NMR (125 MHz, $(CD_3)_2CO$), δ, ppm: 32.83, 116.43, 118.92, 127.60, 128.37, 129.29, 129.41, 129.60, 130.22, 132.74, 143.00, 143.57, 160.84, 168.56, 178.72; HRMS: calcd for [M+H] $C_{22}H_{18}ClN_2O_2$, 377.1051; found 377.1056.

5-(2-(4-Chlorophenyl)-2-(4-methylphenyl)ethyl)-3-(4-hydroxyphenyl)-1,2,4-oxadiazole (2x). Oily compound, yield of 67 %; **^{1}**H NMR (500 MHz, $(CD_3)_2CO$) δ, ppm: 2.24 s (3H, Me), 3.76 – 3.77 m (2H, CH_2), 4.76 t (J = 8.2 Hz, 1H, CH), 6.95 d (J = 8.7 Hz, $2H_{arom.}$), 7.11 d (J = 8.0 Hz, $2H_{arom.}$), 7.31 t (J = 8.4 Hz, $4H_{arom.}$), 7.44 d (J = 8.4 Hz, $2H_{arom.}$), 7.83 – 7.86 m ($2H_{arom.}$); **^{13}**C NMR (125 MHz, $(CD_3)_2CO$) δ, ppm: 21.0, 33.2, 48.6, 116.8, 128.5, 129.5, 129.9, 130.3, 130.4, 132.9, 137.3, 140.8, 143.5, 154.1, 161.2, 168.8, 179.1; HRMS: calcd for [M+H] $C_{23}H_{19}ClN_3O_3$, 420.1109; found 420.1117.

5-(2-(4-Methoxyphenyl)-2-phenylethyl)-3-(4-nitrophenyl)-1,2,4-oxadiazole (2y). Oily compound; yield of 92 %; ^{1}H NMR (500 MHz, $(CD_3)_2CO$), δ, ppm: 3.71 s (3H, OMe), 3.85 d (J = 8.2 Hz, 2H, CH_2), 4.76 t (J = 8.2 Hz, 1H, CH), 6.84 d (J = 8.6 Hz, $2H_{arom.}$), 7.18 d (J = 7.3 Hz, $1H_{arom.}$), 7.29 d (J = 7.6 Hz, $2H_{arom.}$), 7.34 d (J = 8.6, $2H_{arom.}$), 7.41 d (J = 7.6, $2H_{arom.}$), 8.24 d (J = 8.9 Hz, $2H_{arom.}$), 8.34 d (J = 8.8 Hz, $2H_{arom.}$); ^{13}C NMR (500 MHz, $(CD_3)_2CO$), δ, ppm: 33.3, 48.7, 114.7, 116.2, 125.0, 127.5, 128.4, 129.1, 129.4, 129.5, 133.5, 135.7, 144.3, 150.4, 159.4, 167.4, 180.6; HRMS: calcd for [M+Na] $C_{23}H_{19}N_3NaO_4$, 424.1273; found 424.1264.

5-(2-(4-Methoxyphenyl)-2-phenylethyl)-3-(4-methoxyphenyl)-1,2,4-oxadiazole (2z). Oily compound, yield of 50 %; ^{1}H NMR (500 MHz, $(CD_3)_2CO$), δ, ppm: 3.72 s (3H, OMe), 3.76 dd (J = 8.2, 1.0 Hz, 2H, CH_2), 3.86 s (3H, OMe), 4.27 t (J = 8.2 Hz, 1H, CH), 7.05 d (J = 8.8, $2H_{arom.}$), 7.17 t (J = 7.3 Hz, $1H_{arom.}$), 7.30-7.27 m ($2H_{arom.}$), 7.33 d (J = 8.8 Hz, $2H_{arom.}$), 7.40 d (J = 7.3, $2H_{arom.}$), 7.93 d (J = 8.8 Hz, $2H_{arom.}$);^{13}C NMR (125 MHz, $(CD_3)_2CO$) δ, ppm: 33.3, 48.7, 55.4, 55.7, 114.7,

115.1, 120.1, 128.4, 128.5, 129.3, 129.4, 129.5, 135.9, 144.5, 159.4, 163.0, 168.7, 179.2; HRMS: calcd for [M+H] $C_{18}H_{17}N_2O_3$, 309.1234; found 309.1228.

3-(4-Methoxyphenyl)-5-(2-(3,4-dimethoxyphenyl)-2-phenylethyl)-1,2,4-oxadiazole (2za). Oily compound, yield of 53 %; ^{1}H NMR (500 MHz, $(CD_3)_2CO$), δ, ppm: 3.73 s (3H, OMe), 3.76 s (3H, OMe), 3.78 – 3.75 m (2H, CH_2), 3.85s (3H, OMe), 4.71 t (*J* = 8.2 Hz, 1H, CH), 6.91 dd (*J* = 8.3, 1.5 Hz, $1H_{arom.}$), 7.02 – 7.05 m ($3H_{arom.}$), 7.28 t (*J* = 7.5 Hz, $2H_{arom.}$), 7.41 d (*J* = 7.5 Hz, $2H_{arom.}$), 7.93 d (*J* = 8.9 Hz, $2H_{arom.}$);^{13}C NMR (125 MHz, $(CD_3)_2CO$), δ, ppm: 33.0, 48.7, 55.4, 55.7, 55.8, 112.4, 114.8, 119.0, 119.7, 120.1, 127.0, 128.0, 128.9, 129.2, 136.4, 144.0, 148.8, 149.9, 162.6, 168.1, 179.0; HRMS: calcd for [M+Na] $C_{25}H_{24}N_2NaO_4$, 439.1634; found 439.1629.

1.4. NMR spectra of cations **Ca** and **Cm**

Cation Ca : ^{1}H NMR (400 MHz, FSO_3H, -80 ºC), δ, ppm: 7.53 br.d (*J* = 15.6 Hz, 1H, =CH^{α}), 7.60-8.20 m ($10H_{arom.}$), 9.10 br.d (*J* = 15.6 Hz, 1H, =$CH^{ß}$), 12.97 (N^4H^+); ^{13}C NMR (100 MHz, FSO_3H, -40 ºC), δ, ppm: 98.7, 113.4, 130.8, 131.0, 132.0, 132.8, 134.5, 140.6, 141.3, 159.4 (=$C^{ß}$), 169.0, 172.4; ^{15}N NMR (FSO_3H, -80 ºC), δ, ppm (from ^{1}H–^{15}N HSQC data): ~ 150 (N^4H^+).

Cation Cm: ^{1}H NMR (400 MHz, FSO_3H, -60 ºC), δ, ppm: 4.24 s (3H, MeO), 4.29 s (3H, MeO), 7.33 br.d (*J* = 13.6 Hz, 1H, =CH^{α}), 7.39-7.53 m ($4H_{arom.}$), 8.14 br.s ($4H_{arom.}$), 8.93 br.d (*J* = 13.6 Hz, 1H, =$CH^{ß}$), 12.55 (N^4H^+); ^{13}C NMR (100 MHz, FSO_3H, -60 ºC), δ, ppm: 58.4 (MeO), 58.9 (MeO), 96.3, 105.6, 117.0, 117.9, 128.7, 134.6, 156.9 (=$C^{ß}$), 164.5, 166.0, 167.7, 168.1, 170.4; ^{15}N NMR (FSO_3H, -80 ºC), δ, ppm (from ^{1}H–^{15}N HSQC data): ~ 140 (N^4H^+).

2. NMR spectra (^{1}H, ^{13}C, ^{19}F, NOESY, HSQC) for compounds 1, 2 and cations C

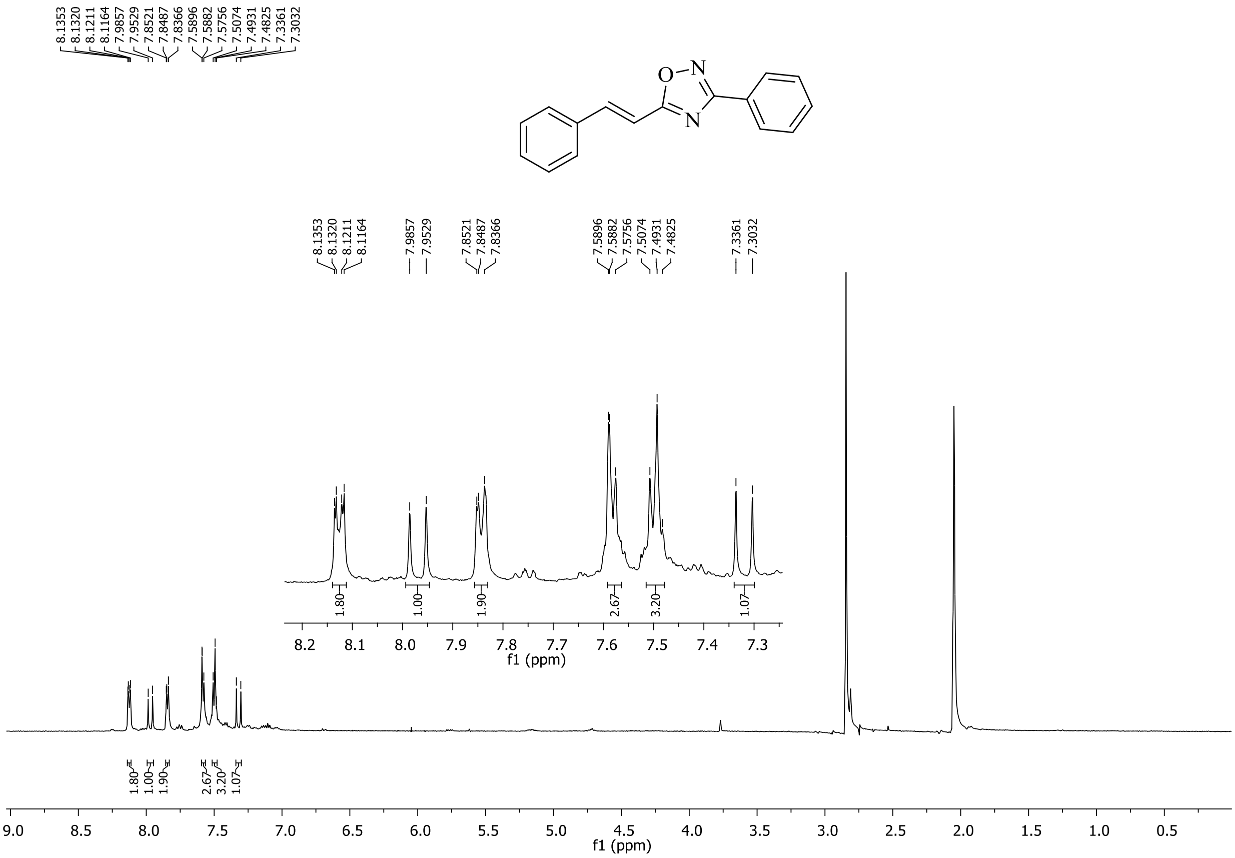

Fig. S1. ^{1}H NMR spectrum of compound **1a** [500 MHz, $(CD_3)_2CO$].

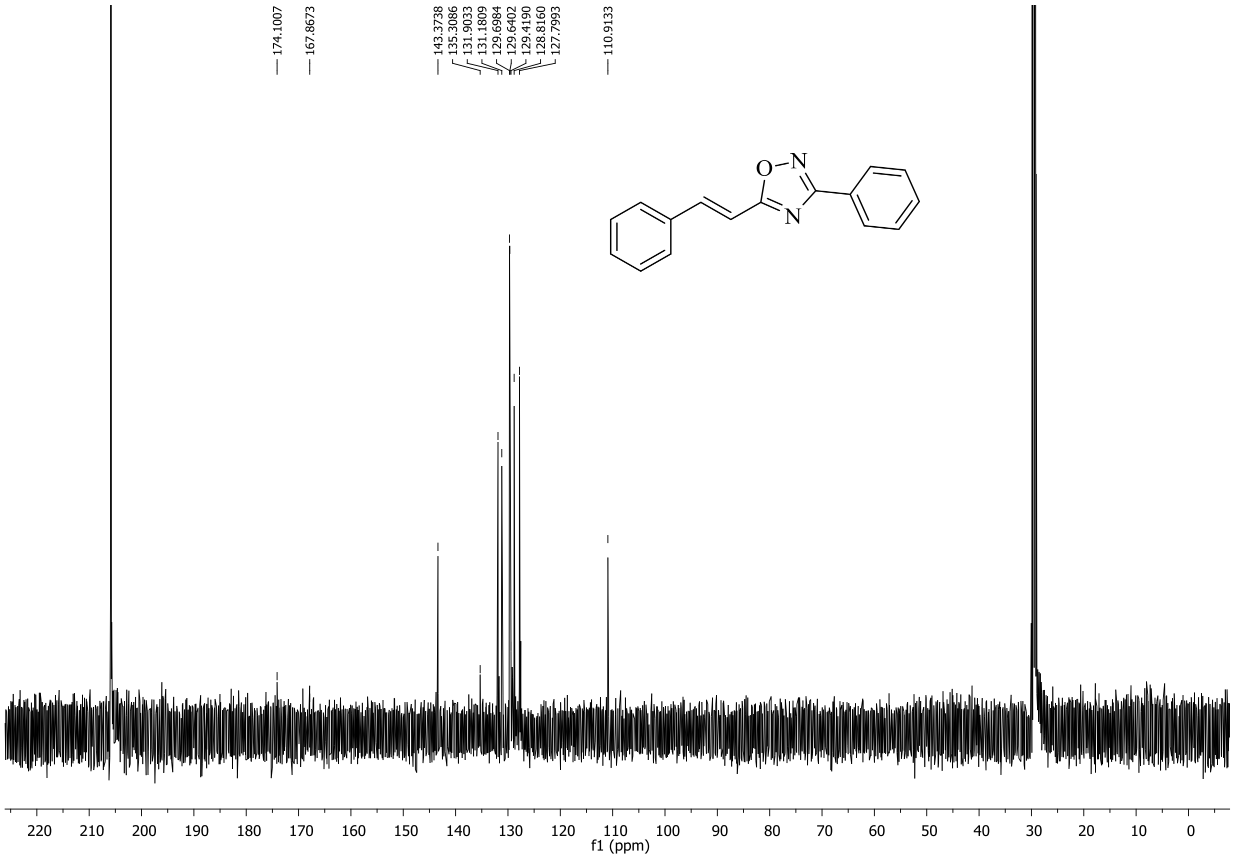

Fig. S2. ^{13}C NMR spectrum of compound **1a** [125 MHz, $(CD_3)_2CO$].

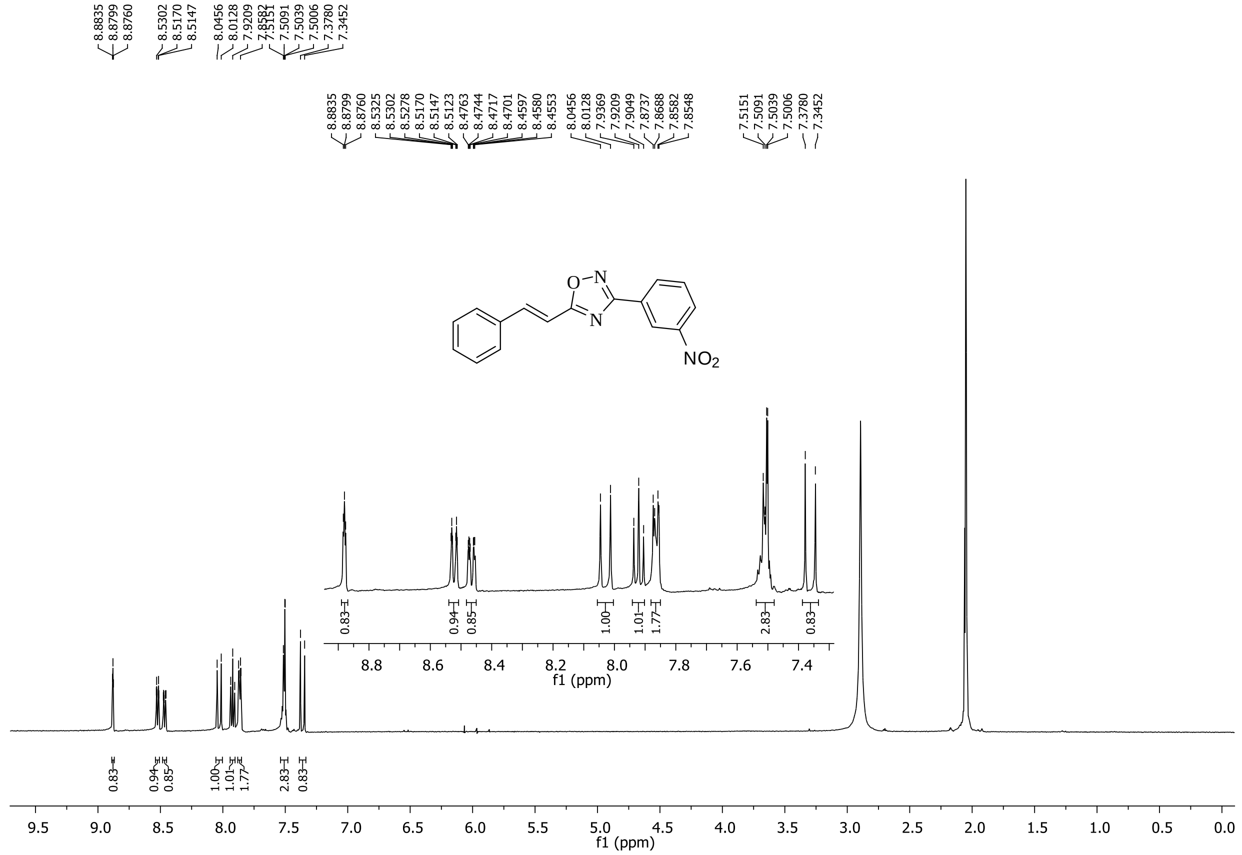

Fig. S3. ^{1}H NMR spectrum of compound **1b** [500 MHz, $(CD_3)_2CO$].

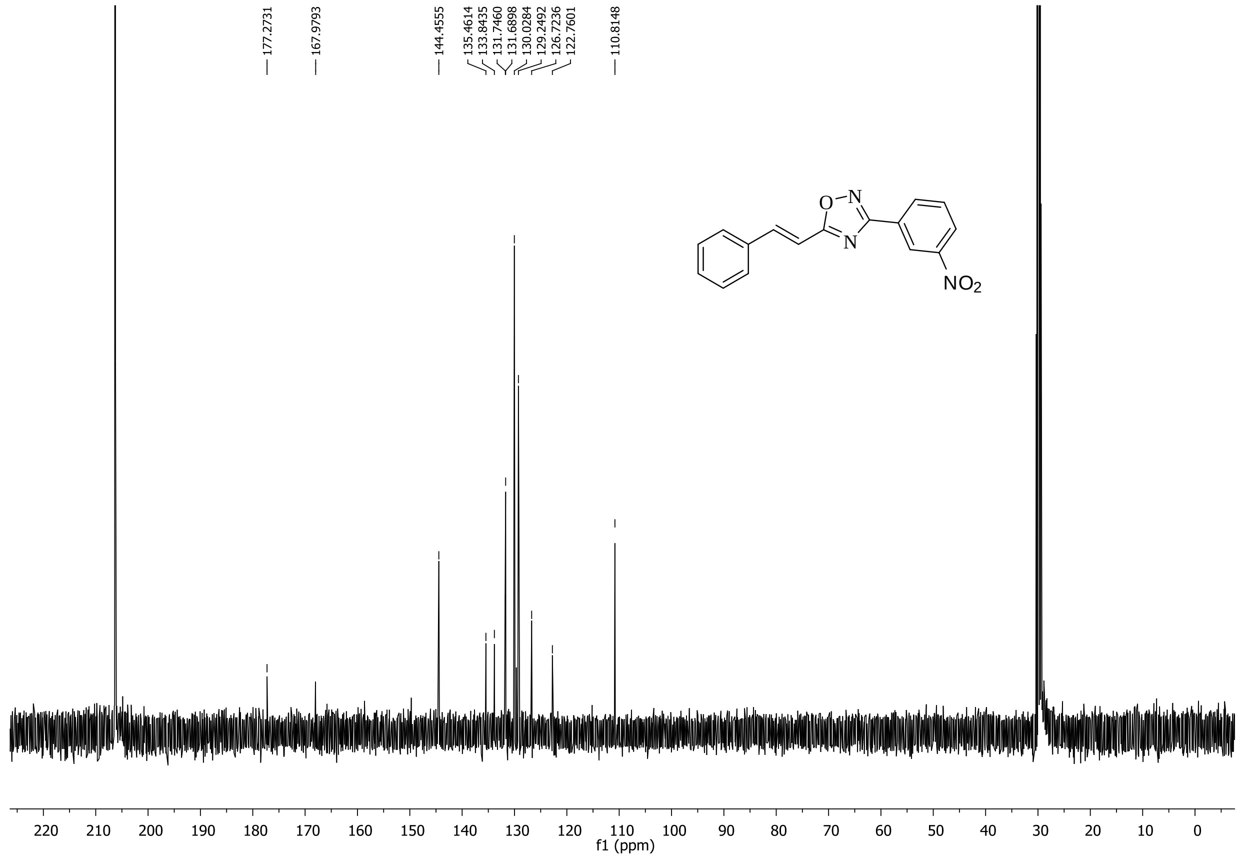

Fig. S4. ^{13}C NMR spectrum of compound **1b** [125 MHz, $(CD_3)_2CO$].

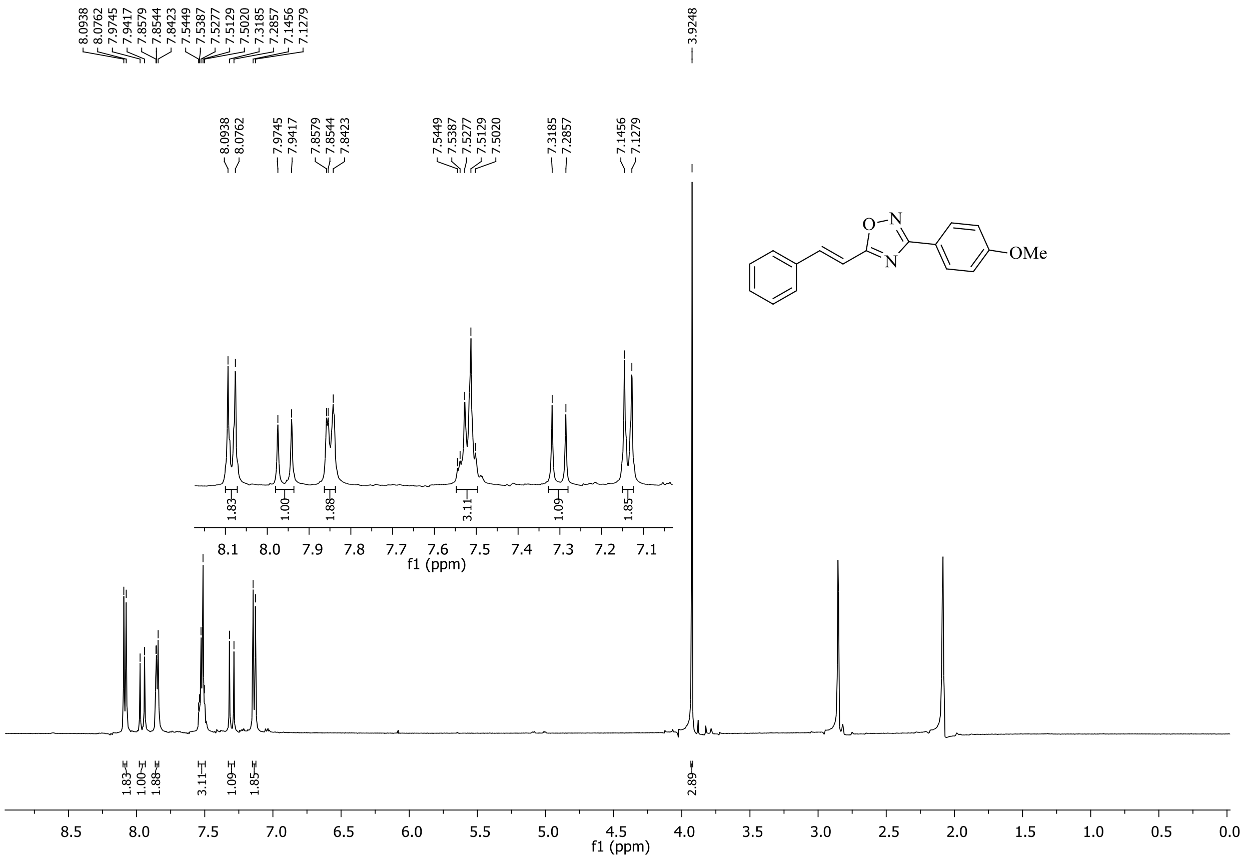

Fig. S5. ^{1}H NMR spectrum of compound **1c** [500 MHz, $(CD_3)_2CO$].

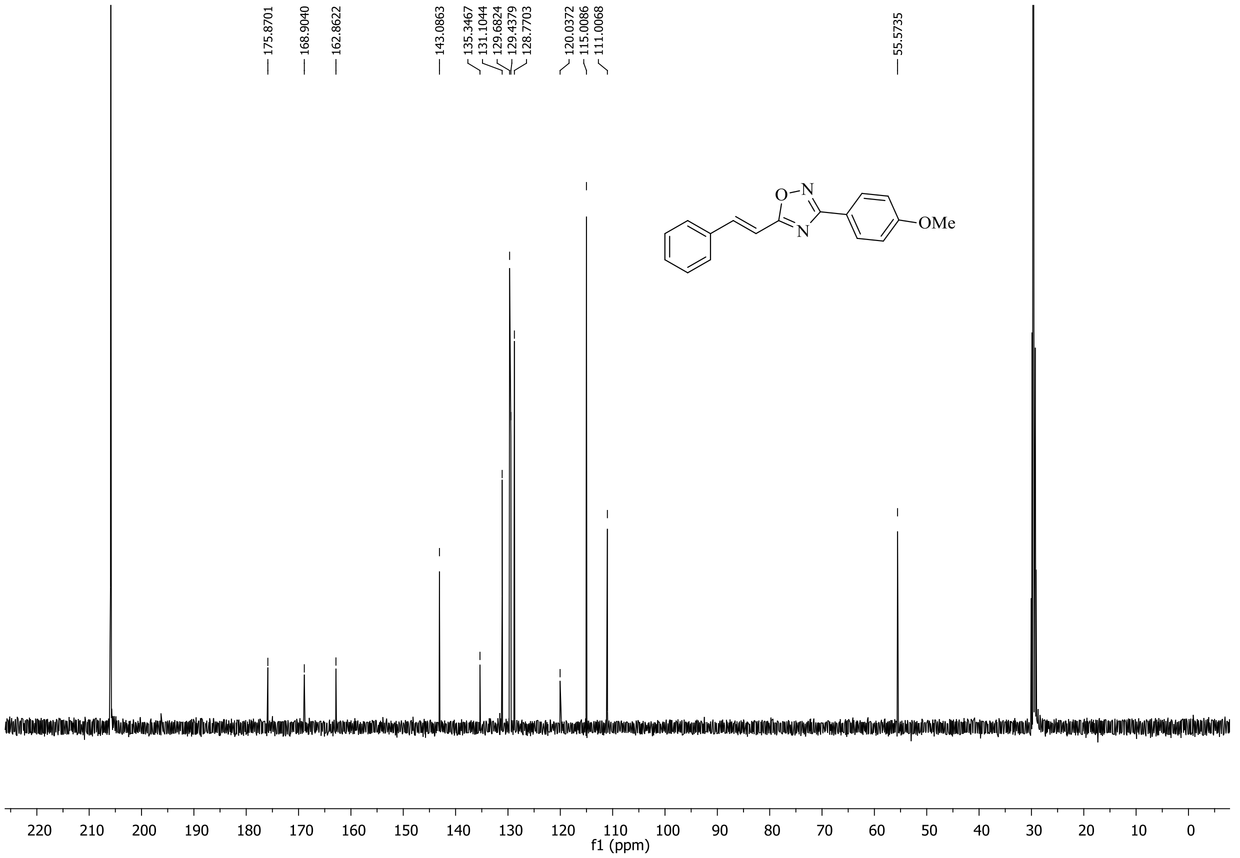

Fig. S6. ^{13}C NMR spectrum of compound **1c** [125 MHz, $(CD_3)_2CO$].

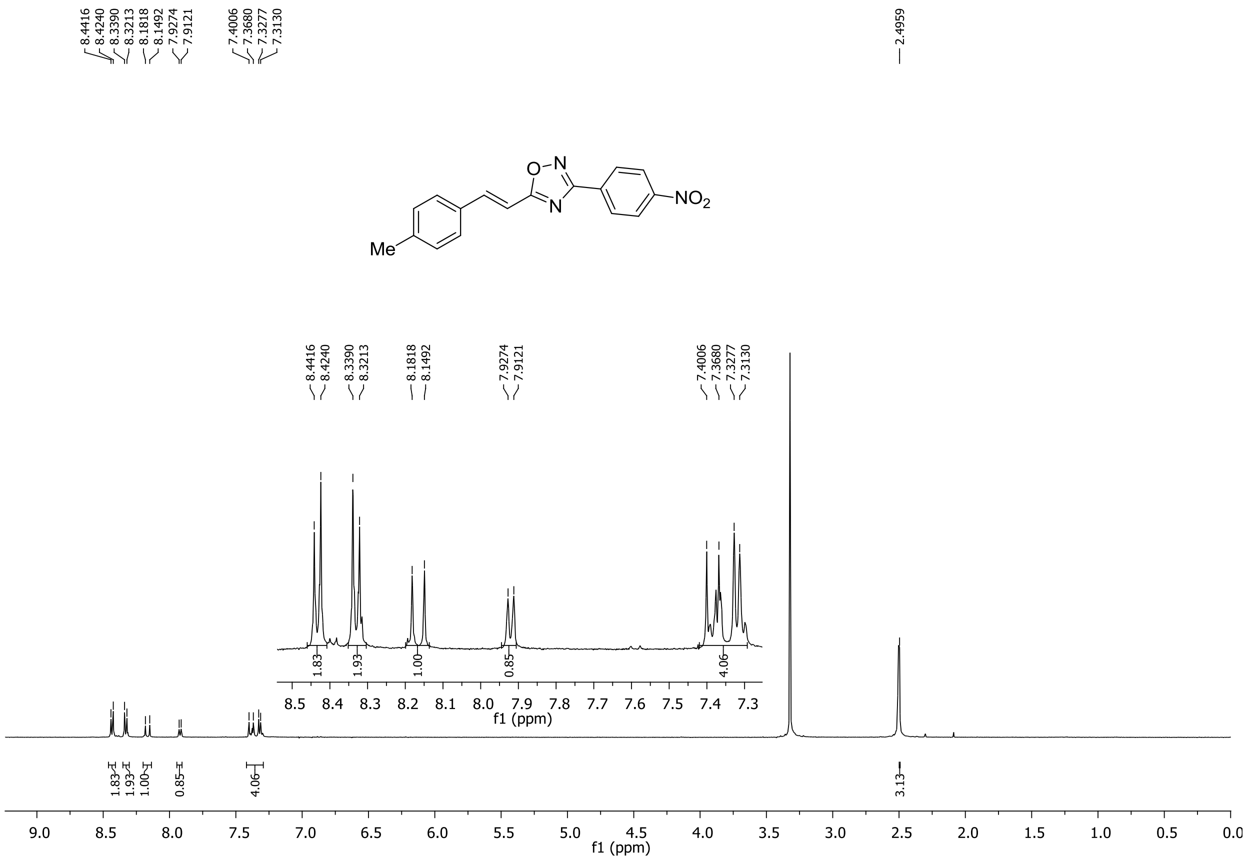

Fig. S7. ^{1}H NMR spectrum of compound **1d** [500 MHz, DMSO-d6].

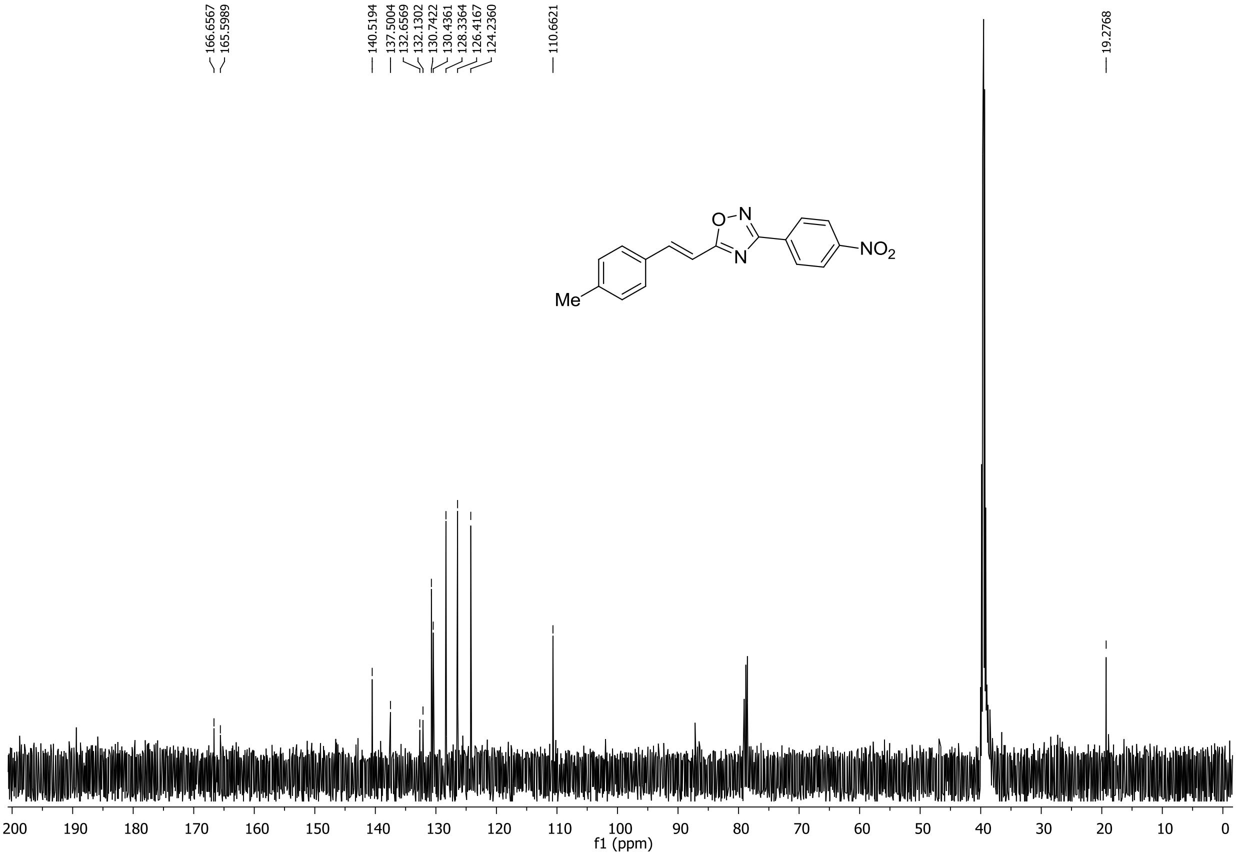

Fig. S8. ^{13}C NMR spectrum of compound **1d** 125 MHz, DMSO-d6].

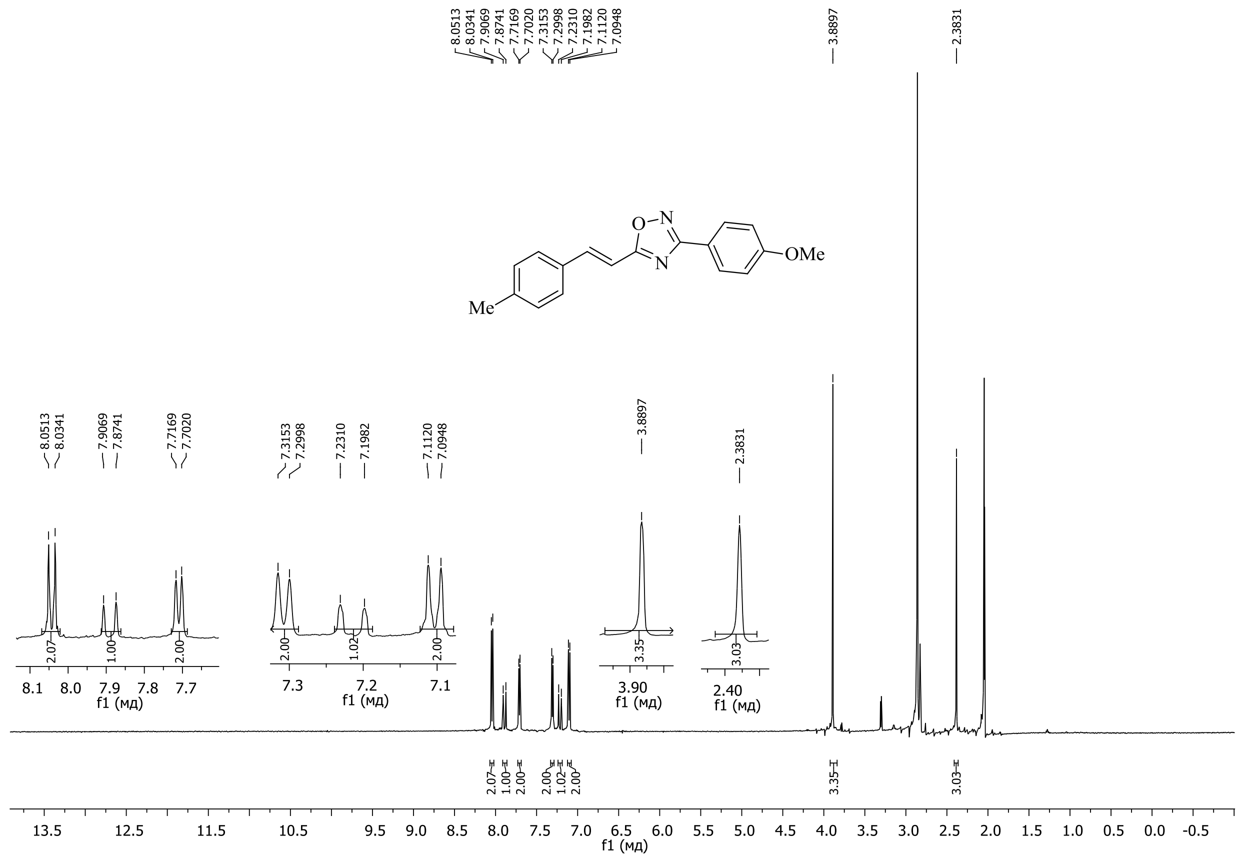

Fig. S9. ^{1}H NMR spectrum of compound **1e** [500 MHz, $(CD_3)_2CO$].

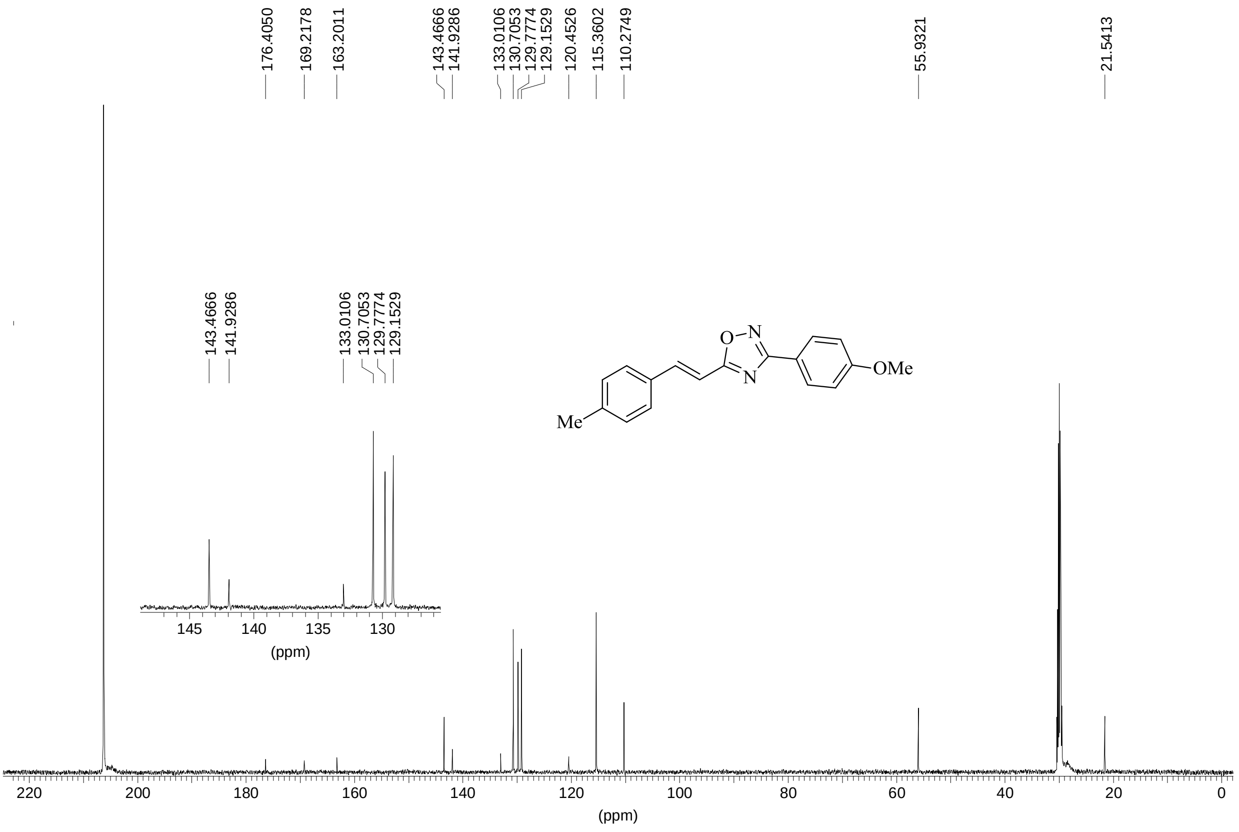

Fig. S10. ^{13}C NMR spectrum of compound **1e** [125 MHz, $(CD_3)_2CO$].

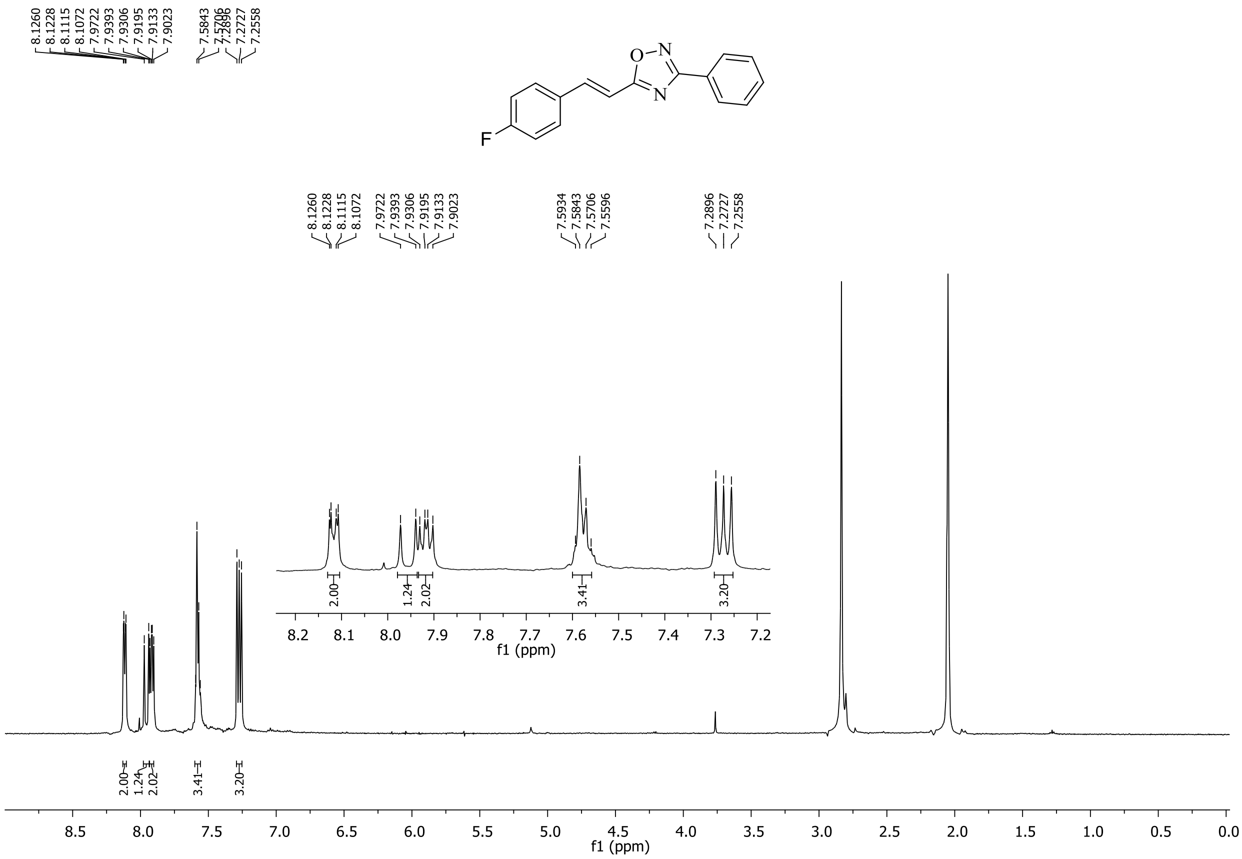

Fig. S11. ^{1}H NMR spectrum of compound **1f** [500 MHz, $(CD_3)_2CO$].

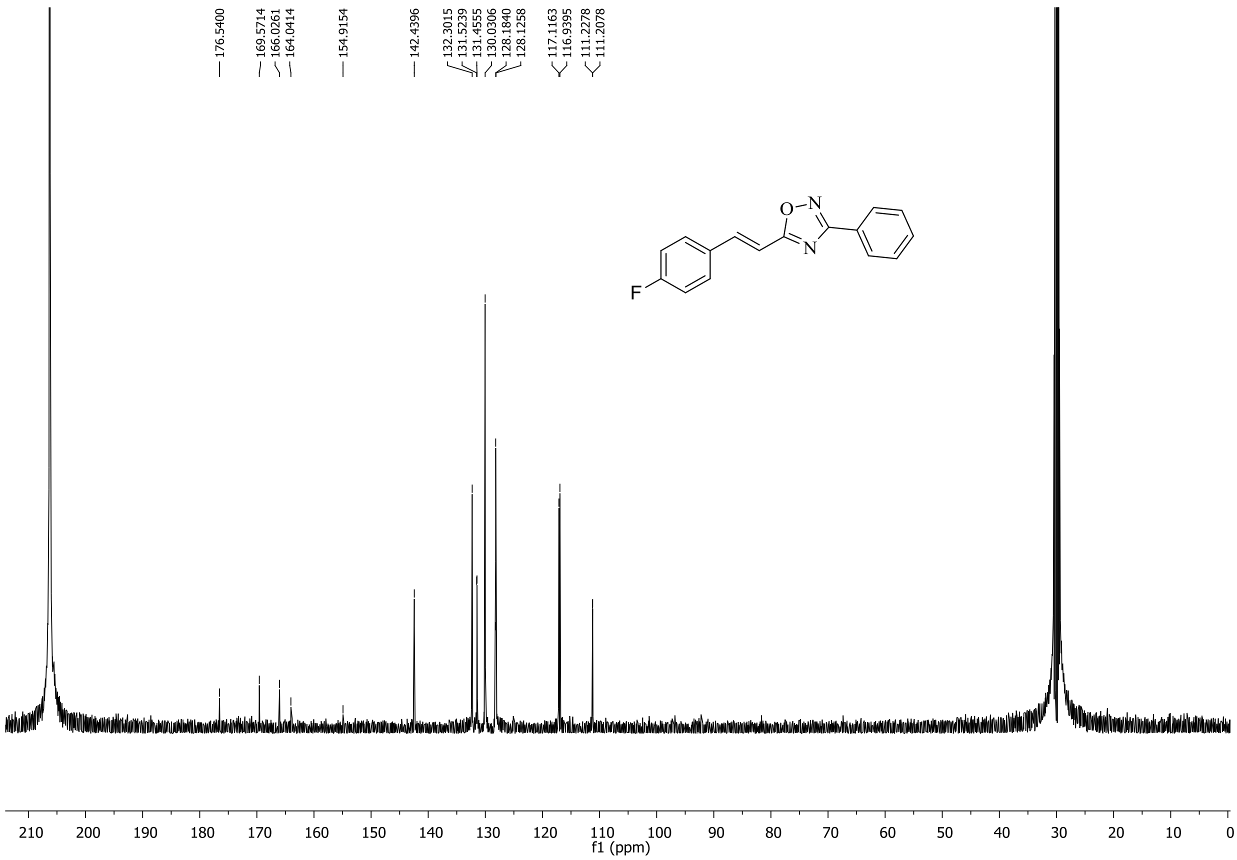

Fig. S12. ^{13}C NMR spectrum of compound **1f** [125 MHz, $(CD_3)_2CO$].

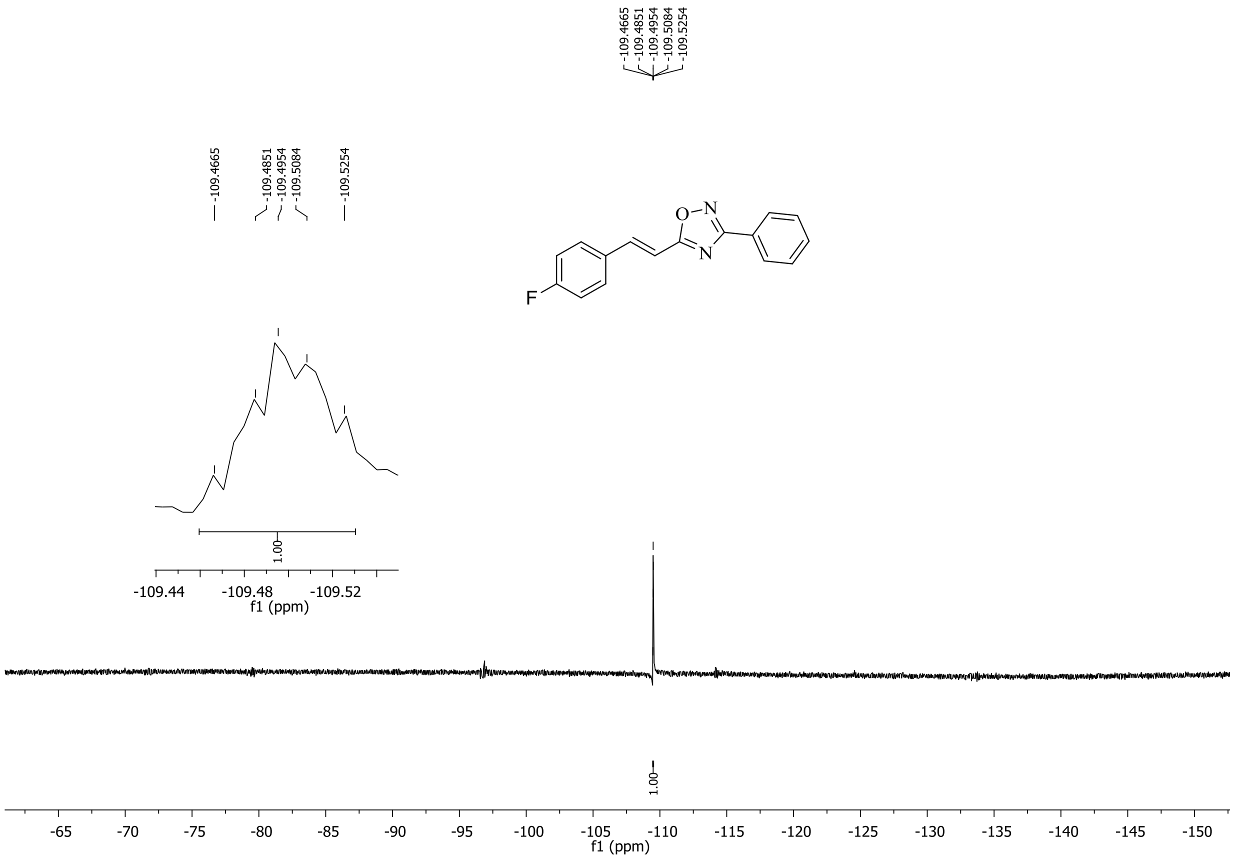

Fig. S13. ^{19}F NMR spectrum of compound **1f** [470 MHz, $(CD_3)_2CO$].

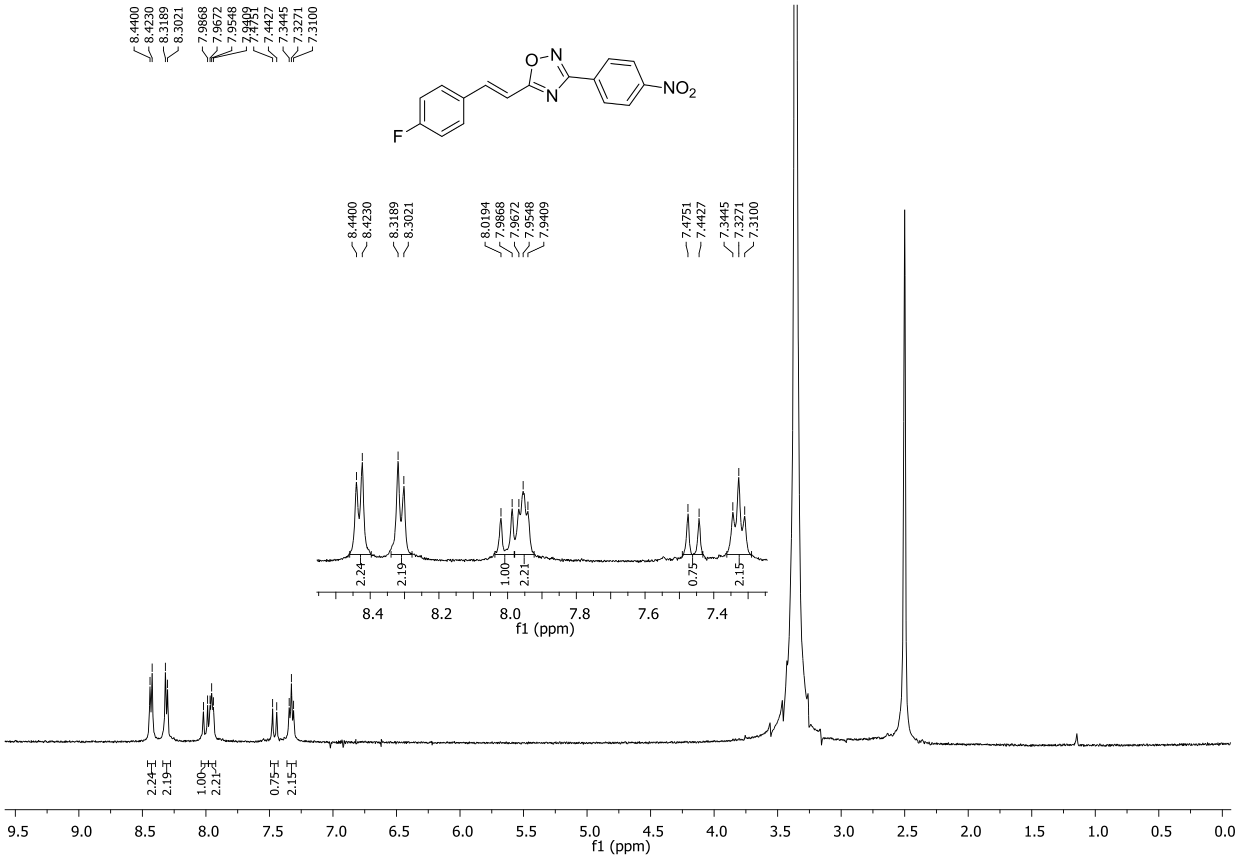

Fig. S14. ^{1}H NMR spectrum of compound **1g** [500 MHz, DMSO-d6].

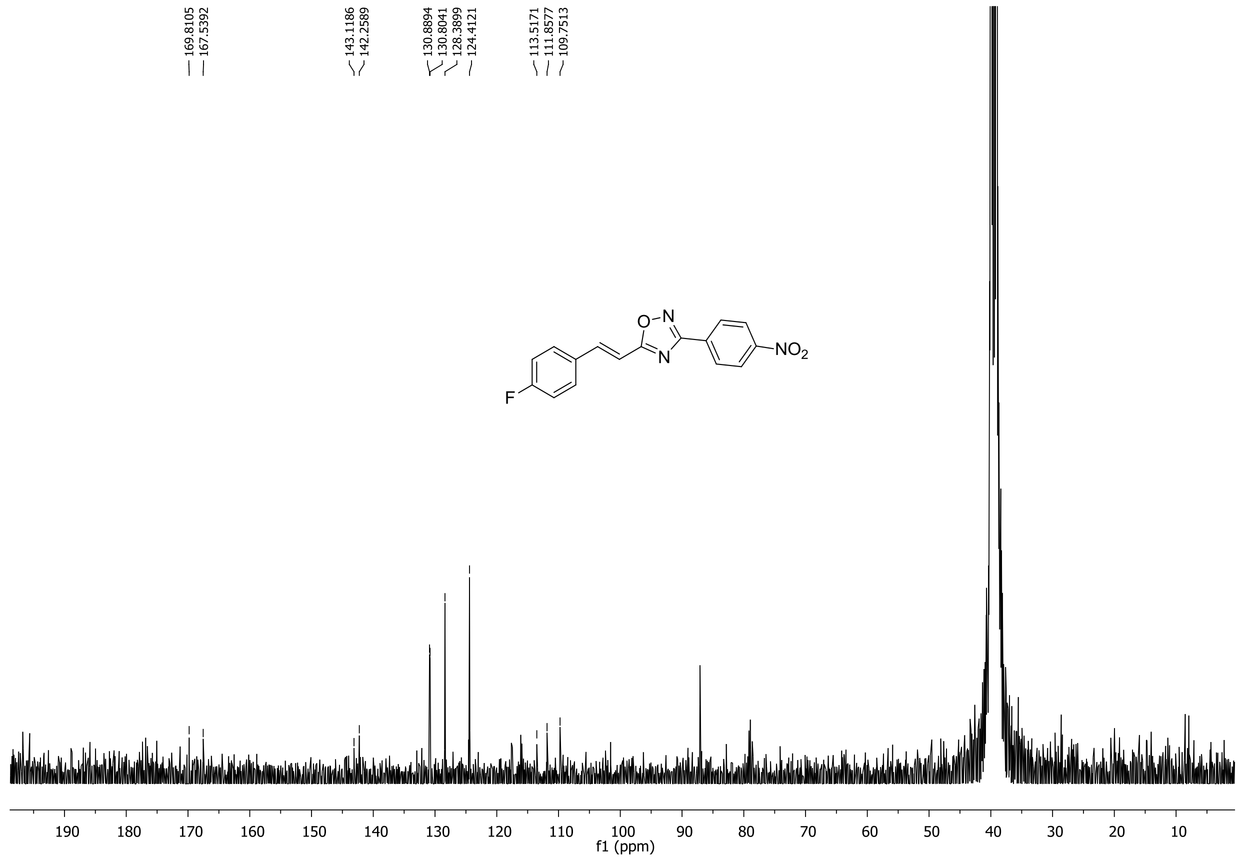

Fig. S15. ^{13}C NMR spectrum of compound **1g** [125 MHz, DMSO-d6].

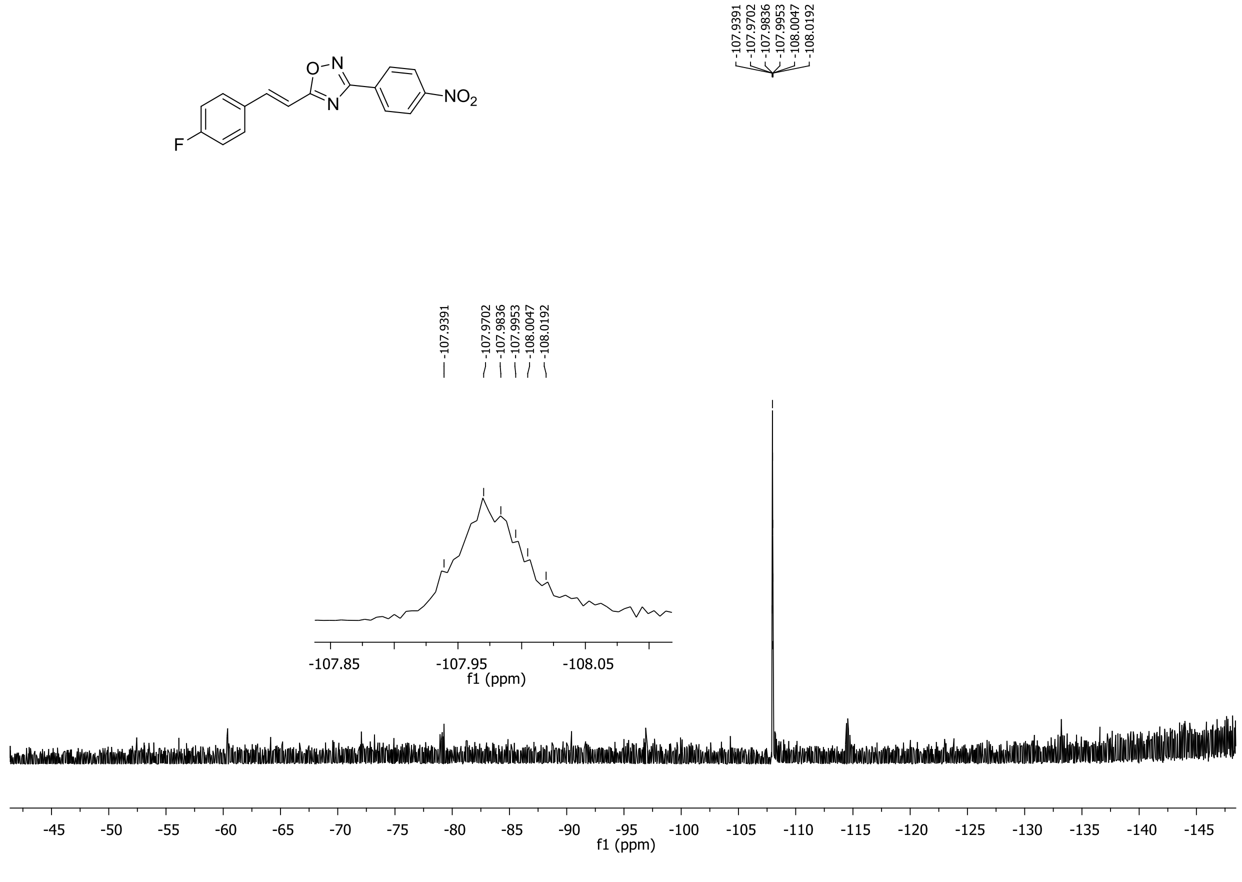

Fig. S16. ^{19}F NMR spectrum of compound **1g** [470 MHz, DMSO-d6].

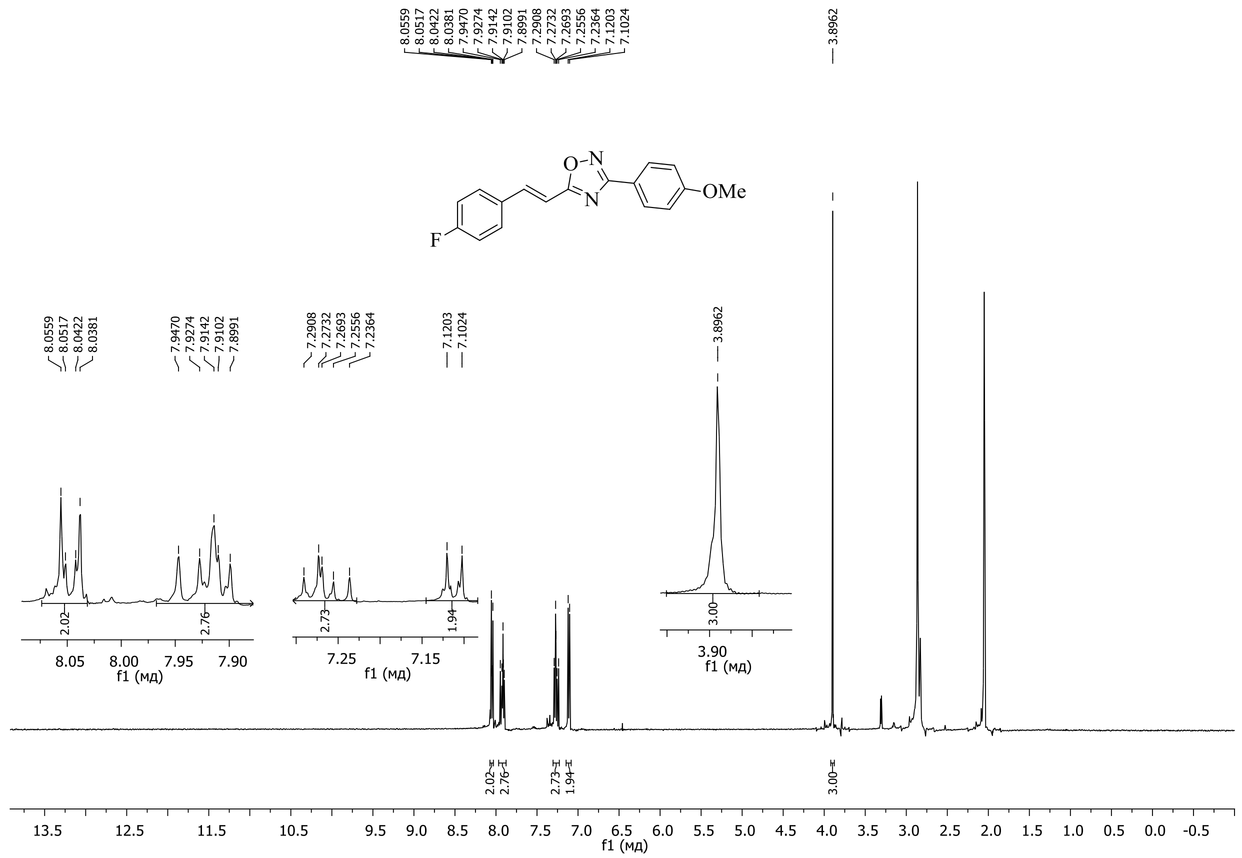

Fig. S17. ^{1}H NMR spectrum of compound **1h** [500 MHz, $(CD_3)_2CO$].

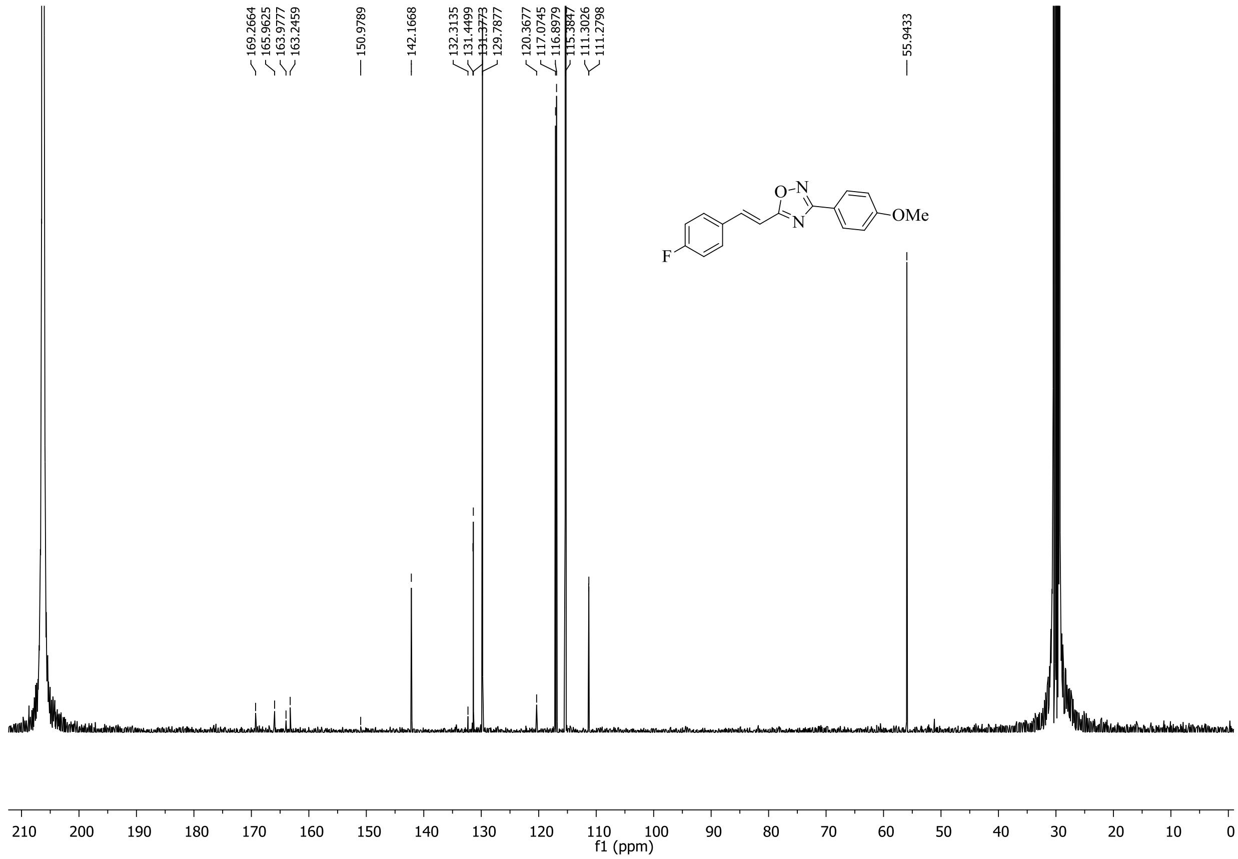

Fig. S18. ^{13}C NMR spectrum of compound **1h** [125 MHz, $(CD_3)_2CO$].

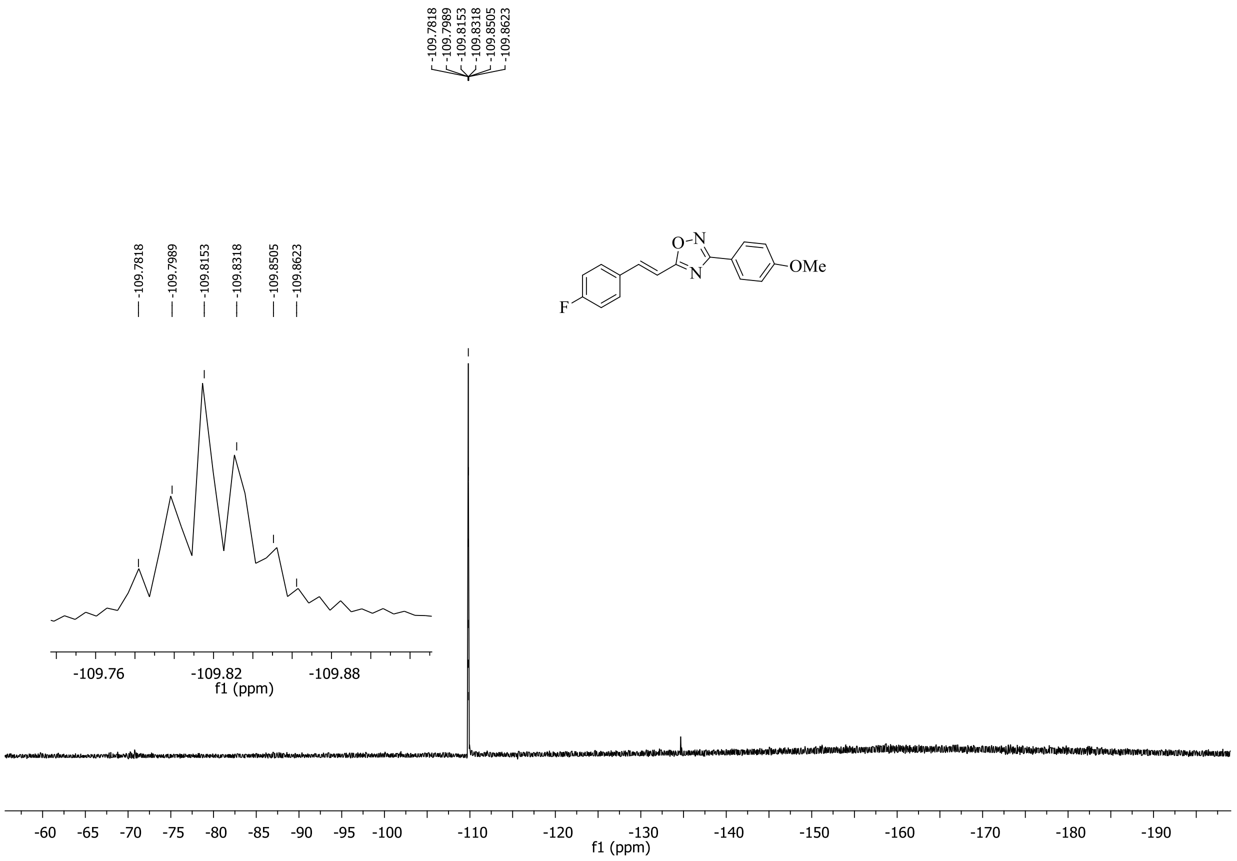

Fig. S19. ^{19}F NMR spectrum of compound **1h** [470 MHz, $(CD_3)_2CO$].

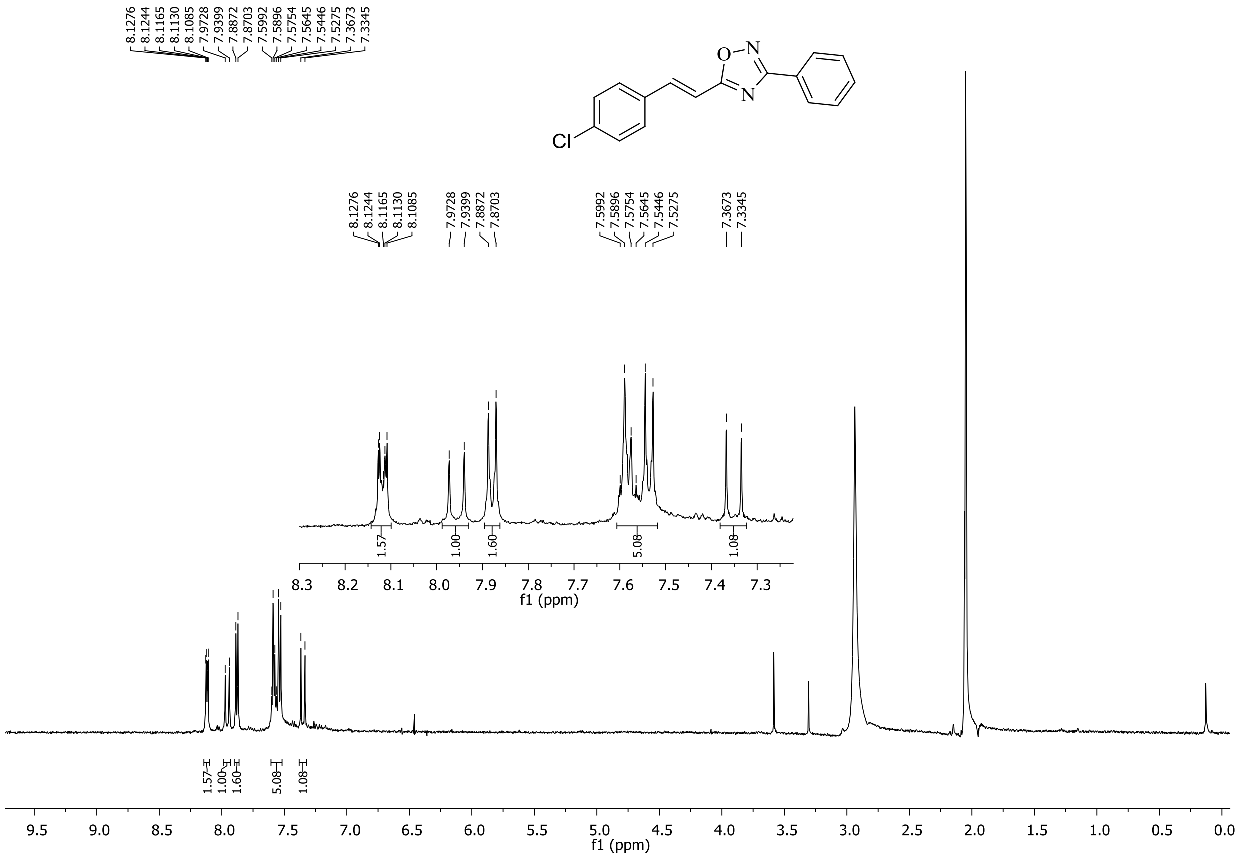

Fig. S20. ^{1}H NMR spectrum of compound **1i** [500 MHz, $(CD_3)_2CO$].

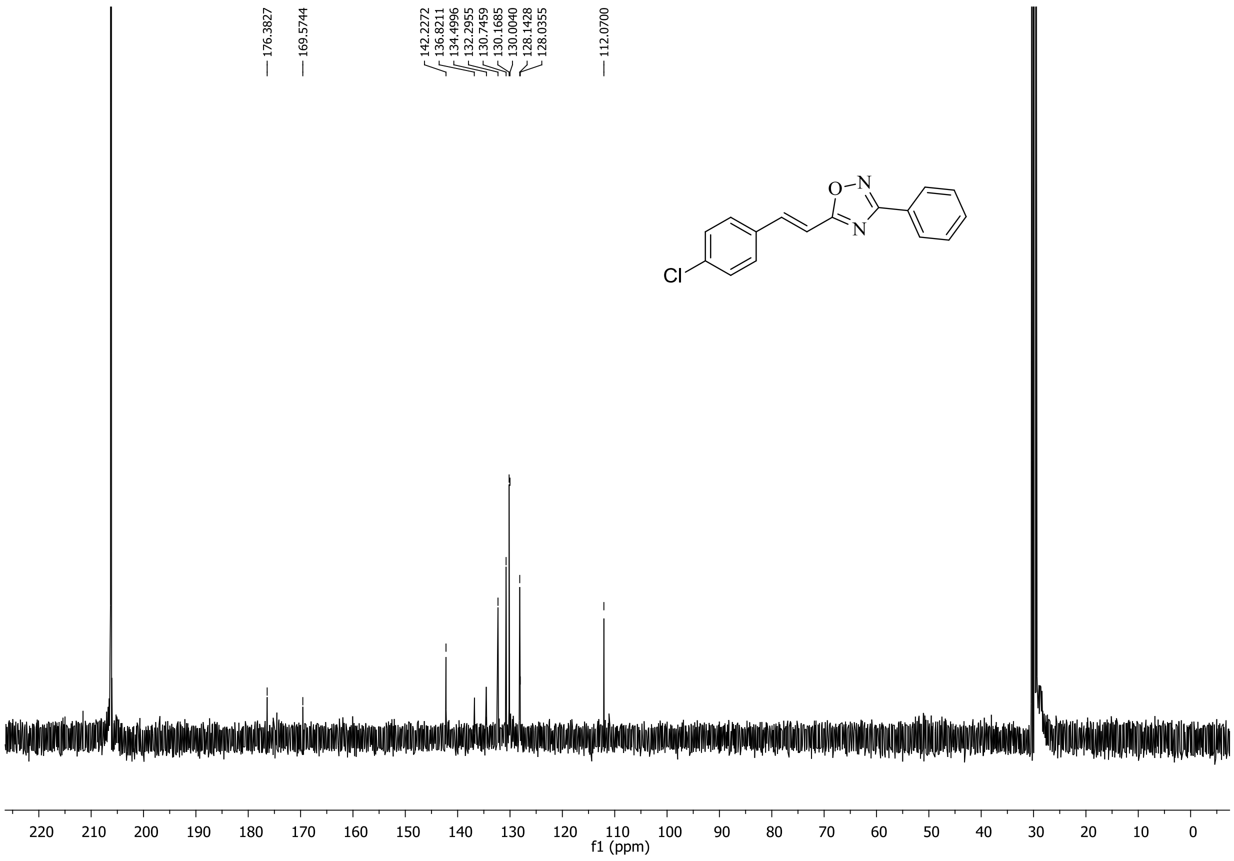

Fig. S21. ^{13}C NMR spectrum of compound **1i** [125 MHz, $(CD_3)_2CO$].

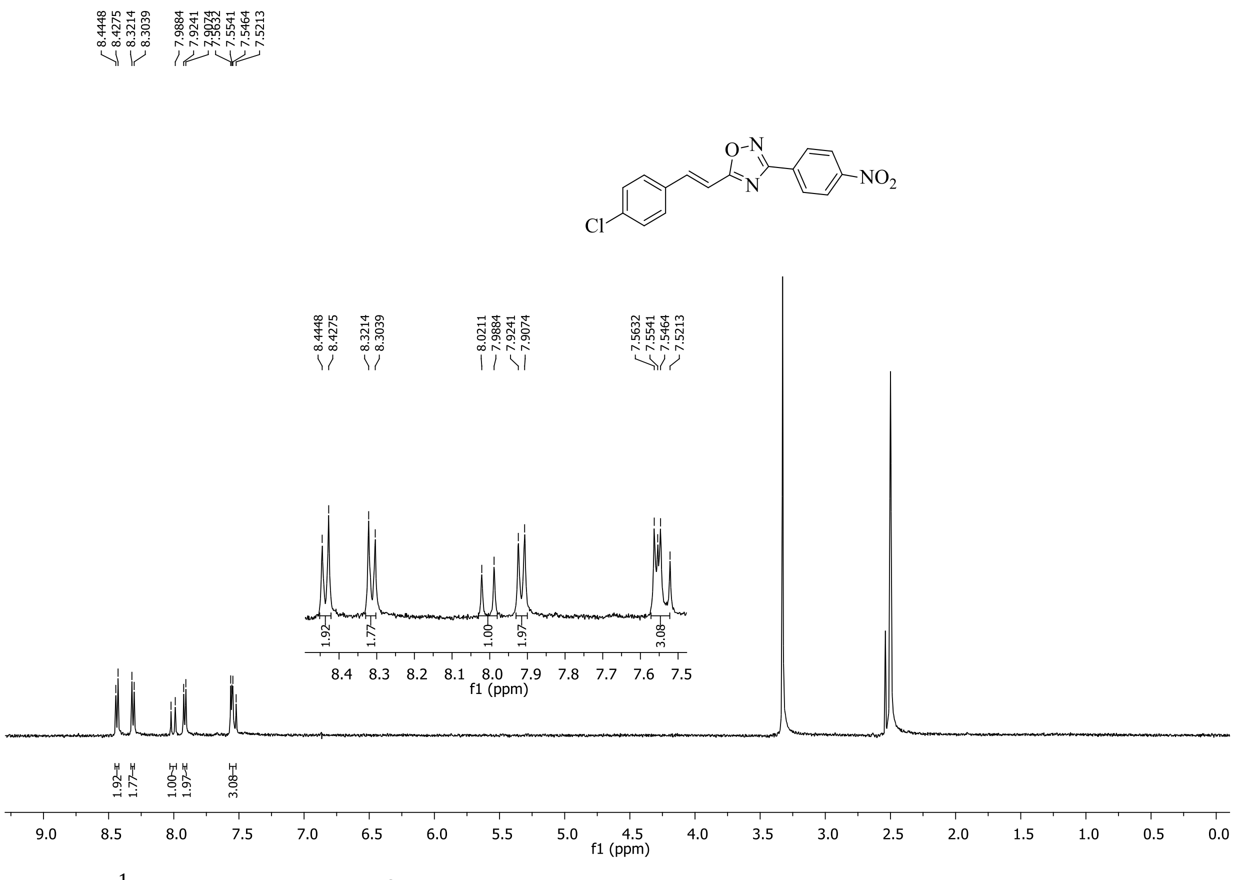

Fig. S22. ^{1}H NMR spectrum of compound **1j** [500 MHz, DMSO-d6].

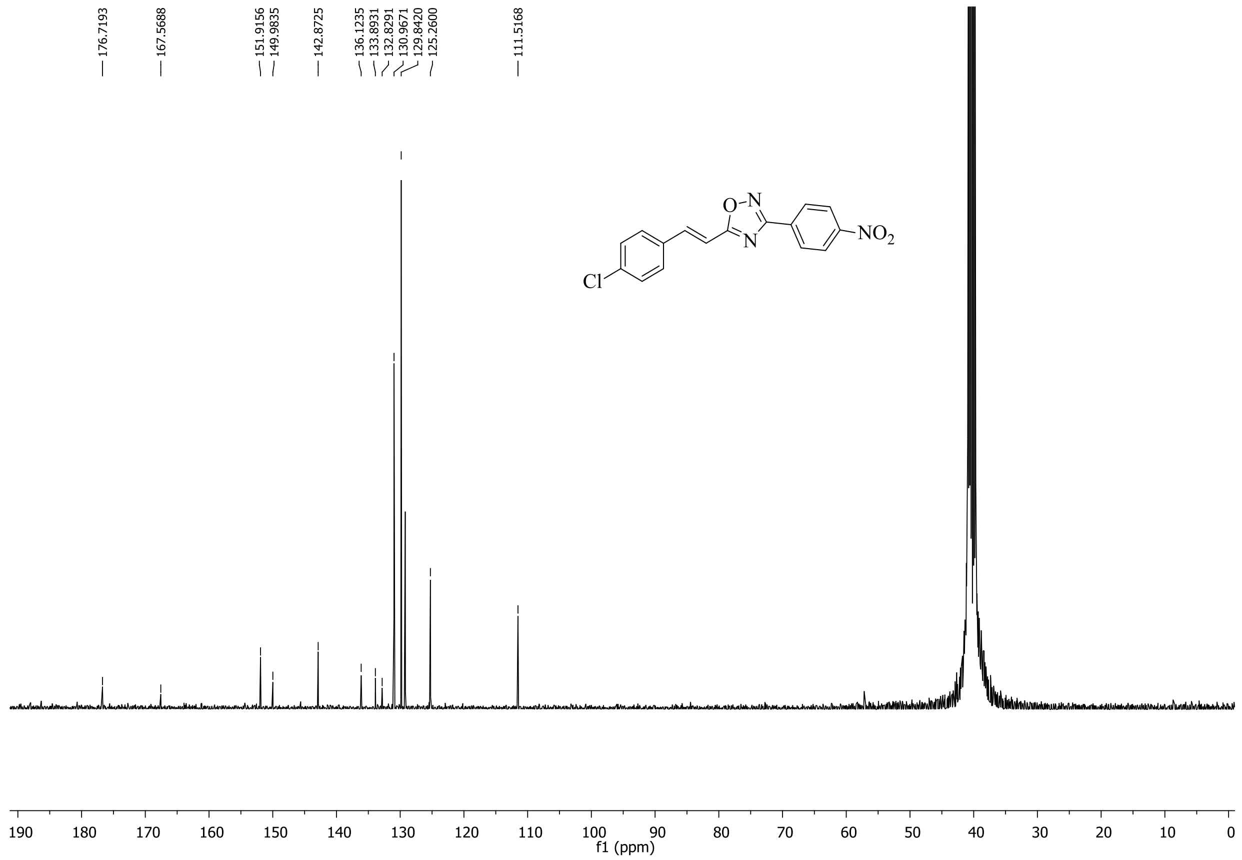

Fig. S23. ^{13}C NMR spectrum of compound **1j** [125 MHz, DMSO-d6].

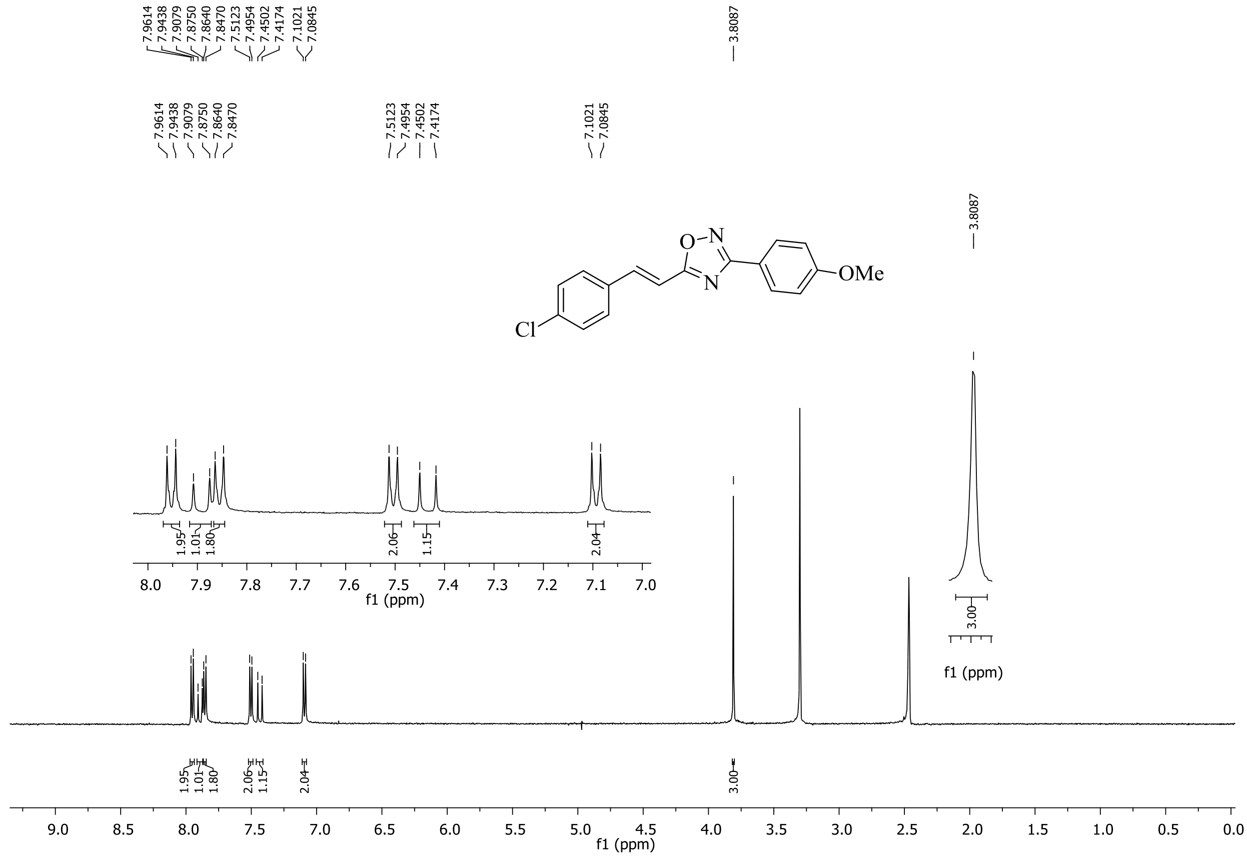

Fig. S24. ^{1}H NMR spectrum of compound **1k** [500 MHz, DMSO-d6].

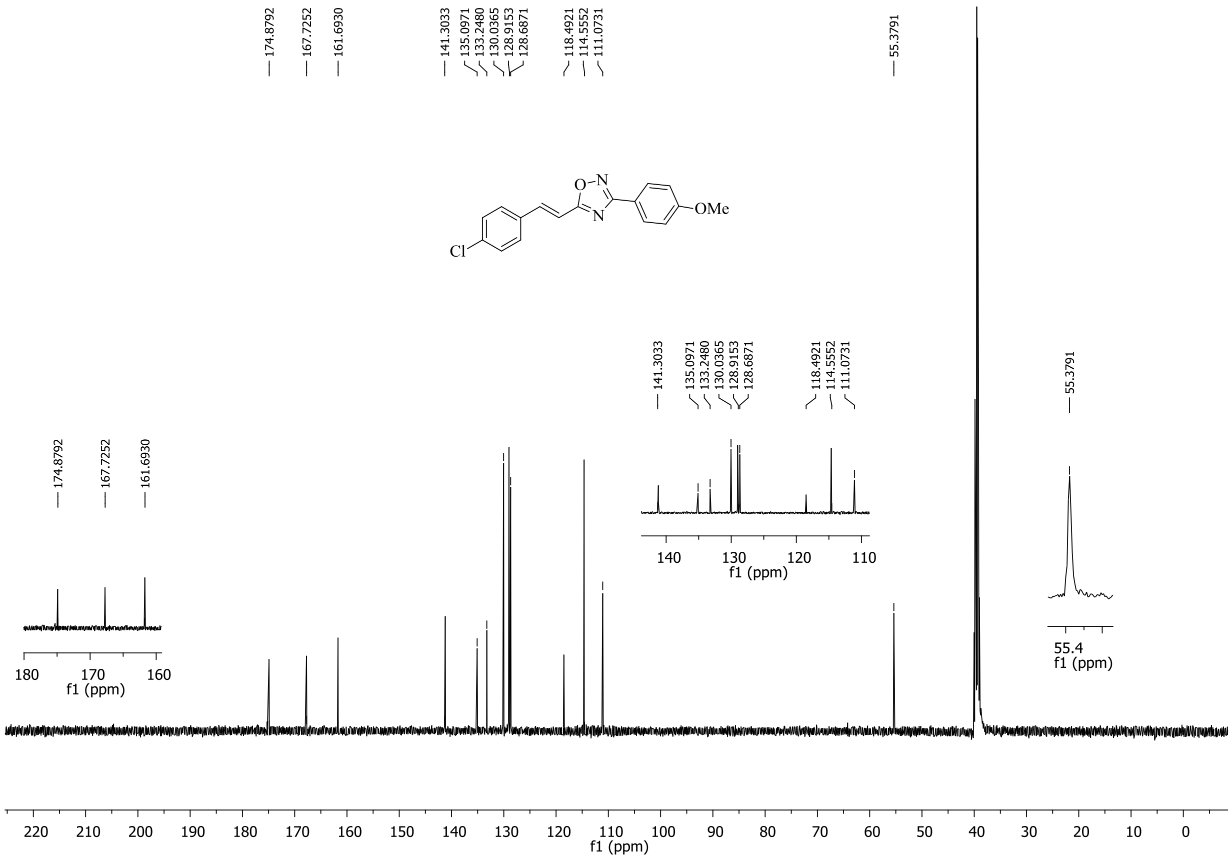

Fig. S25. ^{13}C NMR spectrum of compound **1k** [125 MHz, DMSO-d6].

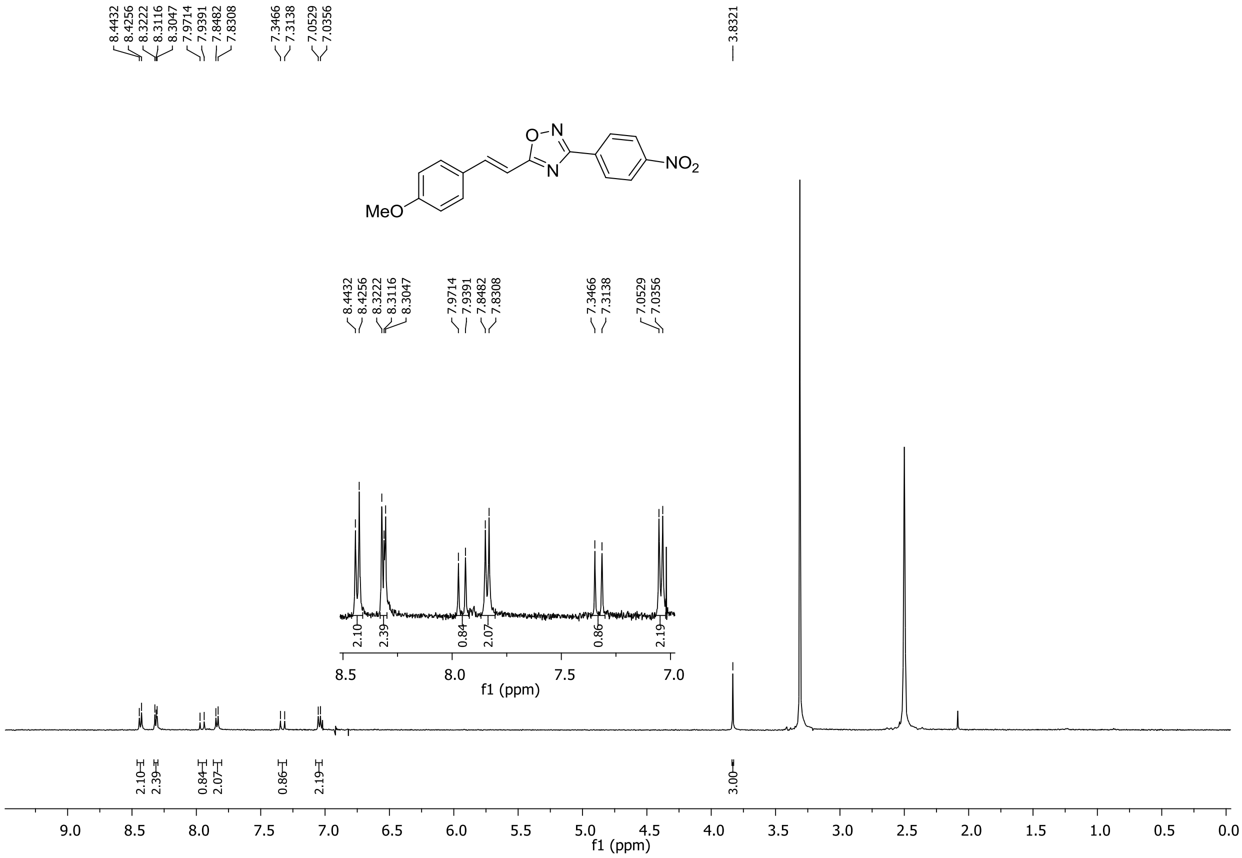

Fig. S26. ^{1}H NMR spectrum of compound **1l** [500 MHz, DMSO-d6].

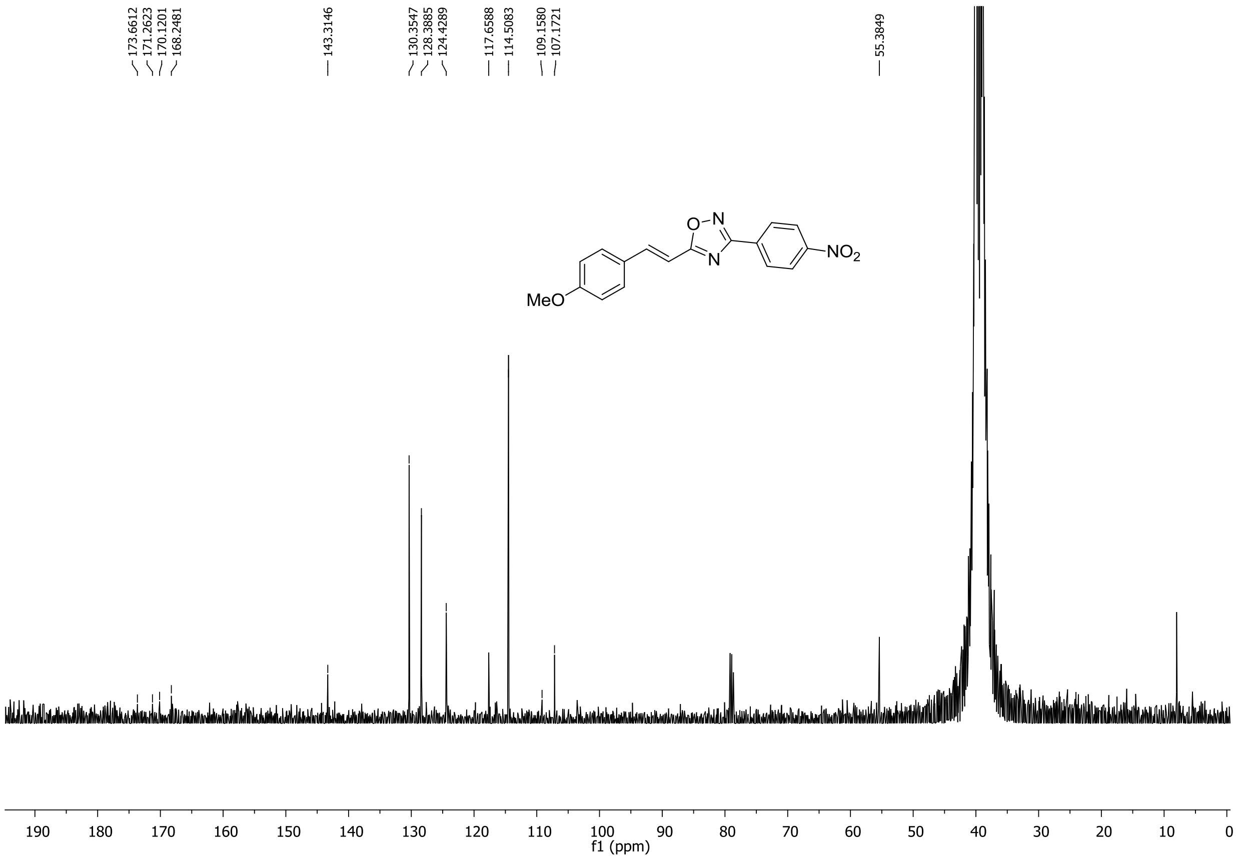

Fig. S27. ^{13}C NMR spectrum of compound **1l** [125 MHz, DMSO-d6].

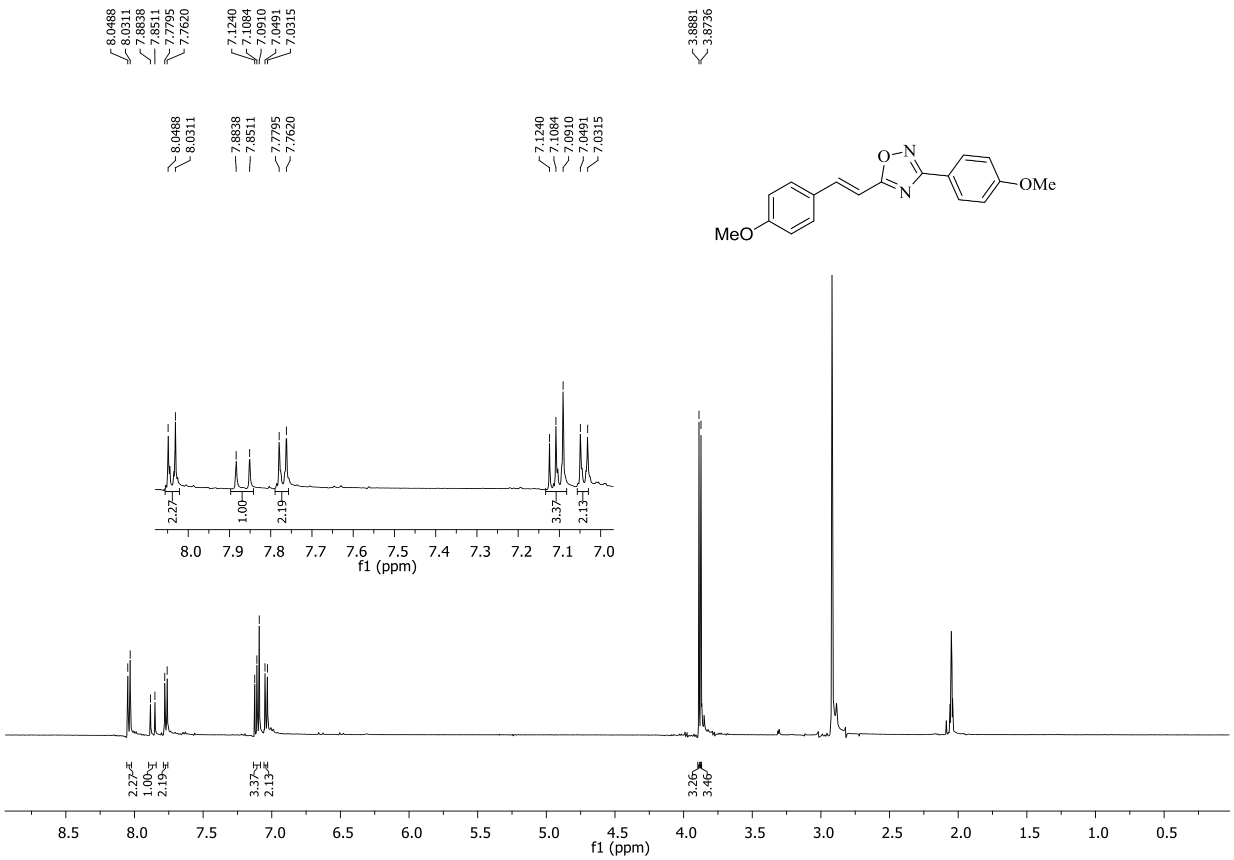

Fig. S28. ^{1}H NMR spectrum of compound **1m** [500 MHz, $(CD_3)_2CO$].

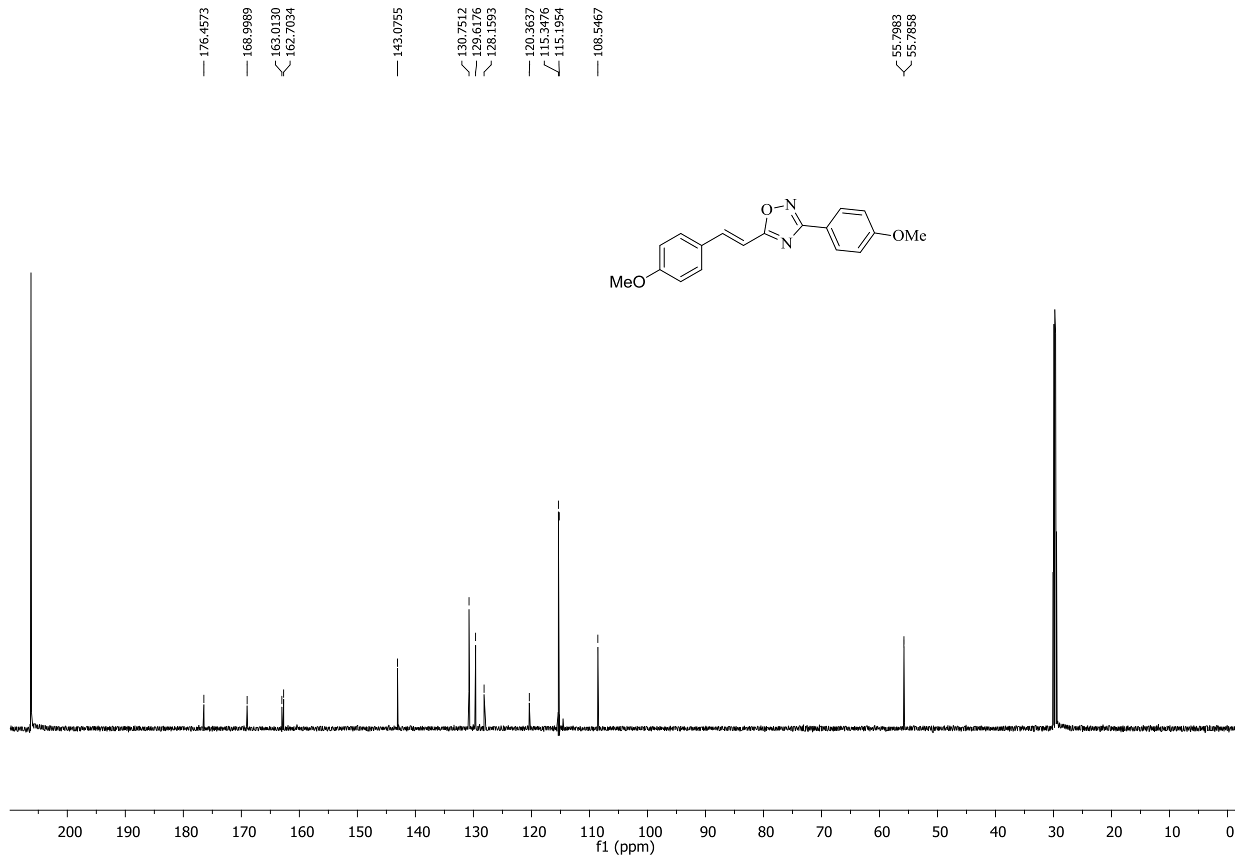

Fig. S29. ^{13}C NMR spectrum of compound **1m** [125 MHz, $(CD_3)_2CO$].

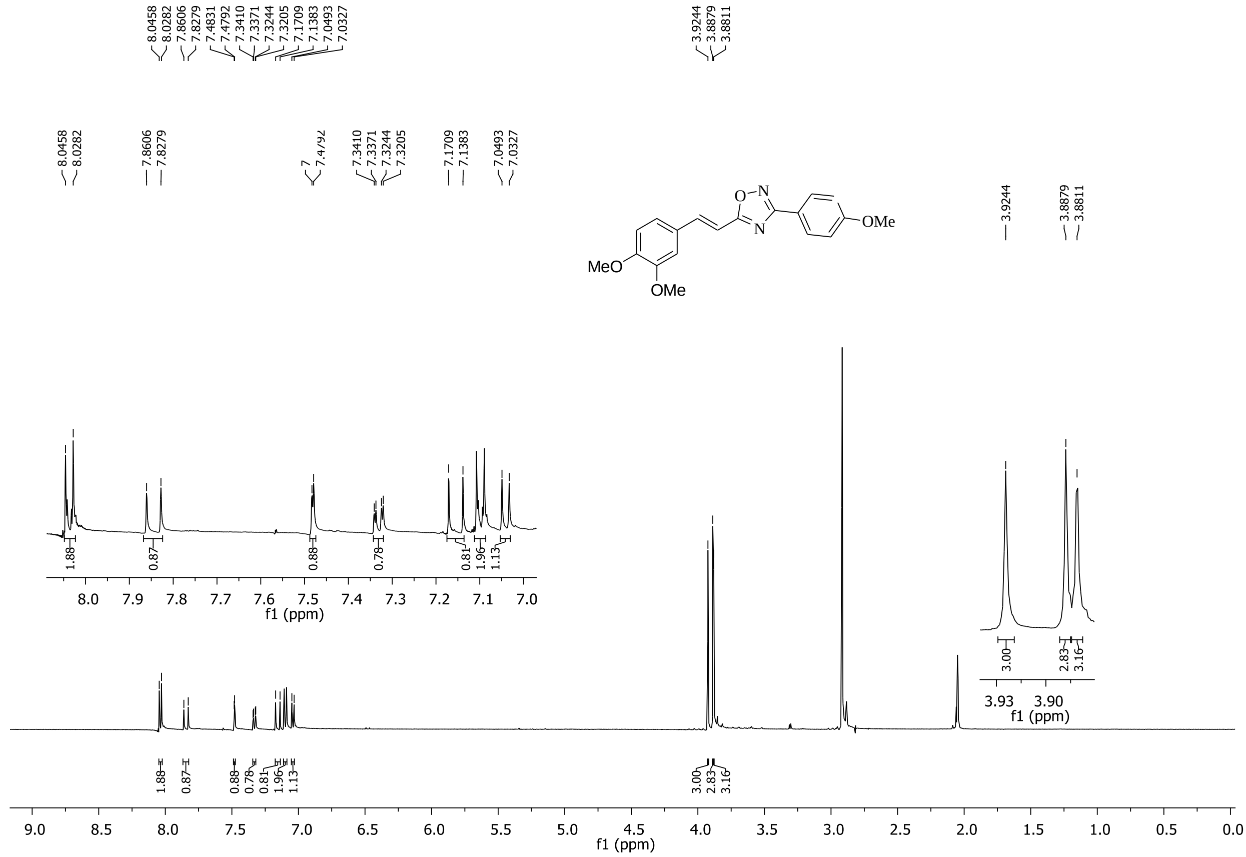

Fig. S30. ^{1}H NMR spectrum of compound **1n** [500 MHz, $(CD_3)_2CO$].

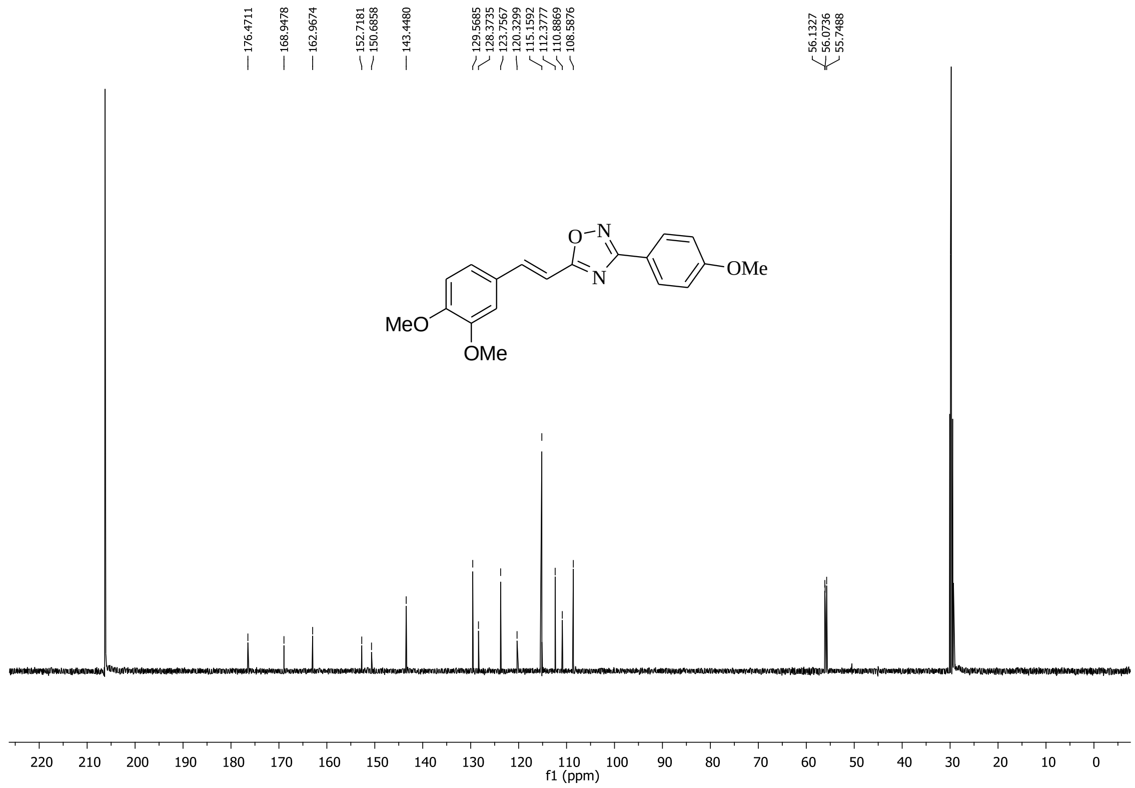

Fig. S31. ^{13}C NMR spectrum of compound **1n** [125 MHz, $(CD_3)_2CO$].

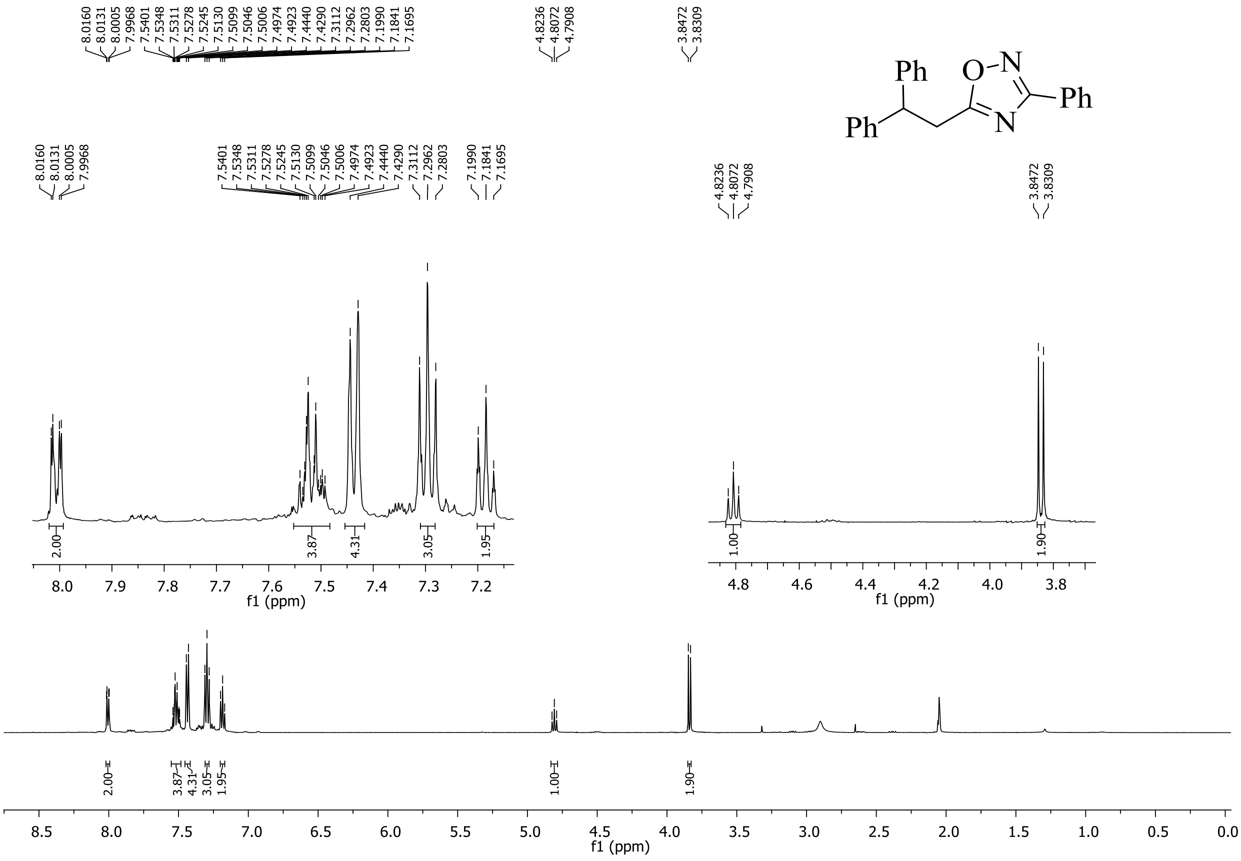

Fig. S32. ^{1}H NMR spectrum of compound **2a** [500 MHz, $(CD_3)_2CO$].

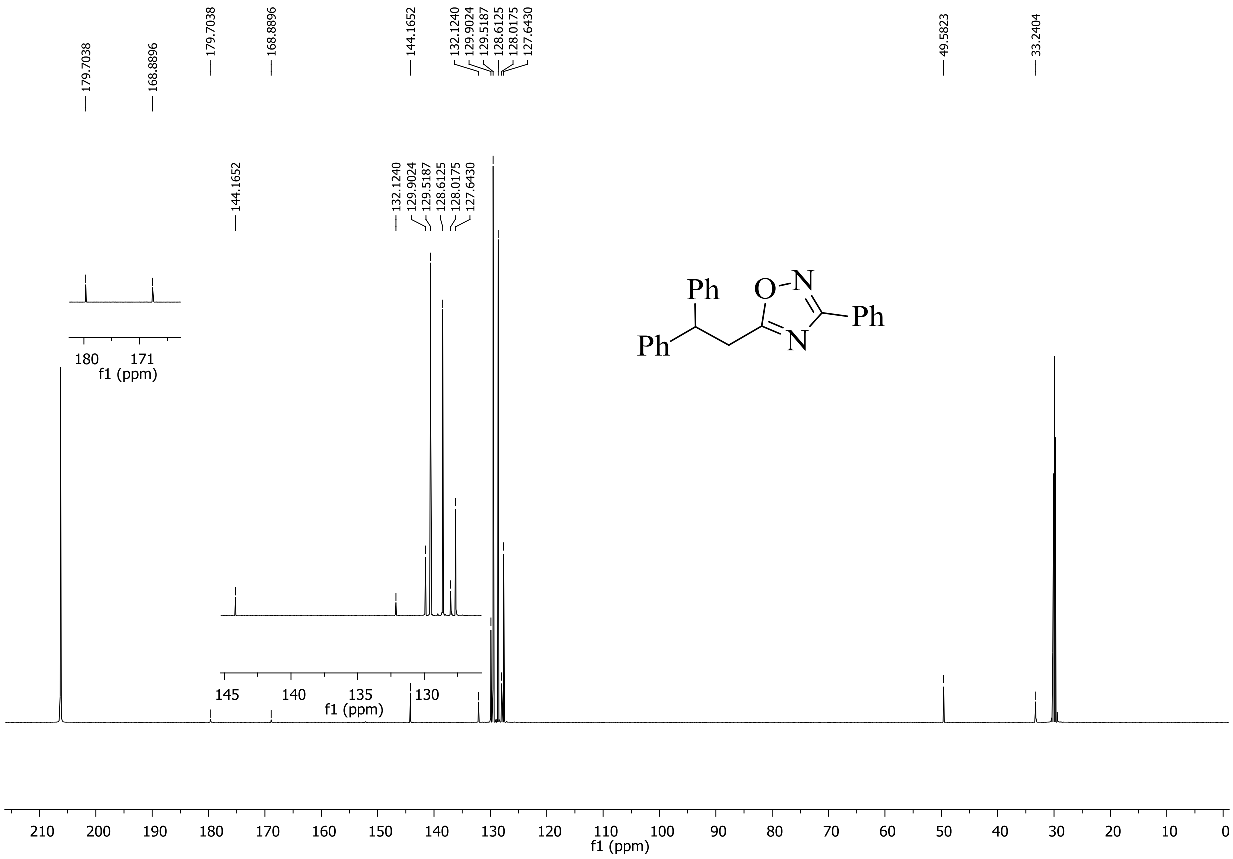

Fig. S33. ^{13}C NMR spectrum of compound **2a** [125 MHz, $(CD_3)_2CO$].

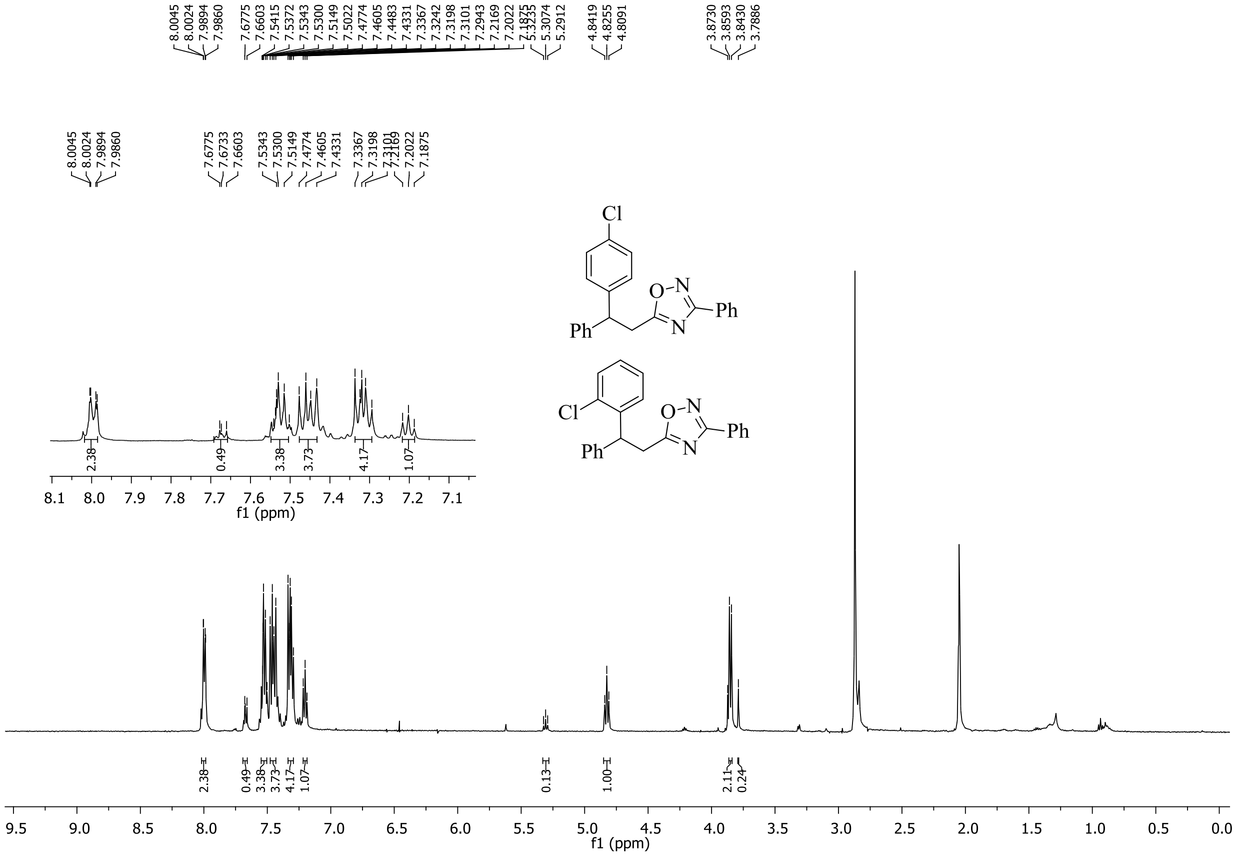

Fig. S34. ^{1}H NMR spectrum of compound **2b, 2c** [500 MHz, $(CD_3)_2CO$].

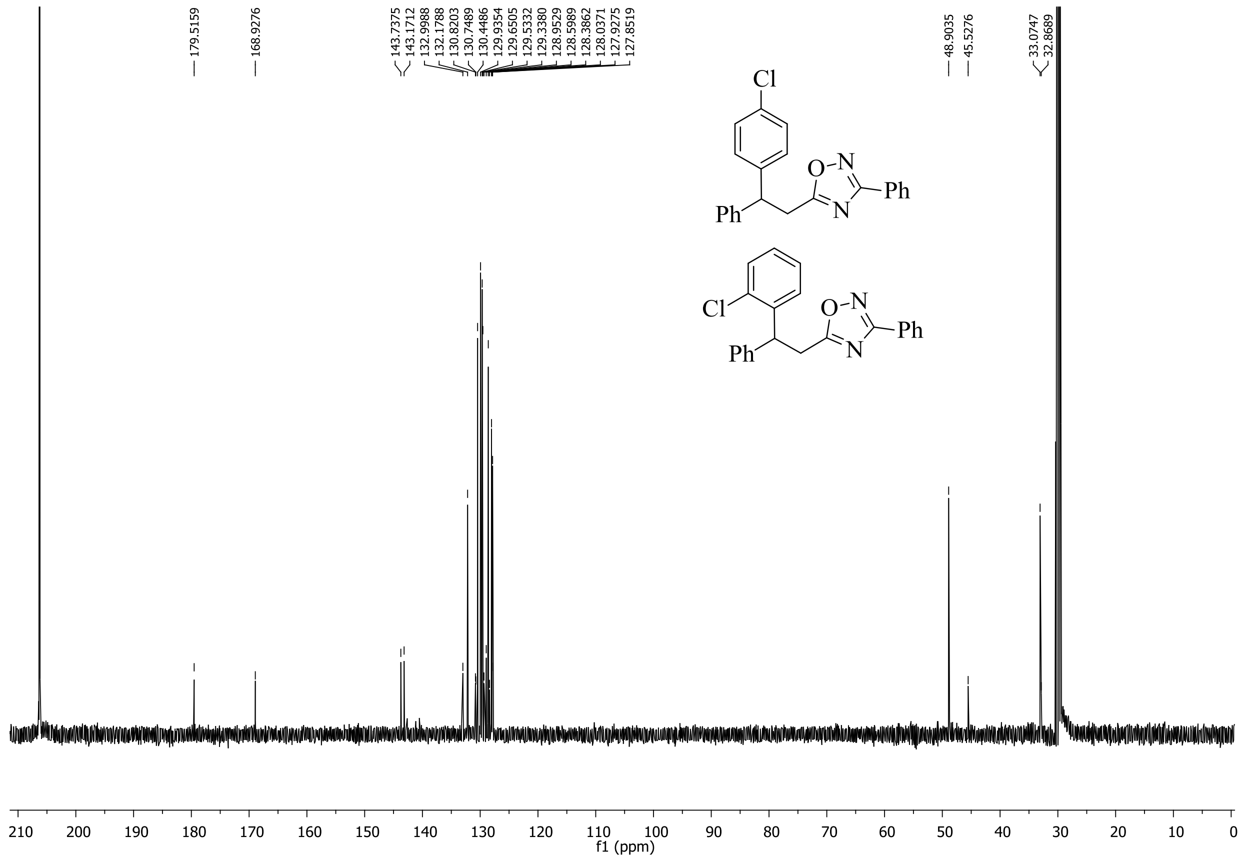

Fig. S35. ^{13}C NMR spectrum of compound **2b, 2c** [125 MHz, $(CD_3)_2CO$].

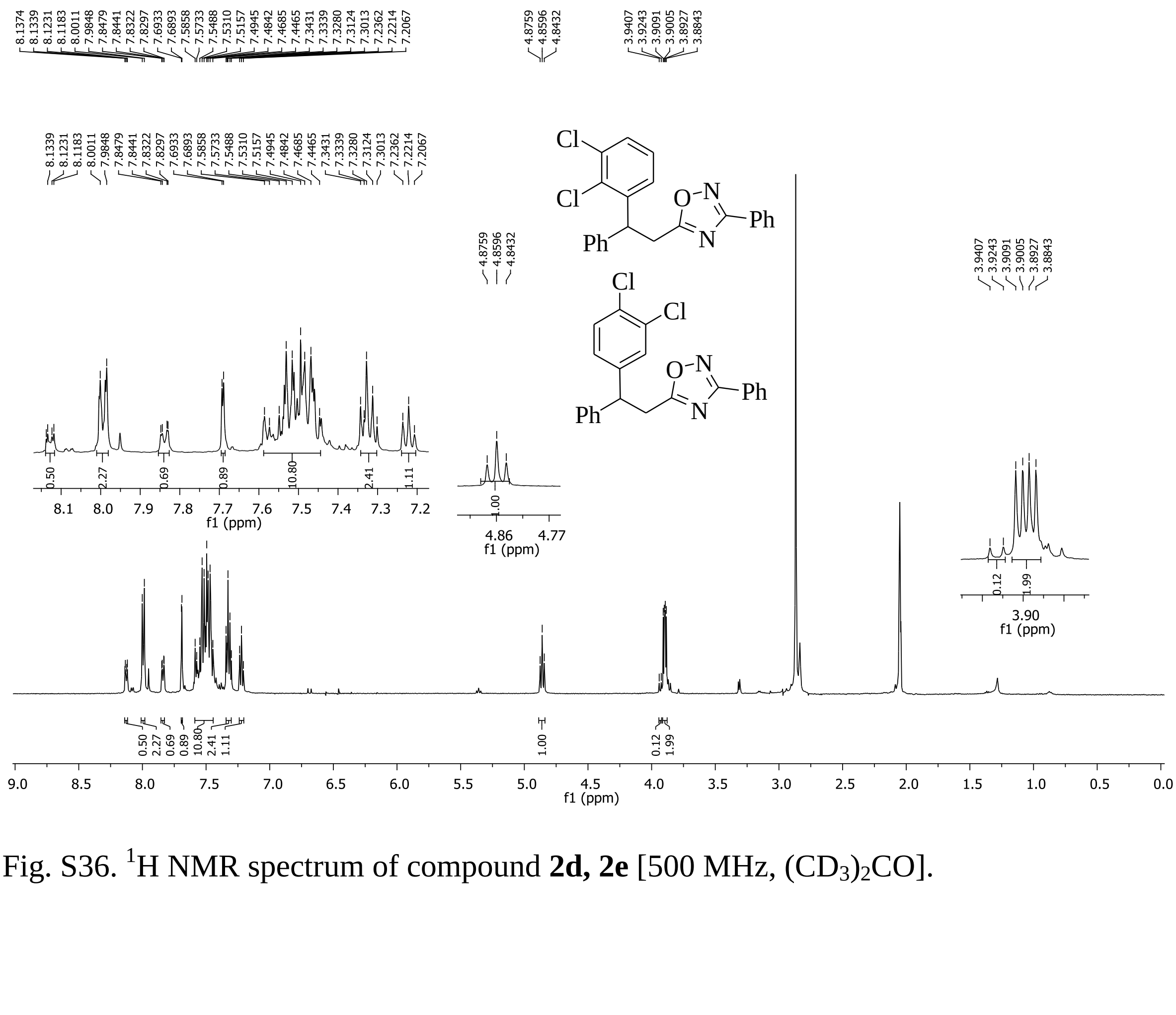

Fig. S36. ^{1}H NMR spectrum of compound **2d, 2e** [500 MHz, $(CD_3)_2CO$].

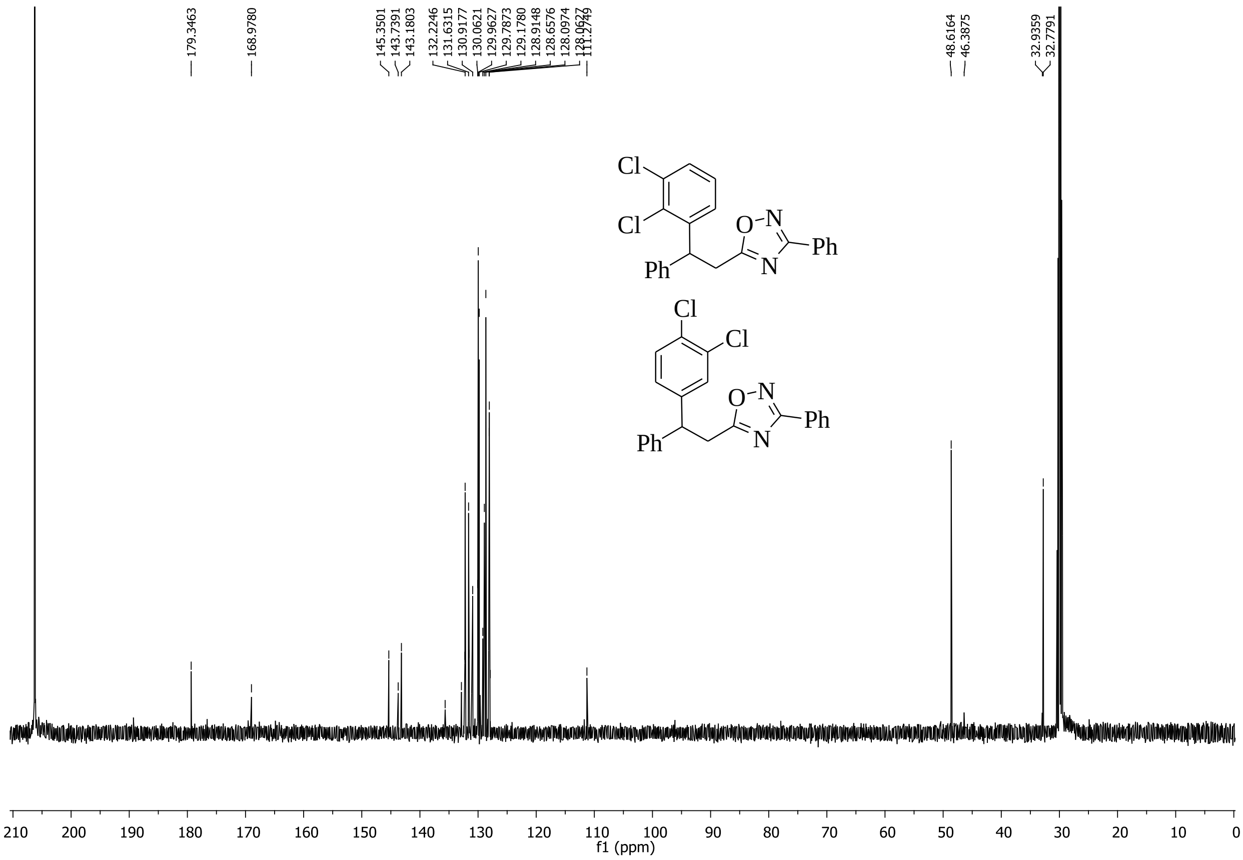

Fig. S37. ^{13}C NMR spectrum of compound **2d, 2e** [125 MHz, $(CD_3)_2CO$].

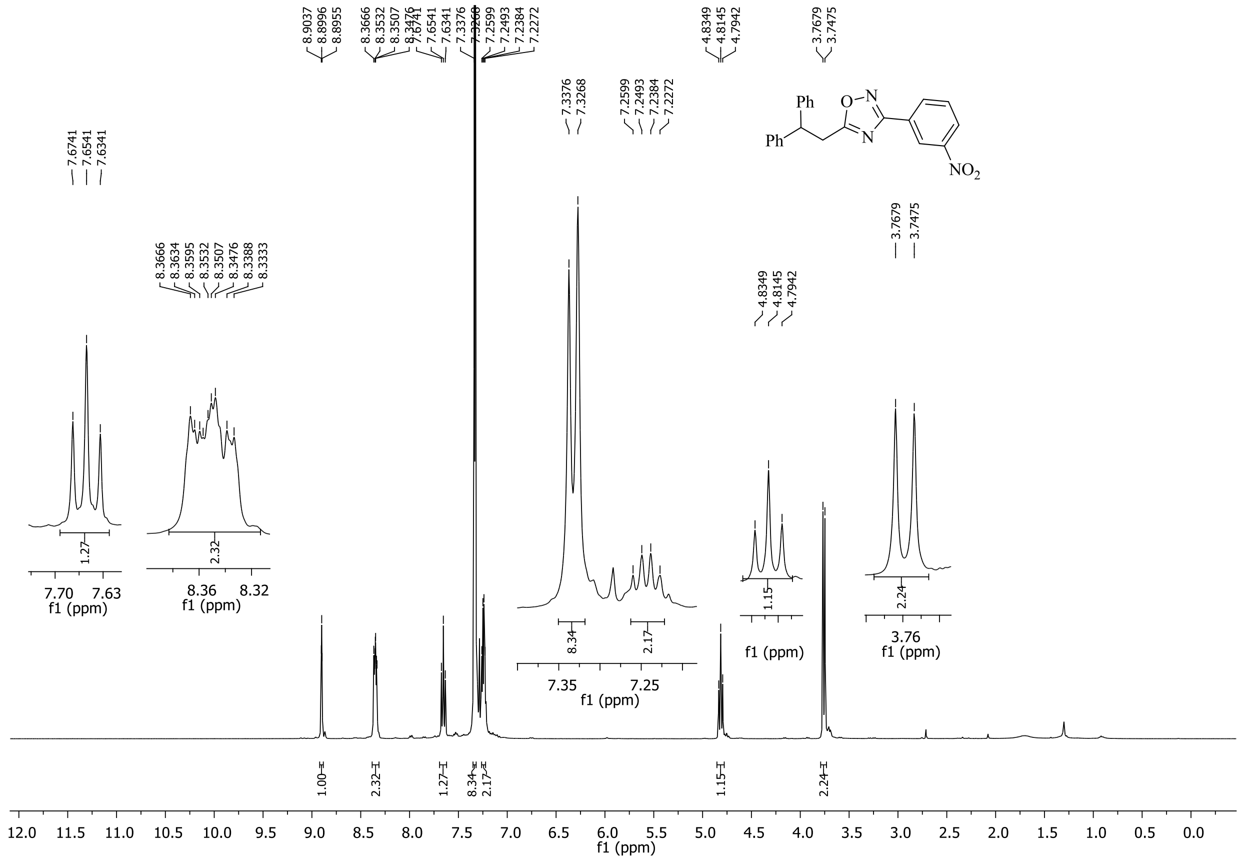

Fig. S38. ^{1}H NMR spectrum of compound **2f** [400 MHz, $CDCl_3$].

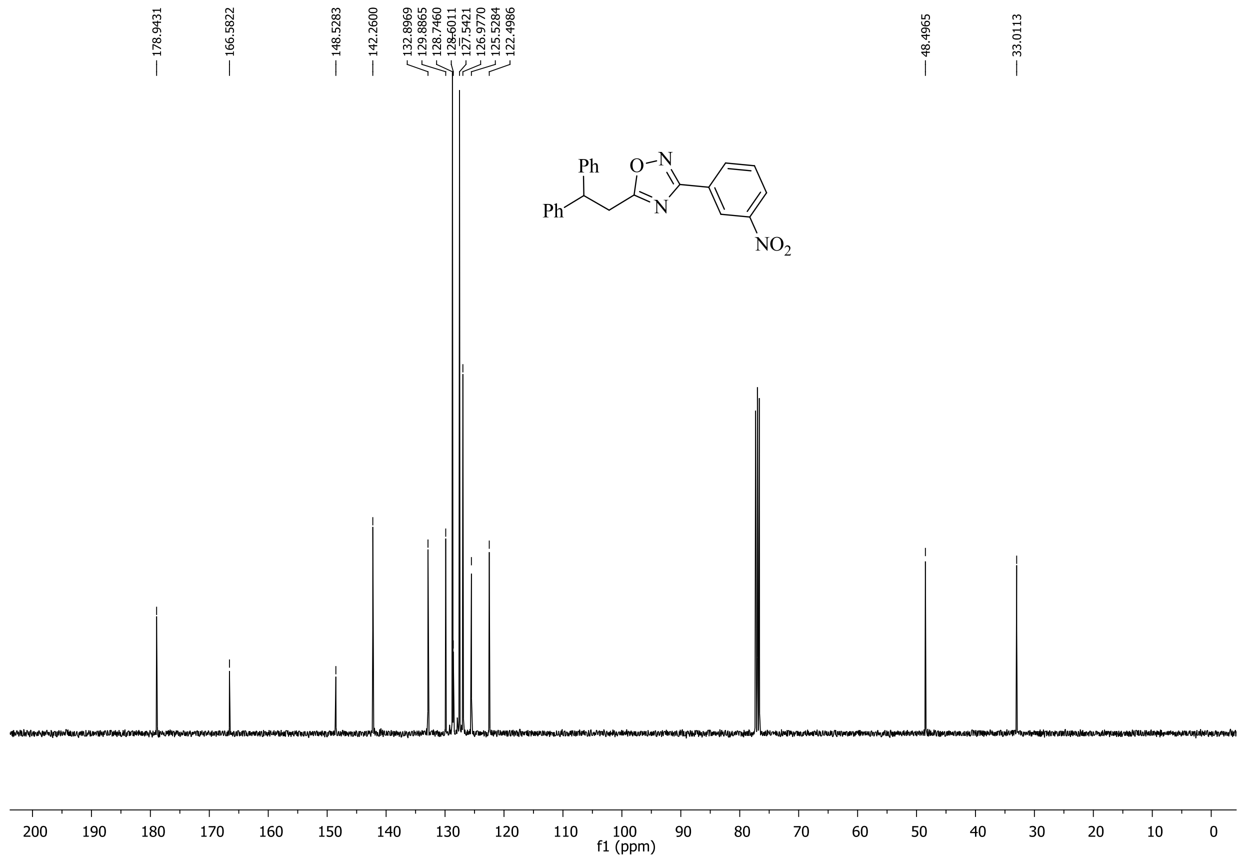

Fig. S39. ^{13}C NMR spectrum of compound **2f** [100 MHz, $CDCl_3$].

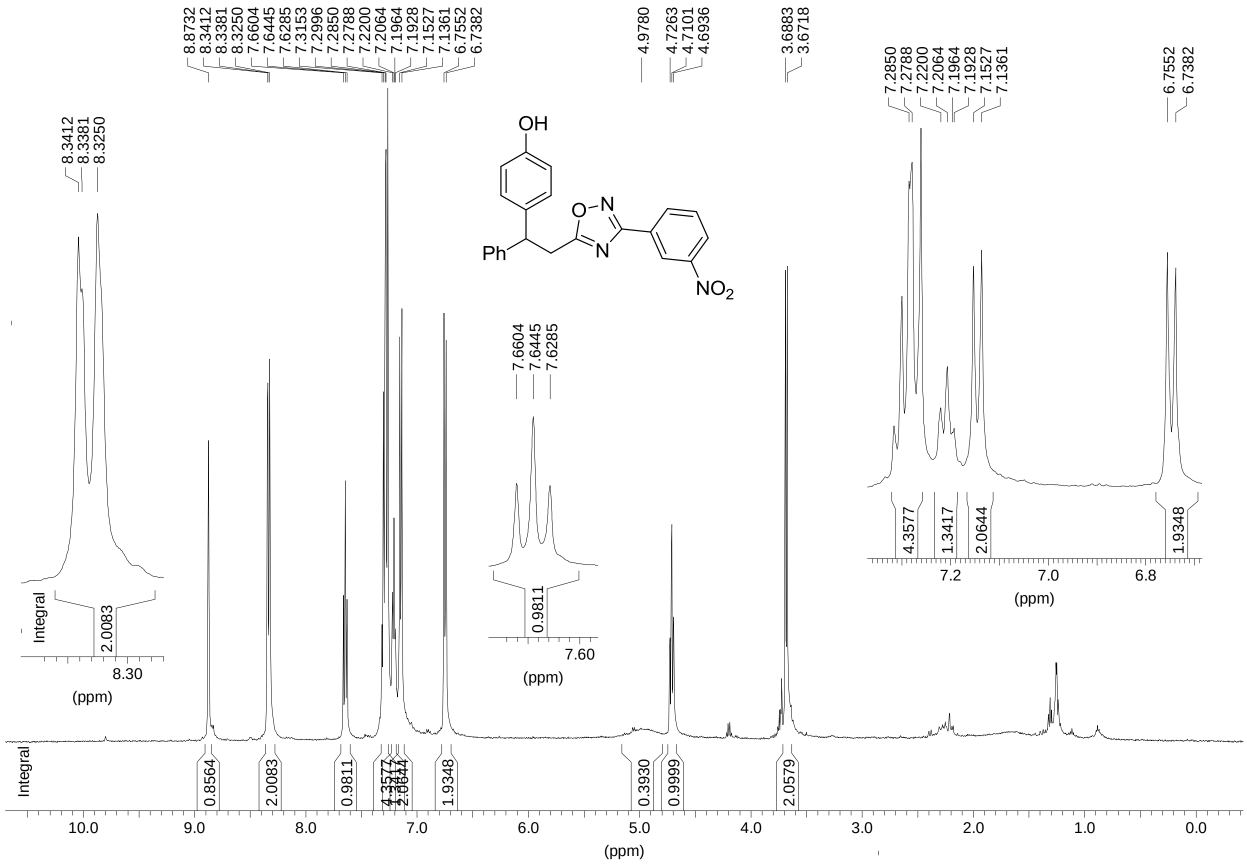

Fig. S40. ^{1}H NMR spectrum of compound **2g** [500 MHz, $CDCl_3$].

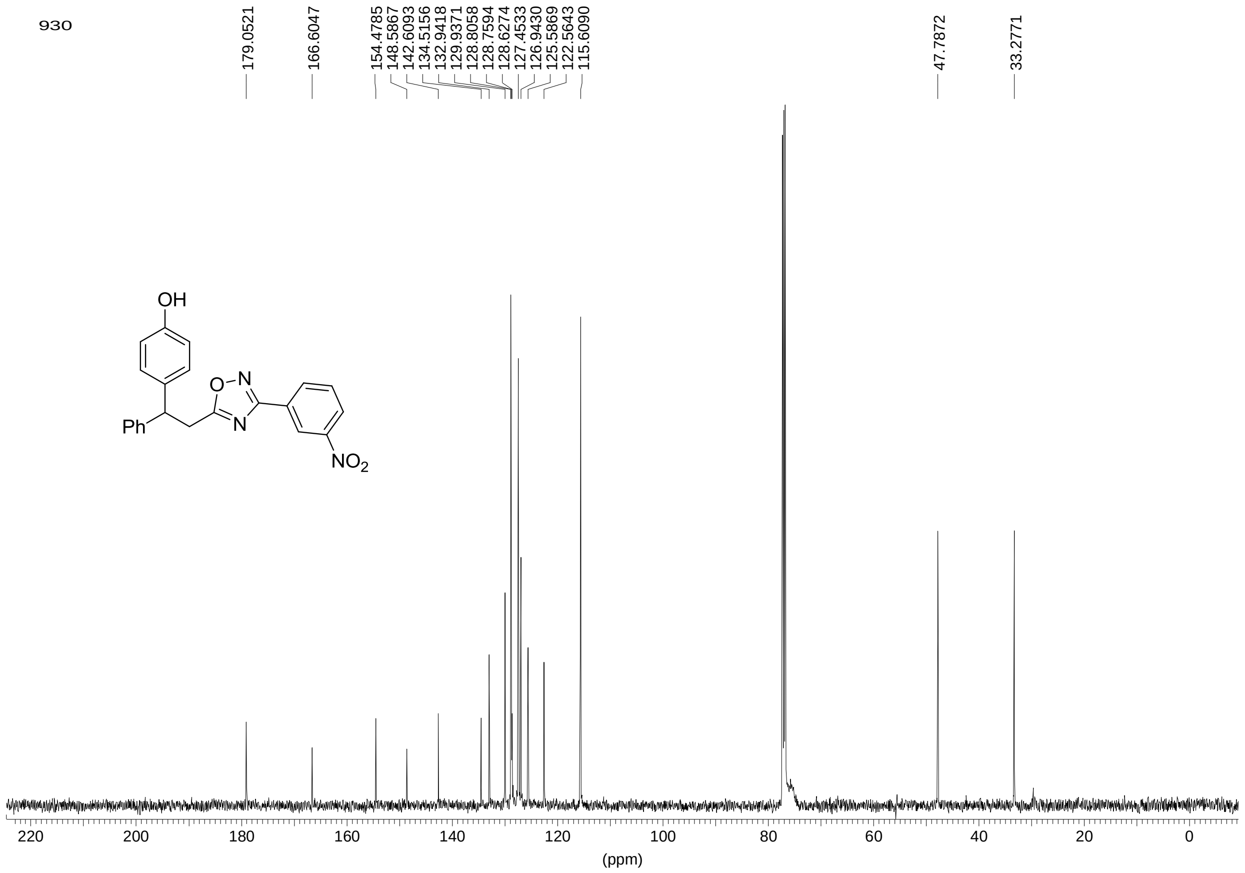

Fig. S41. ^{13}C NMR spectrum of compound **2g** [125 MHz, $CDCl_3$].

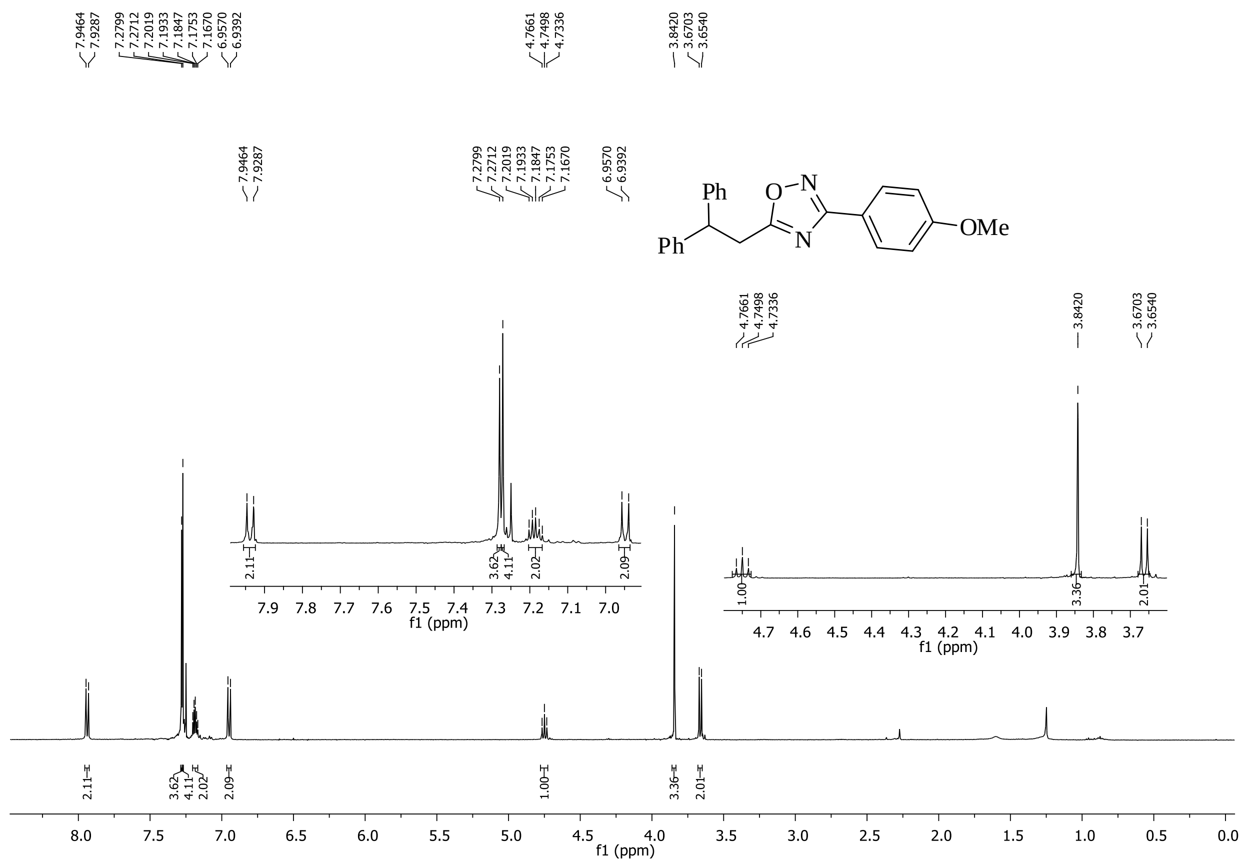

Fig. S42. ^{1}H NMR spectrum of compound **2h** [500 MHz, $CDCl_3$].

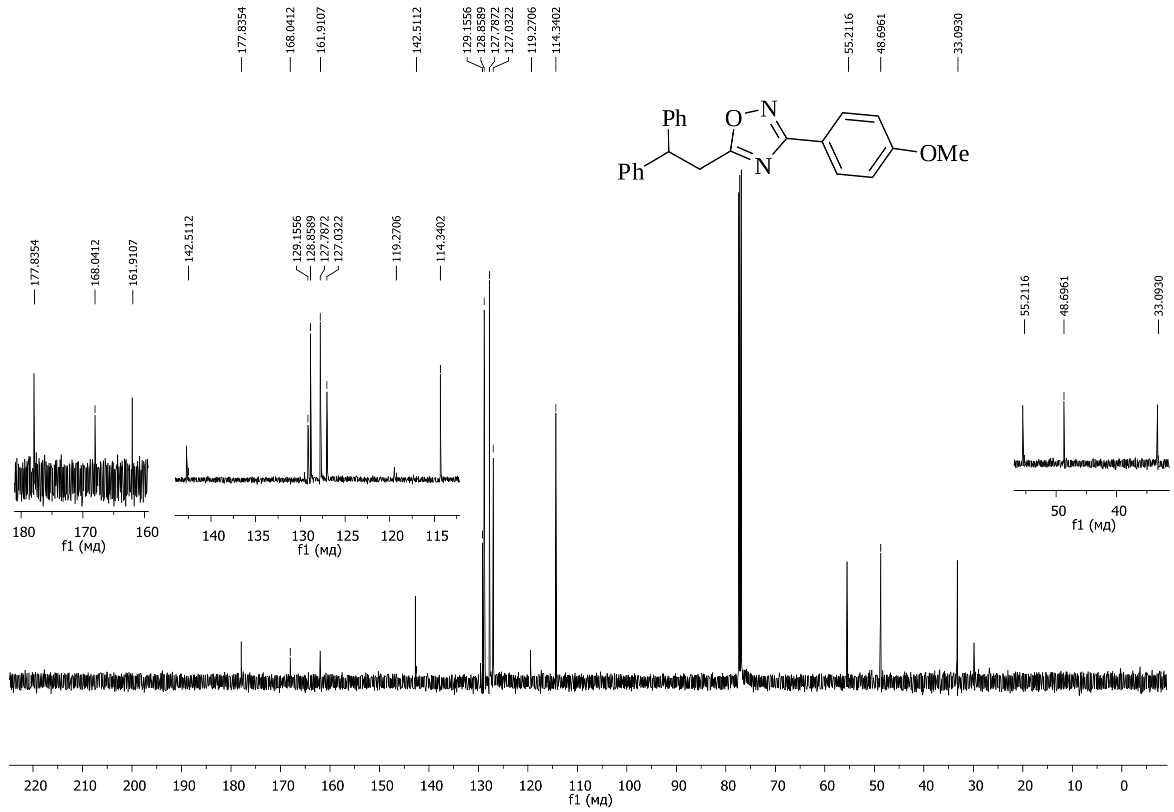

Fig. S43. ^{13}C NMR spectrum of compound **2h** [125 MHz, $CDCl_3$].

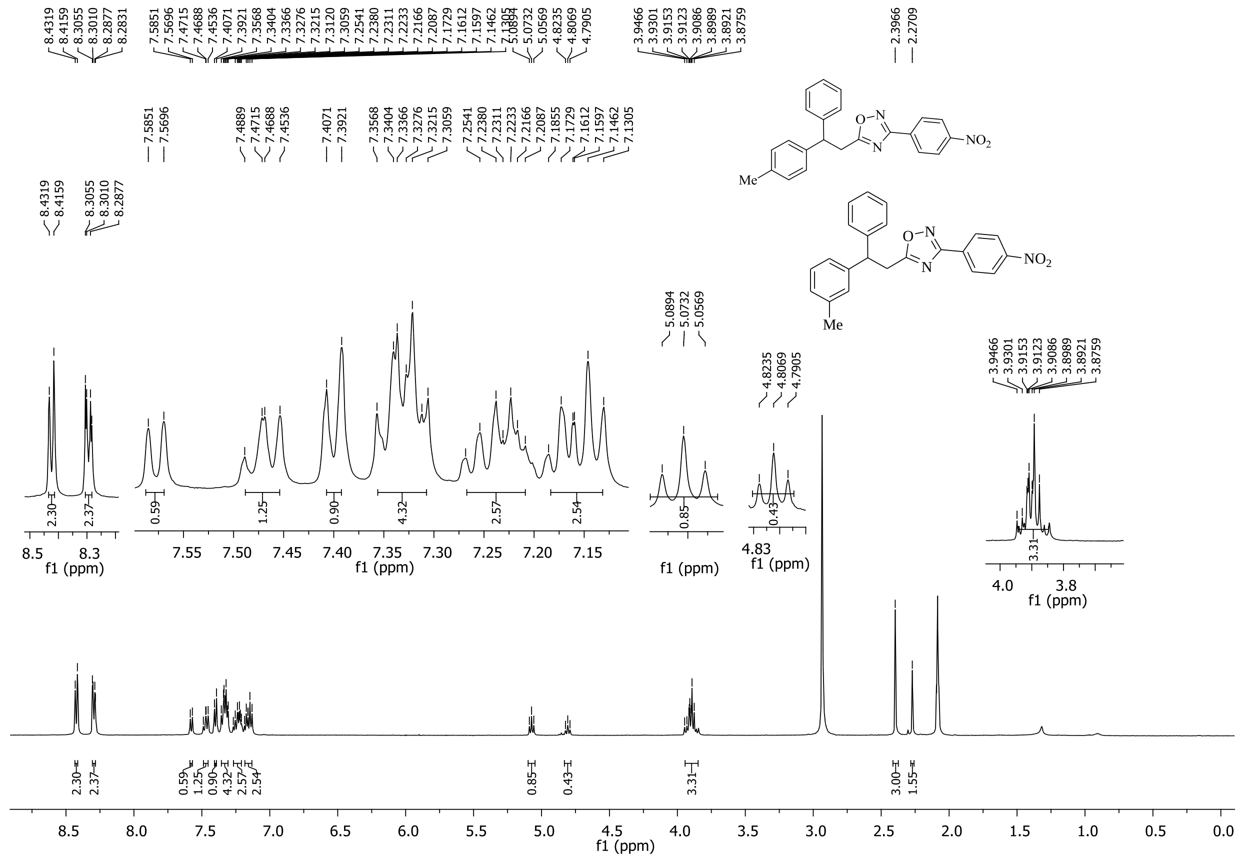

Fig. S44. ^{1}H NMR spectrum of compounds **2i, 2j** [500 MHz, $(CD_3)_2CO$].

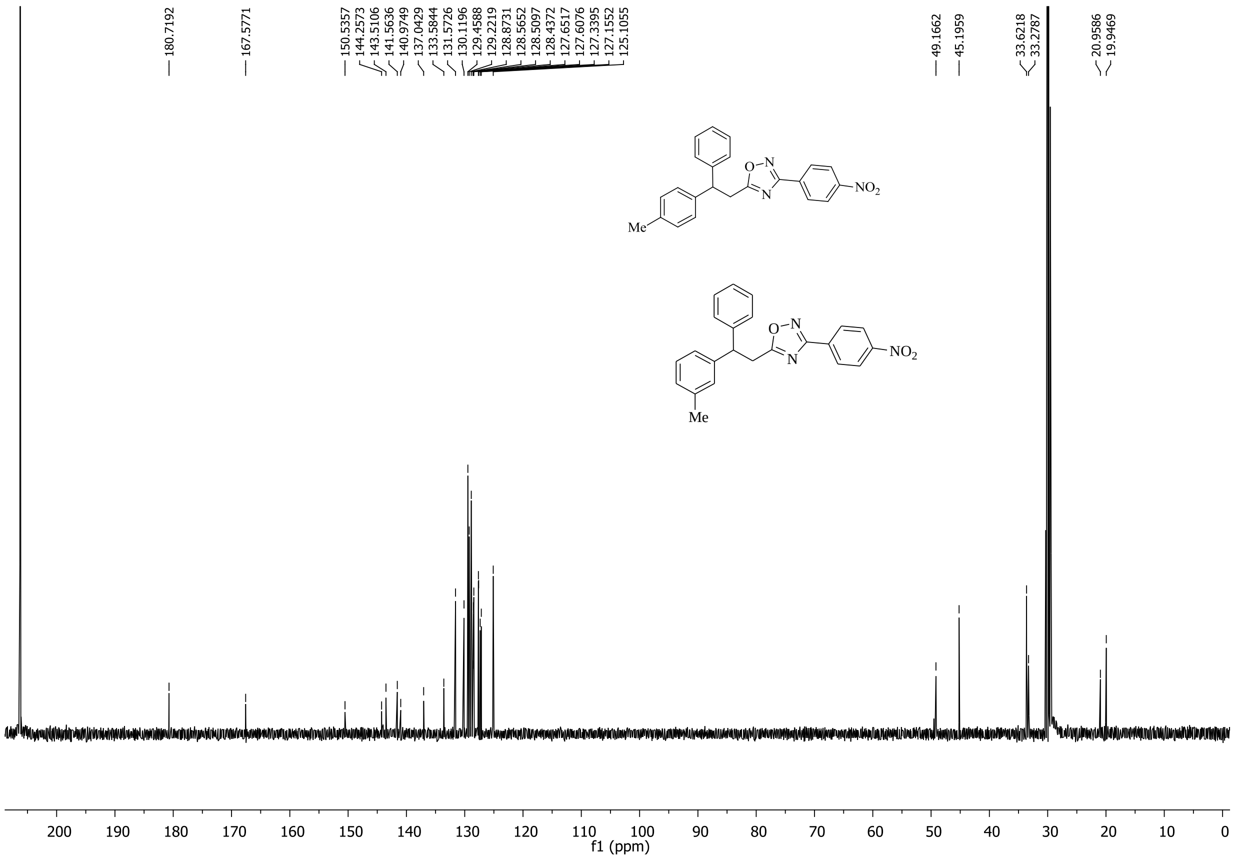

Fig. S45. ^{13}C NMR spectrum of compounds **2i, 2j** [125 MHz, $(CD_3)_2CO$].

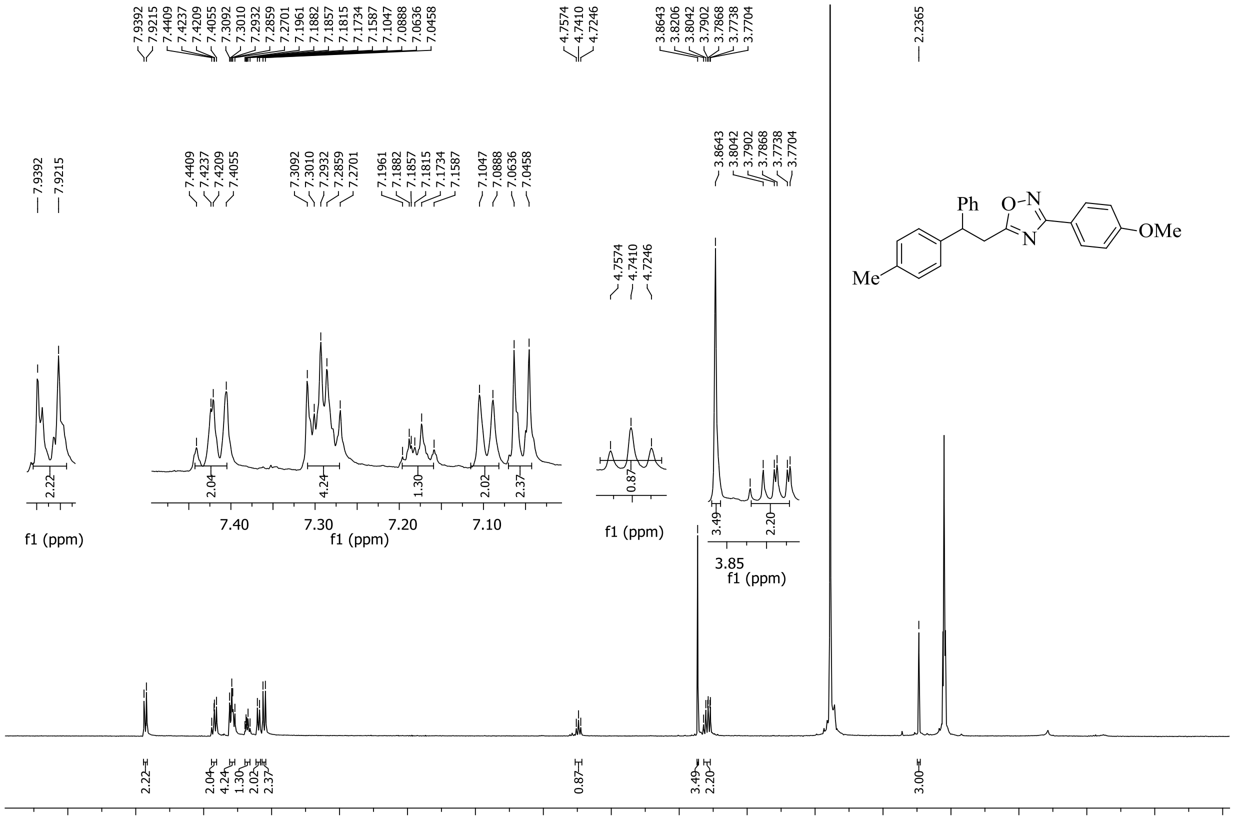

Fig. S46. ^{1}H NMR spectrum of compound **2k** [500 MHz, $(CD_3)_2CO$].

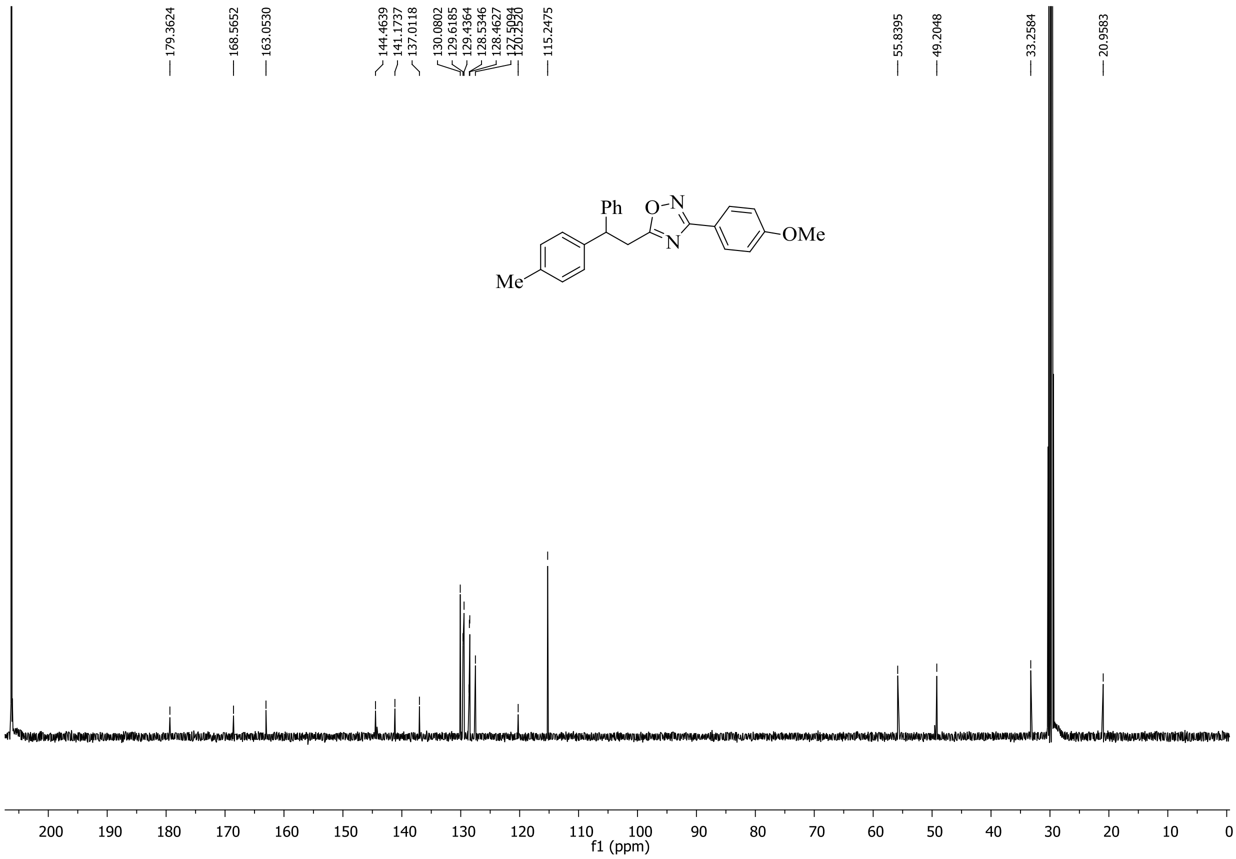

Fig. S47. ^{13}C NMR spectrum of compound **2k** [125 MHz, $(CD_3)_2CO$].

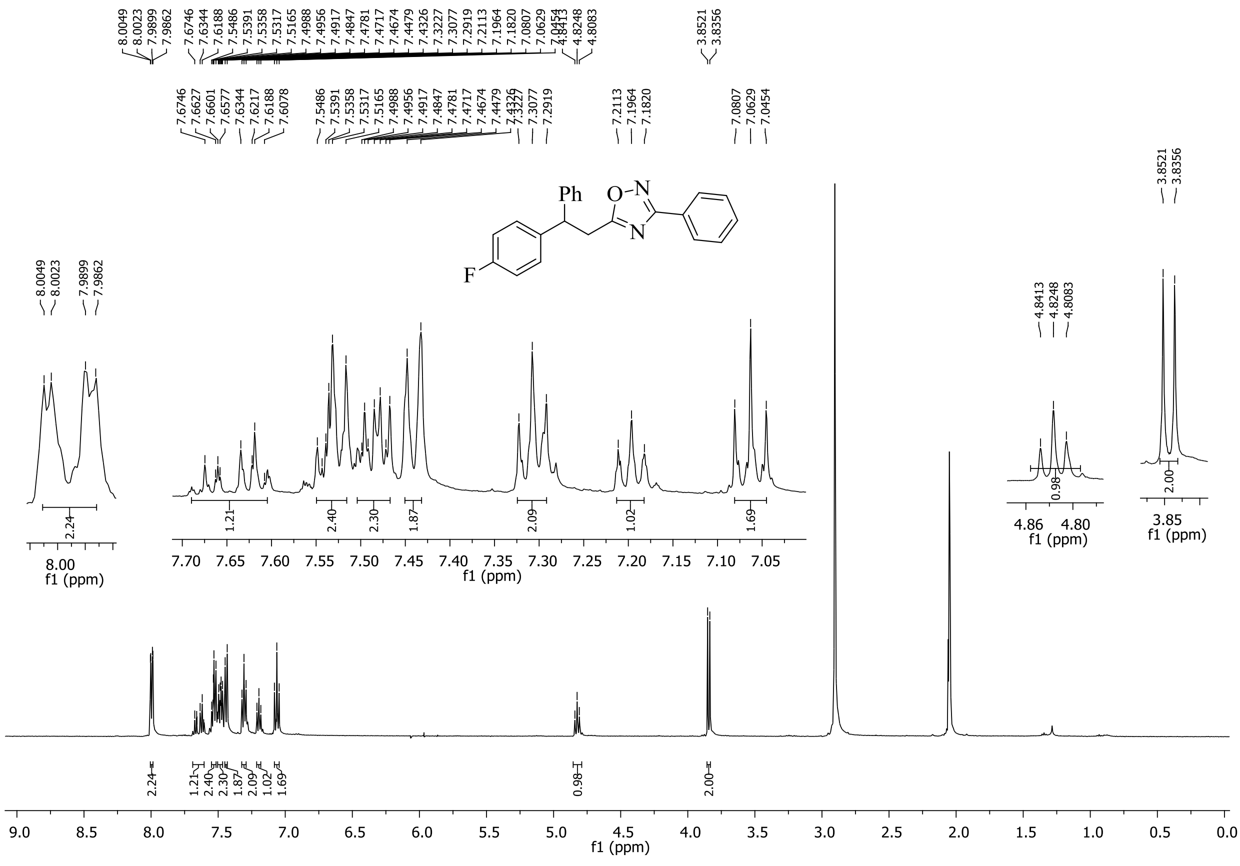

Fig. S48. ^{1}H NMR spectrum of compound **2l** [500 MHz, $(CD_3)_2CO$].

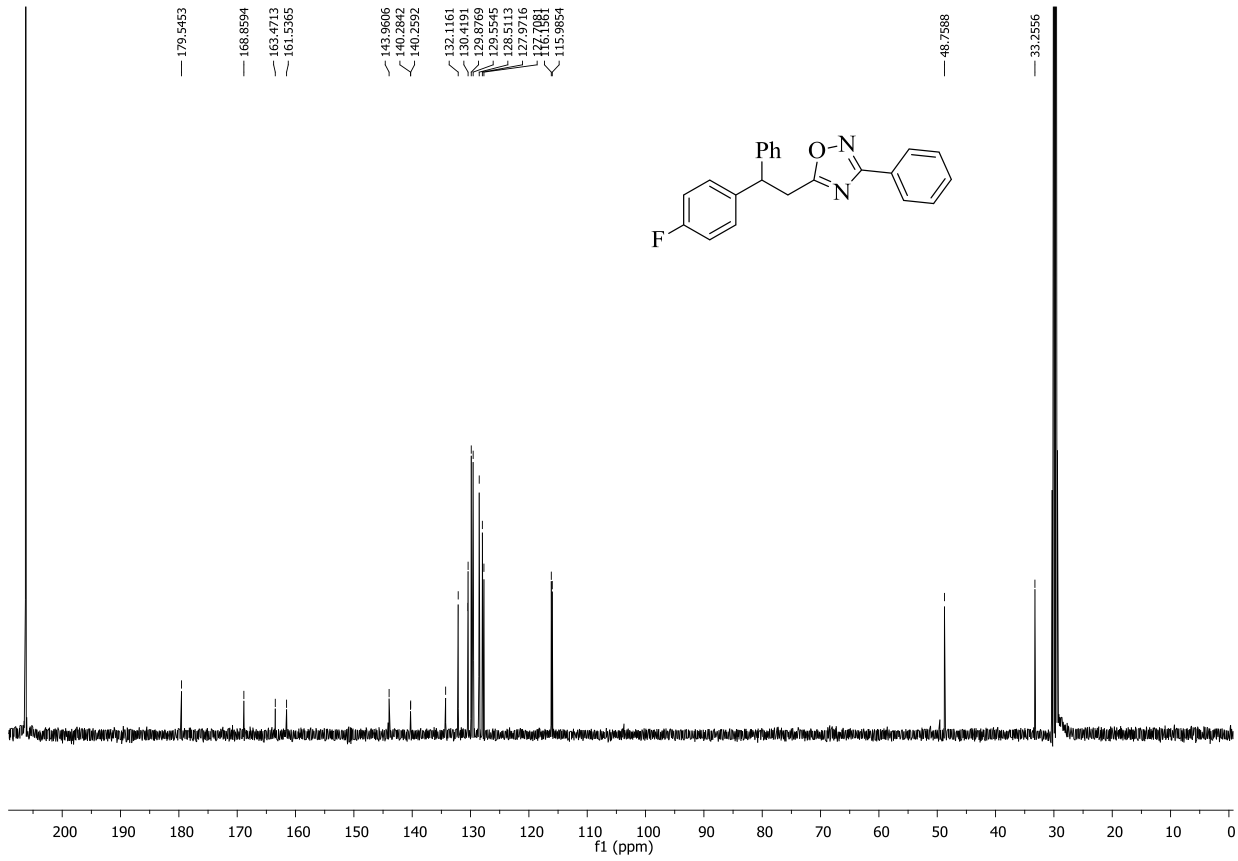

Fig. S49. ^{13}C NMR spectrum of compound **2l** [125 MHz, $(CD_3)_2CO$].

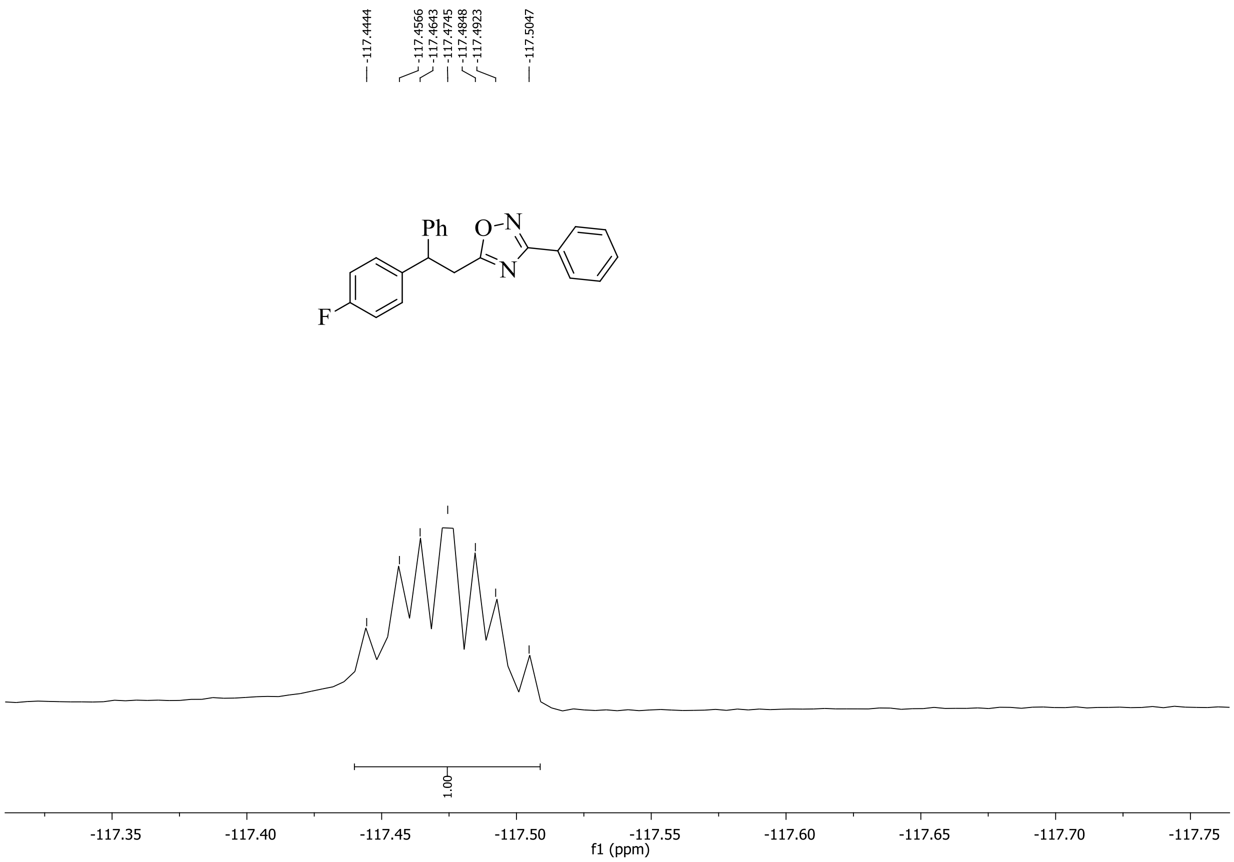

Fig. S50. ^{19}F NMR spectrum of compound **2l** [470 MHz, $(CD_3)_2CO$].

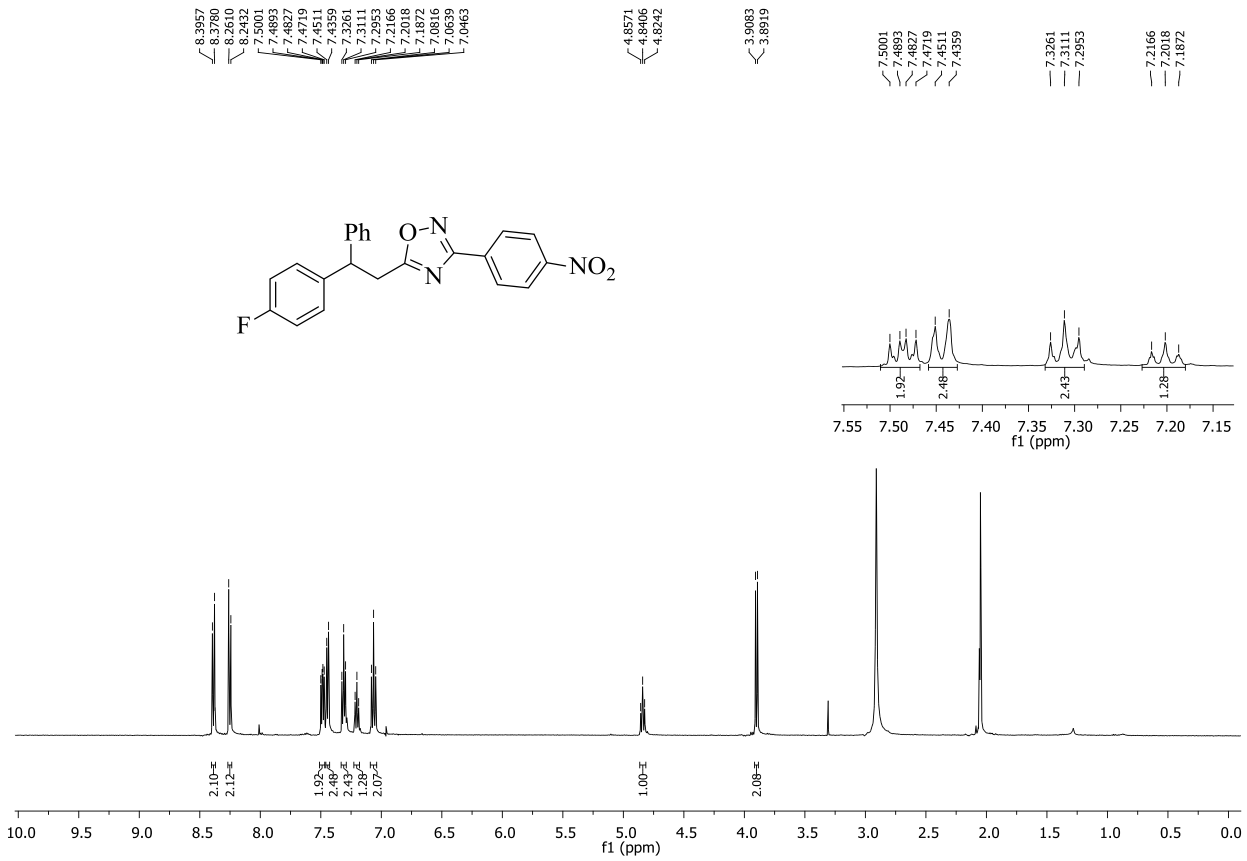

Fig. S51. ^{1}H NMR spectrum of compound **2m** [500 MHz, $(CD_3)_2CO$].

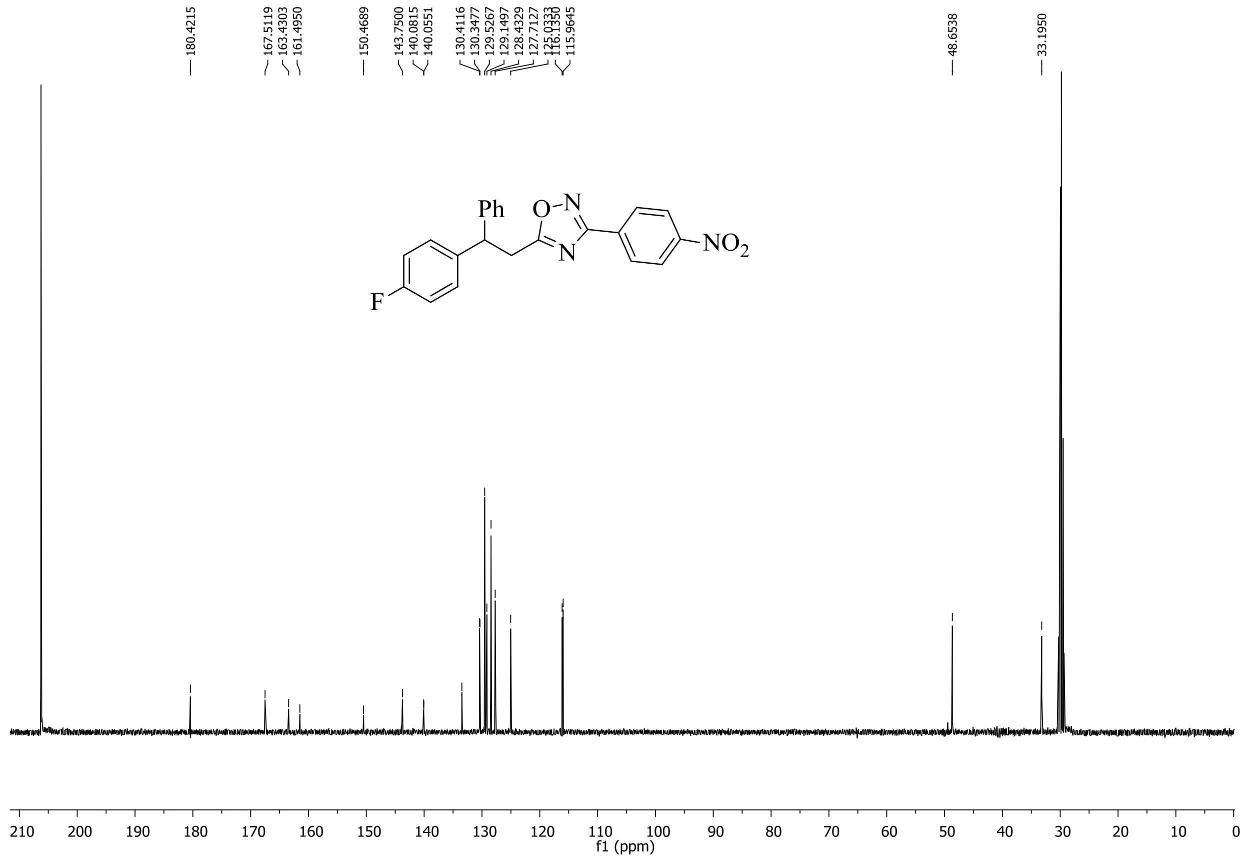

Fig. S52. ^{13}C NMR spectrum of compound **2m** [125 MHz, $(CD_3)_2CO$].

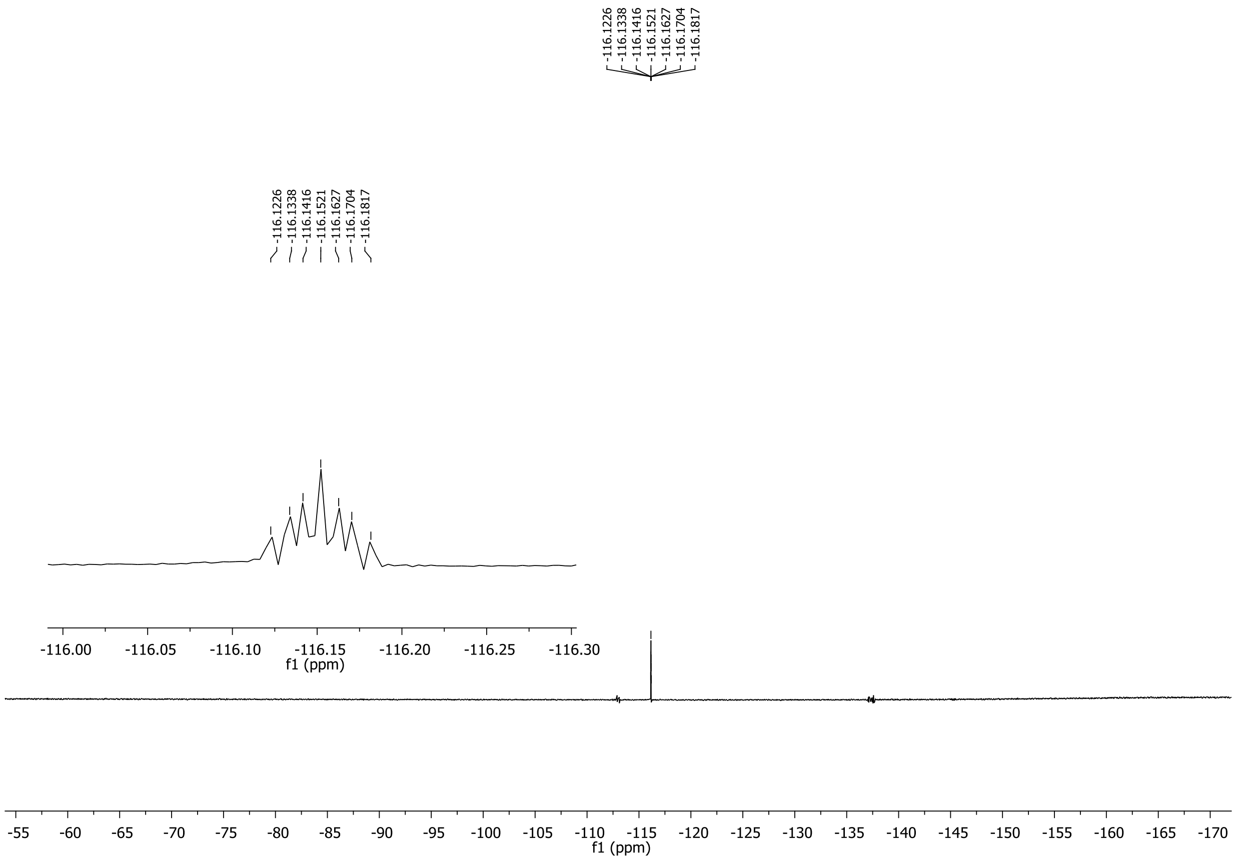

Fig. S53.^{19}F NMR spectrum of compound **2m** [470 MHz, $(CD_3)_2CO$].

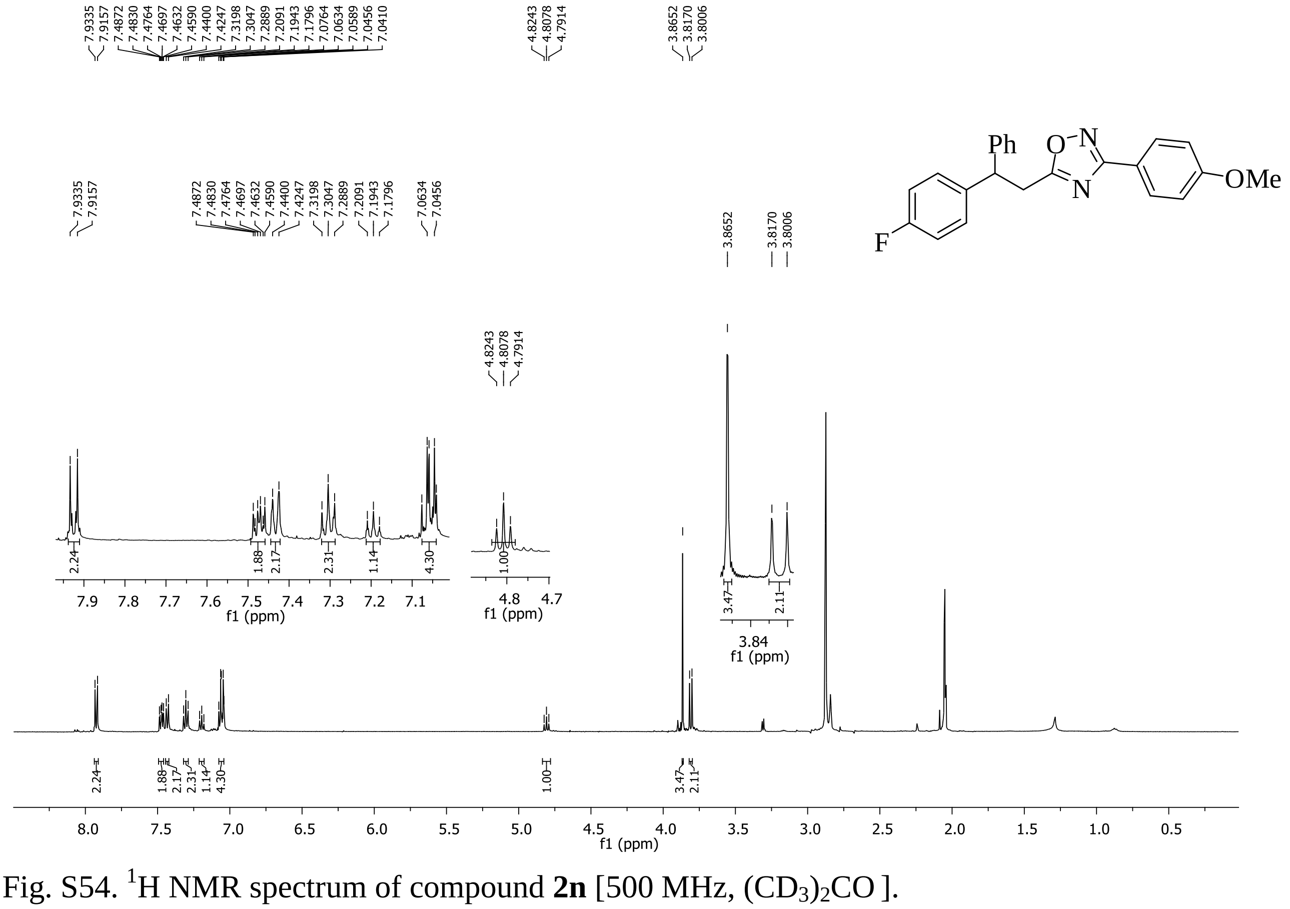

Fig. S54. ^{1}H NMR spectrum of compound **2n** [500 MHz, $(CD_3)_2CO$].

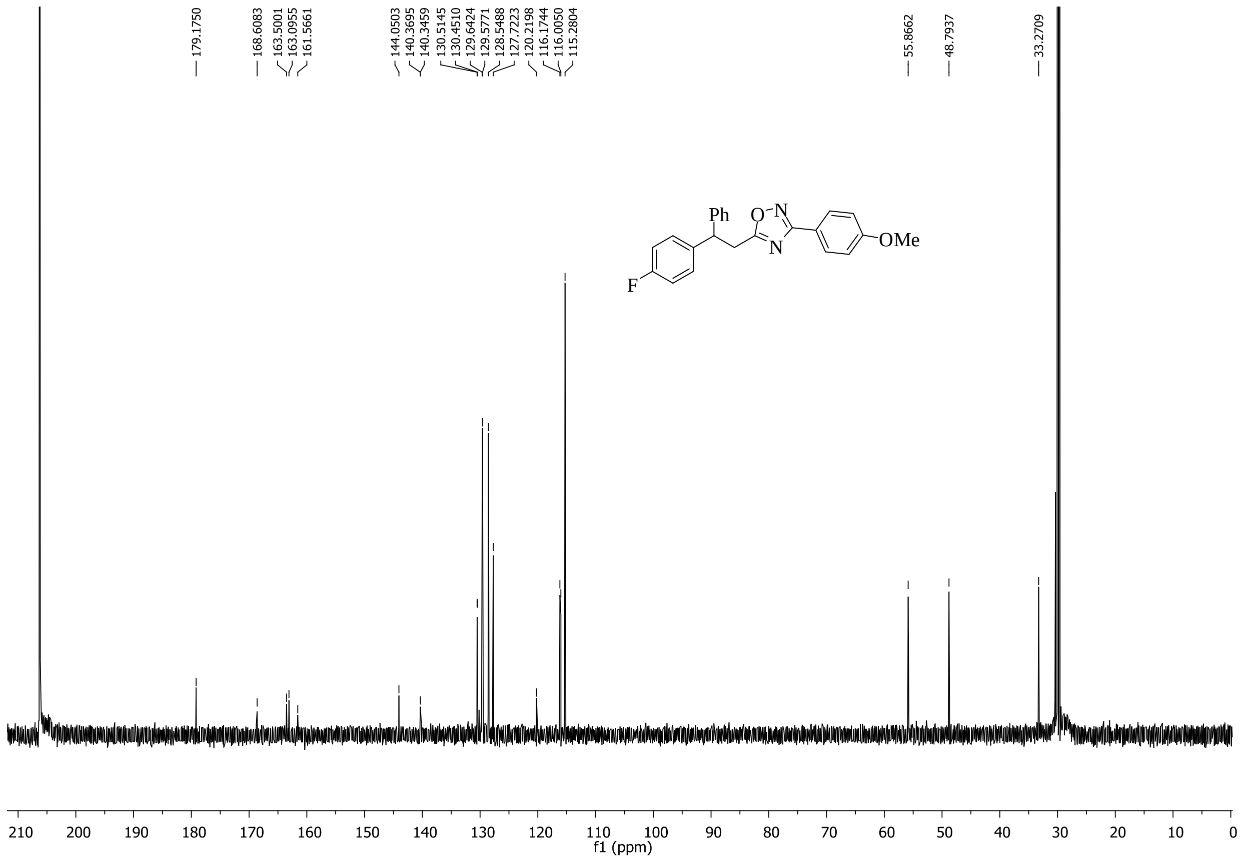

Fig. S55. ^{13}C NMR spectrum of compound **2n** [125 MHz, $(CD_3)_2CO$].

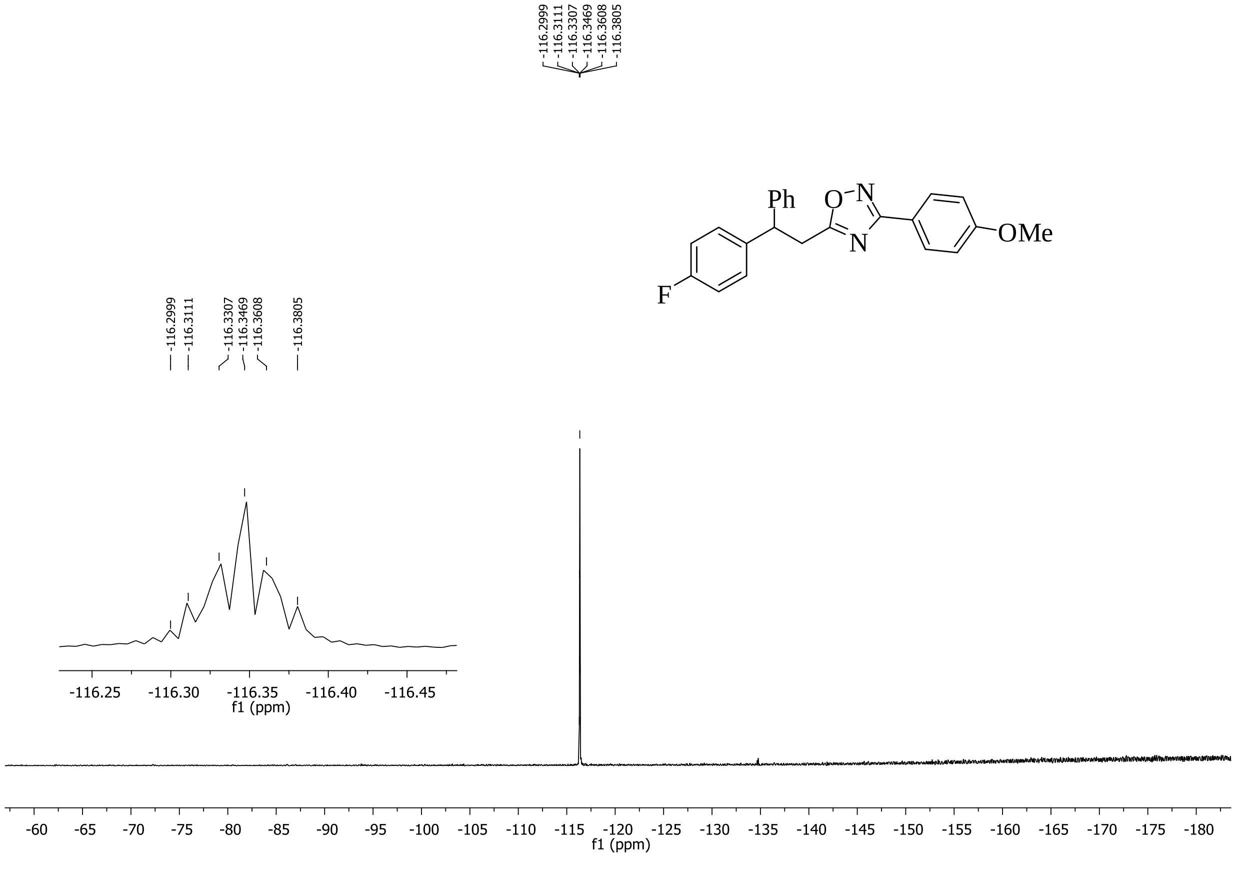

Fig. S56. ^{19}F NMR spectrum of compound **2n** [470 MHz, $(CD_3)_2CO$].

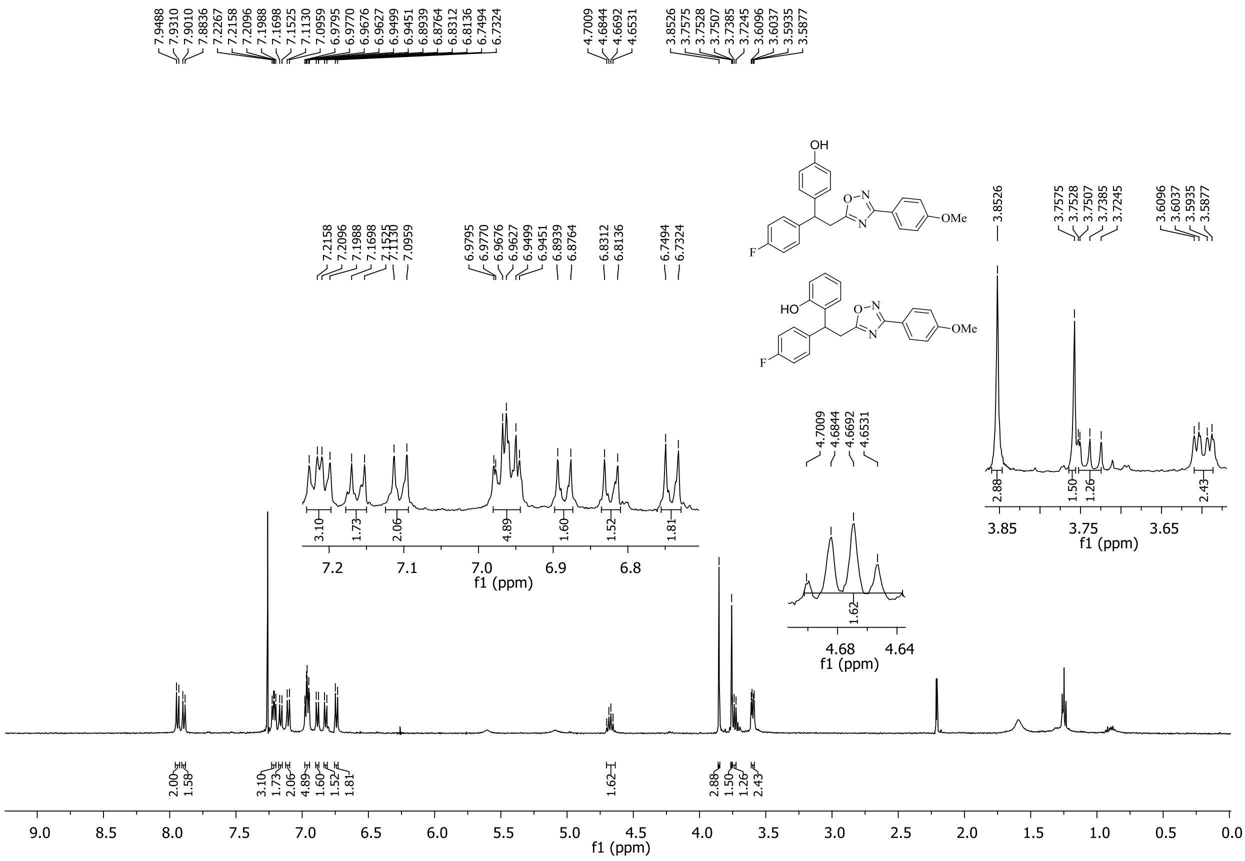

Fig. S57. ^{1}H NMR spectrum of compounds **2o, 2p** [500 MHz, $(CD_3)_2CO$].

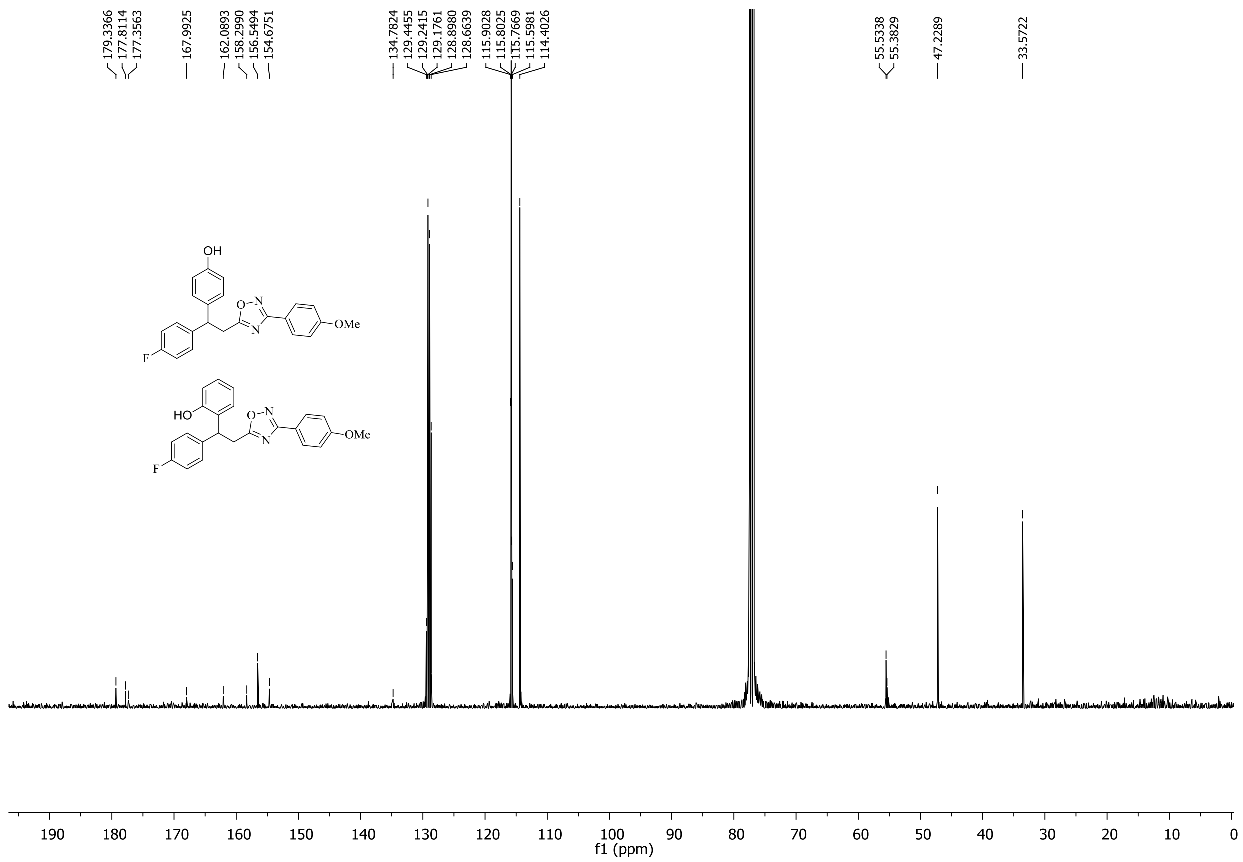

Fig. S58. ^{13}C NMR spectrum of compounds **2o, 2p** [125 MHz, $(CD_3)_2CO$].

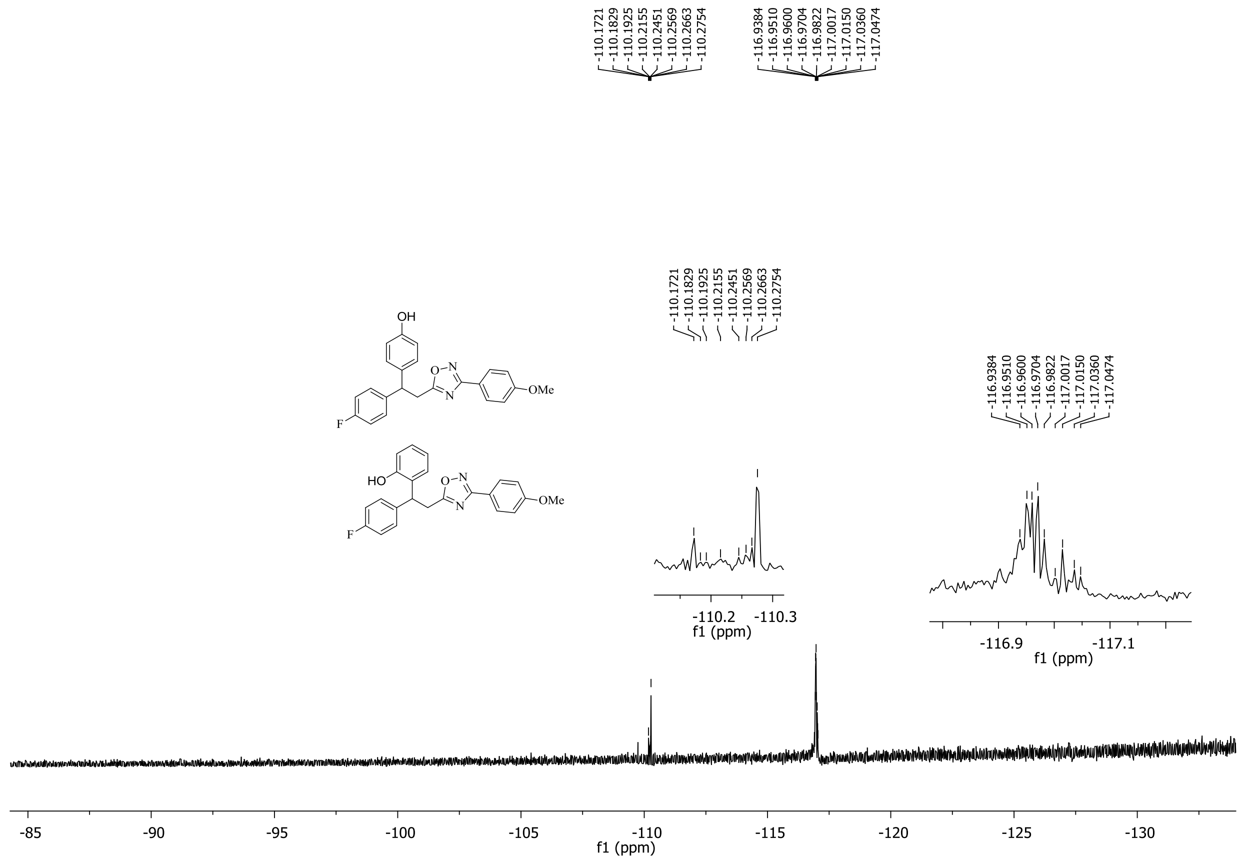

Fig. S59. ^{19}F NMR spectrum of compounds **2o,2p** [470 MHz, $(CD_3)_2CO$].

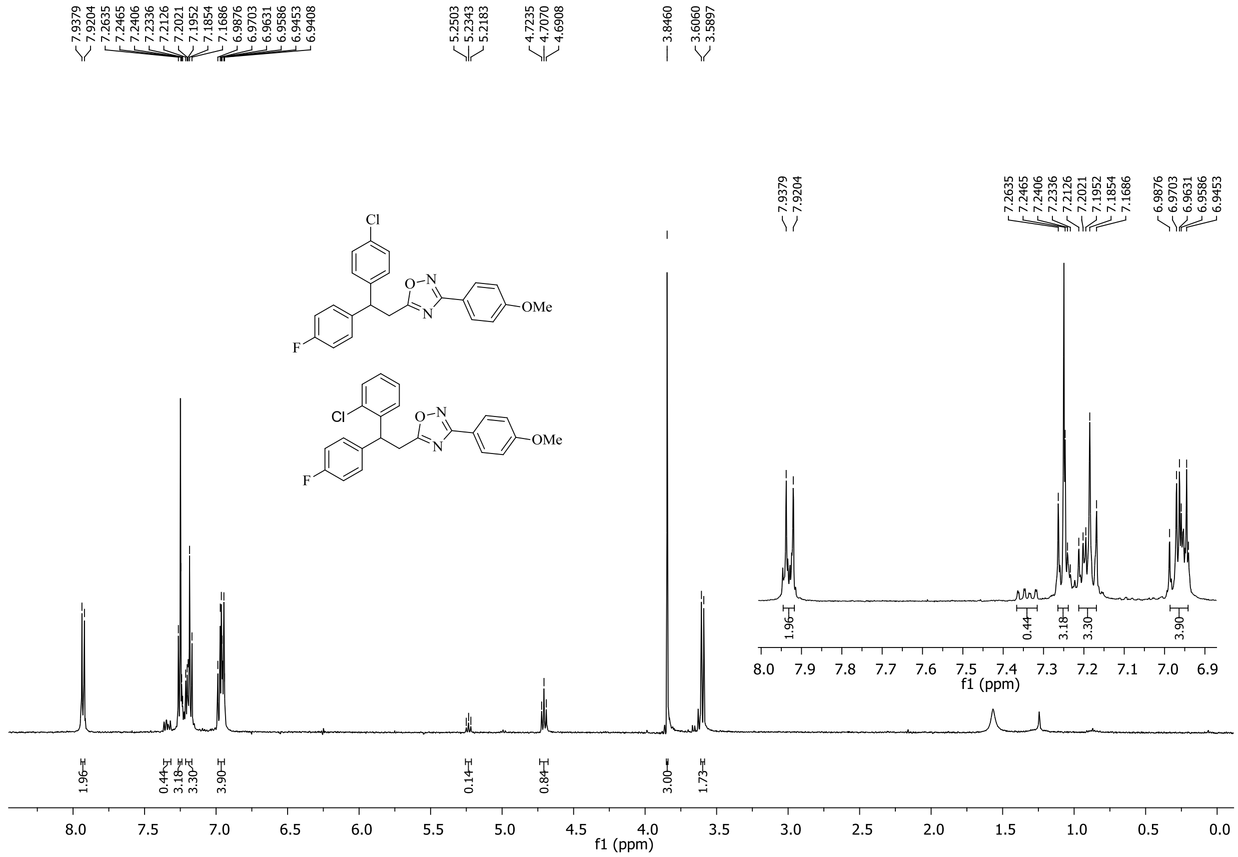

Fig. S60. ^{1}H NMR spectrum of compounds **2q, 2r** [500 MHz, $(CD_3)_2CO$].

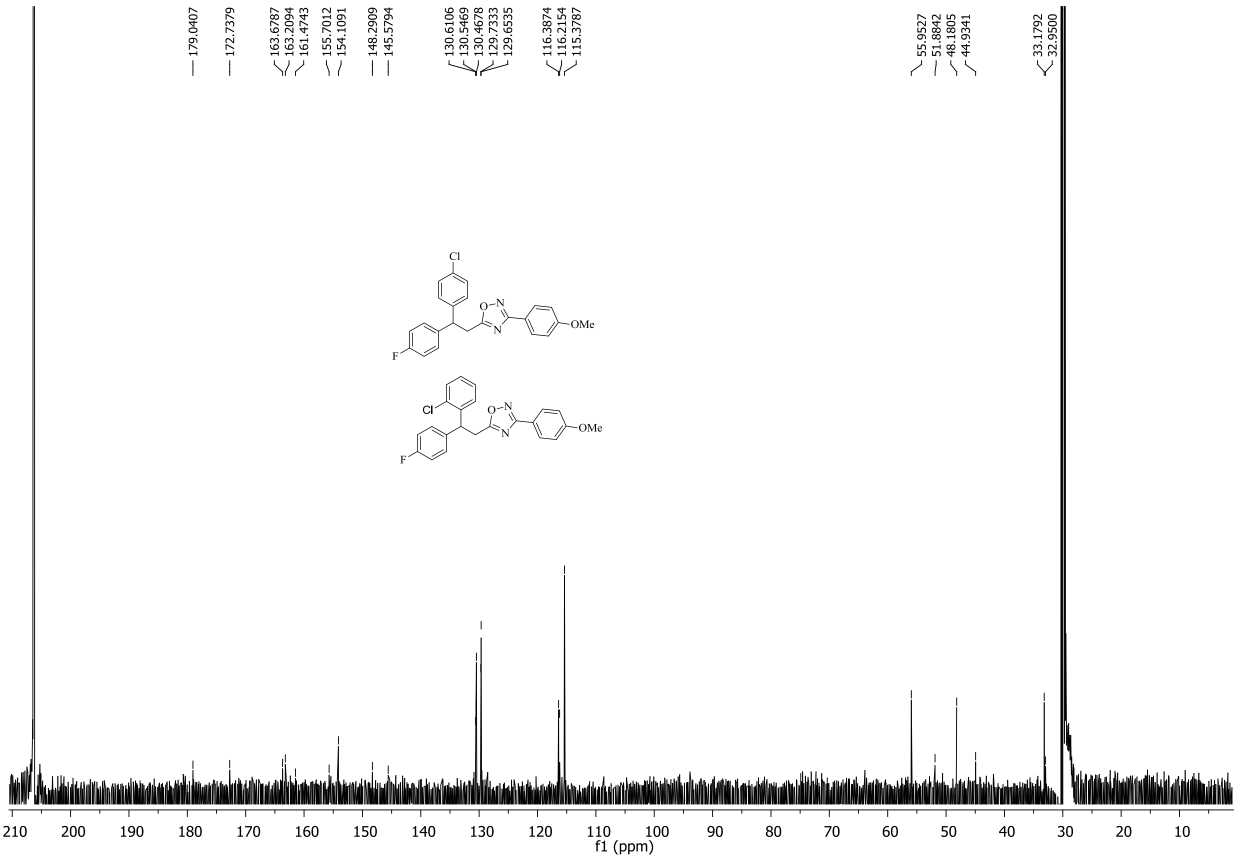

Fig. S61. ^{13}C NMR spectrum of compounds **2q, 2r** [125 MHz, $(CD_3)_2CO$].

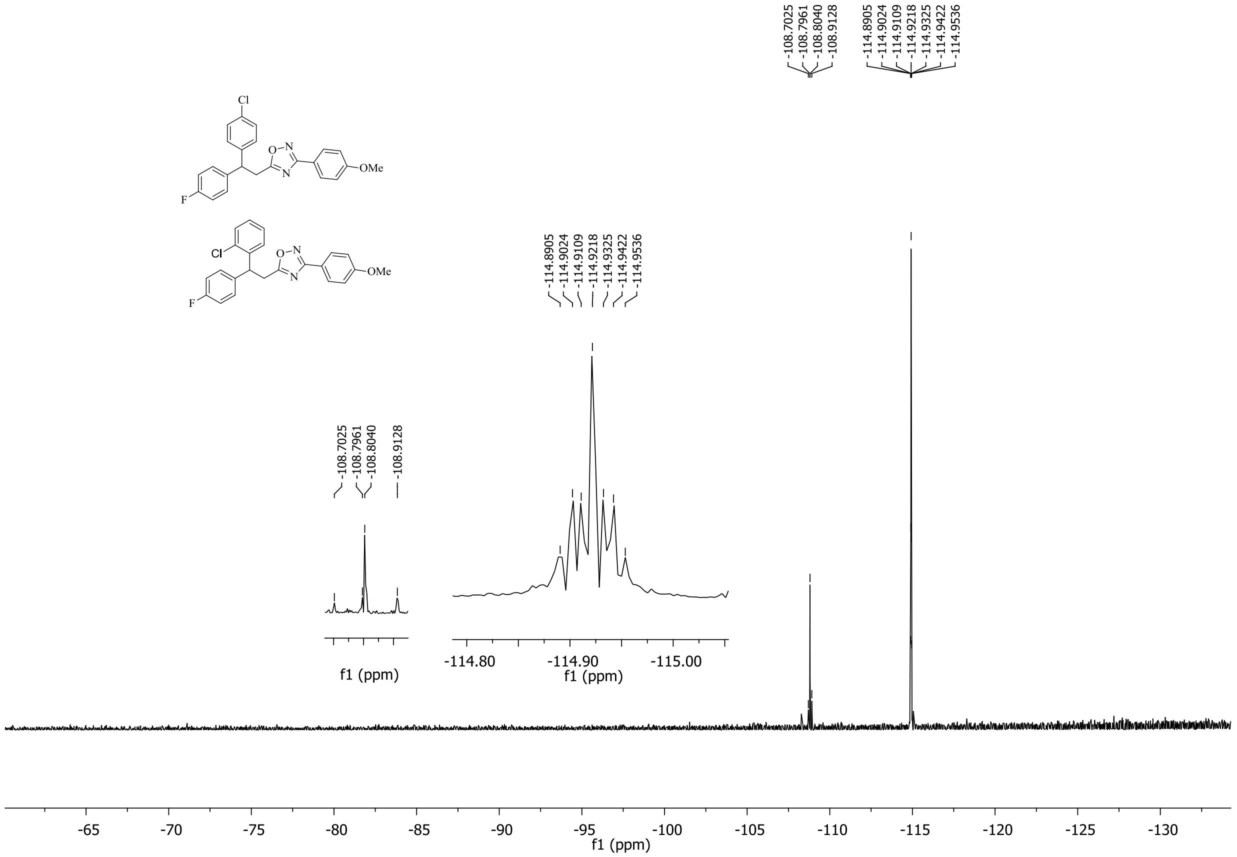

Fig. S62. ^{19}F NMR spectrum of compounds **2q,2r** [470 MHz, $(CD_3)_2CO$].

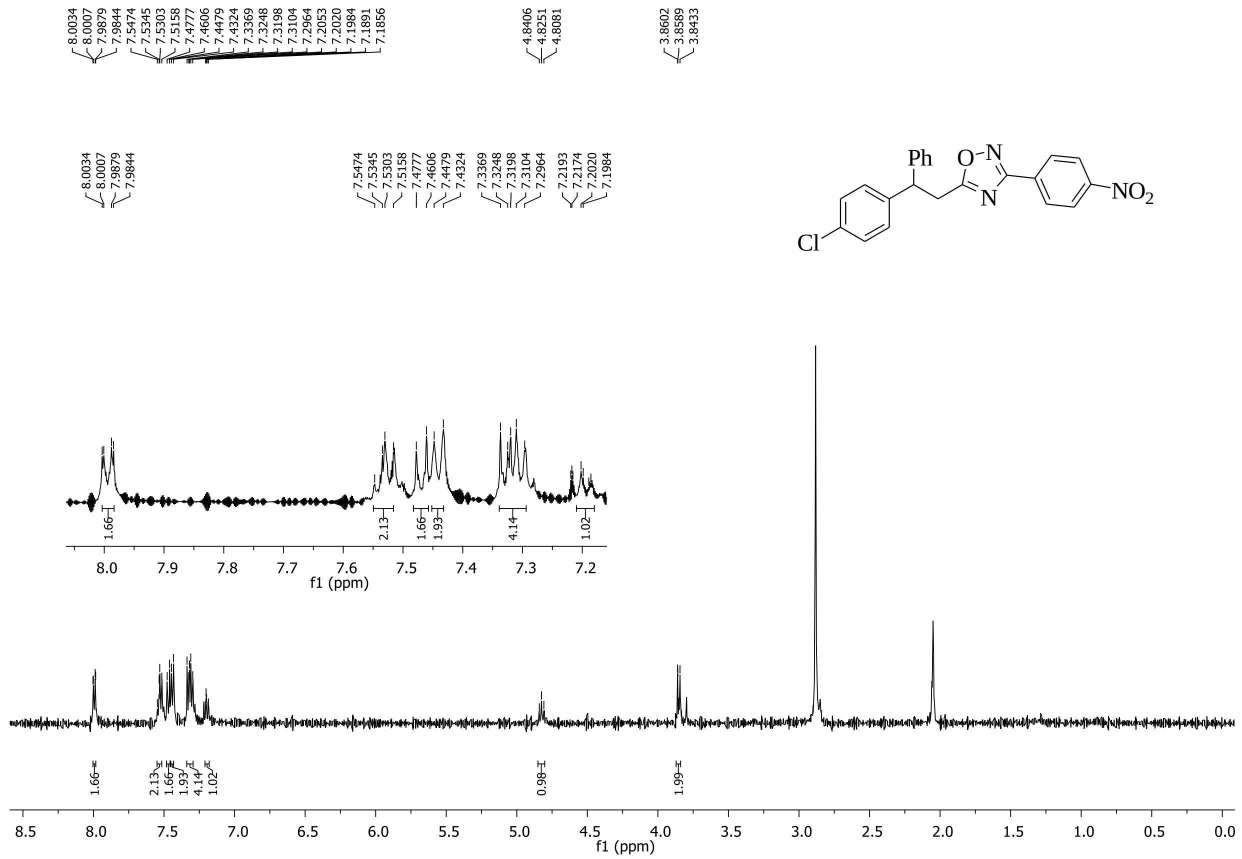

Fig. S63. ^{1}H NMR spectrum of compound **2t** [500 MHz, $(CD_3)_2CO$].

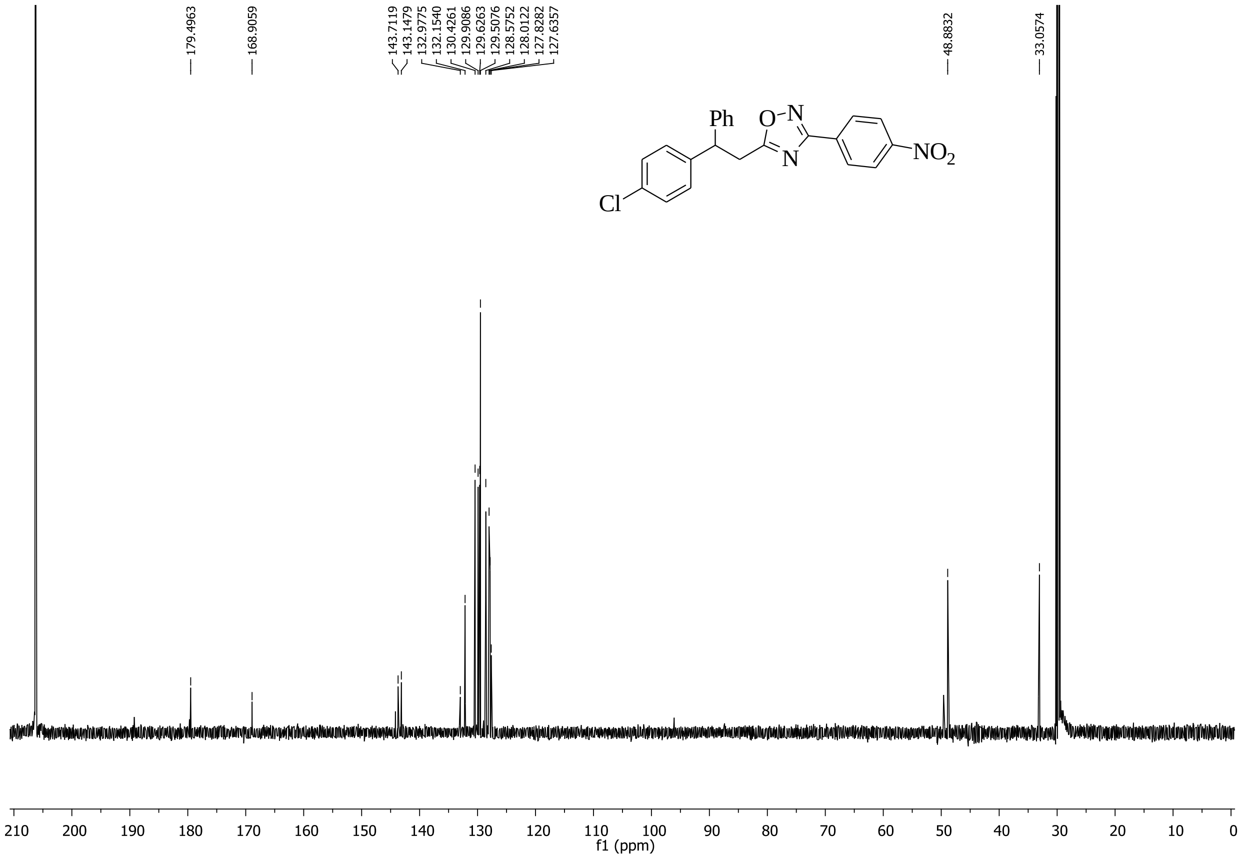

Fig. S64. ^{13}C NMR spectrum of compound **2t** [125 MHz, $(CD_3)_2CO$].

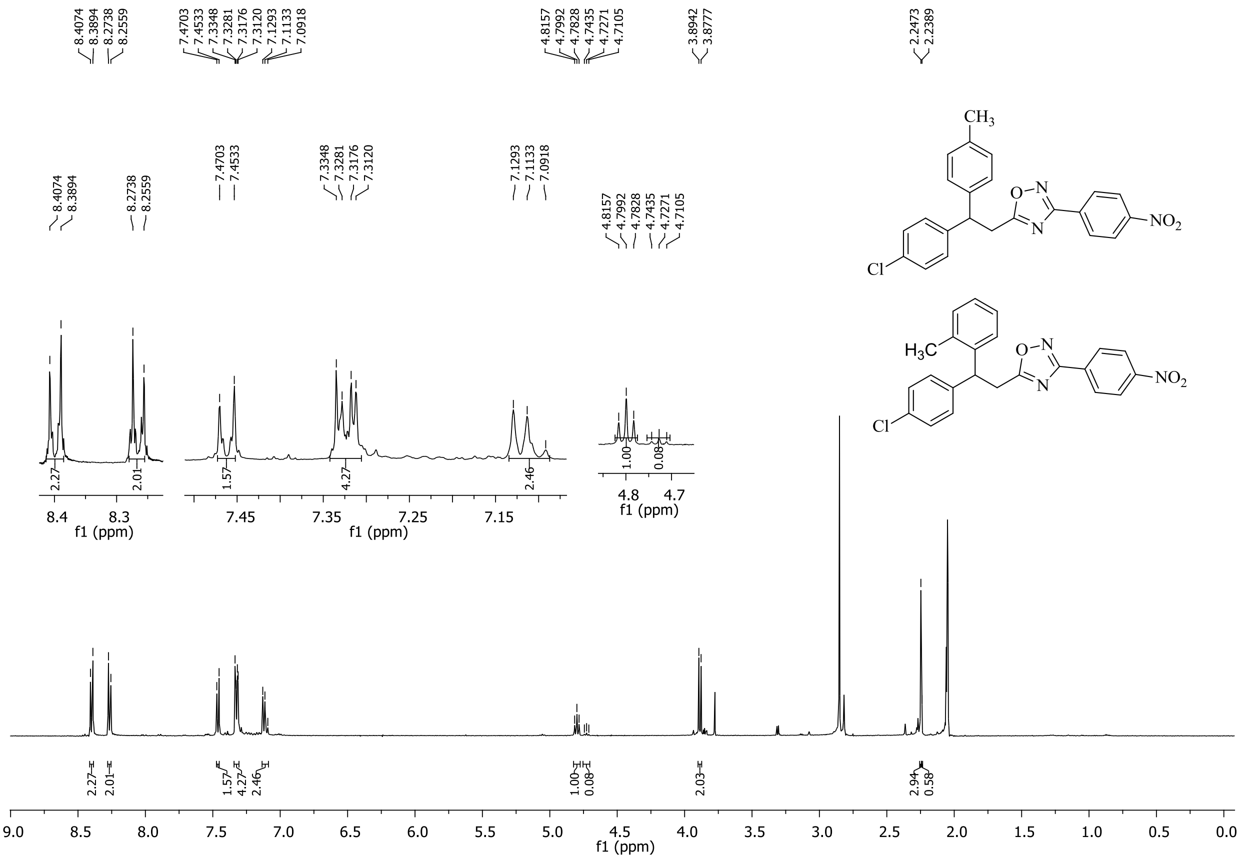

Fig. S65. ^{1}H NMR spectrum of compounds **2u, 2v** [500 MHz, $(CD_3)_2CO$].

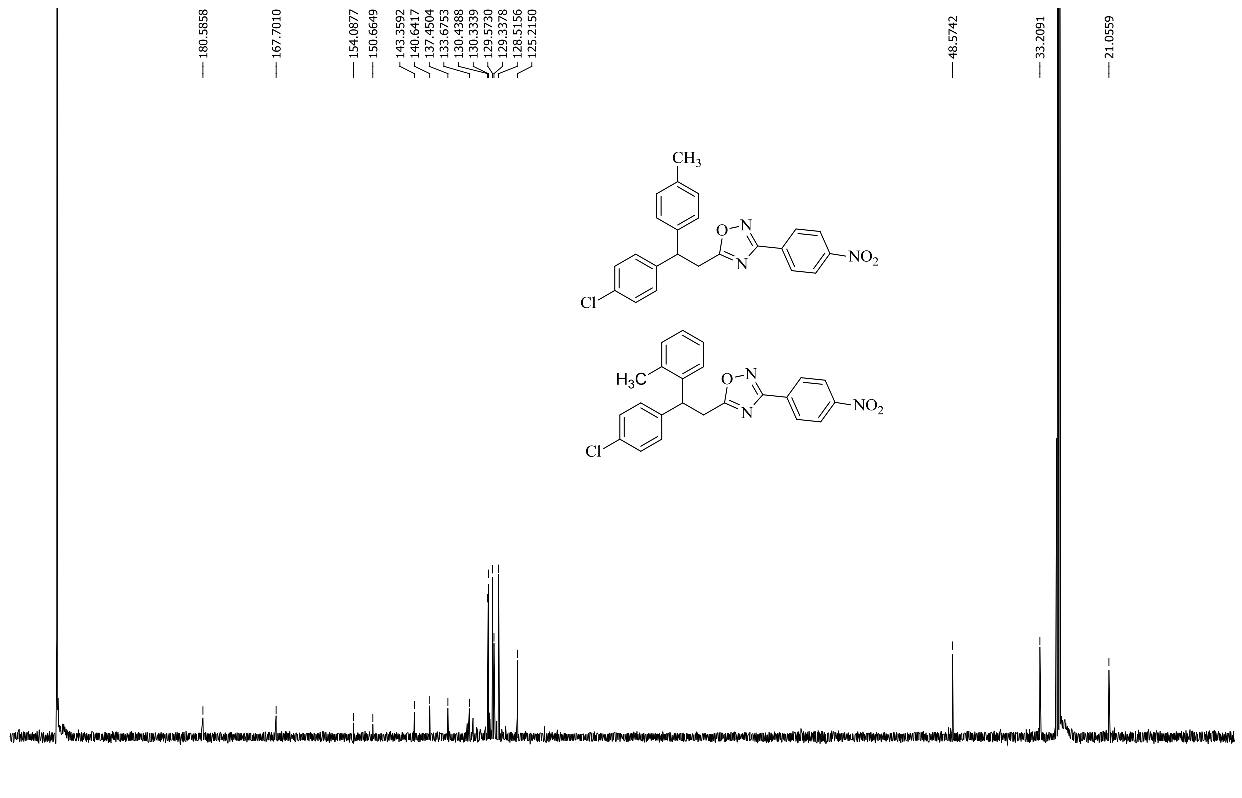

Fig. S66. ^{13}C NMR spectrum of compounds **2u, 2v** [125 MHz, $(CD_3)_2CO$].

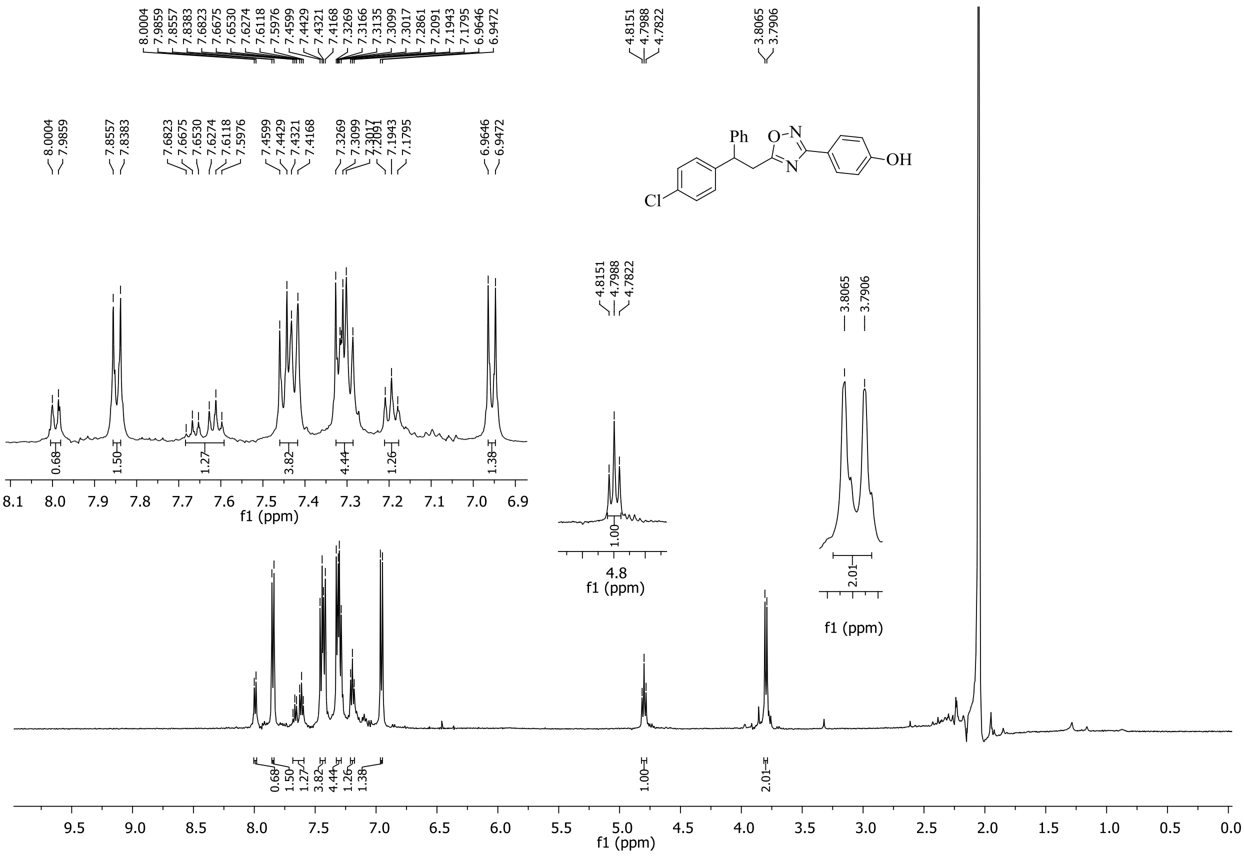

Fig. S67. ^{1}H NMR spectrum of compound **2w** [500 MHz, $(CD_3)_2CO$].

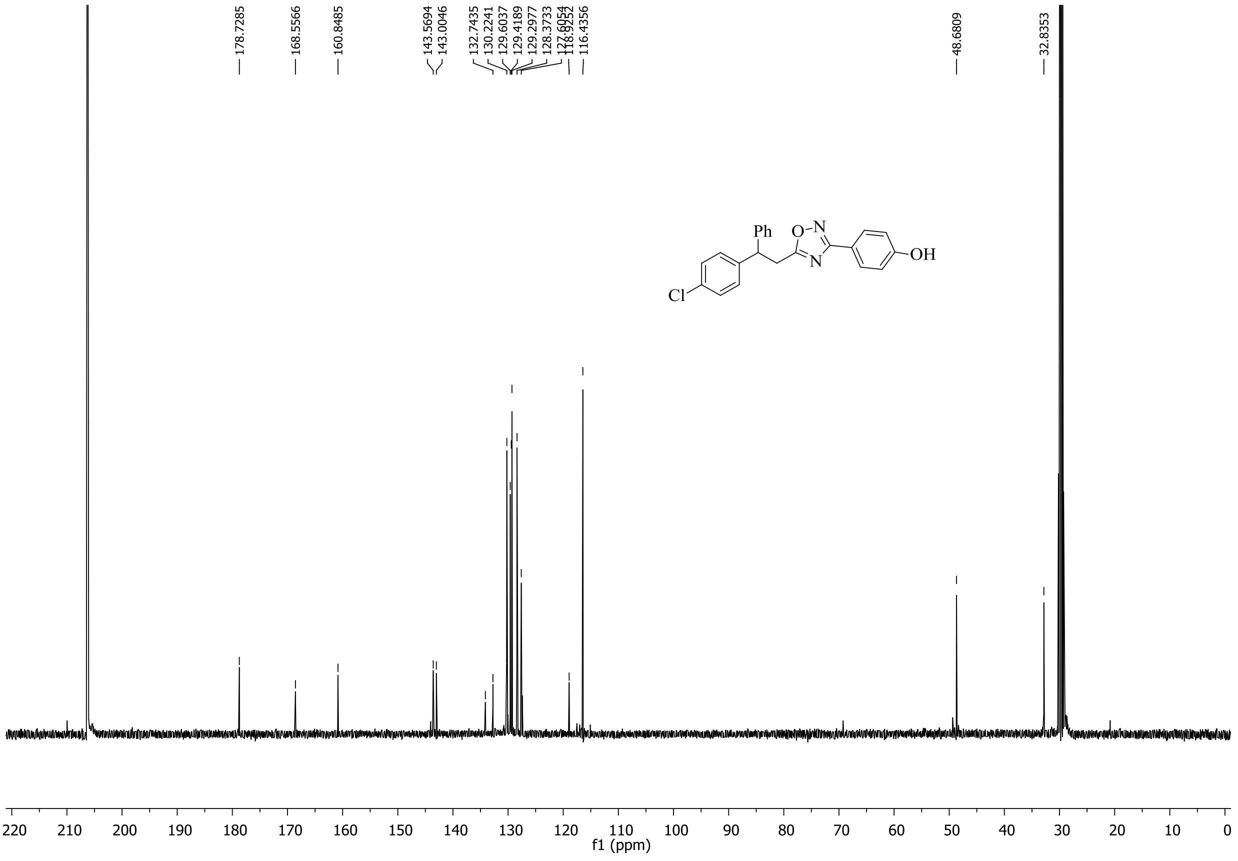

Fig. S68. ^{13}C NMR spectrum of compound **2w** [125 MHz, $(CD_3)_2CO$].

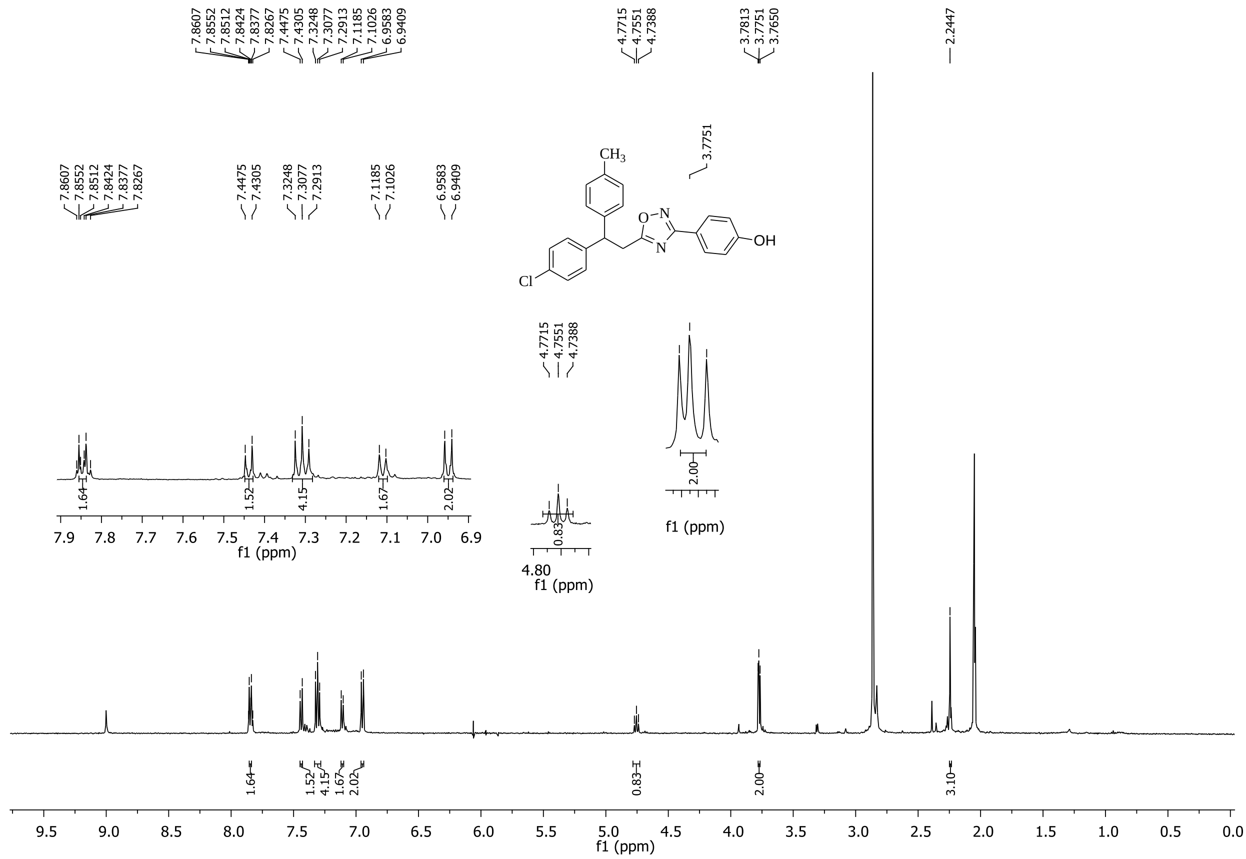

Fig. S69. ^{1}H NMR spectrum of compound **2x** [500 MHz, $(CD_3)_2CO$].

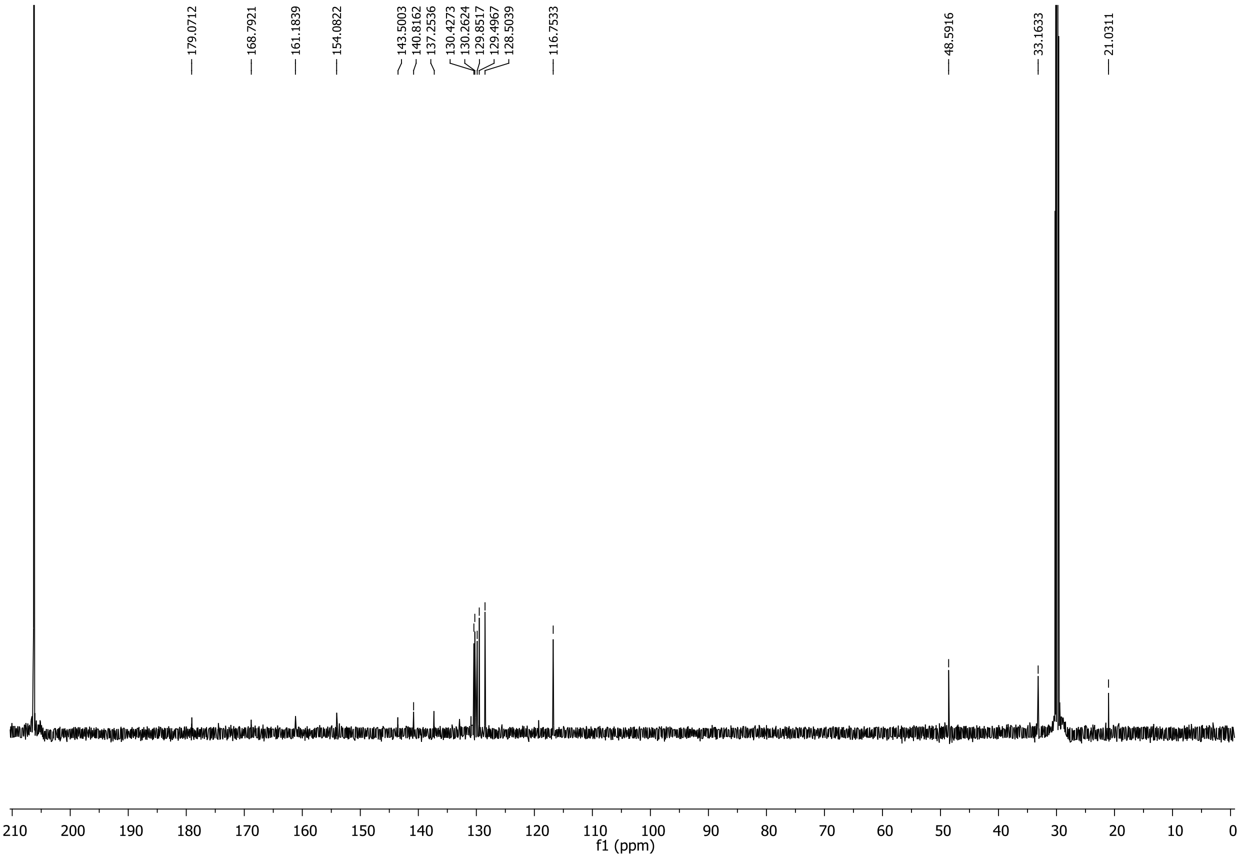

Fig. S70. ^{13}C NMR spectrum of compound **2x** [125 MHz, $(CD_3)_2CO$].

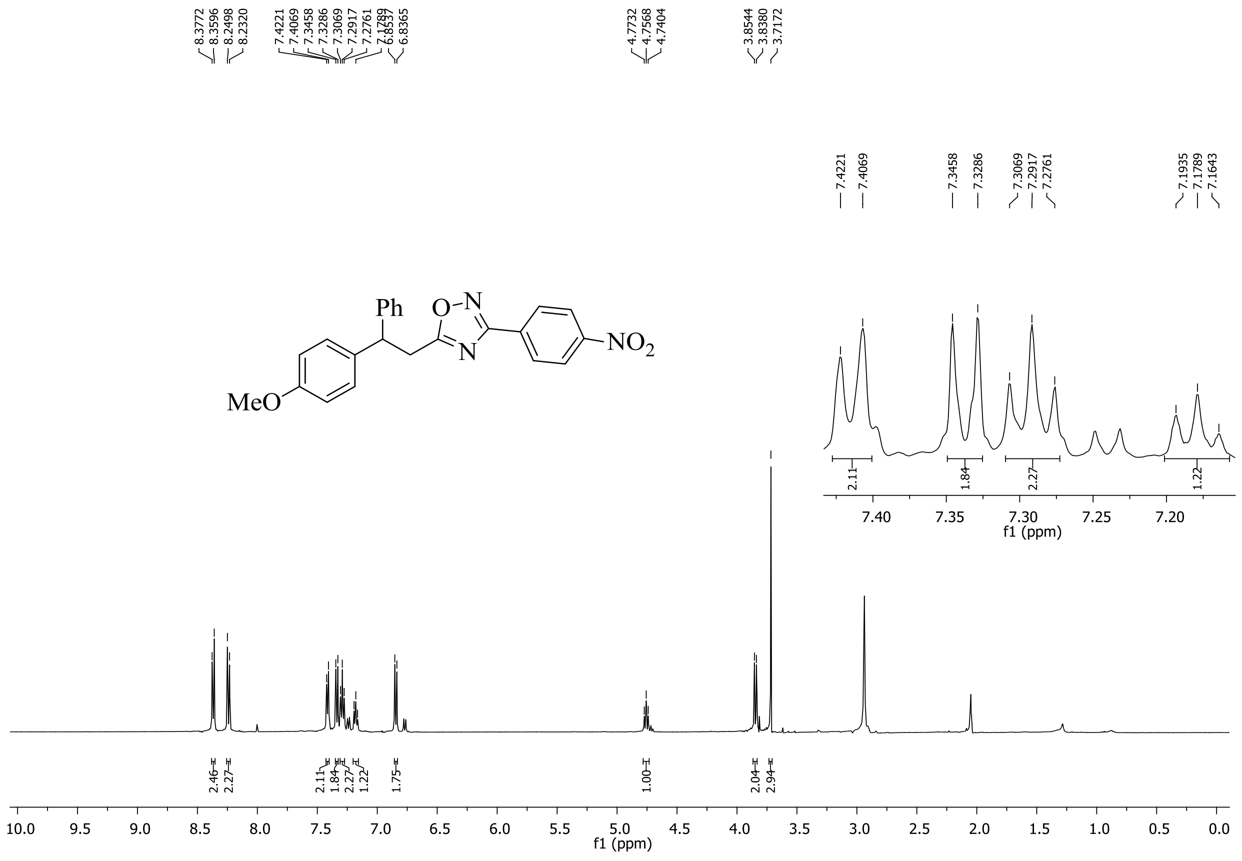

Fig. S71. ^{1}H NMR spectrum of compound **2y** [500 MHz, $(CD_3)_2CO$].

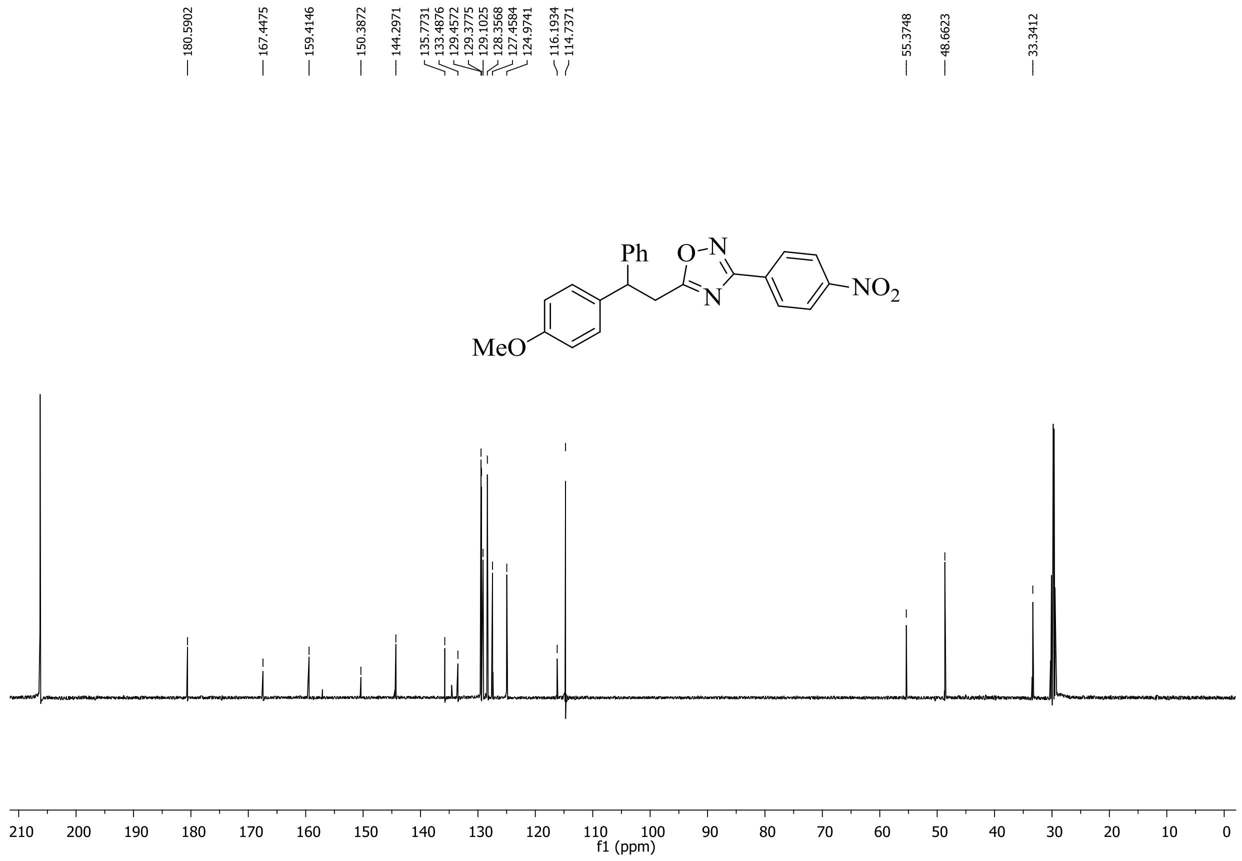

Fig. S72. ^{13}C NMR spectrum of compound **2y** [125 MHz, $(CD_3)_2CO$].

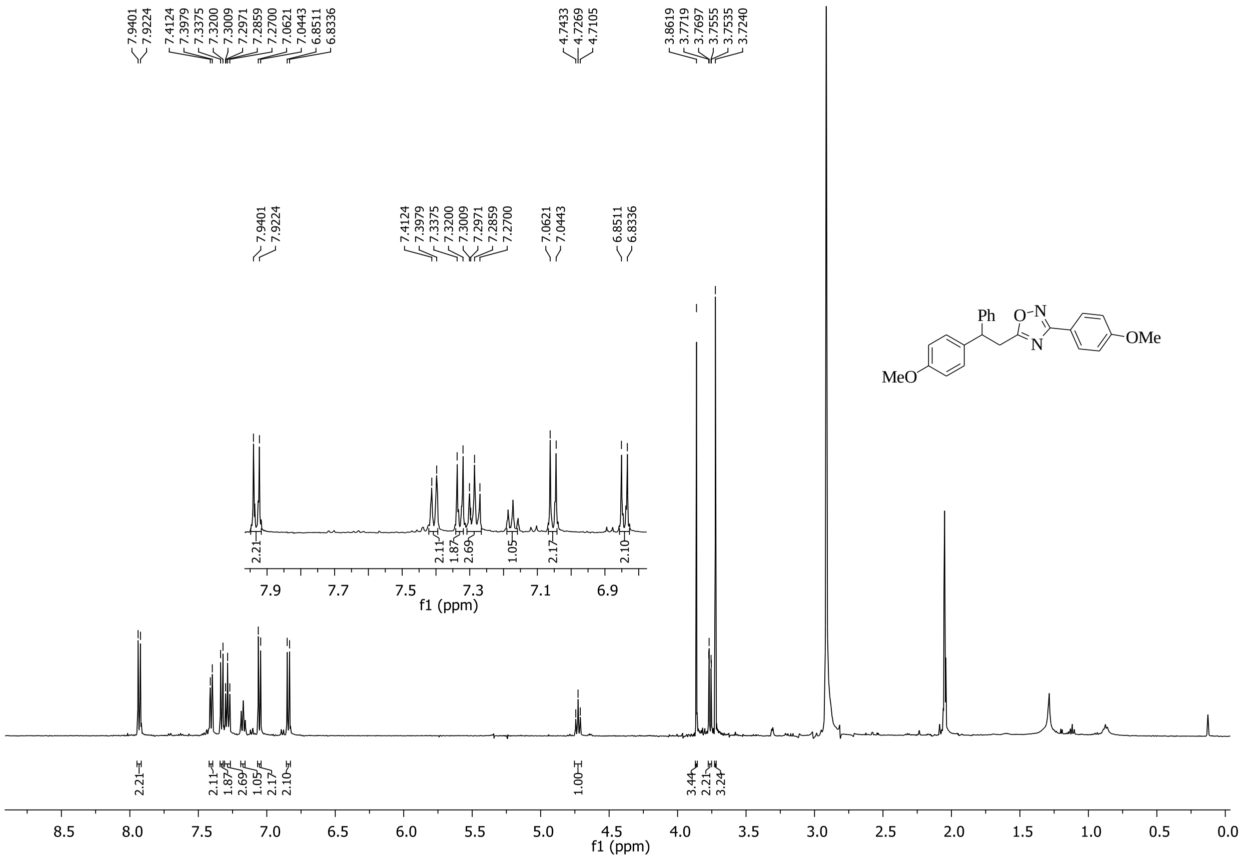

Fig. S73. ^{1}H NMR spectrum of compound **2z** [500 MHz, $(CD_3)_2CO$].

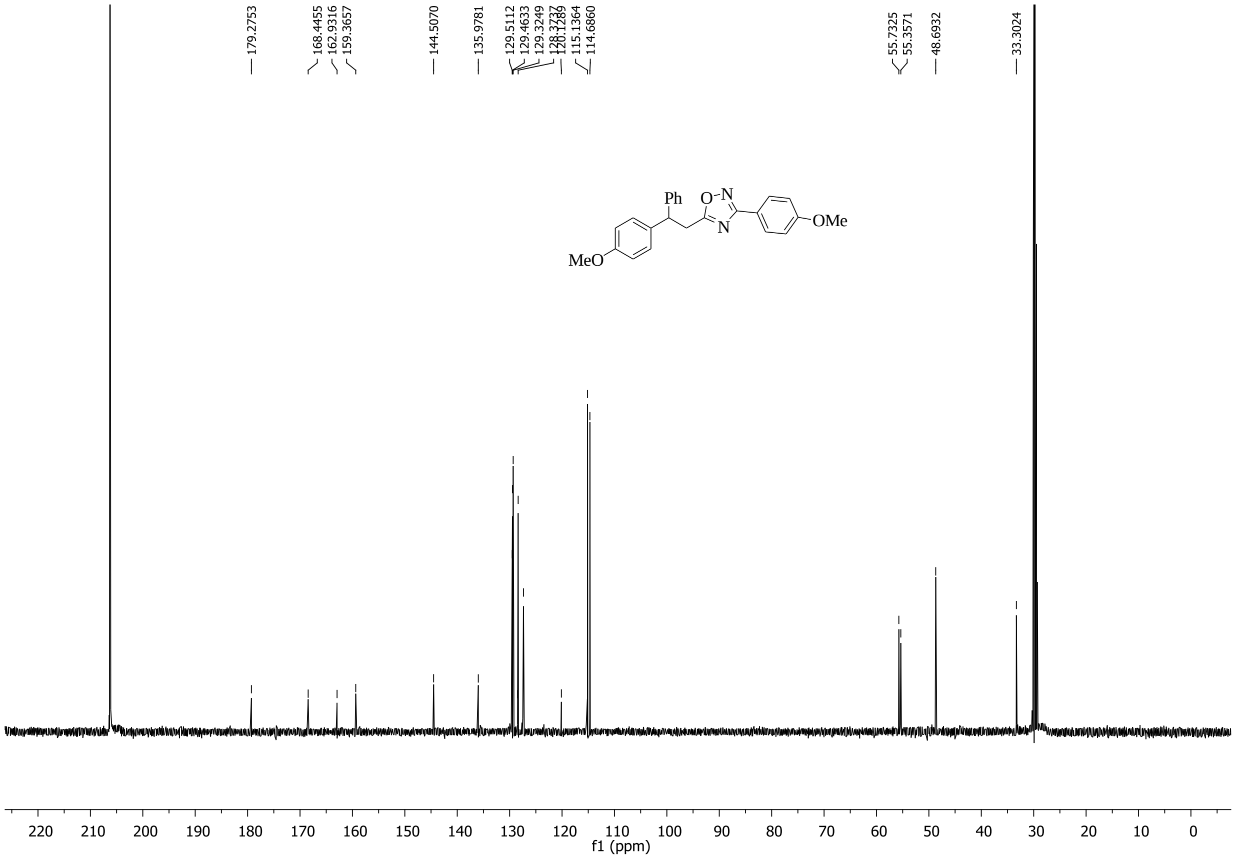

Fig. S74. ^{13}C NMR spectrum of compound **2z** [125 MHz, $(CD_3)_2CO$].

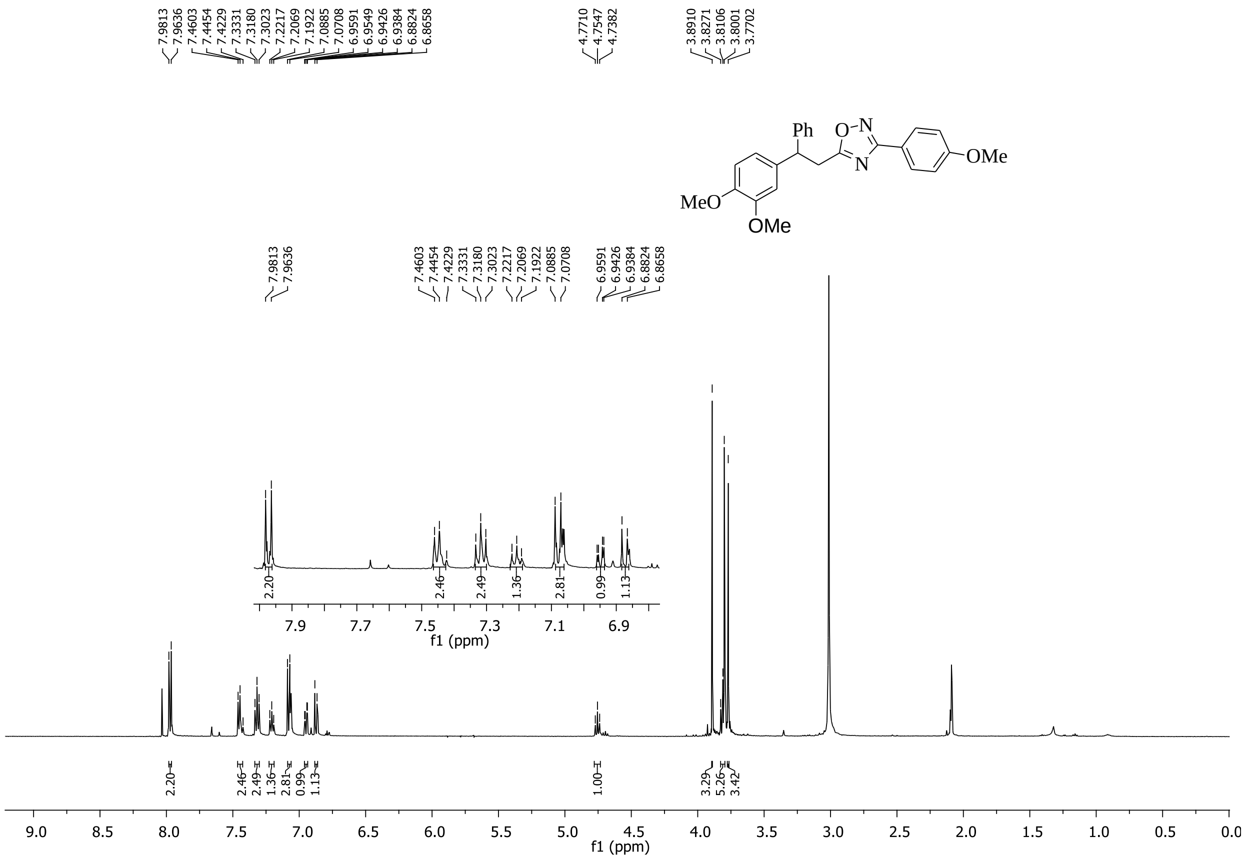

Fig. S75. ^{1}H NMR spectrum of compound **2za** [500 MHz, $(CD_3)_2CO$].

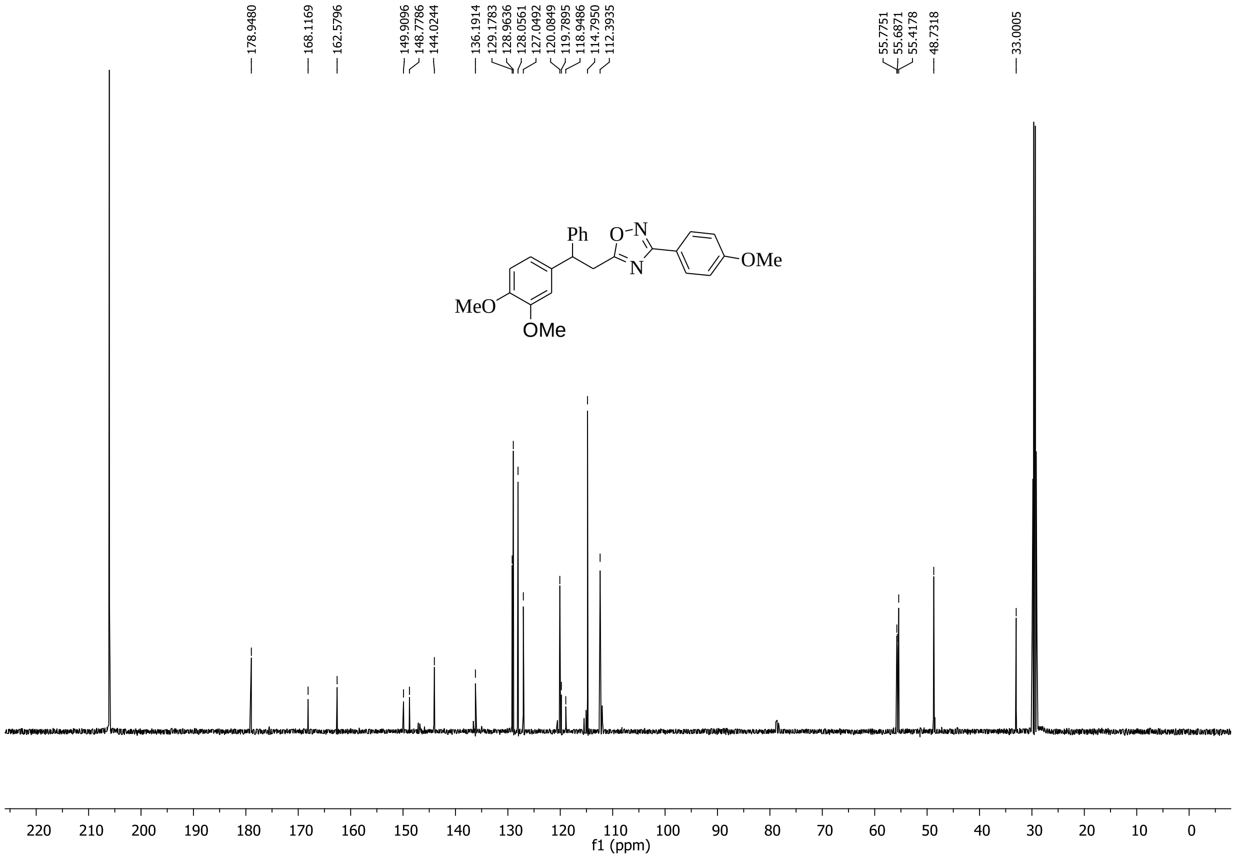

Fig. S76. ^{13}C NMR spectrum of compound **2za** [125 MHz, $(CD_3)_2CO$].

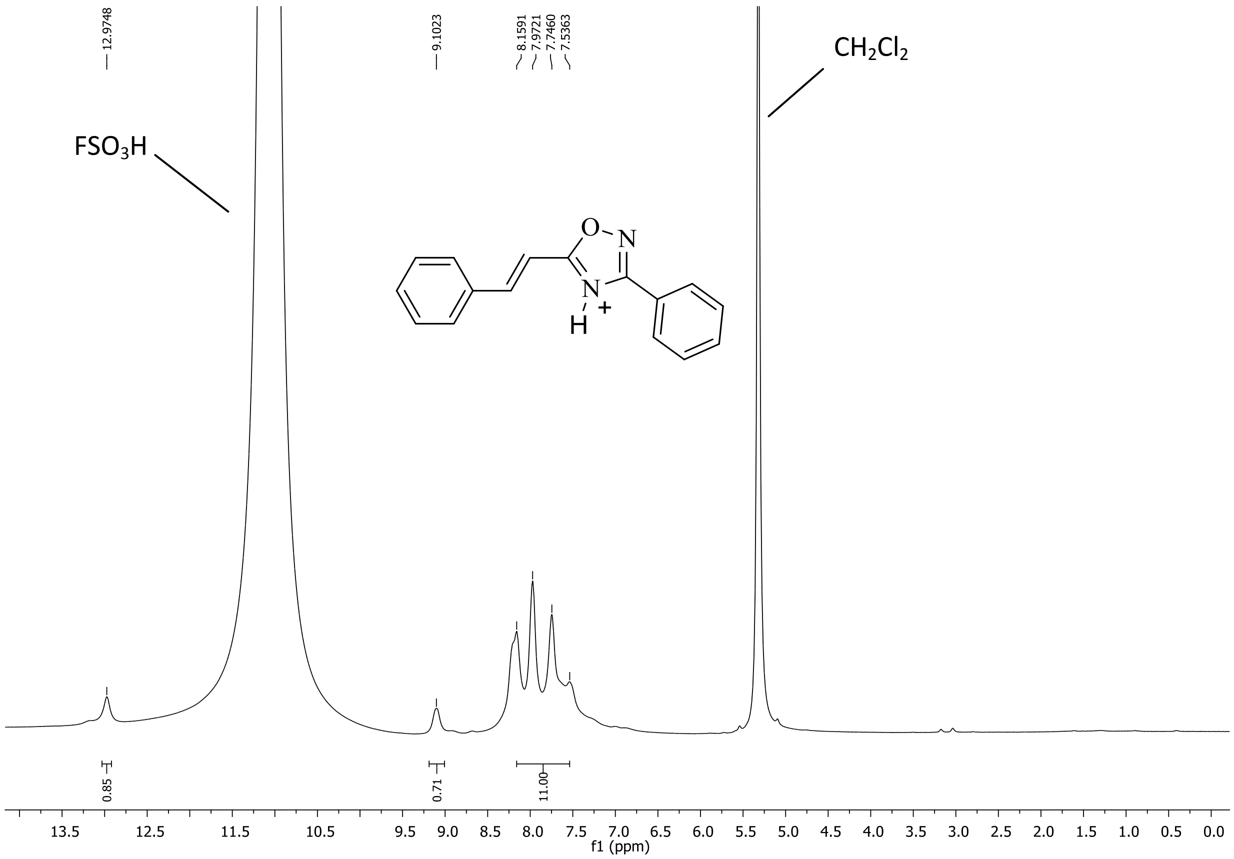

Fig. S77. ^{1}H NMR spectrum of cation **Ca** [400 MHz, FSO_3H, CH_2Cl_2, – 80 °C].

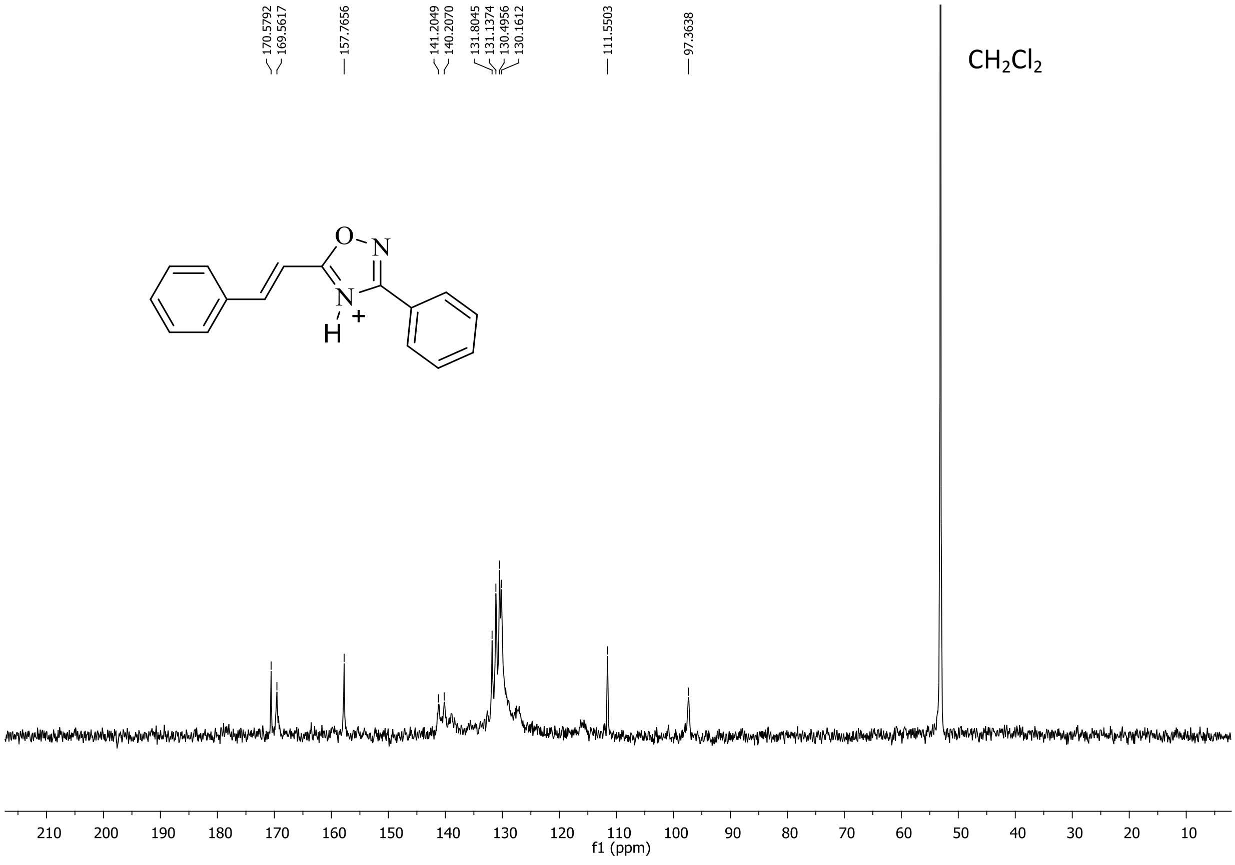

Fig. S78. ^{13}C NMR spectrum of cation **Ca** [125 MHz, FSO_3H, CH_2Cl_2, – 80 °C].

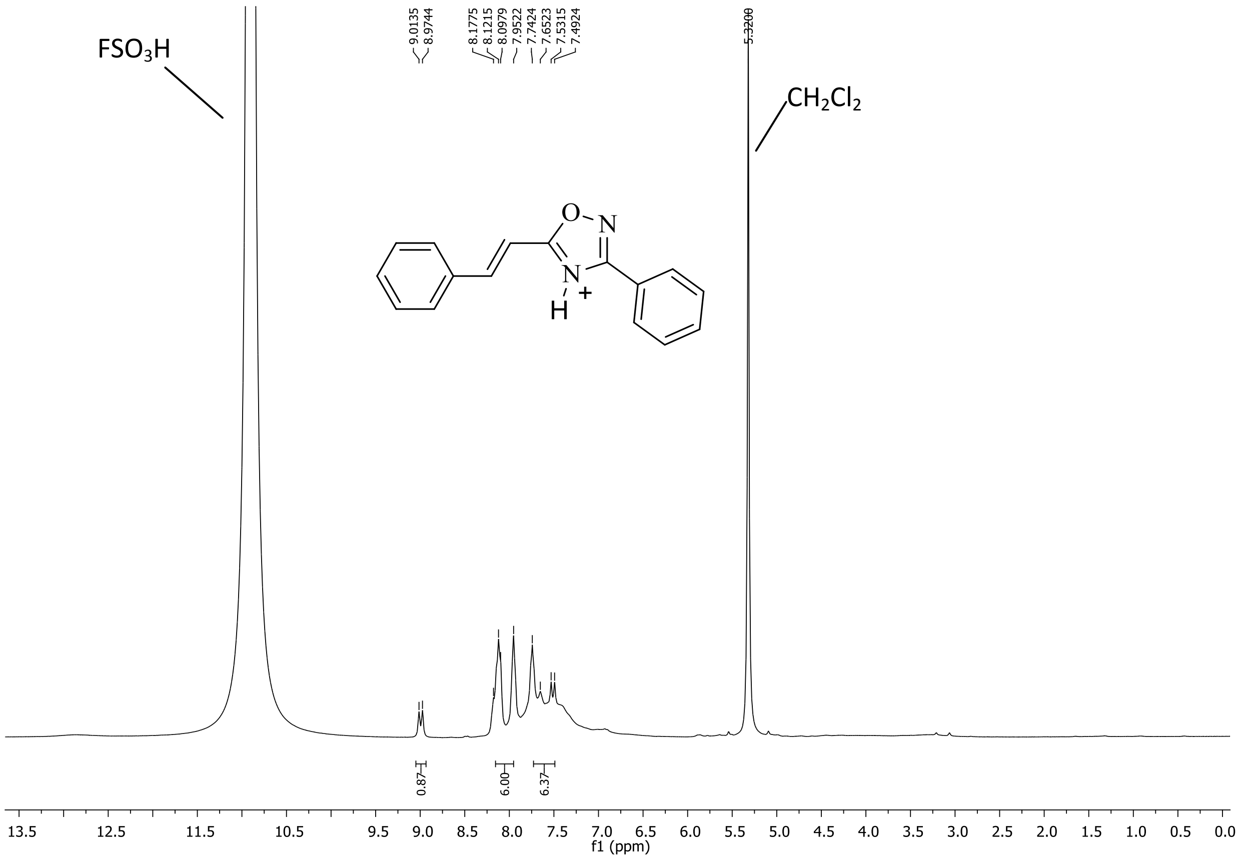

Fig. S79. ^{1}H NMR spectrum of cation **Ca** [400 MHz, FSO_3H, CH_2Cl_2, – 40 °C].

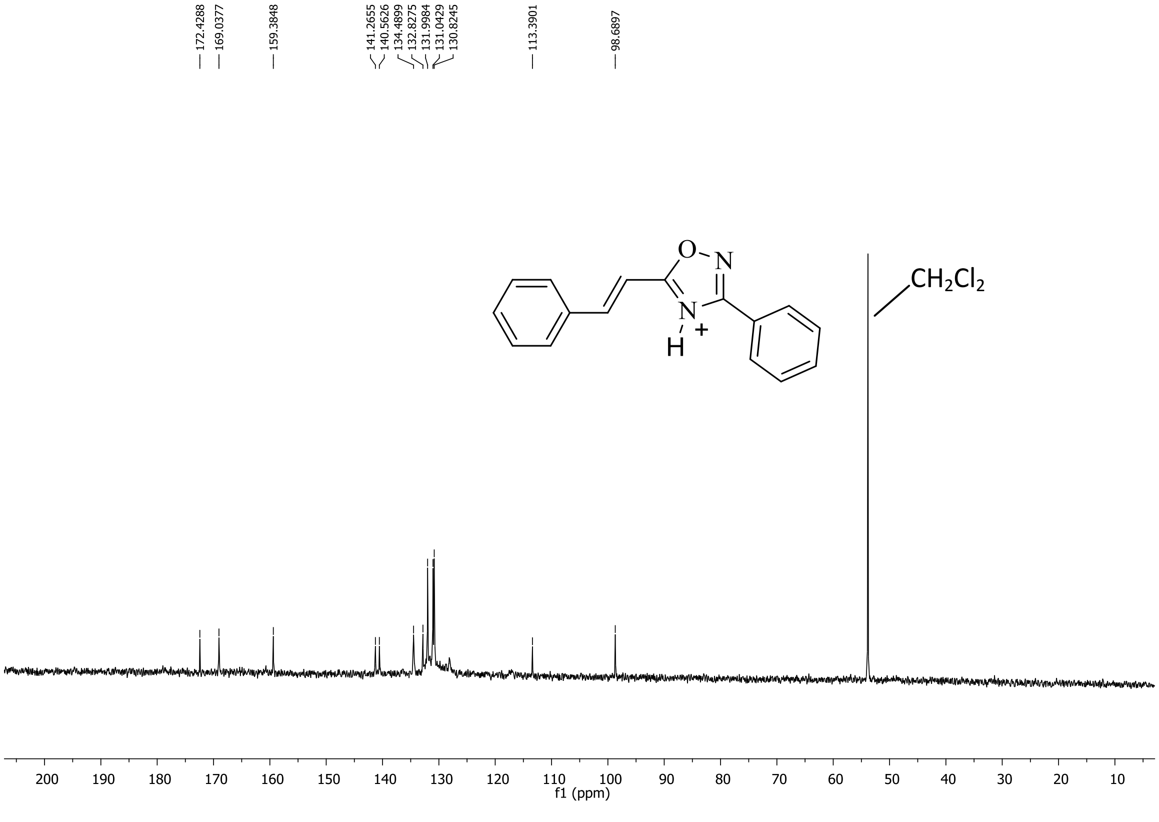

Fig. S80. ^{13}C NMR spectrum of cation **Ca** [125 MHz, FSO_3H, CH_2Cl_2, – 40 °C].

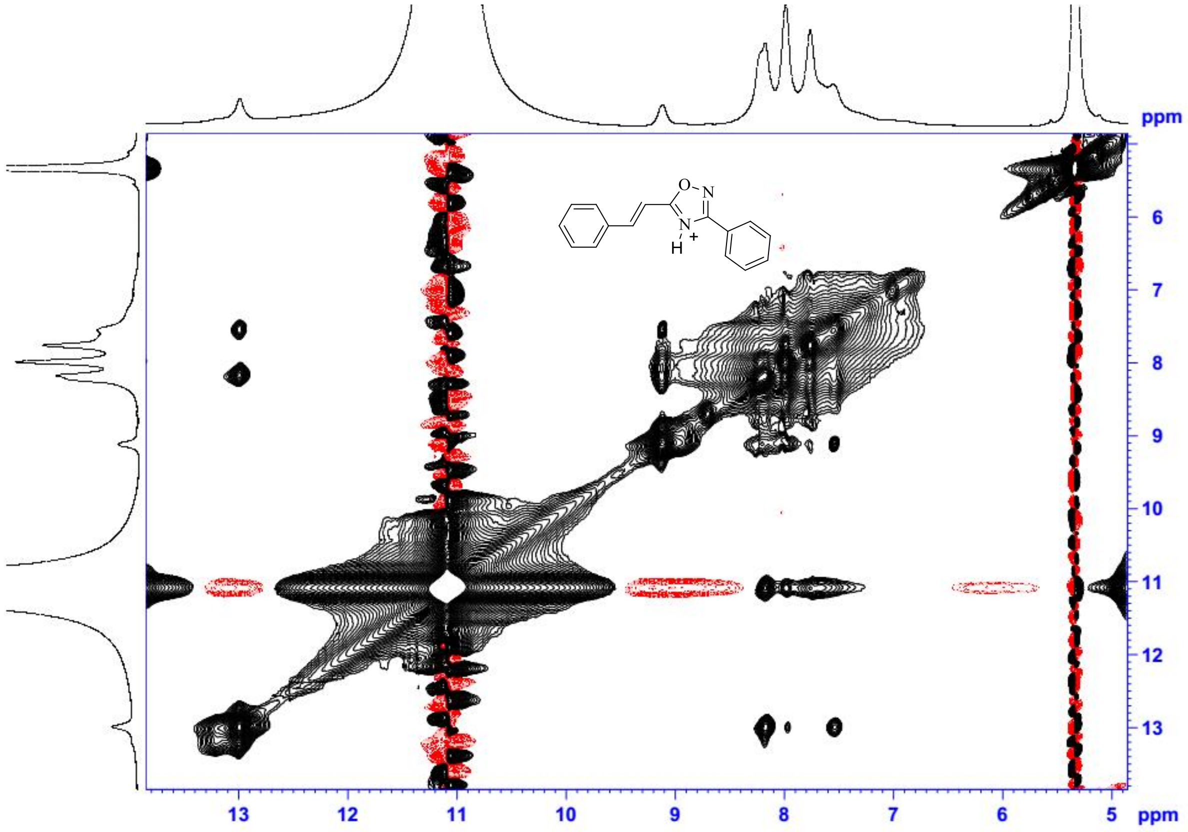

Fig. S81. NOESY spectrum of cation **Ca** in FSO_3H at – 80 °C.

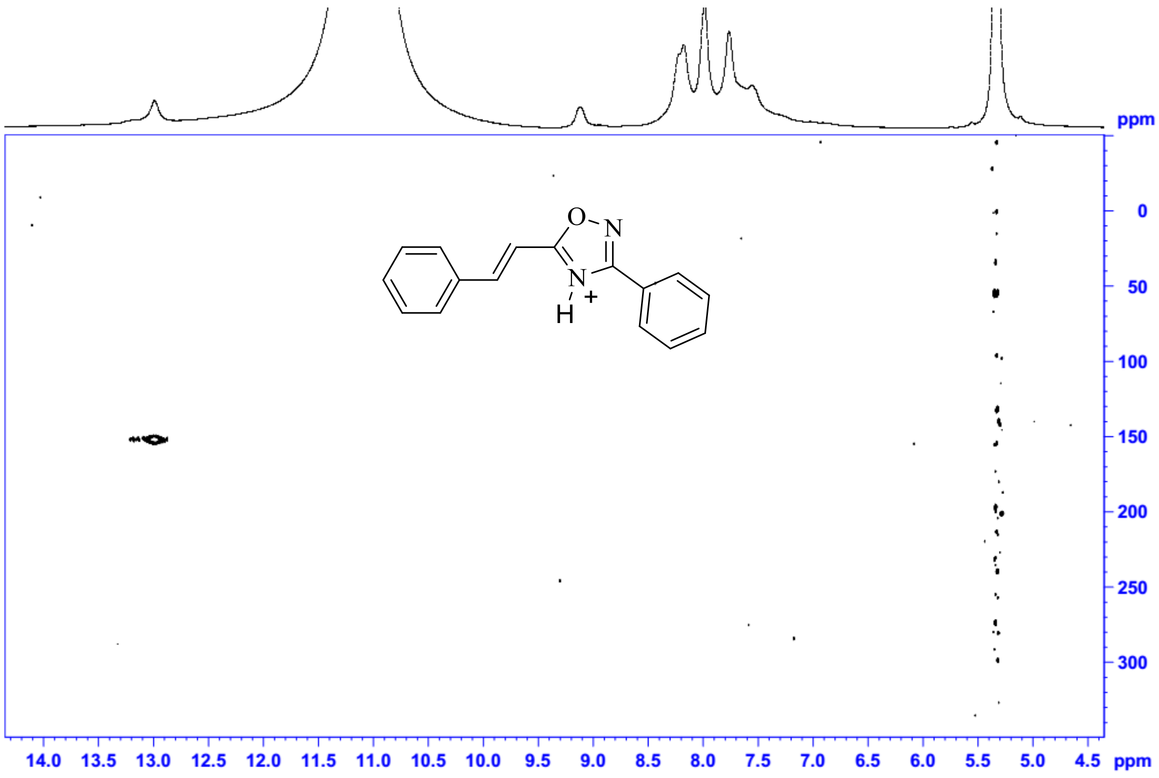

Fig. S82. ^1H – ^{15}N HSQC spectrum of cation **Ca** in FSO_3H at – 80 °C.

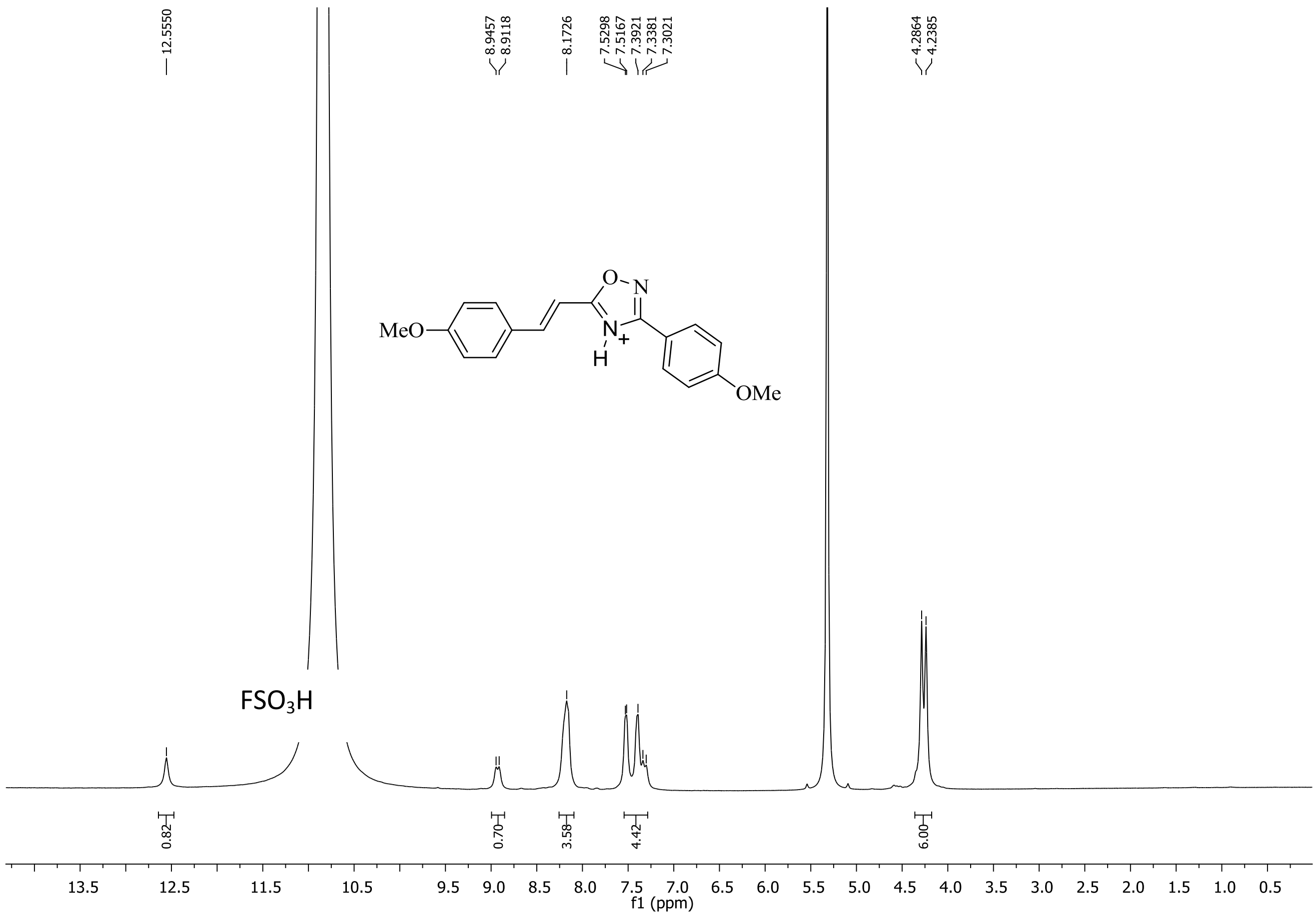

Fig. S83. ^{1}H NMR spectrum of cation **Cm** [400 MHz, FSO_3H, CH_2Cl_2, – 60 °C].

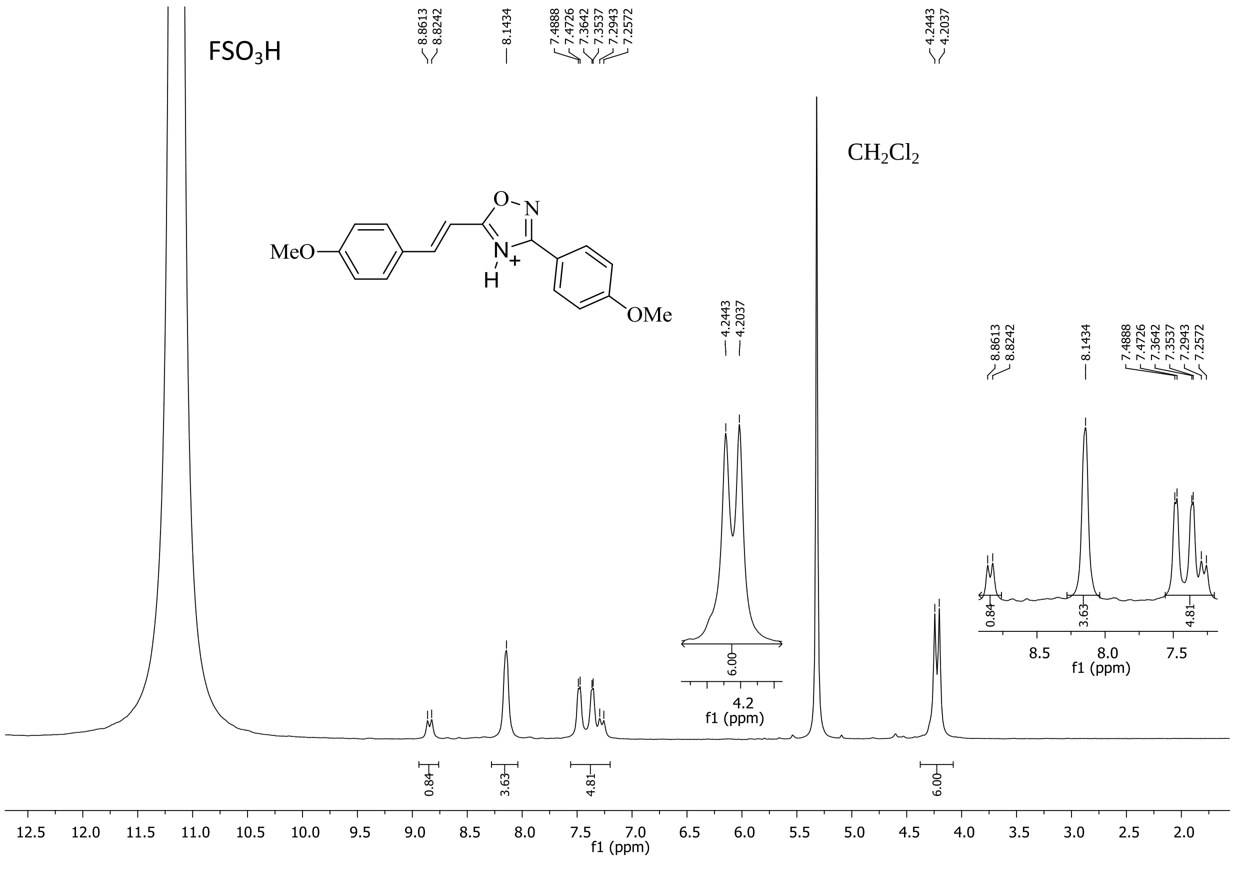

Fig. S84 ^{1}H NMR spectrum of cation **Cm** [400 MHz, FSO_3H, CH_2Cl_2, – 40 °C] .

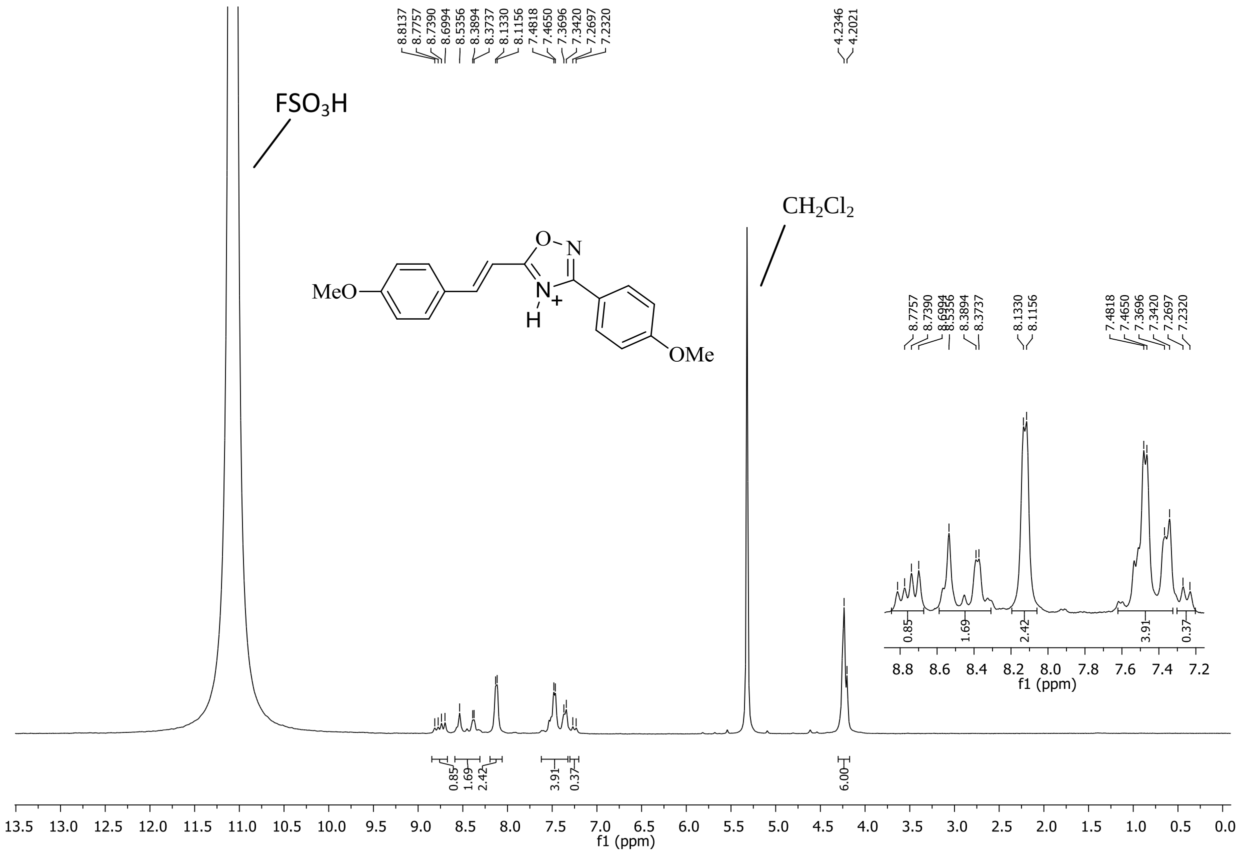

Fig. S85. ^{1}H NMR spectrum of of cation **Cm** [400 MHz, FSO_3H, CH_2Cl_2, – 20 °C].

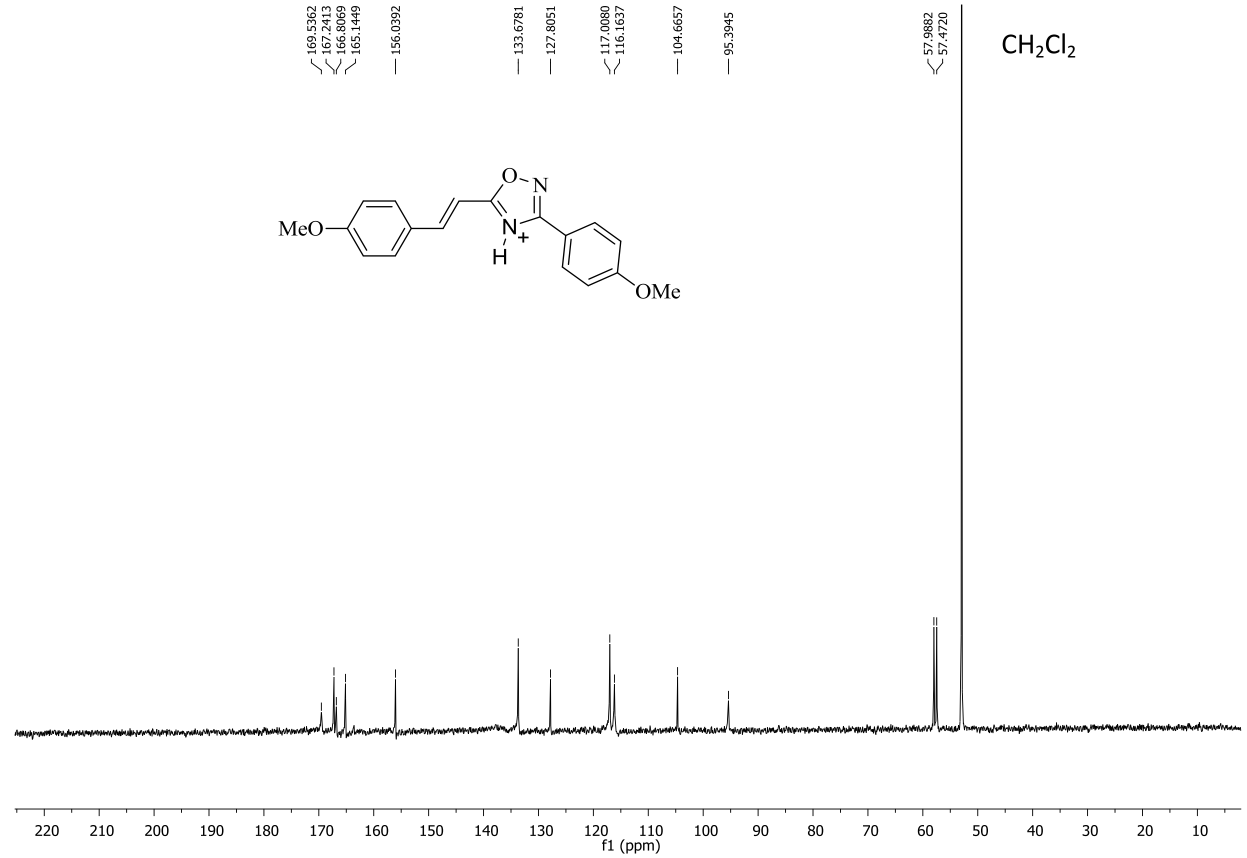

Fig. S86. ^{13}C NMR spectrum of of cation **Cm** [100 MHz, FSO_3H, CH_2Cl_2, – 60 °C].

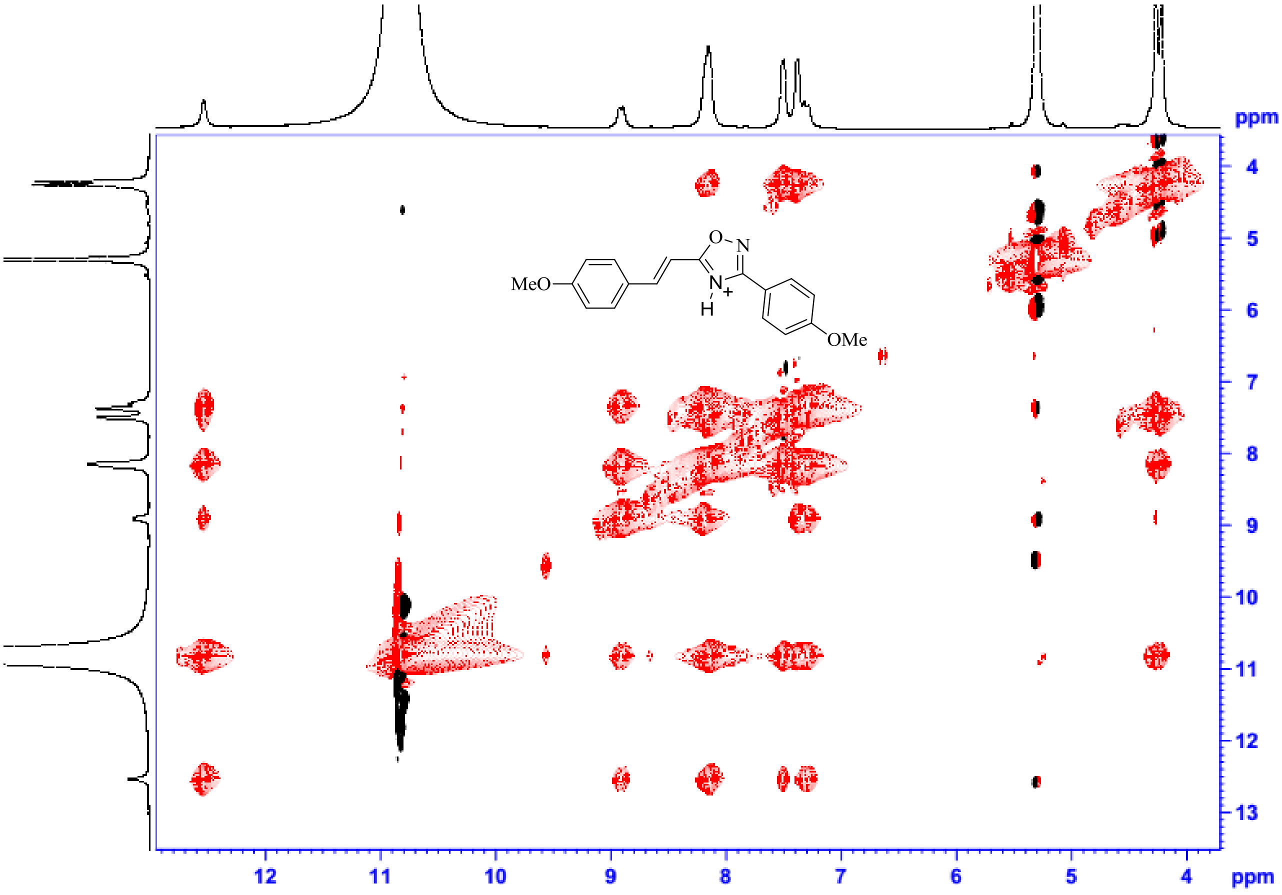

Fig. S87. NOESY spectrum of cation **Cm** in FSO_3H at – 60 °C.

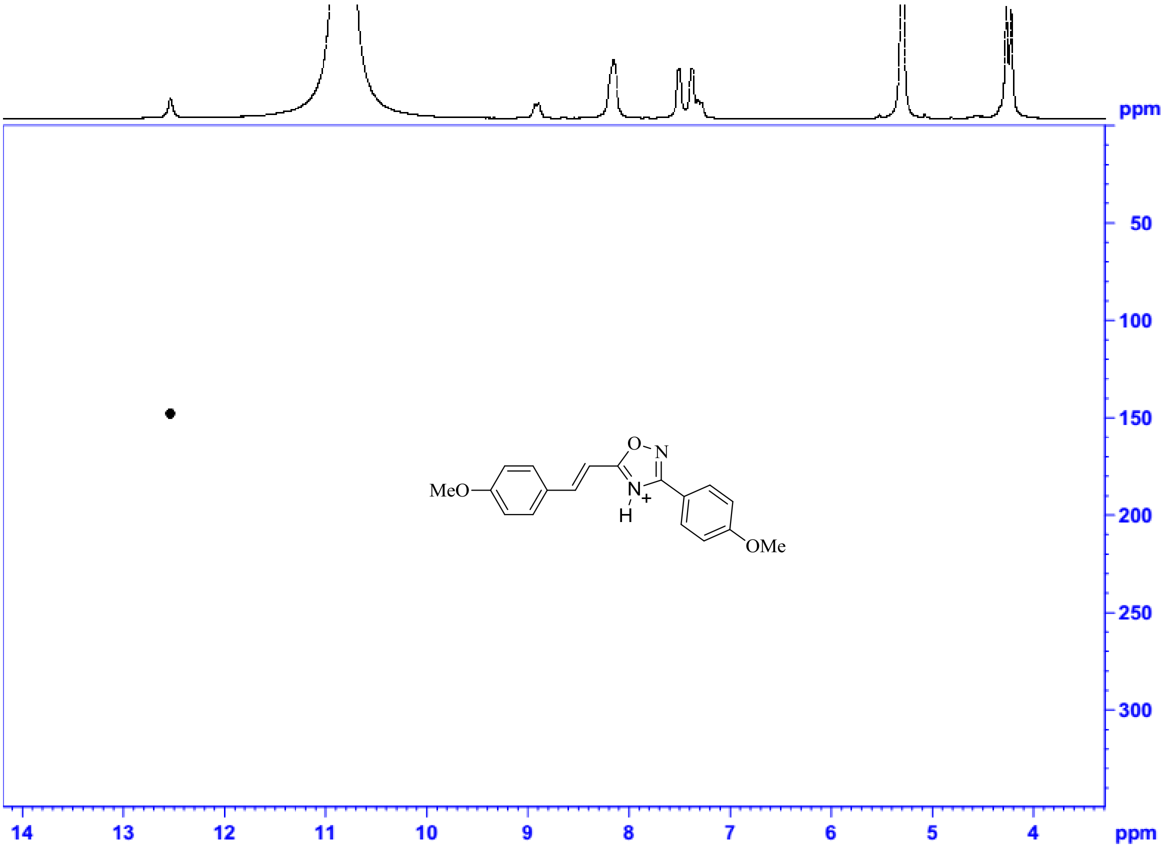

Fig. S88. ^{1}H–^{15}N HSQC spectrum of cation **Cm** in FSO_3H at – 60 °C.

3. Data on DFT calculations of 1a and cations A, B, C, D, E, F.

1a

E= -801.862023075 h, G^{298}= -801.66545 h, μ= 3.36 D

Optimized geometry, A

N_{at}	Atom	x	y	z
1	C	2.141284	-0.375311	-0.000005
2	O	0.311174	-1.484506	-0.000154
3	C	0.047558	-0.163779	0.000056
4	C	3.567717	-0.024468	0.000004
5	C	4.552729	-1.019730	0.000064
6	C	3.950306	1.320120	-0.000049
7	C	5.896183	-0.671083	0.000074
8	C	5.297603	1.663876	-0.000035
9	C	6.272759	0.671164	0.000024
10	H	4.263942	-2.060674	0.000105
11	H	3.191471	2.088147	-0.000098
12	H	6.650590	-1.445399	0.000121
13	H	5.584373	2.706250	-0.000074
14	H	7.320122	0.939609	0.000032
15	C	-1.312143	0.311890	0.000070
16	H	-1.400962	1.388122	0.000131
17	C	-2.384851	-0.499494	-0.000002
18	H	-2.213826	-1.569466	-0.000070
19	C	-3.787926	-0.098949	0.000003
20	C	-4.768289	-1.103311	-0.000037
21	C	-4.206965	1.242327	0.000047
22	C	-6.120586	-0.783563	-0.000031
23	C	-5.556238	1.559890	0.000054
24	C	-6.518858	0.549391	0.000015
25	H	-4.460807	-2.140709	-0.000073
26	H	-3.479446	2.041315	0.000073
27	H	-6.860089	-1.572138	-0.000062
28	H	-5.862240	2.596871	0.000087
29	H	-7.569893	0.802806	0.000020
30	N	1.712614	-1.614715	0.000004
31	N	1.133911	0.561500	-0.000071

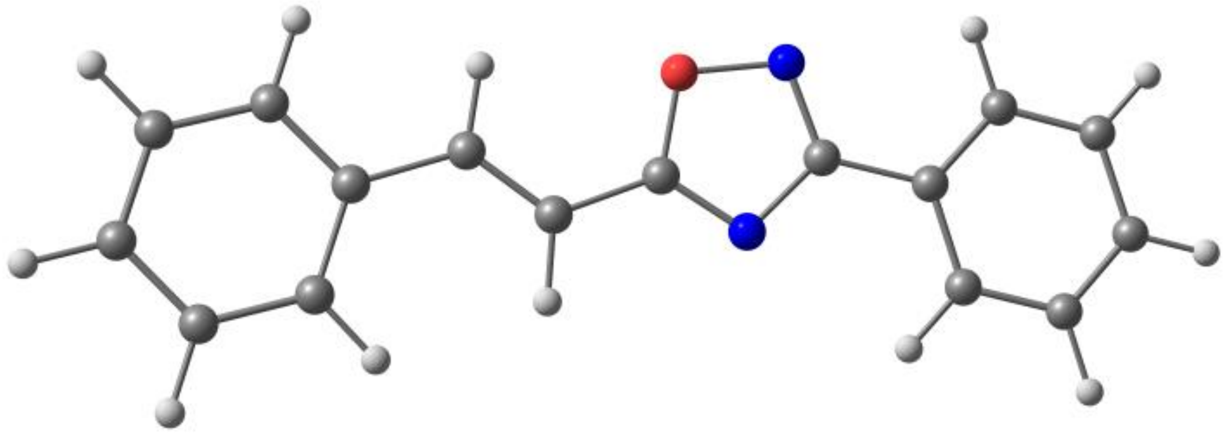

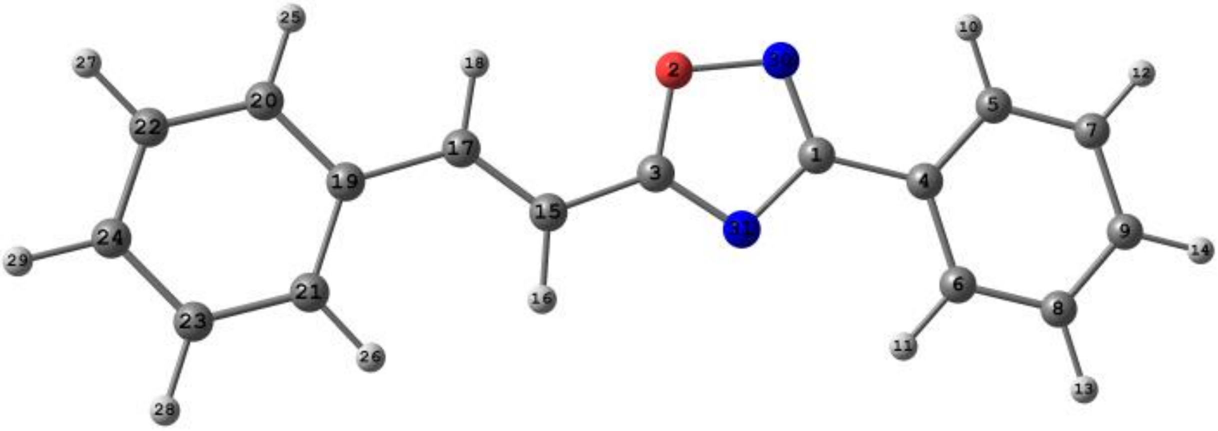

Summary of Natural Population Analysis: Natural charges, e

Atom	No	Natural Charge	Natural Population Core	Valence	Rydberg	Total
C	1	0.35531	1.99919	3.61437	0.03114	5.64469
O	2	-0.34787	1.99970	6.32036	0.02781	8.34787
C	3	0.55563	1.99918	3.41552	0.02967	5.44437
C	4	-0.11642	1.99876	4.08926	0.02839	6.11642
C	5	-0.16328	1.99891	4.14258	0.02179	6.16328
C	6	-0.56447	1.99893	3.37338	1.19217	6.56447
C	7	-0.21073	1.99917	4.18920	0.02236	6.21073
C	8	-0.23058	1.99918	4.19851	0.03288	6.23058
C	9	-0.18614	1.99914	4.16870	0.01830	6.18614
H	10	0.22190	0.00000	0.77567	0.00243	0.77810
H	11	0.21543	0.00000	0.77920	0.00537	0.78457
H	12	0.21700	0.00000	0.78111	0.00189	0.78300
H	13	0.21678	0.00000	0.78127	0.00195	0.78322
H	14	0.21580	0.00000	0.78246	0.00175	0.78420
C	15	-0.25926	1.99883	4.24143	0.01900	6.25926
H	16	0.22944	0.00000	0.76838	0.00218	0.77056
C	17	-0.10908	1.99908	4.08937	0.02063	6.10908
H	18	0.21663	0.00000	0.78119	0.00219	0.78337
C	19	-0.10314	1.99901	4.08489	0.01924	6.10314
C	20	-0.16794	1.99908	4.15018	0.01868	6.16794
C	21	-0.17200	1.99907	4.15490	0.01803	6.17200
C	22	-0.20638	1.99915	4.18698	0.02025	6.20638
C	23	-0.20154	1.99916	4.18233	0.02004	6.20154
C	24	-0.18666	1.99916	4.16729	0.02021	6.18666
H	25	0.21567	0.00000	0.78250	0.00182	0.78433
H	26	0.21356	0.00000	0.78450	0.00194	0.78644
H	27	0.21779	0.00000	0.78050	0.00171	0.78221
H	28	0.21714	0.00000	0.78116	0.00170	0.78286
H	29	0.21635	0.00000	0.78208	0.00157	0.78365
N	30	-0.19291	1.99934	5.16305	0.03053	7.19291
N	31	-0.53974	1.99935	5.50845	0.03193	7.53974
* Total *		-0.43370	37.98338	90.80079	1.64954	130.43370

Bond lengths, Å

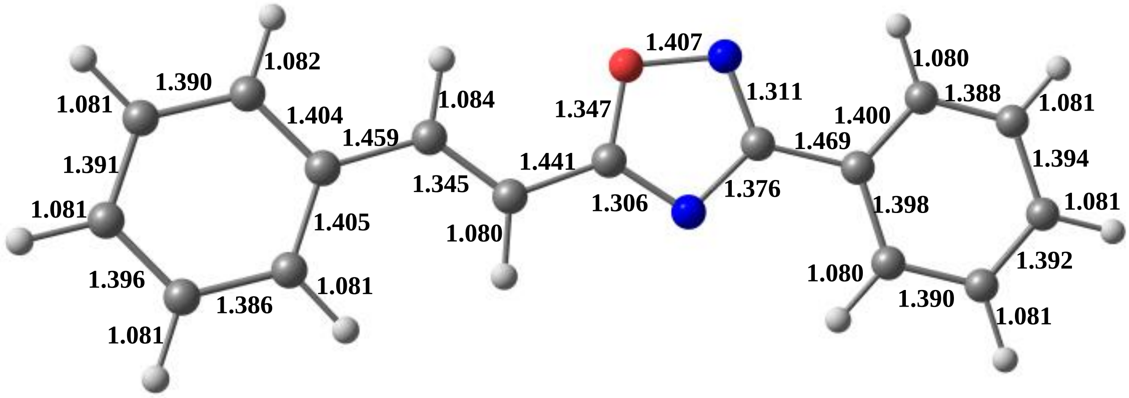

A

E= -802.279427851 h, G^{298}= -802.070538 h, μ= 5.87 D

Optimized geometry, A

N_{at}	Atom	x	y	z
1	C	2.142007	-0.339566	0.000008
2	O	0.268622	-1.499422	0.000001
3	C	0.021114	-0.161392	0.000017
4	C	3.552938	-0.004068	0.000006
5	C	4.544145	-0.995445	0.000005
6	C	3.914898	1.350069	0.000003
7	C	5.880658	-0.631525	0.000003
8	C	5.255413	1.703821	0.000002
9	C	6.237380	0.716297	0.000002
10	H	4.286558	-2.044683	0.000007
11	H	3.146573	2.107814	0.000003
12	H	6.643927	-1.395736	0.000003
13	H	5.534395	2.747439	0.000001
14	H	7.281712	0.994992	0.000001
15	C	-1.316199	0.321408	0.000020
16	H	-1.391709	1.397652	0.000042
17	C	-2.397417	-0.491764	-0.000006
18	H	-2.230590	-1.562393	-0.000028
19	C	-3.788738	-0.087568	-0.000009
20	C	-4.771417	-1.092675	-0.000016
21	C	-4.199489	1.258367	-0.000004
22	C	-6.120398	-0.767279	-0.000016
23	C	-5.545940	1.579173	-0.000005
24	C	-6.509982	0.568862	-0.000010
25	H	-4.465743	-2.130228	-0.000022
26	H	-3.468764	2.053930	-0.000002
27	H	-6.864607	-1.550823	-0.000021
28	H	-5.850791	2.616057	-0.000002
29	H	-7.559952	0.826300	-0.000011
30	N	1.638458	-1.569323	-0.000007
31	N	1.128934	0.551729	0.000014
32	H	2.024429	-2.502089	-0.000030

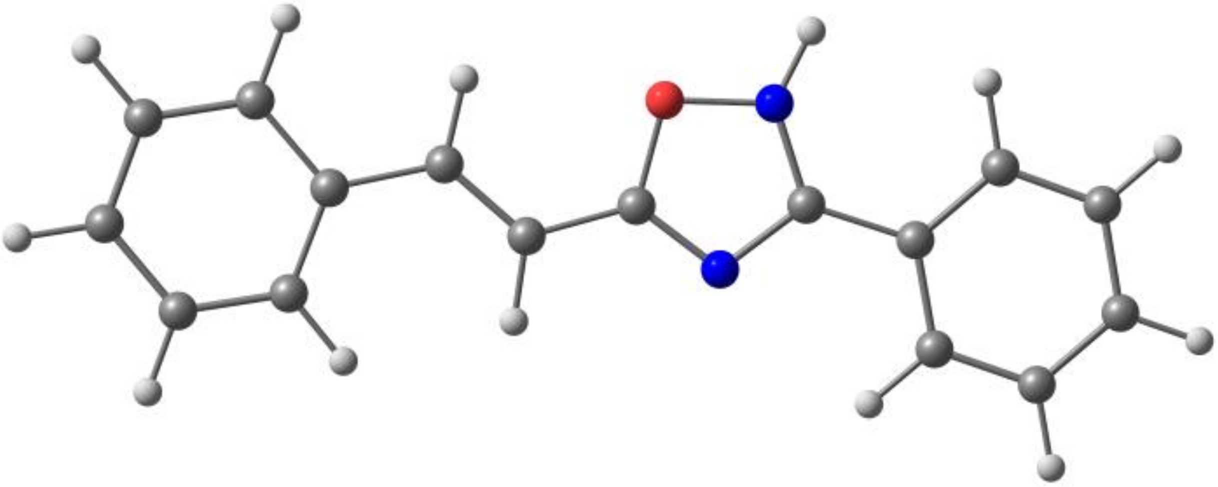

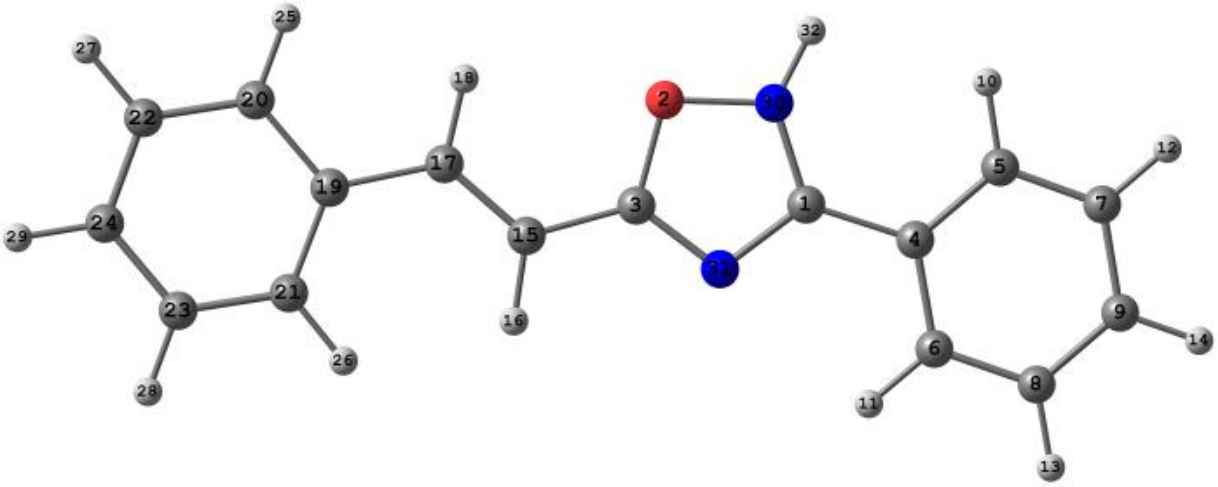

Summary of Natural Population Analysis: Natural charges, e

Atom	No	Natural Charge	Natural Population Core	Valence	Rydberg	Total
C	1	0.45713	1.99917	3.49787	0.04584	5.54287
O	2	-0.28305	1.99970	6.25966	0.02369	8.28305
C	3	0.59248	1.99914	3.36959	0.03879	5.40752
C	4	-0.54795	1.99899	4.32441	0.22455	6.54795
C	5	-0.17293	1.99892	4.11988	0.05413	6.17293
C	6	0.24836	1.99897	2.54877	1.20391	5.75164
C	7	-0.26933	1.99917	4.17397	0.09619	6.26933
C	8	-0.66857	1.99920	4.41191	0.25747	6.66857
C	9	-0.21176	1.99914	4.13122	0.08140	6.21176
H	10	0.21299	0.00000	0.77949	0.00752	0.78701
H	11	0.07765	0.00000	0.79499	0.12736	0.92235
H	12	0.22554	0.00000	0.77133	0.00314	0.77446
H	13	0.21819	0.00000	0.77353	0.00828	0.78181
H	14	0.22071	0.00000	0.77527	0.00402	0.77929
C	15	-0.29245	1.99883	4.27220	0.02142	6.29245
H	16	0.24451	0.00000	0.75273	0.00276	0.75549
C	17	-0.04357	1.99909	4.02472	0.01975	6.04357
H	18	0.22241	0.00000	0.77572	0.00187	0.77759

C	19	-0.12247	1.99902	4.10405	0.01940	6.12247
C	20	-0.13429	1.99891	4.11555	0.01984	6.13429
C	21	-0.15296	1.99907	4.13652	0.01737	6.15296
C	22	-0.21326	1.99917	4.19304	0.02105	6.21326
C	23	-0.20071	1.99916	4.18153	0.02003	6.20071
C	24	-0.16078	1.99916	4.14235	0.01927	6.16078
H	25	0.21860	0.00000	0.77972	0.00168	0.78140
H	26	0.21704	0.00000	0.78106	0.00190	0.78296
H	27	0.22194	0.00000	0.77640	0.00166	0.77806
H	28	0.22127	0.00000	0.77703	0.00171	0.77873
H	29	0.21963	0.00000	0.77884	0.00153	0.78037
N	30	-0.18910	1.99917	5.16007	0.02986	7.18910
N	31	-0.51458	1.99932	5.46873	0.04653	7.51458
H	32	0.46384	0.00000	0.53357	0.00259	0.53616
* Total *		0.10451	37.98328	90.48570	2.42651	130.89549

Bond lengths, Å

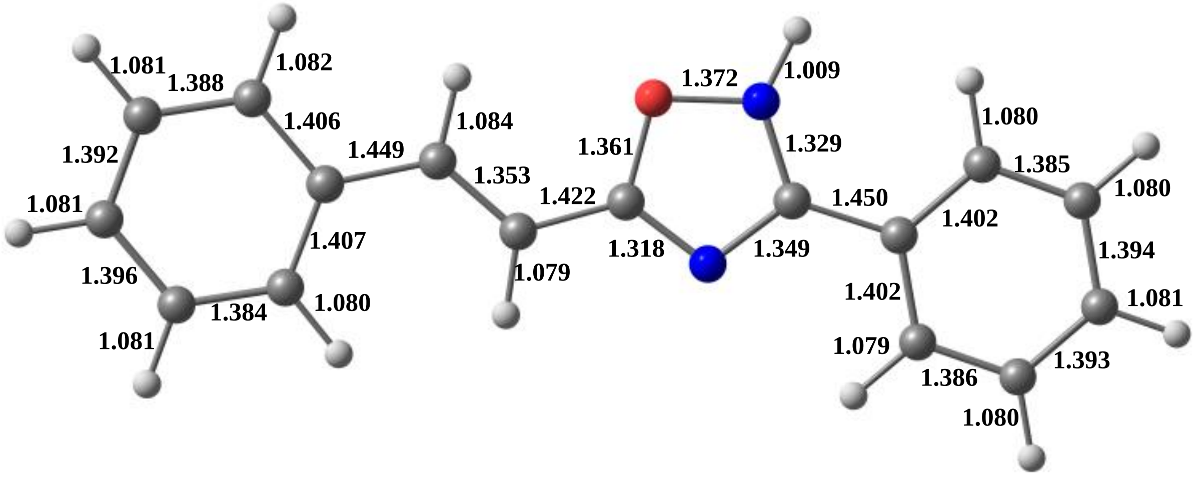

B

E= -802.655894994 h, G^{298}= -802.434876 h, μ= 10.45 D

Optimized geometry, A

N	atom	x	y	z
1	C	2.131177	-0.294993	0.043108
2	O	0.182350	-1.315262	0.055081
3	C	0.050669	0.000699	0.295053
4	C	3.548059	-0.034530	-0.041160
5	C	4.479341	-1.068240	-0.229743
6	C	3.983207	1.294609	0.074733
7	C	5.828036	-0.767887	-0.303660
8	C	5.336156	1.582393	-0.002368
9	C	6.256999	0.554792	-0.191020
10	H	4.165013	-2.098438	-0.314425
11	H	3.261345	2.083076	0.221229
12	H	6.546405	-1.561364	-0.447090
13	H	5.672824	2.604891	0.084953
14	H	7.311986	0.782165	-0.249393
15	C	-1.307772	0.561109	0.535630
16	H	-1.418951	1.453552	-0.077518
17	C	-2.432878	-0.394286	0.399729
18	H	-2.212863	-1.438283	0.589072
19	C	-3.741838	-0.079374	0.117908
20	C	-4.693132	-1.153823	0.114564
21	C	-4.193345	1.253715	-0.154356
22	C	-6.017794	-0.903462	-0.139704
23	C	-5.520173	1.483833	-0.401934
24	C	-6.427830	0.410442	-0.395561
25	H	-4.347735	-2.156817	0.318969
26	H	-3.493724	2.074829	-0.166654
27	H	-6.739008	-1.706102	-0.143263
28	H	-5.873319	2.482749	-0.606820
29	H	-7.471785	0.606502	-0.597287
30	N	1.533216	-1.471151	-0.099909
31	N	1.174334	0.644759	0.294344
32	H	1.834996	-2.411890	-0.316490
33	H	-1.320140	0.921991	1.575732

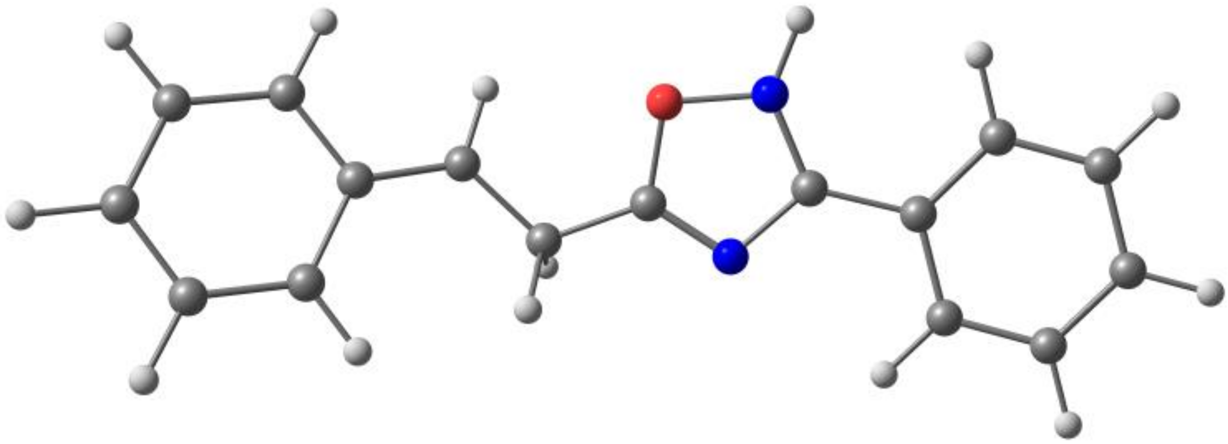

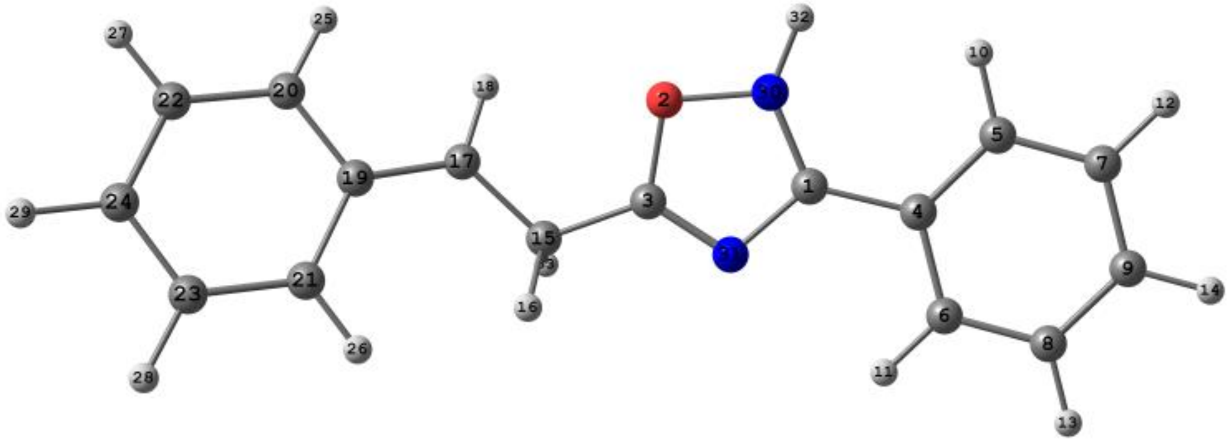

Summary of Natural Population Analysis: Natural charges, e

Atom	No	Natural Charge	Natural Population: Core	Valence	Rydberg	Total
C	1	0.47229	1.99916	3.48452	0.04402	5.52771
O	2	-0.26267	1.99968	6.23926	0.02372	8.26267
C	3	0.65697	1.99923	3.30351	0.04029	5.34303
C	4	-0.54177	1.99898	4.32575	0.21704	6.54177
C	5	-0.16249	1.99892	4.11030	0.05327	6.16249
C	6	0.24969	1.99898	2.55689	1.19445	5.75031
C	7	-0.26756	1.99917	4.17291	0.09548	6.26756
C	8	-0.66207	1.99919	4.40730	0.25558	6.66207
C	9	-0.19806	1.99914	4.11868	0.08023	6.19806
H	10	0.21617	0.00000	0.77653	0.00729	0.78383
H	11	0.05944	0.00000	0.80944	0.13112	0.94056
H	12	0.22802	0.00000	0.76895	0.00303	0.77198
H	13	0.22011	0.00000	0.77124	0.00865	0.77989
H	14	0.22277	0.00000	0.77331	0.00392	0.77723
C	15	-0.54036	1.99908	4.51974	0.02154	6.54036
H	16	0.29515	0.00000	0.70225	0.00260	0.70485
C	17	0.18777	1.99906	3.79642	0.01675	5.81223
H	18	0.24010	0.00000	0.75798	0.00192	0.75990
C	19	-0.15468	1.99898	4.13623	0.01946	6.15468
C	20	-0.02409	1.99910	4.00705	0.01794	6.02409
C	21	-0.05057	1.99909	4.03450	0.01698	6.05057
C	22	-0.21796	1.99914	4.19891	0.01990	6.21796
C	23	-0.20502	1.99915	4.18603	0.01984	6.20502
C	24	-0.00315	1.99919	3.98553	0.01843	6.00315
H	25	0.23914	0.00000	0.75924	0.00162	0.76086
H	26	0.23545	0.00000	0.76274	0.00182	0.76455
H	27	0.24472	0.00000	0.75367	0.00161	0.75528
H	28	0.24378	0.00000	0.75463	0.00159	0.75622
H	29	0.23657	0.00000	0.76214	0.00129	0.76343
N	30	-0.17346	1.99916	5.14468	0.02962	7.17346
N	31	-0.49164	1.99928	5.44404	0.04832	7.49164
H	32	0.47282	0.00000	0.52468	0.00249	0.52718
H	33	0.33042	0.00000	0.66787	0.00171	0.66958

* Total *   1.09582   37.98368   90.51696   2.40353   130.90418

Bond lengths, Å

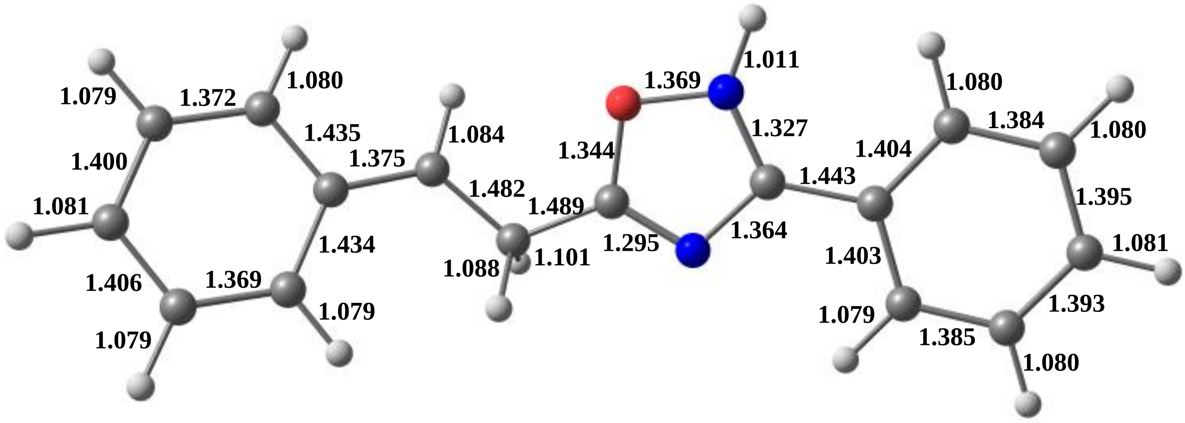

C
E= -802.288502765 h, G^{298}= -802.077771 h, μ= 4.61 D

Optimized geometry, A

N_{at}	Atom	x	y	z
1	C	2.165370	-0.361810	0.000005
2	O	0.287517	-1.433314	-0.000007
3	C	-0.022733	-0.151686	0.000033
4	C	3.583669	-0.016630	-0.000002
5	C	4.534011	-1.047023	0.000038
6	C	4.004649	1.316819	-0.000049
7	C	5.885109	-0.739978	0.000033
8	C	5.361165	1.615020	-0.000053
9	C	6.301622	0.590297	-0.000011
10	H	4.211518	-2.077472	0.000075
11	H	3.297097	2.132850	-0.000088
12	H	6.614242	-1.537269	0.000065
13	H	5.679725	2.647236	-0.000089
14	H	7.356421	0.825988	-0.000013
15	C	-1.346756	0.347070	0.000052
16	H	-1.442496	1.421258	0.000108
17	C	-2.418997	-0.484420	-0.000005
18	H	-2.231071	-1.551326	-0.000058
19	C	-3.812048	-0.103578	-0.000006
20	C	-4.777146	-1.127145	-0.000046
21	C	-4.245022	1.236342	0.000029
22	C	-6.130636	-0.824565	-0.000047
23	C	-5.596061	1.533322	0.000027
24	C	-6.541728	0.505340	-0.000010
25	H	-4.452975	-2.158944	-0.000076
26	H	-3.528506	2.044609	0.000054
27	H	-6.862002	-1.619910	-0.000076
28	H	-5.919699	2.564330	0.000053
29	H	-7.595919	0.744791	-0.000011
30	N	1.700779	-1.576773	-0.000020
31	N	1.117205	0.542007	0.000038
32	H	1.180840	1.549479	0.000060

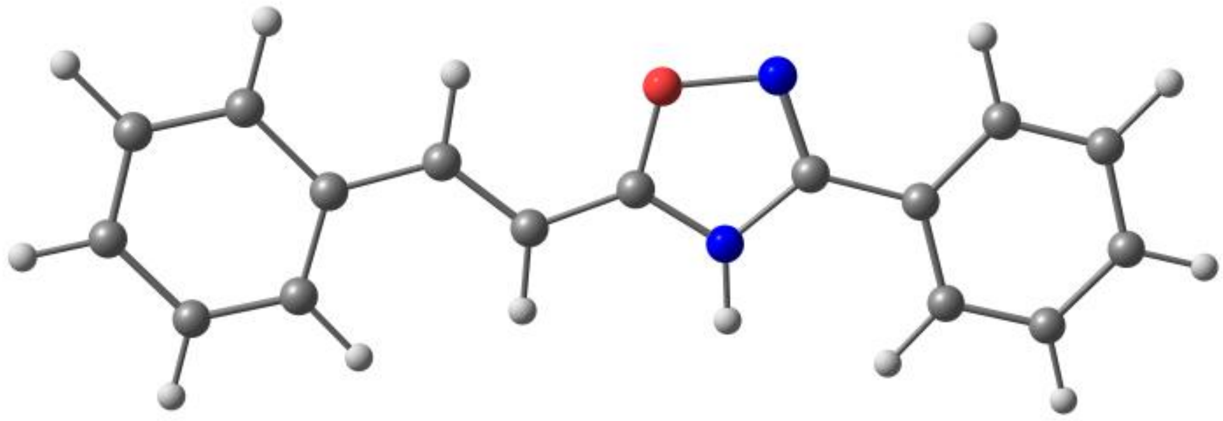

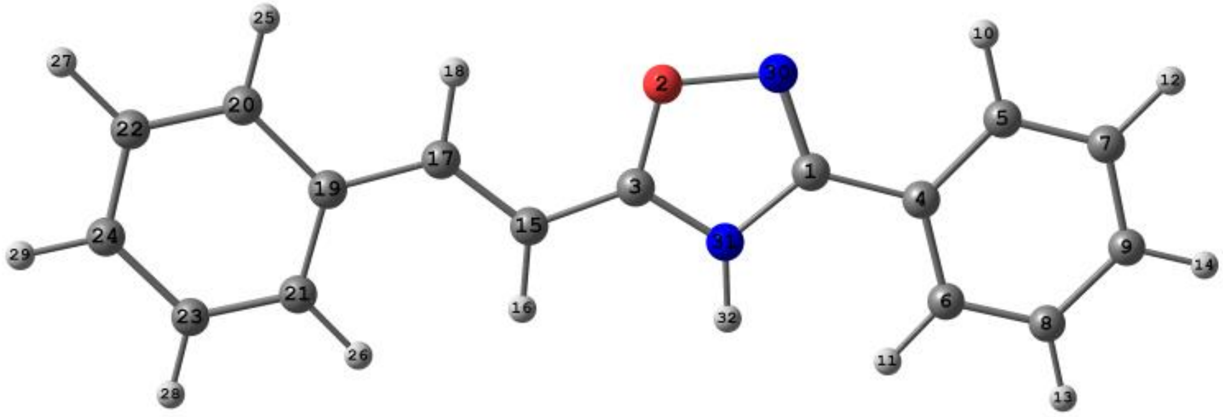

Summary of Natural Population Analysis: Natural charges, e

Atom	No	Natural Charge	Natural Population Core	Valence	Rydberg	Total
C	1	0.39132	1.99917	3.55940	0.05011	5.60868
O	2	-0.30273	1.99968	6.27756	0.02549	8.30273
C	3	0.64449	1.99917	3.32236	0.03398	5.35551
C	4	-0.55413	1.99898	4.32440	0.23076	6.55413
C	5	-0.17745	1.99893	4.12351	0.05501	6.17745
C	6	0.24856	1.99896	2.55255	1.19993	5.75144
C	7	-0.27306	1.99916	4.17248	0.10142	6.27306
C	8	-0.67018	1.99920	4.40543	0.26555	6.67018
C	9	-0.22936	1.99914	4.14672	0.08350	6.22936
H	10	0.22383	0.00000	0.76851	0.00766	0.77617
H	11	0.04680	0.00000	0.81753	0.13567	0.95320
H	12	0.22285	0.00000	0.77383	0.00332	0.77715
H	13	0.21688	0.00000	0.77432	0.00880	0.78312
H	14	0.21943	0.00000	0.77650	0.00407	0.78057
C	15	-0.31060	1.99885	4.29101	0.02074	6.31060
H	16	0.24726	0.00000	0.75007	0.00267	0.75274
C	17	-0.01940	1.99910	4.00017	0.02012	6.01940
H	18	0.22672	0.00000	0.77130	0.00198	0.77328
C	19	-0.12889	1.99902	4.11045	0.01942	6.12889
C	20	-0.12560	1.99891	4.10688	0.01981	6.12560
C	21	-0.14590	1.99907	4.12952	0.01731	6.14590
C	22	-0.21347	1.99917	4.19326	0.02104	6.21347
C	23	-0.20052	1.99916	4.18135	0.02002	6.20052
C	24	-0.15120	1.99916	4.13290	0.01914	6.15120
H	25	0.22031	0.00000	0.77803	0.00167	0.77969
H	26	0.21785	0.00000	0.78023	0.00192	0.78215
H	27	0.22355	0.00000	0.77480	0.00165	0.77645
H	28	0.22282	0.00000	0.77548	0.00170	0.77718
H	29	0.22088	0.00000	0.77762	0.00151	0.77912
N	30	-0.14872	1.99932	5.11306	0.03634	7.14872
N	31	-0.52231	1.99918	5.49394	0.02919	7.52231
H	32	0.46657	0.00000	0.52740	0.00602	0.53343
* Total *		0.08658	37.98331	90.48260	2.44750	130.91342

Bond lengths, Å

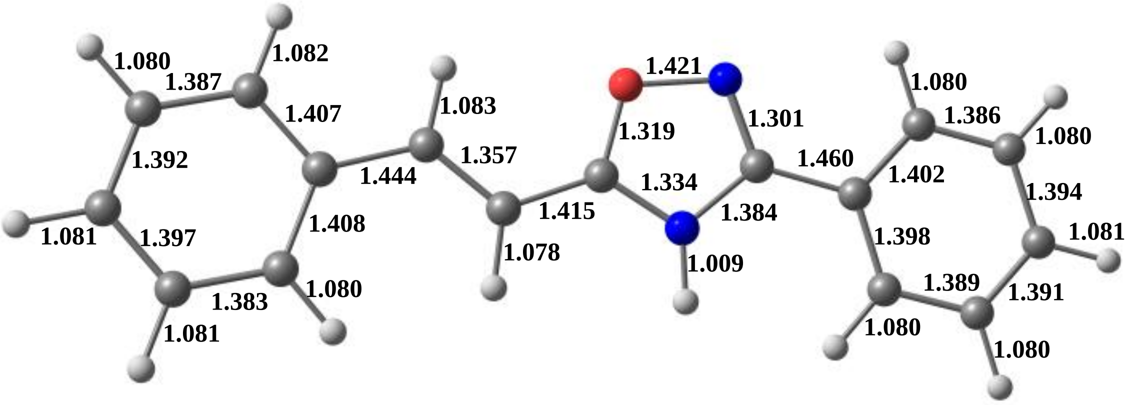

D

E= -802.660438977 h, G^{298}= -802.440318 h, μ= 14.47 D

Optimized geometry, A

N_{at}	Atom	x	y	z
1	C	2.159738	-0.316404	0.136997
2	O	0.233960	-1.260718	0.387165
3	C	0.011946	0.017422	0.338125
4	C	3.579038	-0.044236	-0.031235
5	C	4.429128	-1.085918	-0.426443
6	C	4.097260	1.232454	0.213252
7	C	5.785448	-0.844575	-0.573529
8	C	5.457338	1.462619	0.061999
9	C	6.300805	0.427433	-0.330168
10	H	4.024906	-2.067325	-0.624986
11	H	3.461146	2.042851	0.538863
12	H	6.440593	-1.645967	-0.882507
13	H	5.856559	2.447615	0.254301
14	H	7.359290	0.611212	-0.447650
15	C	-1.348213	0.603089	0.476965
16	H	-1.441459	1.449627	-0.202155
17	C	-2.453499	-0.382277	0.336798
18	H	-2.203483	-1.423761	0.497720
19	C	-3.768743	-0.091694	0.069807
20	C	-4.690534	-1.192820	0.033711
21	C	-4.257504	1.236949	-0.163014
22	C	-6.020385	-0.972349	-0.218256
23	C	-5.588761	1.435906	-0.409384
24	C	-6.465715	0.336697	-0.438623
25	H	-4.317426	-2.190998	0.210639
26	H	-3.582378	2.078340	-0.144243
27	H	-6.718931	-1.794204	-0.247404
28	H	-5.970311	2.429730	-0.585730
29	H	-7.513918	0.509157	-0.639668
30	N	1.630342	-1.499600	0.265802
31	N	1.156243	0.646476	0.182474
32	H	1.271823	1.647155	0.085483
33	H	-1.408268	1.030401	1.490068

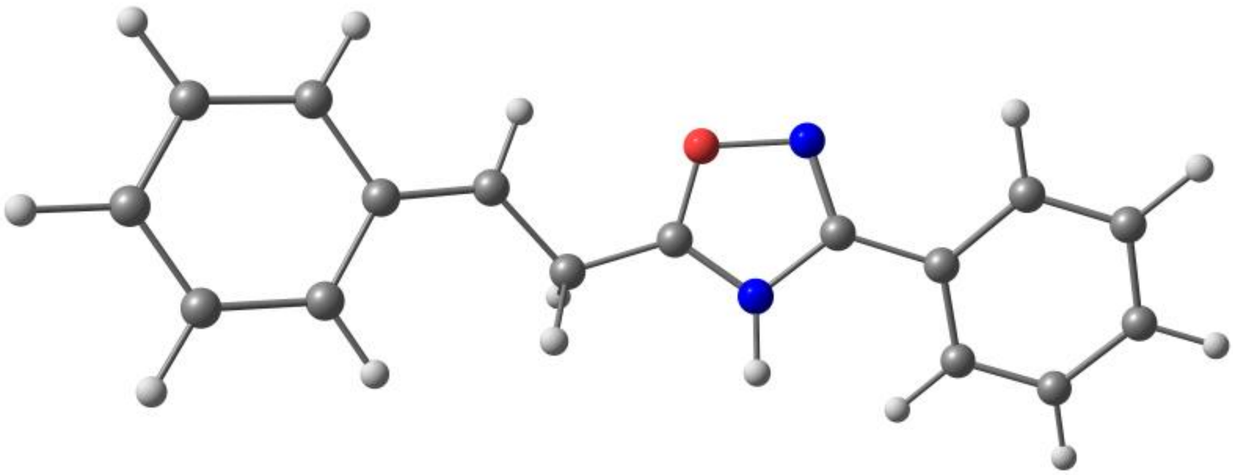

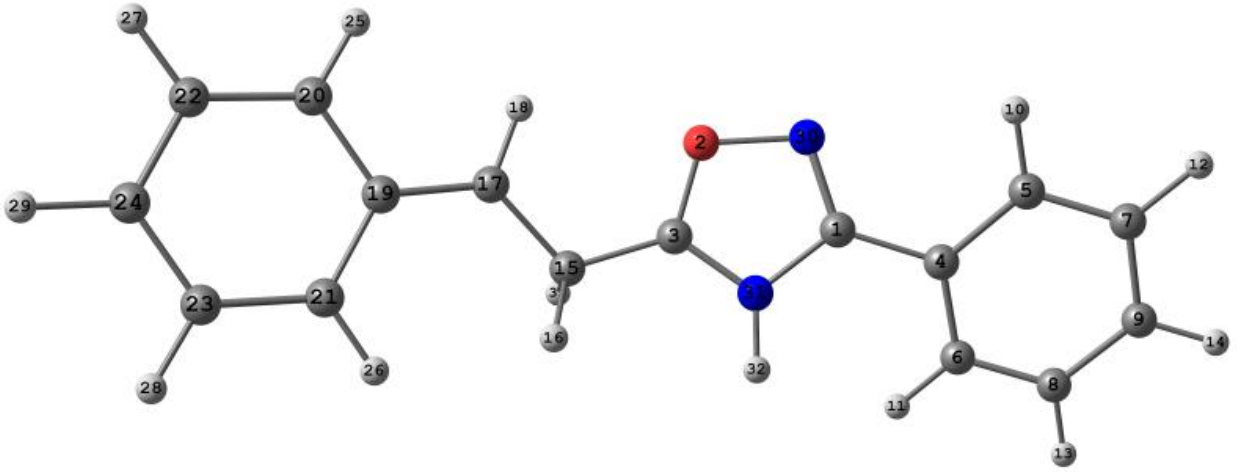

Summary of Natural Population Analysis: Natural charges, e

Atom	No	Natural Charge	Core	Valence	Rydberg	Total
			Natural Population			
C	1	0.42267	1.99918	3.55020	0.02796	5.57733
O	2	-0.27396	1.99965	6.24828	0.02603	8.27396
C	3	0.73074	1.99928	3.24351	0.02647	5.26926
C	4	-0.13705	1.99870	4.11588	0.02247	6.13705
C	5	-0.13518	1.99891	4.11589	0.02038	6.13518
C	6	-0.14716	1.99891	4.12865	0.01960	6.14716
C	7	-0.19896	1.99917	4.17873	0.02106	6.19896
C	8	-0.19937	1.99918	4.17894	0.02125	6.19937
C	9	-0.15493	1.99916	4.13703	0.01874	6.15493
H	10	0.23141	0.00000	0.76655	0.00204	0.76859
H	11	0.21990	0.00000	0.77827	0.00184	0.78010
H	12	0.22648	0.00000	0.77188	0.00163	0.77352
H	13	0.22718	0.00000	0.77119	0.00163	0.77282
H	14	0.22392	0.00000	0.77456	0.00152	0.77608
C	15	-0.53954	1.99910	4.52354	0.01691	6.53954
H	16	0.30226	0.00000	0.69586	0.00188	0.69774
C	17	0.18139	1.99906	3.80210	0.01745	5.81861
H	18	0.24388	0.00000	0.75387	0.00225	0.75612
C	19	-0.15120	1.99898	4.13260	0.01962	6.15120
C	20	-0.02007	1.99910	4.00298	0.01799	6.02007
C	21	-0.05004	1.99909	4.03399	0.01697	6.05004
C	22	-0.21811	1.99914	4.19909	0.01989	6.21811
C	23	-0.20395	1.99915	4.18494	0.01986	6.20395
C	24	0.00310	1.99919	3.97929	0.01842	5.99690
H	25	0.24007	0.00000	0.75828	0.00165	0.75993
H	26	0.23642	0.00000	0.76178	0.00180	0.76358
H	27	0.24581	0.00000	0.75259	0.00160	0.75419
H	28	0.24476	0.00000	0.75366	0.00158	0.75524
H	29	0.23731	0.00000	0.76141	0.00128	0.76269
N	30	-0.11931	1.99933	5.08768	0.03231	7.11931
N	31	-0.48720	1.99915	5.47115	0.01689	7.48720
H	32	0.48337	0.00000	0.51408	0.00255	0.51663

H 33	0.33541	0.00000	0.66281	0.00178	0.66459

===

* Total *	2.00003	37.98345	91.59122	0.42530	129.99997

Bond lengths, Å

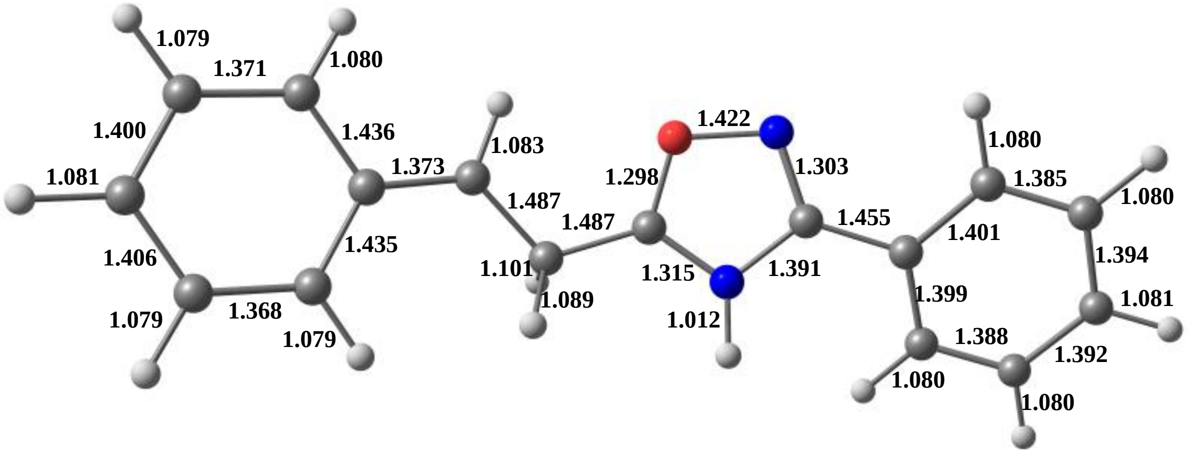

E

E= -802,678502866 h, **G^{298}= -802.456591 h,** **μ= 6.11 D,** 1 imaginary frequency=**-68.7 cm^{-1}**

Optimized geometry, A

N_{at}	Atom	x	y	z
1	C	-2.168020	-0.325266	-0.000007
2	O	-0.249590	-1.458453	0.000001
3	C	0.054111	-0.152975	-0.000020
4	C	-3.565186	0.003886	-0.000002
5	C	-4.527696	-1.022285	-0.000031
6	C	-3.971144	1.349778	0.000034
7	C	-5.871019	-0.699927	-0.000028
8	C	-5.319690	1.657483	0.000037
9	C	-6.267996	0.637121	0.000005
10	H	-4.240107	-2.062716	-0.000060
11	H	-3.256139	2.158242	0.000065
12	H	-6.609667	-1.487197	-0.000053
13	H	-5.631096	2.691097	0.000064
14	H	-7.320134	0.883244	0.000006
15	C	1.349738	0.342035	-0.000028
16	H	1.439439	1.416169	-0.000052
17	C	2.441522	-0.491865	-0.000001
18	H	2.261933	-1.560246	0.000025
19	C	3.812820	-0.098451	0.000001
20	C	4.791163	-1.117559	0.000043
21	C	4.230213	1.252184	-0.000032
22	C	6.137182	-0.799405	0.000051
23	C	5.574880	1.560846	-0.000025
24	C	6.529084	0.538115	0.000017
25	H	4.475656	-2.151606	0.000069
26	H	3.505021	2.052028	-0.000064
27	H	6.880096	-1.583278	0.000083
28	H	5.891533	2.593503	-0.000051
29	H	7.580500	0.789145	0.000023
30	N	-1.628243	-1.528426	0.000009
31	N	-1.111117	0.535238	-0.000027
32	H	-2.009708	-2.465130	0.000033
33	H	-1.174869	1.544381	-0.000047

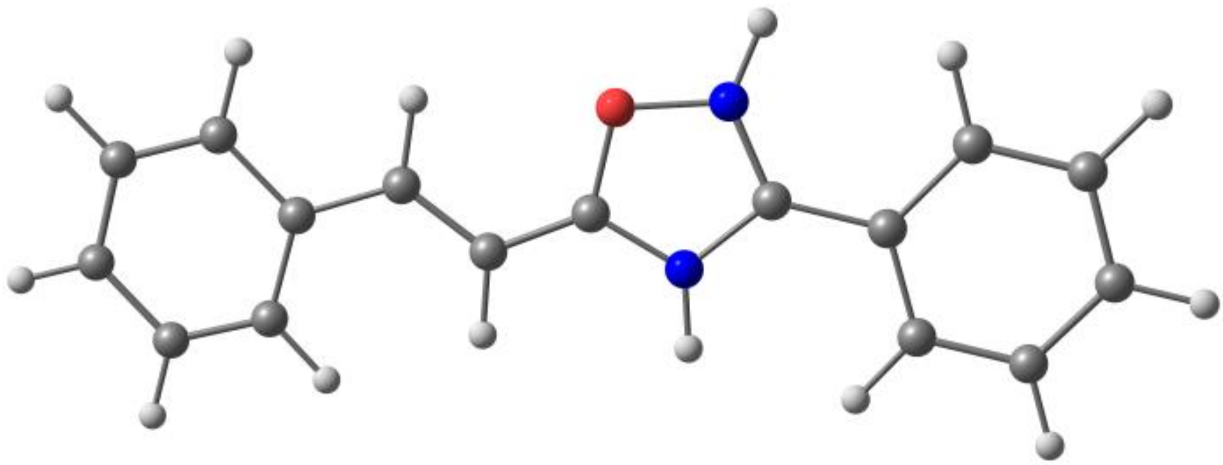

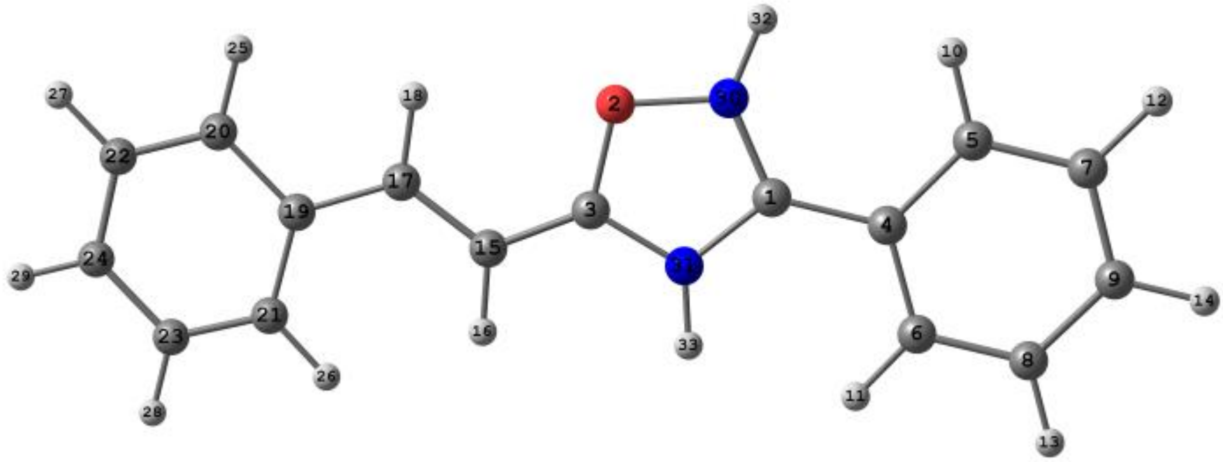

Summary of Natural Population Analysis: Natural charges, e

Atom	No	Natural Charge	Natural Population Core	Valence	Rydberg	Total
C	1	0.54066	1.99916	3.43750	0.02268	5.45934
O	2	-0.25186	1.99968	6.22694	0.02524	8.25186
C	3	0.66078	1.99911	3.31599	0.02411	5.33922
C	4	-0.16390	1.99877	4.13951	0.02561	6.16390
C	5	-0.11103	1.99892	4.09147	0.02064	6.11103
C	6	-0.49353	1.99893	3.29810	1.19650	6.49353
C	7	-0.19540	1.99917	4.17425	0.02197	6.19540
C	8	-0.21573	1.99918	4.18385	0.03270	6.21573
C	9	-0.10885	1.99915	4.09238	0.01733	6.10885
H	10	0.22590	0.00000	0.77206	0.00204	0.77410
H	11	0.21397	0.00000	0.78083	0.00520	0.78603
H	12	0.23501	0.00000	0.76324	0.00175	0.76499
H	13	0.23511	0.00000	0.76313	0.00176	0.76489
H	14	0.23040	0.00000	0.76803	0.00158	0.76960
C	15	-0.31741	1.99884	4.30054	0.01804	6.31741
H	16	0.26248	0.00000	0.73563	0.00189	0.73752
C	17	0.03396	1.99911	3.94721	0.01972	5.96604
H	18	0.23303	0.00000	0.76511	0.00186	0.76697
C	19	-0.13647	1.99902	4.11824	0.01921	6.13647
C	20	-0.09580	1.99892	4.07743	0.01944	6.09580
C	21	-0.12010	1.99907	4.10395	0.01708	6.12010
C	22	-0.21459	1.99917	4.19458	0.02085	6.21459
C	23	-0.20041	1.99915	4.18133	0.01992	6.20041
C	24	-0.11212	1.99917	4.09422	0.01872	6.11212
H	25	0.22556	0.00000	0.77284	0.00160	0.77444
H	26	0.22248	0.00000	0.77572	0.00179	0.77752
H	27	0.22952	0.00000	0.76887	0.00161	0.77048
H	28	0.22876	0.00000	0.76957	0.00167	0.77124
H	29	0.22544	0.00000	0.77312	0.00144	0.77456
N	30	-0.15809	1.99914	5.13733	0.02162	7.15809
N	31	-0.49792	1.99917	5.48144	0.01730	7.49792
H	32	0.48373	0.00000	0.51403	0.00224	0.51627
H	33	0.48738	0.00000	0.51030	0.00232	0.51262

==
* Total *   1.58097   37.98285   90.82875   1.60743   130.41903

Bond lengths, Å

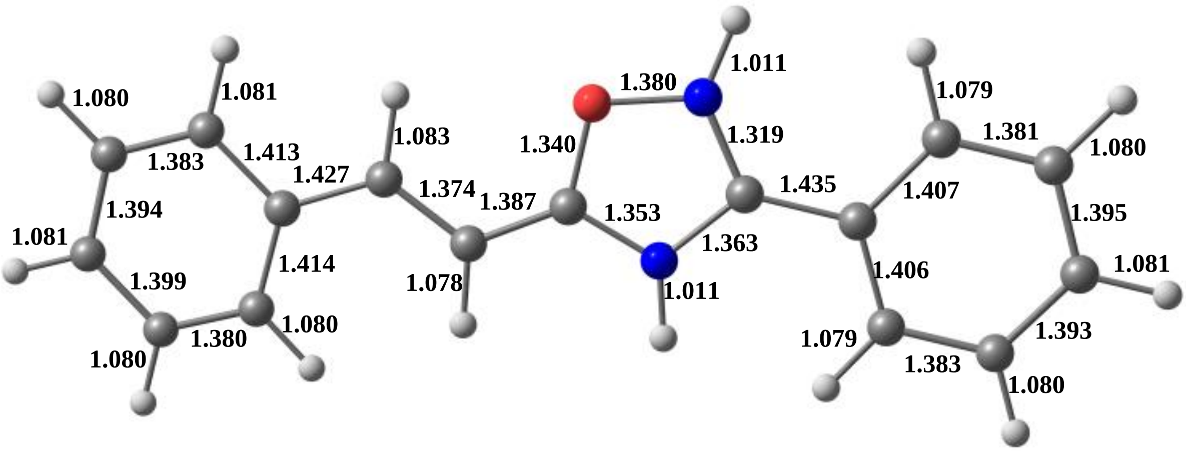

F

E= -803.034494727 h, G^{298}= -802.80029 h, μ= 3.86 D

Optimized geometry, A

N_{at}	Atom	x	y	z
1	C	2.180911	-0.281635	0.099162
2	O	0.204603	-1.277502	0.291287
3	C	-0.001987	0.012423	0.307012
4	C	3.571921	-0.021284	-0.040010
5	C	4.455050	-1.079498	-0.343262
6	C	4.064169	1.287884	0.140104
7	C	5.804718	-0.822074	-0.460850
8	C	5.419265	1.524894	0.023042
9	C	6.286989	0.475372	-0.276328
10	H	4.091124	-2.082962	-0.506700
11	H	3.409657	2.109308	0.390240
12	H	6.484297	-1.625449	-0.700735
13	H	5.801717	2.523753	0.167165
14	H	7.345990	0.668641	-0.368742
15	C	-1.357660	0.587601	0.464862
16	H	-1.443633	1.444050	-0.204761
17	C	-2.472483	-0.393666	0.318684
18	H	-2.232181	-1.440265	0.457040
19	C	-3.784038	-0.085876	0.071967
20	C	-4.713469	-1.183685	0.018087
21	C	-4.269554	1.252299	-0.124898
22	C	-6.042979	-0.950710	-0.216096
23	C	-5.601201	1.462144	-0.351992
24	C	-6.483525	0.366573	-0.399201
25	H	-4.343440	-2.187463	0.167046
26	H	-3.591457	2.090792	-0.093194
27	H	-6.746341	-1.767729	-0.259447
28	H	-5.980814	2.461297	-0.499408
29	H	-7.532932	0.548920	-0.584663
30	N	1.563522	-1.449977	0.165427
31	N	1.153194	0.640370	0.191891
32	H	1.264960	1.648403	0.153377
33	H	-1.392693	1.007158	1.483549
34	H	1.875134	-2.415778	0.186017

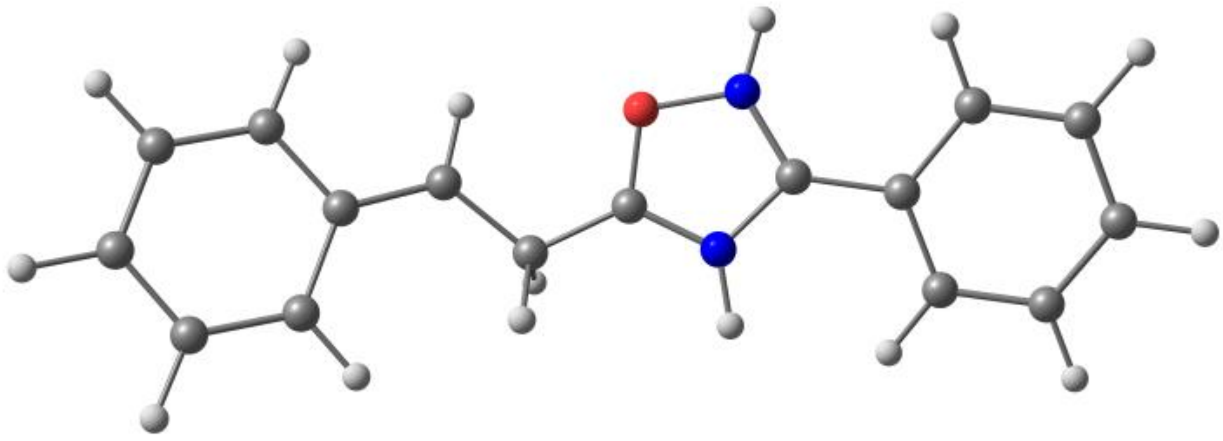

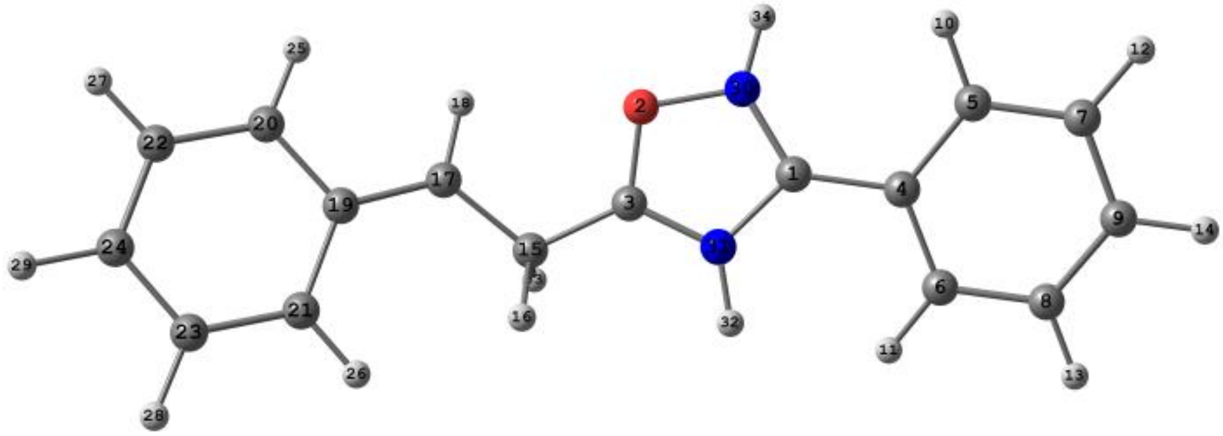

Summary of Natural Population Analysis: Natural charges, e

Atom	No	Natural Charge	Core	Valence	Rydberg	Total
C	1	0.52212	1.99914	3.43460	0.04413	5.47788
O	2	-0.20421	1.99964	6.17868	0.02589	8.20421
C	3	0.77623	1.99929	3.19082	0.03366	5.22377
C	4	-0.53680	1.99897	4.32541	0.21242	6.53680
C	5	-0.13139	1.99893	4.07907	0.05339	6.13139
C	6	0.26003	1.99897	2.54916	1.19184	5.73997
C	7	-0.25847	1.99916	4.16573	0.09357	6.25847
C	8	-0.64273	1.99918	4.39396	0.24958	6.64273
C	9	-0.15124	1.99915	4.07350	0.07859	6.15124
H	10	0.22532	0.00000	0.76703	0.00765	0.77468
H	11	0.02324	0.00000	0.83992	0.13684	0.97676
H	12	0.23824	0.00000	0.75882	0.00294	0.76176
H	13	0.23073	0.00000	0.76069	0.00858	0.76927
H	14	0.23119	0.00000	0.76506	0.00376	0.76881
C	15	-0.54752	1.99909	4.52786	0.02057	6.54752
H	16	0.31741	0.00000	0.68030	0.00229	0.68259
C	17	0.16218	1.99903	3.82171	0.01709	5.83782
H	18	0.24688	0.00000	0.75117	0.00195	0.75312
C	19	-0.14059	1.99898	4.12219	0.01942	6.14059
C	20	-0.01477	1.99910	3.99770	0.01796	6.01477
C	21	-0.04739	1.99908	4.03145	0.01686	6.04739
C	22	-0.21763	1.99914	4.19849	0.02000	6.21763
C	23	-0.20240	1.99915	4.18341	0.01984	6.20240
C	24	0.01563	1.99920	3.96682	0.01835	5.98437
H	25	0.24161	0.00000	0.75679	0.00160	0.75839
H	26	0.23762	0.00000	0.76065	0.00174	0.76238
H	27	0.24794	0.00000	0.75047	0.00159	0.75206
H	28	0.24691	0.00000	0.75153	0.00157	0.75309
H	29	0.23891	0.00000	0.75982	0.00127	0.76109
N	30	-0.14918	1.99914	5.12210	0.02793	7.14918
N	31	-0.48382	1.99914	5.45387	0.03082	7.48382
H	32	0.50297	0.00000	0.49195	0.00508	0.49703
H	33	0.35067	0.00000	0.64766	0.00167	0.64933

H 34 0.49802 0.00000 0.49964 0.00234 0.50198
===
* Total * 2.08568 37.98350 90.55805 2.37277 130.91432

Bond lengths, Å

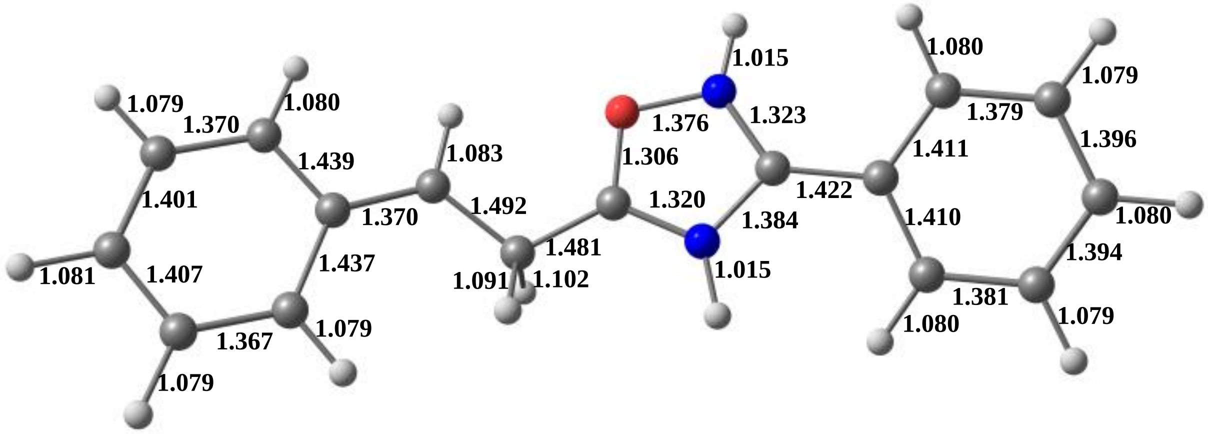

Supplement: File 1 — Experimental part, NMR spectra and DFT calculations. [file Beilstein_J_Org_Chem-13-883-s001.pdf]
